# Supplementary material for: Cobalt-catalyzed difluoroalkylation of tertiary aryl ketones for facile synthesis of quaternary alkyl difluorides
Source: Nat Commun. 2018 Nov 23;9:4951. doi: 10.1038/s41467-018-07525-y (PMC6251884; doi:10.1038/s41467-018-07525-y)
Supplement: Supplementary file 1 — Supplementary Information [file 41467_2018_7525_MOESM1_ESM.pdf]

**Cobalt-Catalyzed Difluoroalkyaltion of Tertiary Aryl Ketones for Facile Synthesis of Quaternary Alkyl Difluorides**

Li *et al.*

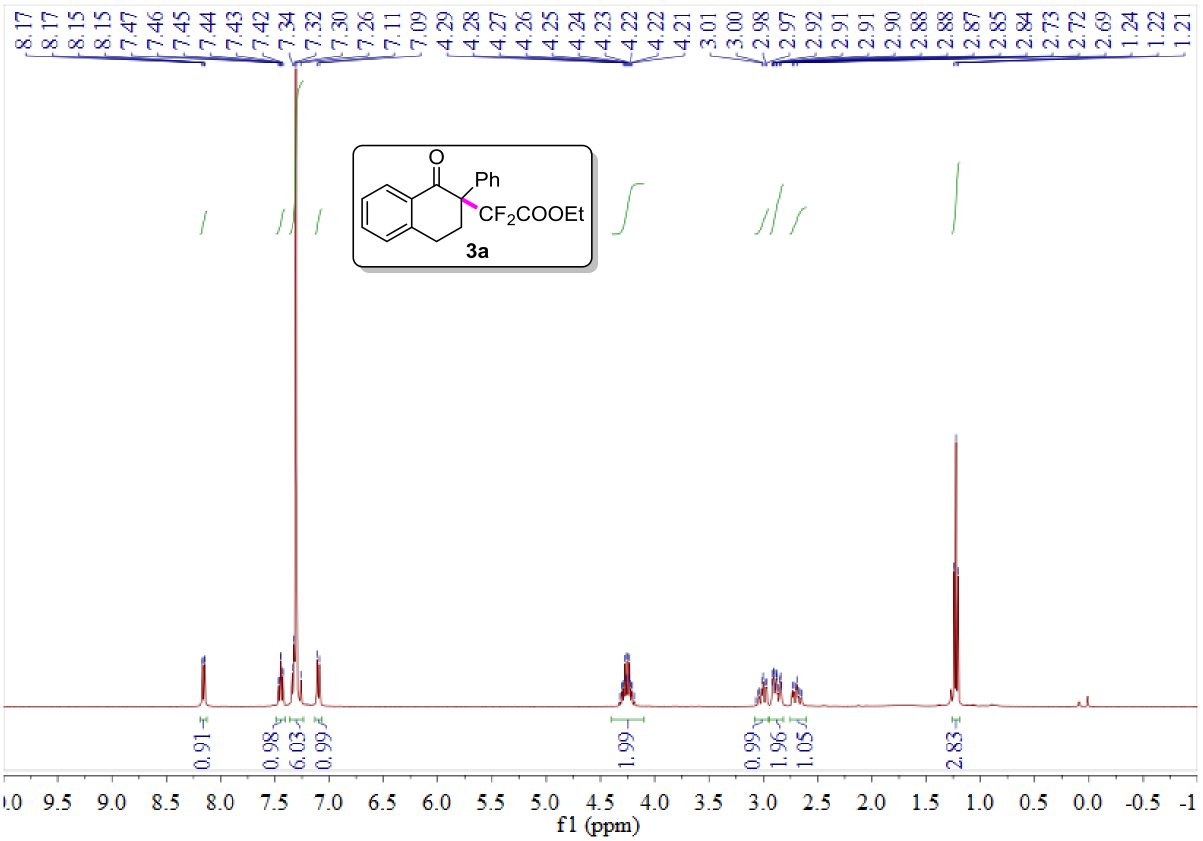


Supplementary Figure 1. $^{1}H$ NMR of 3a

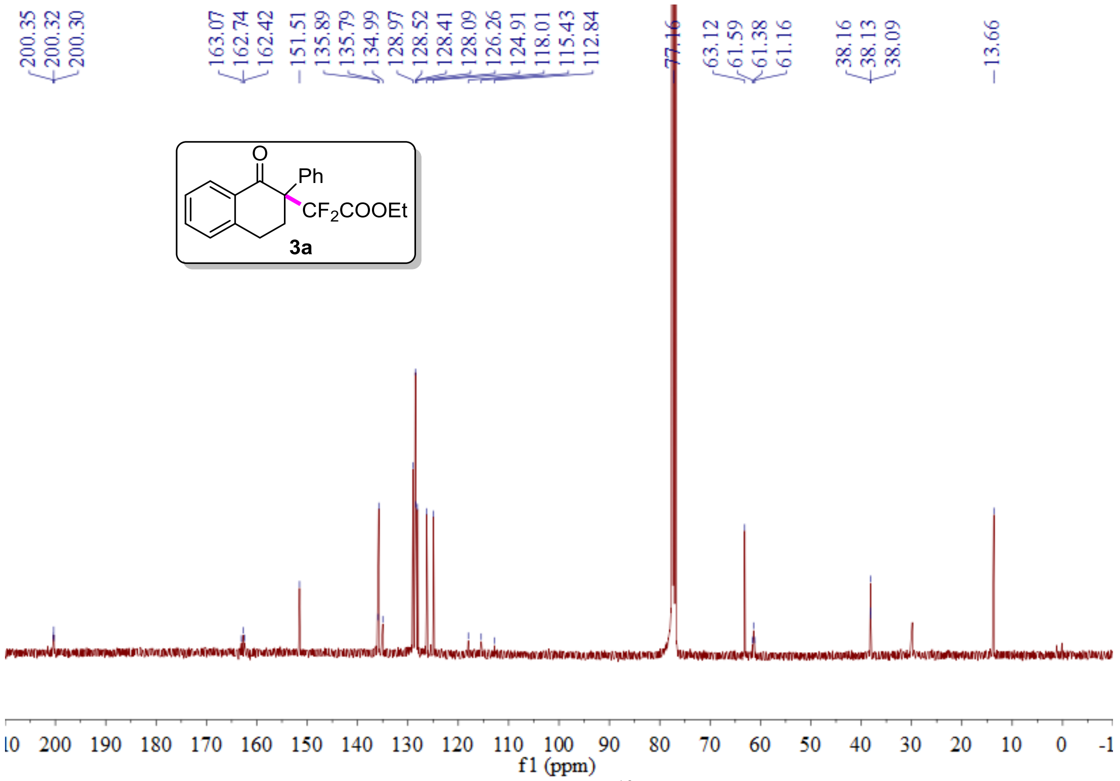


Supplementary Figure 2. $^{13}C$ NMR of 3a

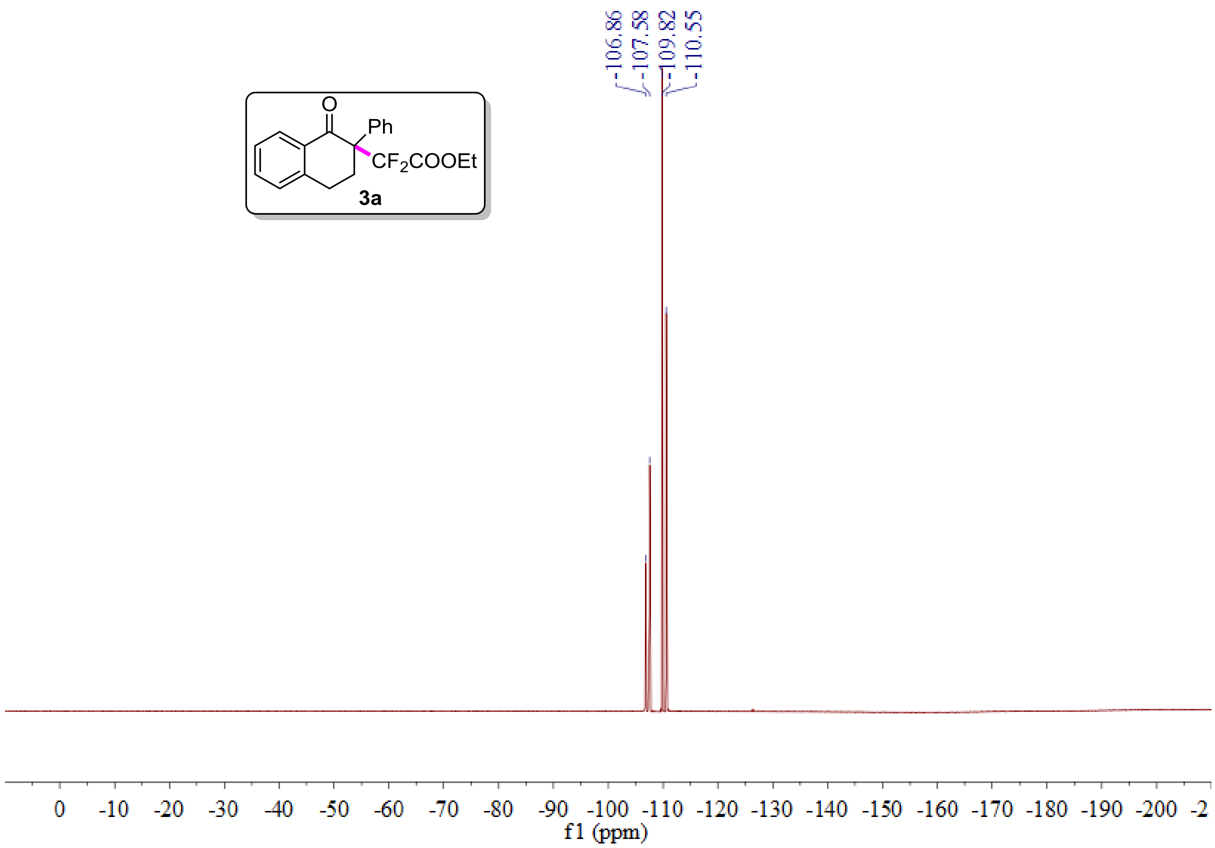


Supplementary Figure 3. $^{19}$F NMR of 3a

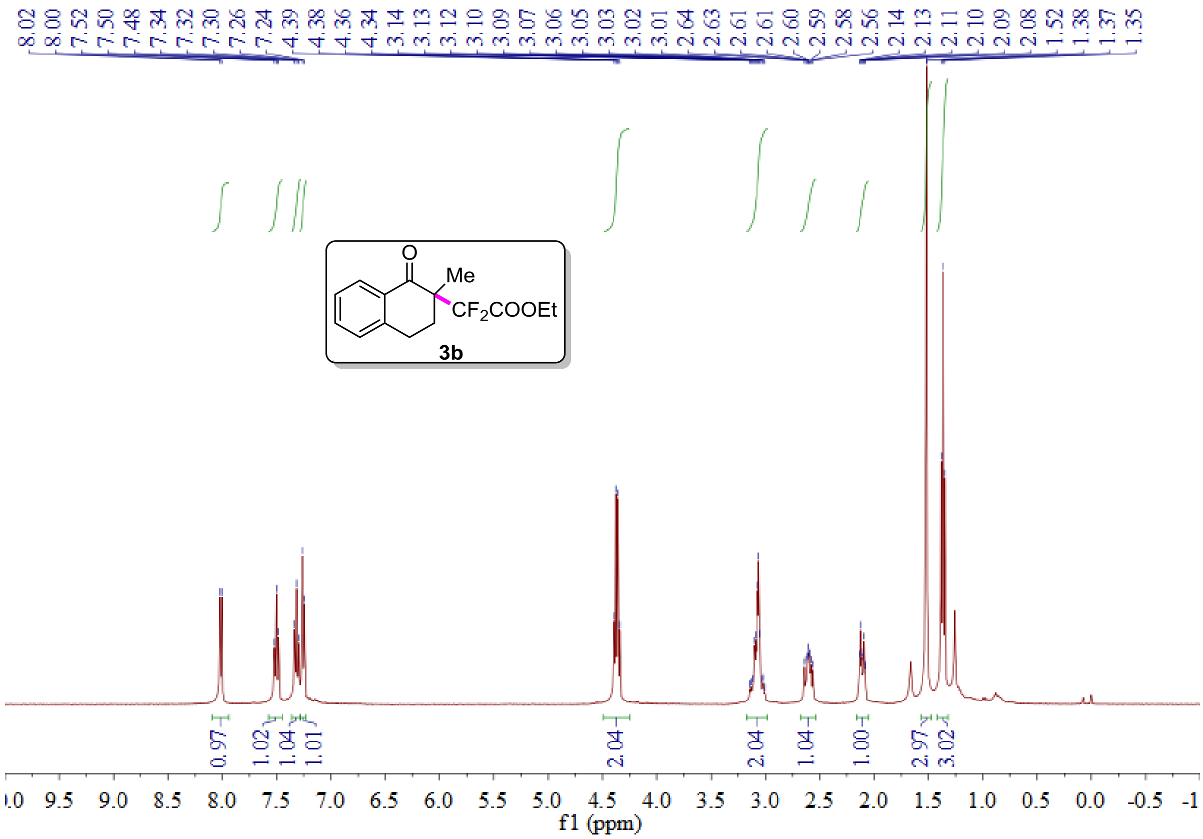


Supplementary Figure 4. $^{1}$H NMR of 3b

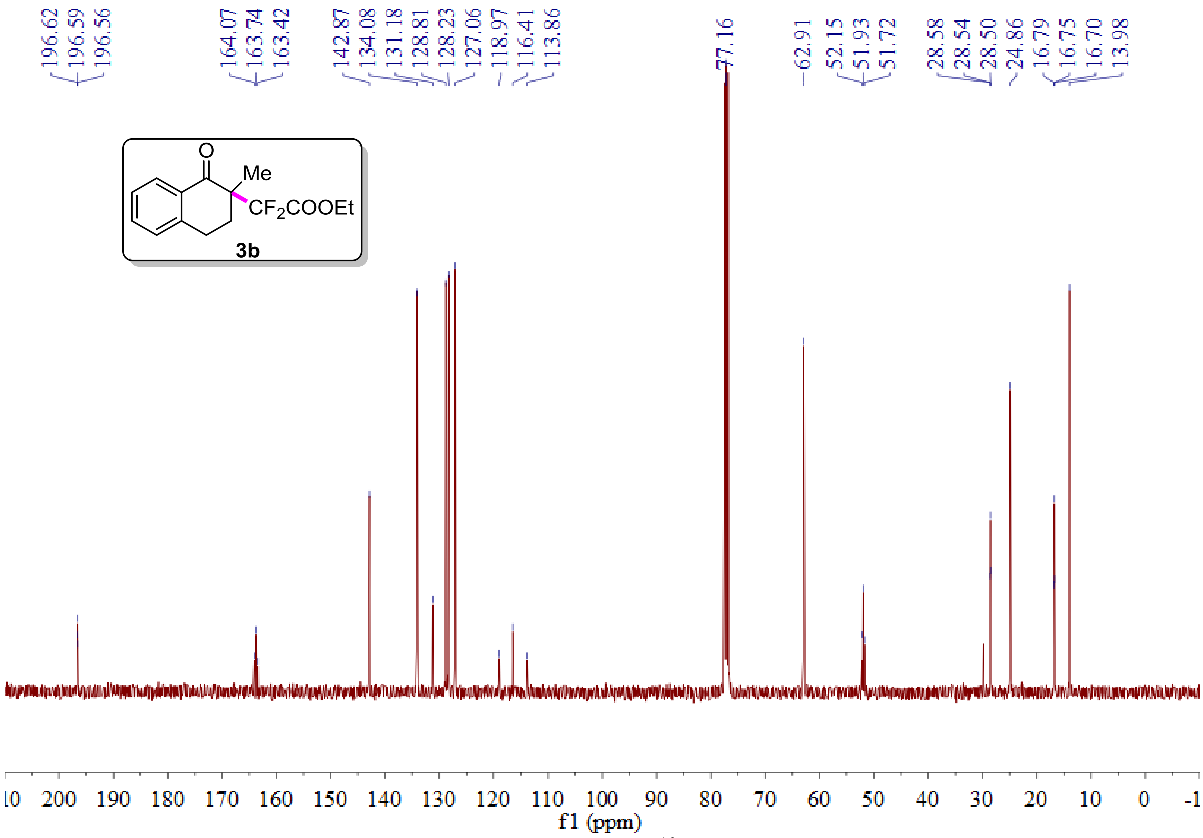

Supplementary Figure 5. $^{13}C$ NMR of 3b

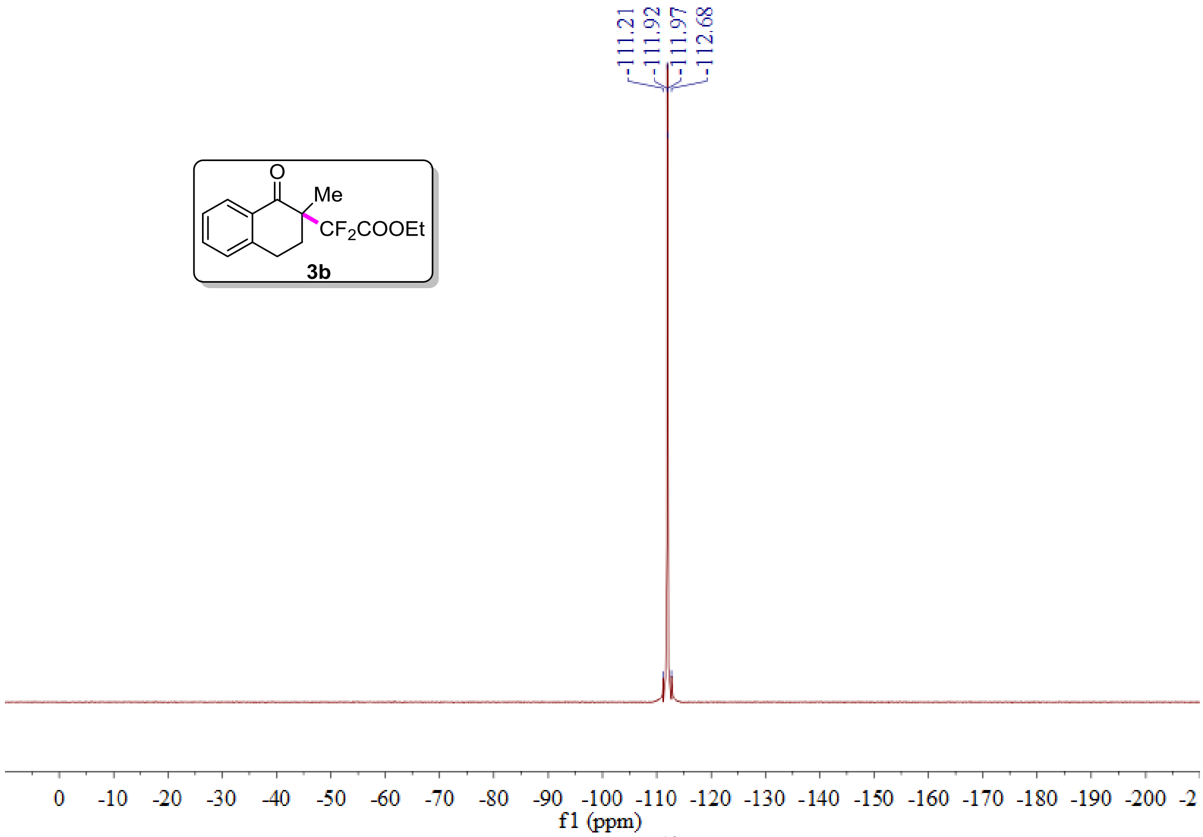

Supplementary Figure 6. $^{19}F$ NMR of 3b

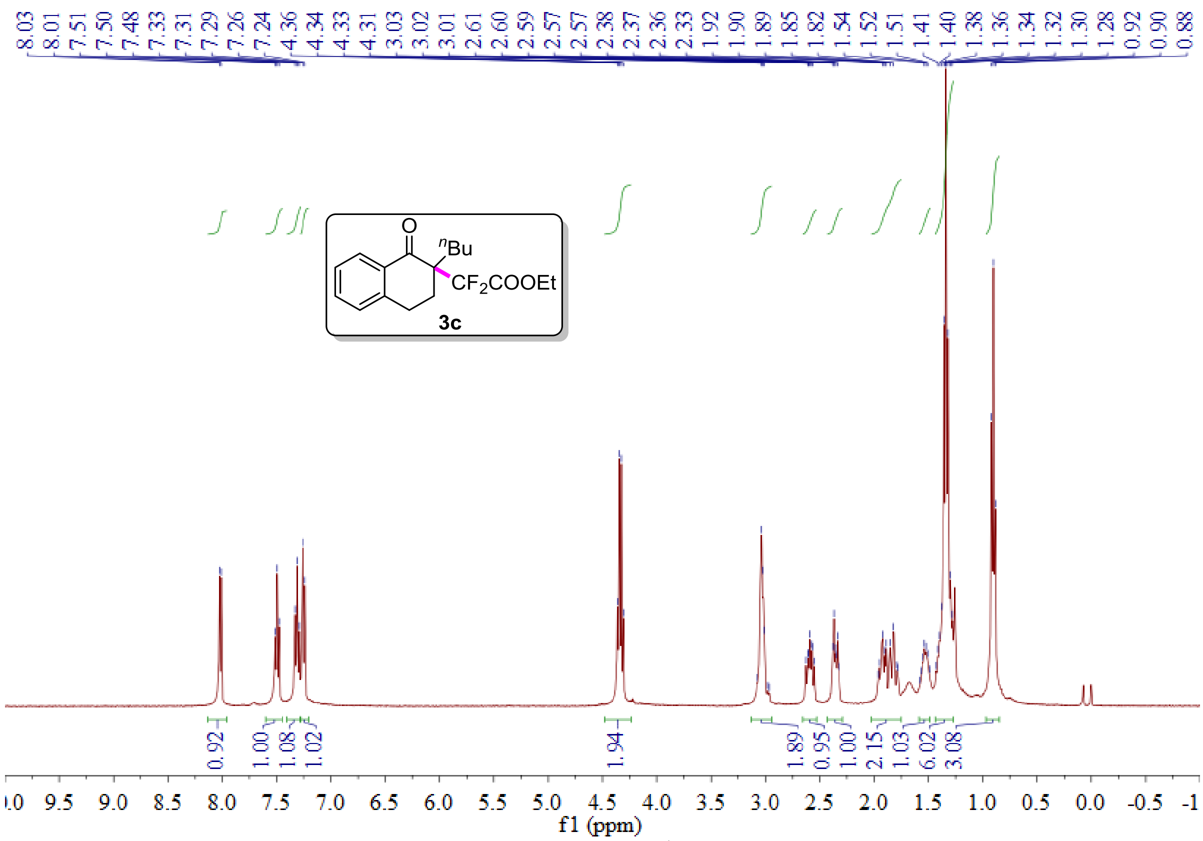

Supplementary Figure 7. $^{1}H$ NMR of 3c

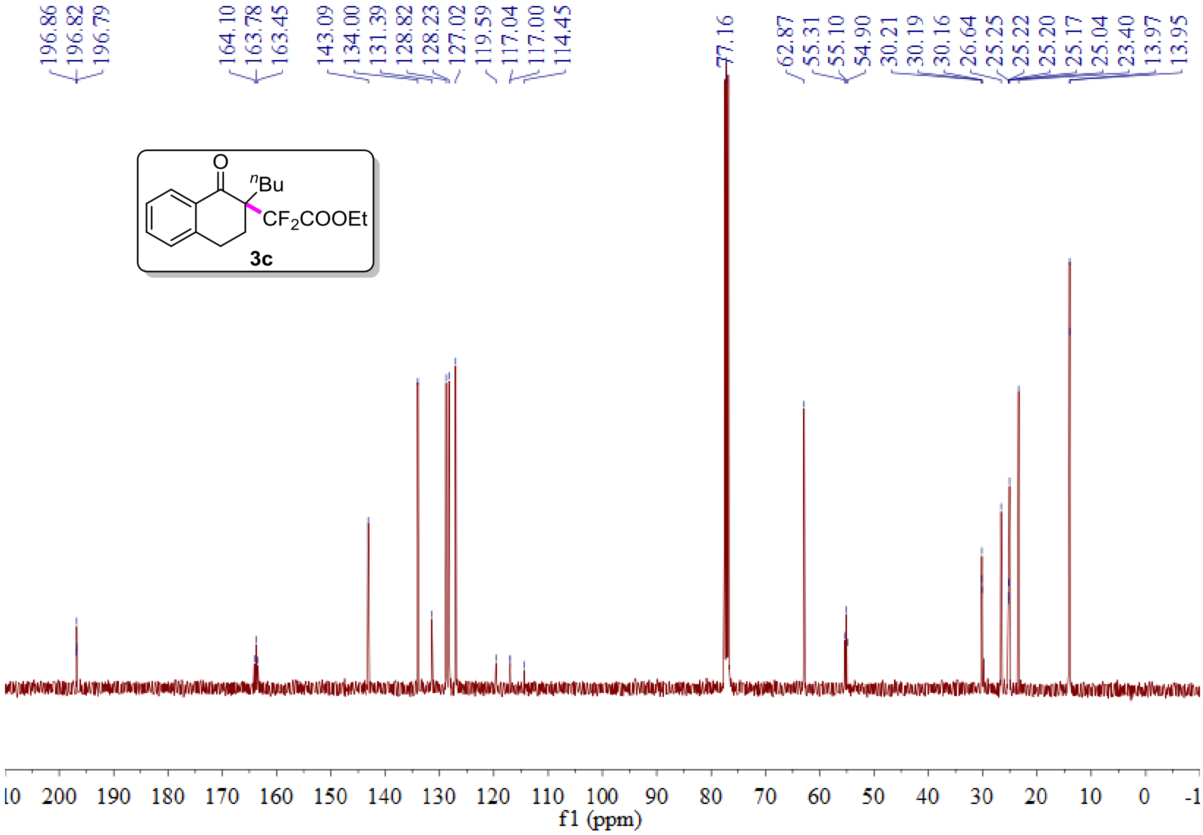

Supplementary Figure 8. $^{13}C$ NMR of 3c

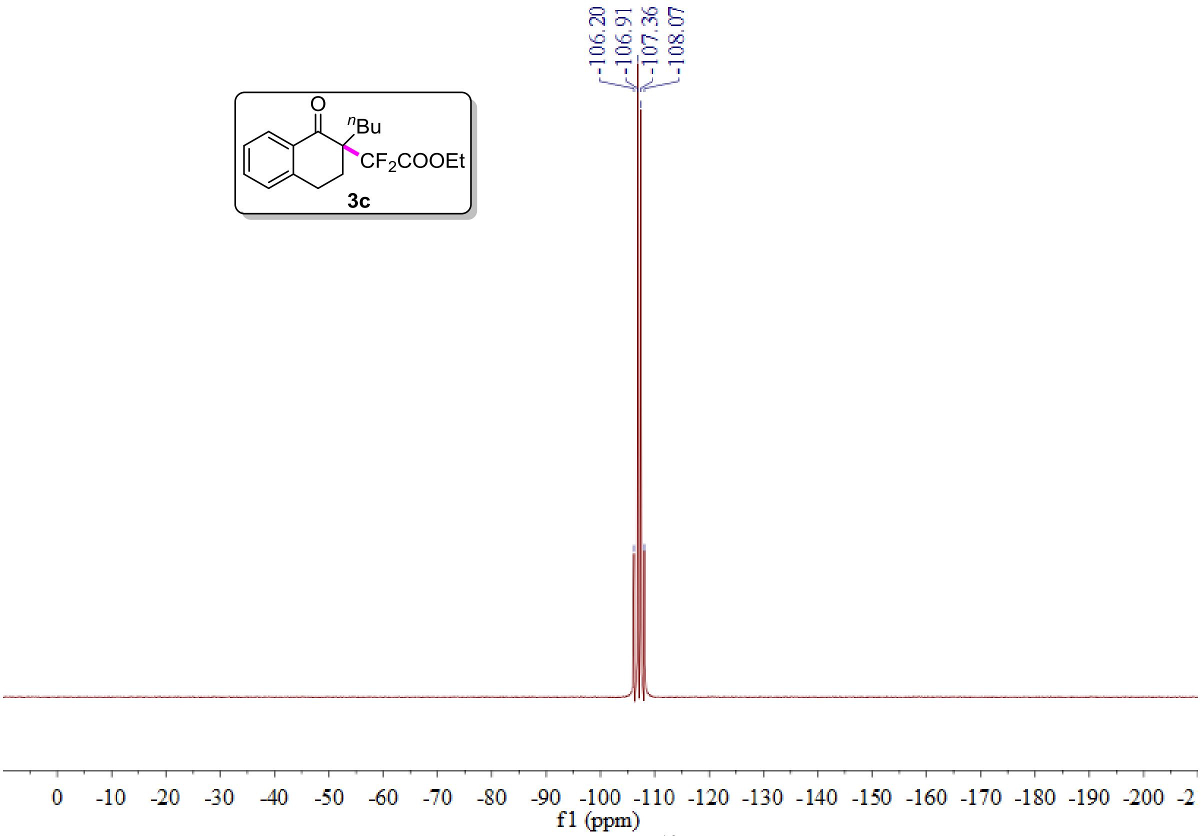

Supplementary Figure 9. $^{19}F$ NMR of 3c

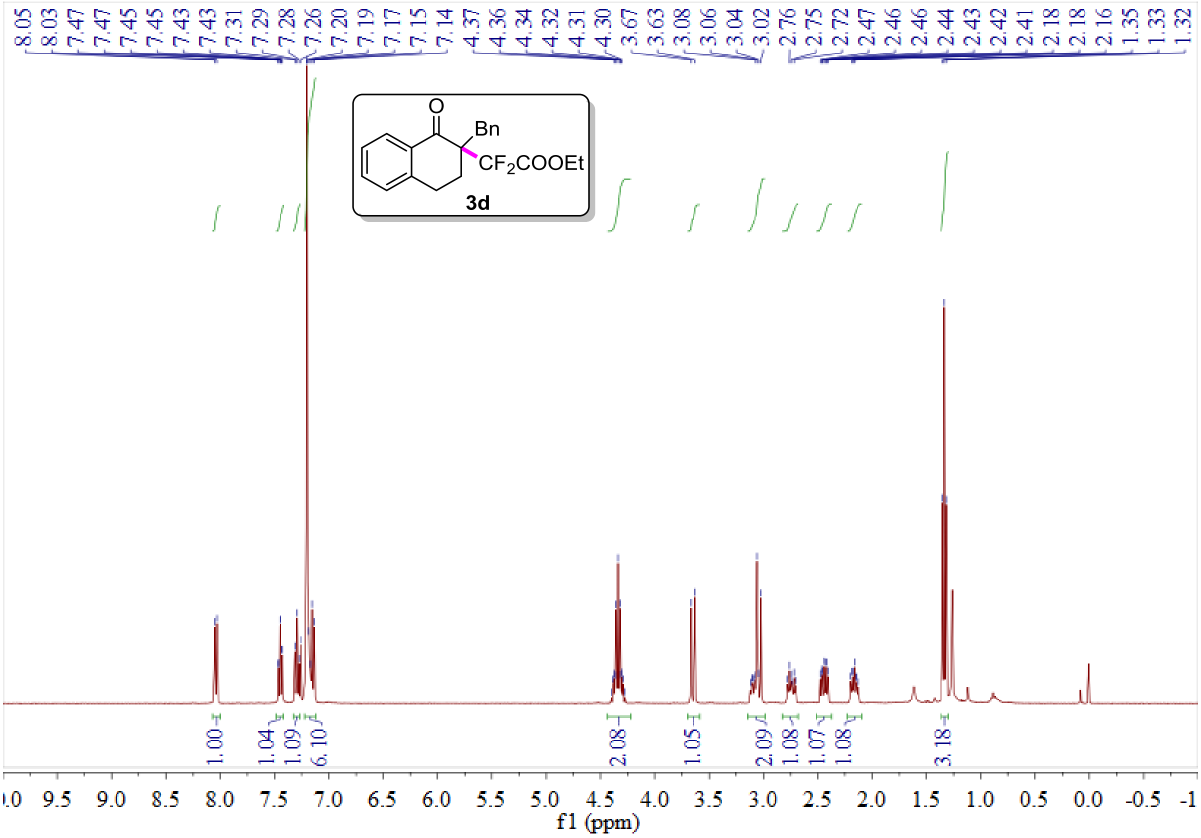

Supplementary Figure 10. $^{1}H$ NMR of 3d

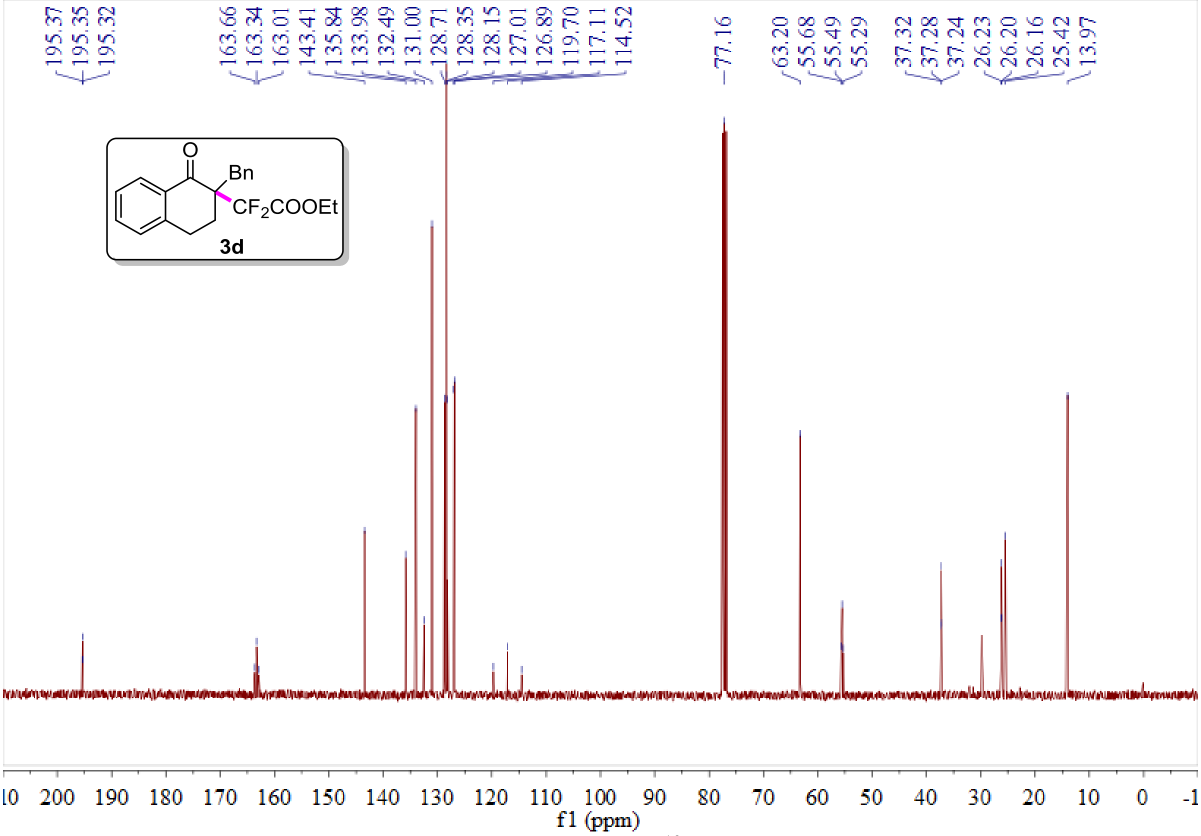


Supplementary Figure 11. $^{13}C$ NMR of 3d

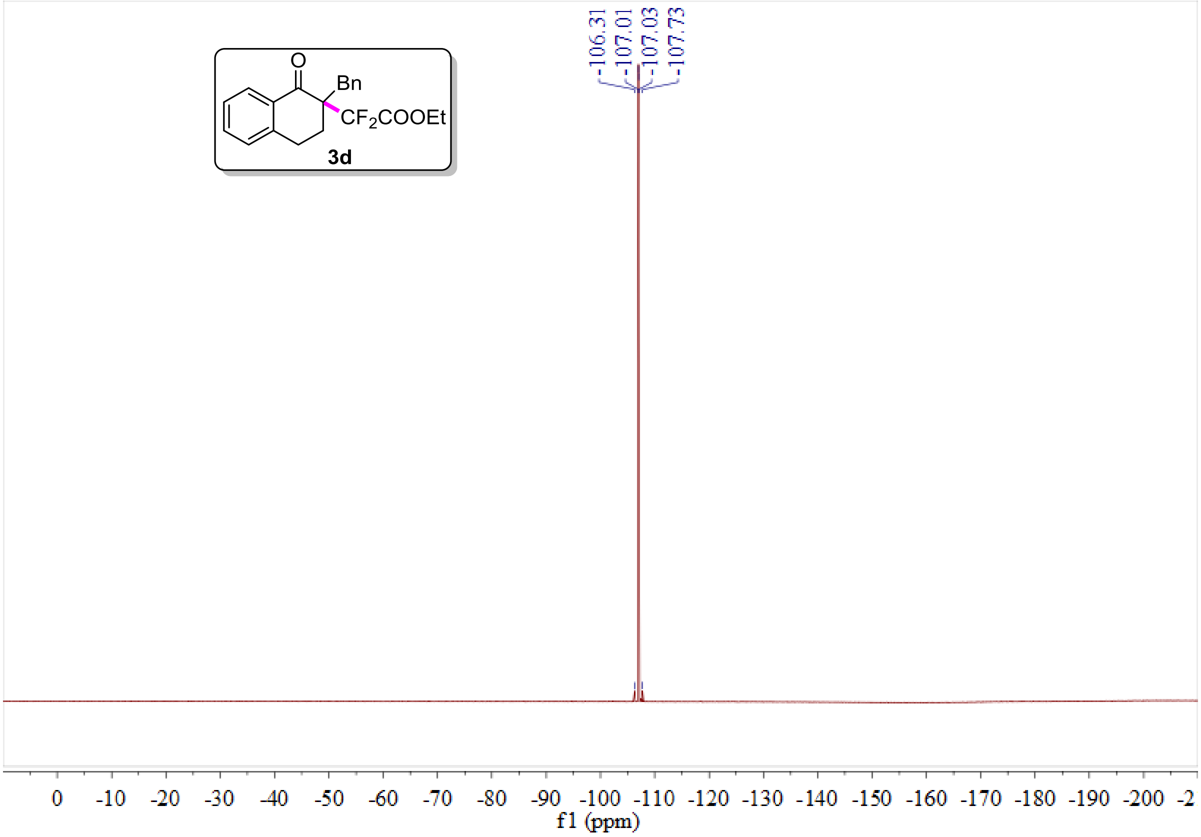


Supplementary Figure 12. $^{19}F$ NMR of 3d

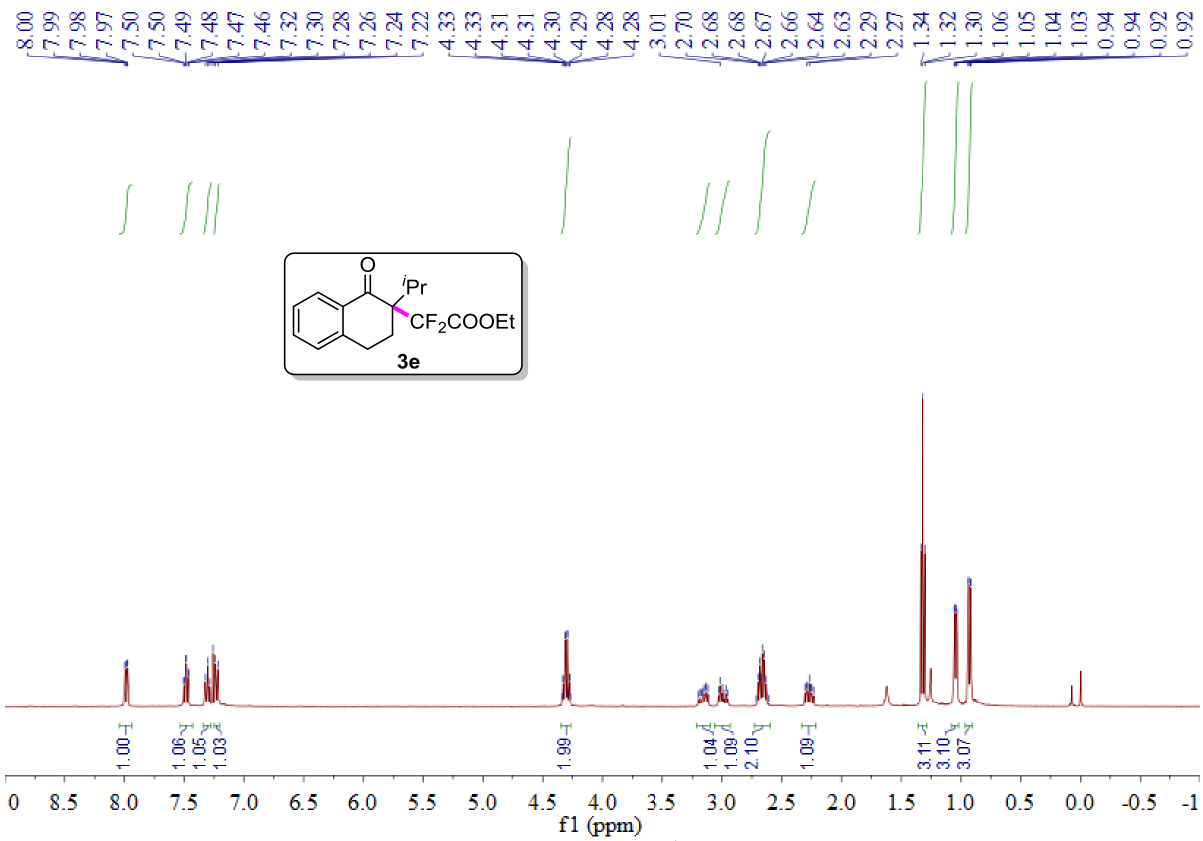

Supplementary Figure 13. $^{1}H$ NMR of 3e

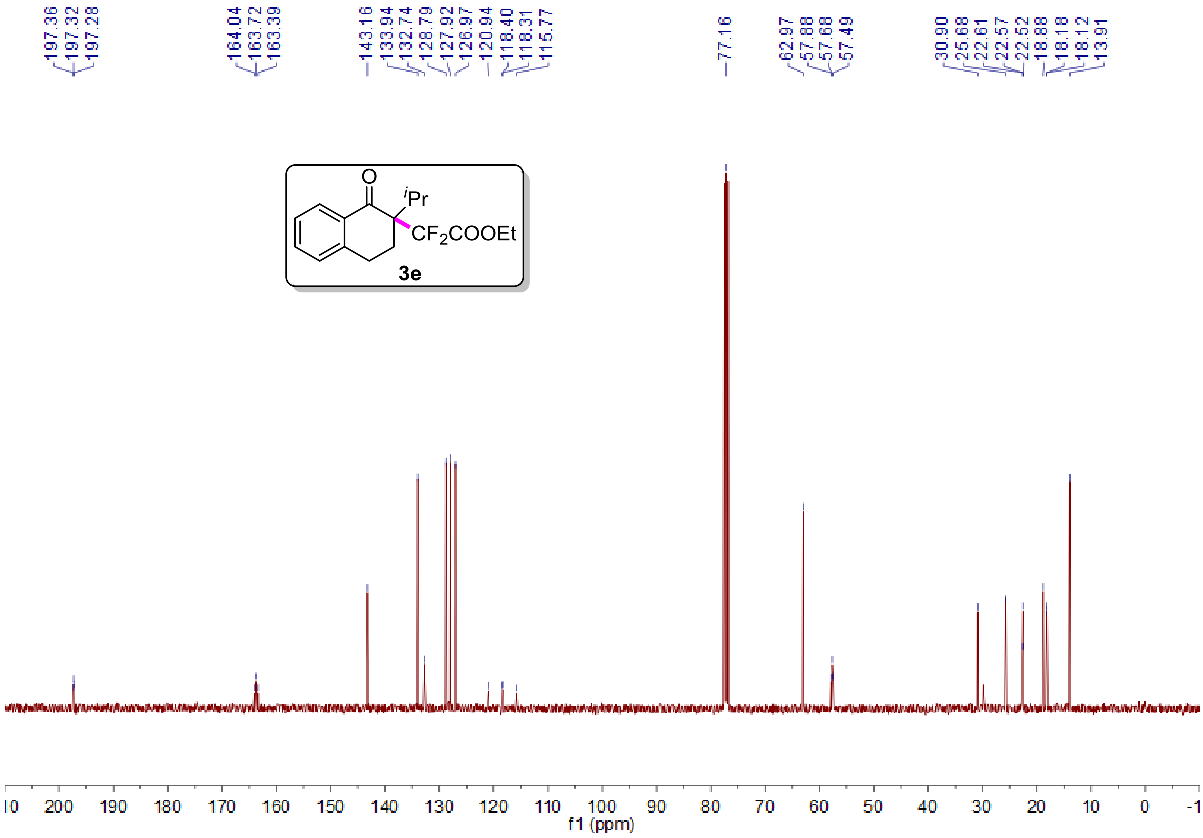

Supplementary Figure 14. $^{13}C$ NMR of 3e

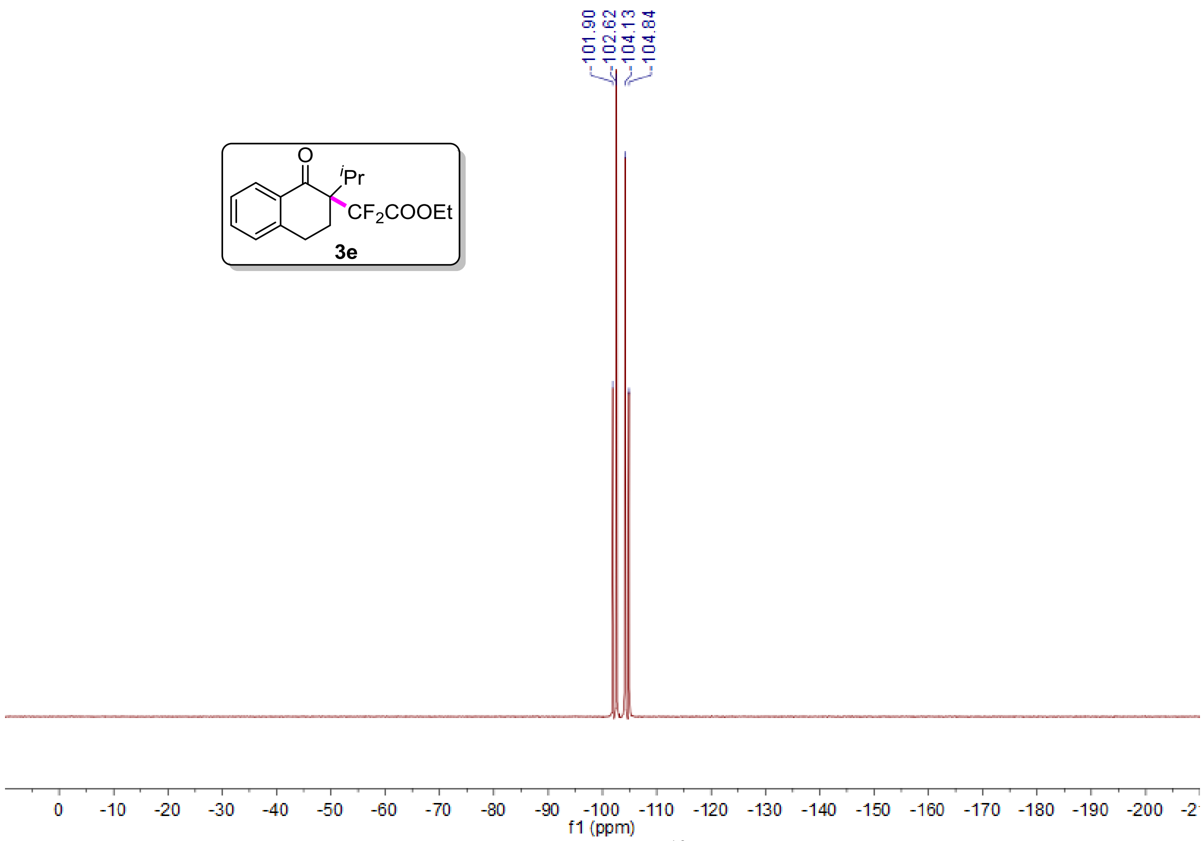

Supplementary Figure 15. $^{19}F$ NMR of 3e

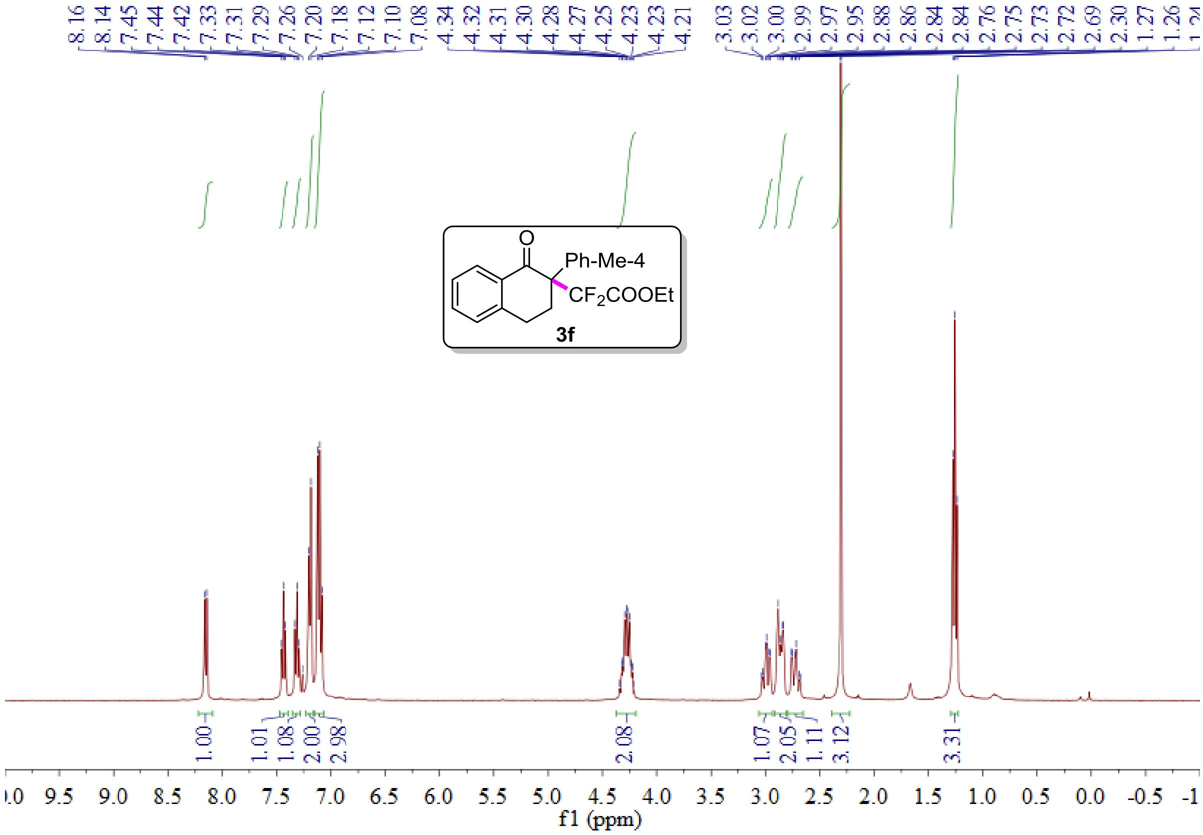

Supplementary Figure 16. $^{1}H$ NMR of 3f

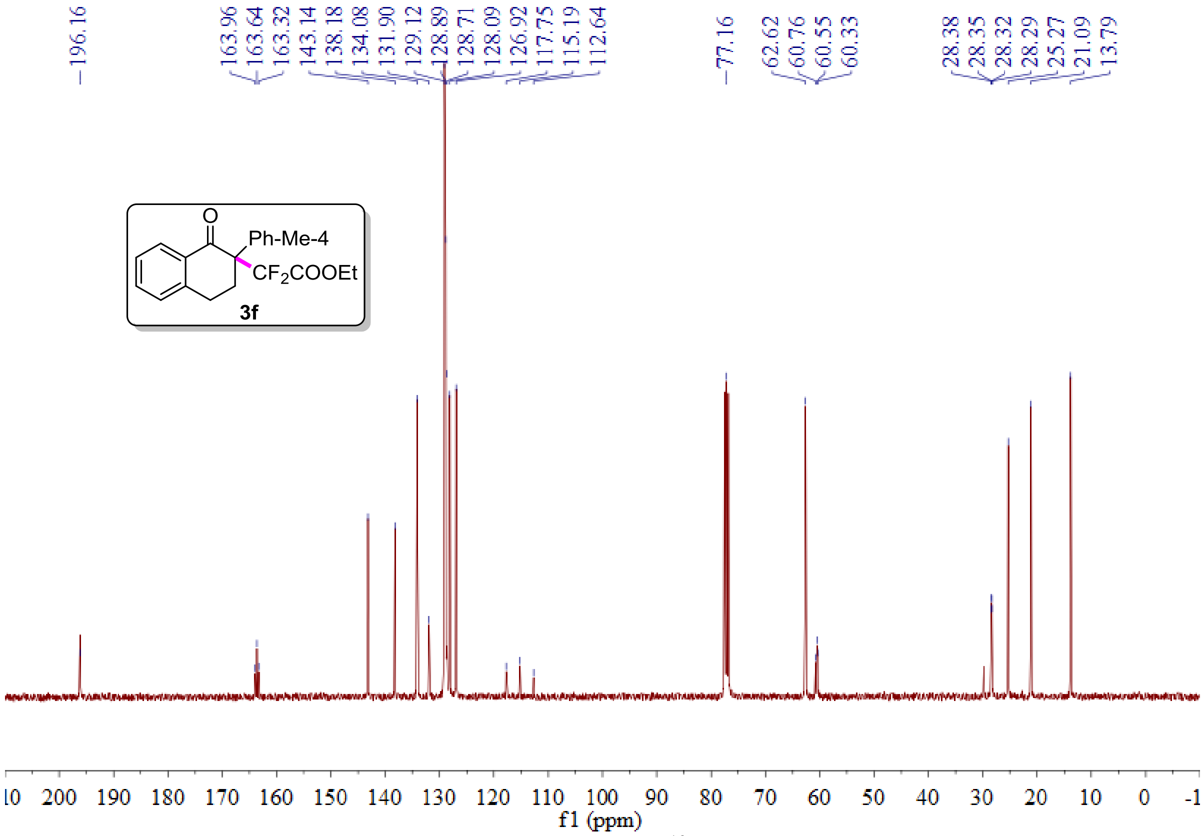


Supplementary Figure 17. $^{13}$C NMR of 3f

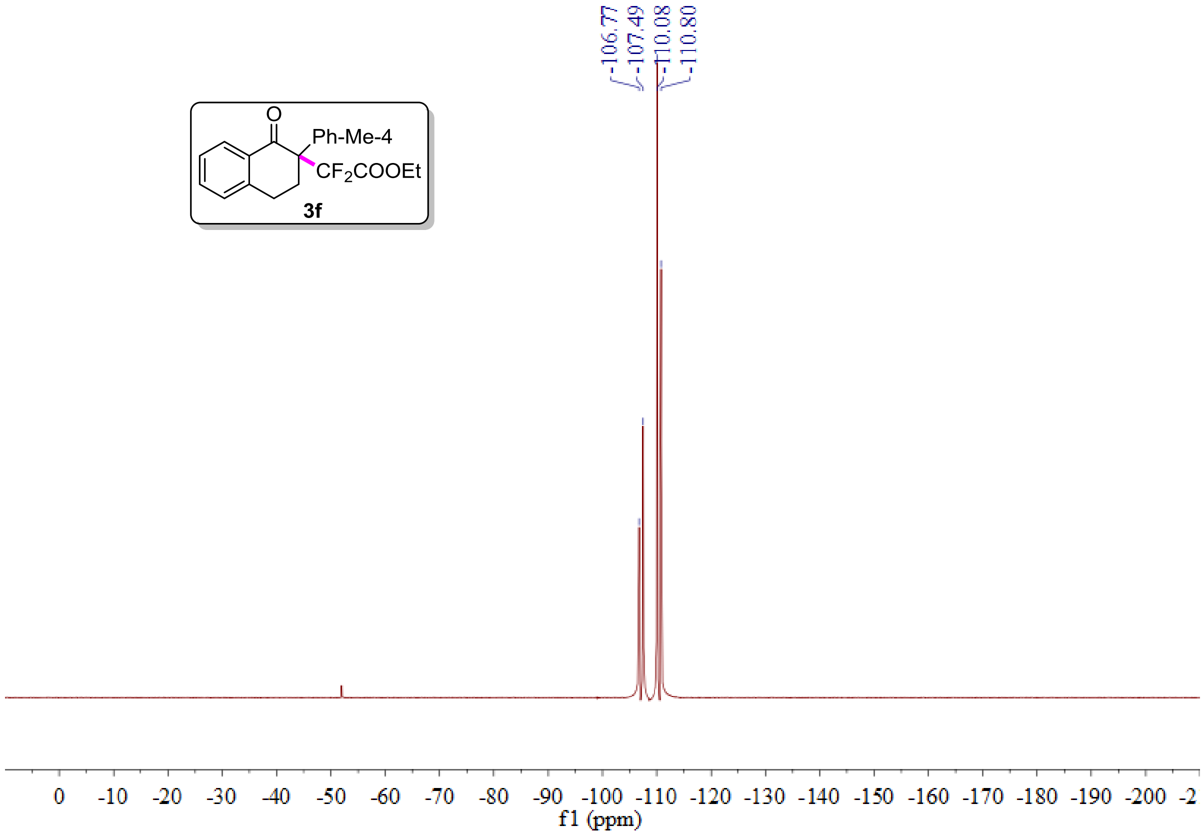


Supplementary Figure 18. $^{19}$F NMR of 3f

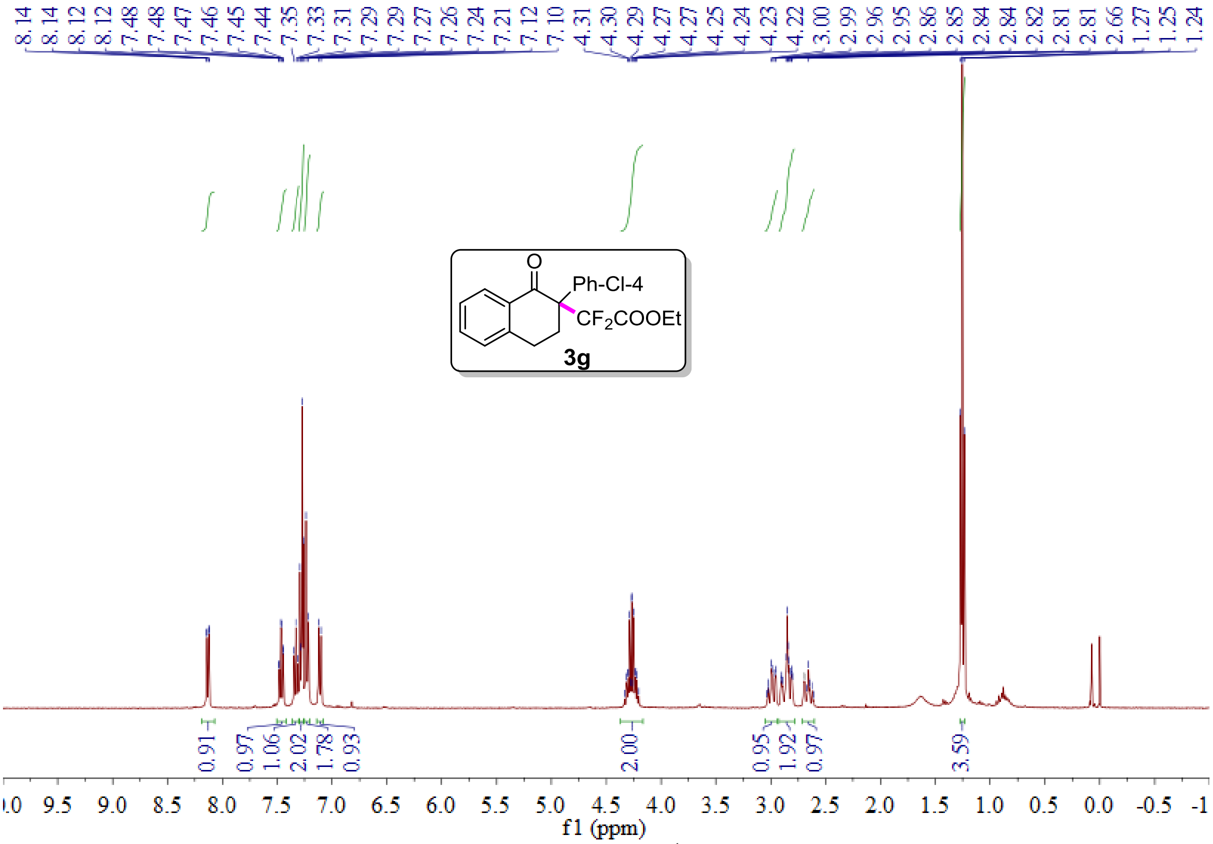

Supplementary Figure 19. $^{1}$H NMR of 3g

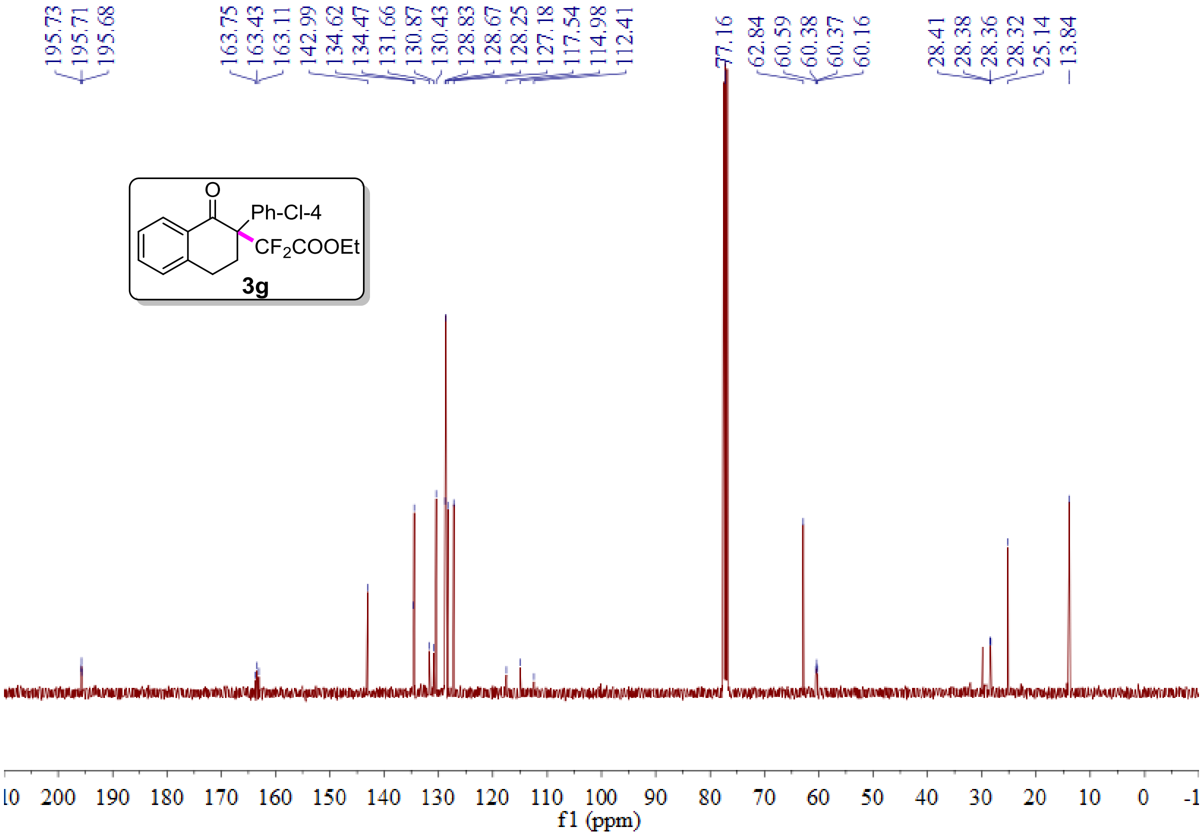

Supplementary Figure 20. $^{13}$C NMR of 3g

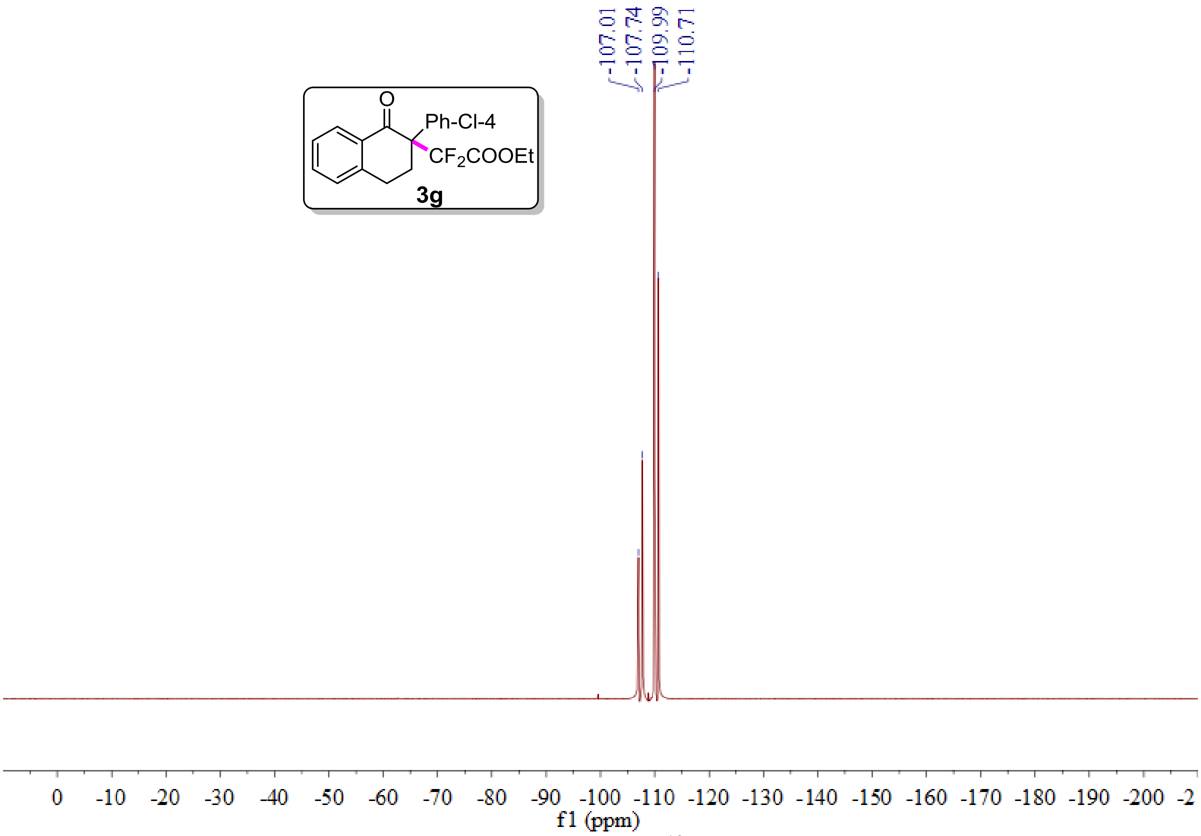

Supplementary Figure 21. $^{19}$F NMR of 3g

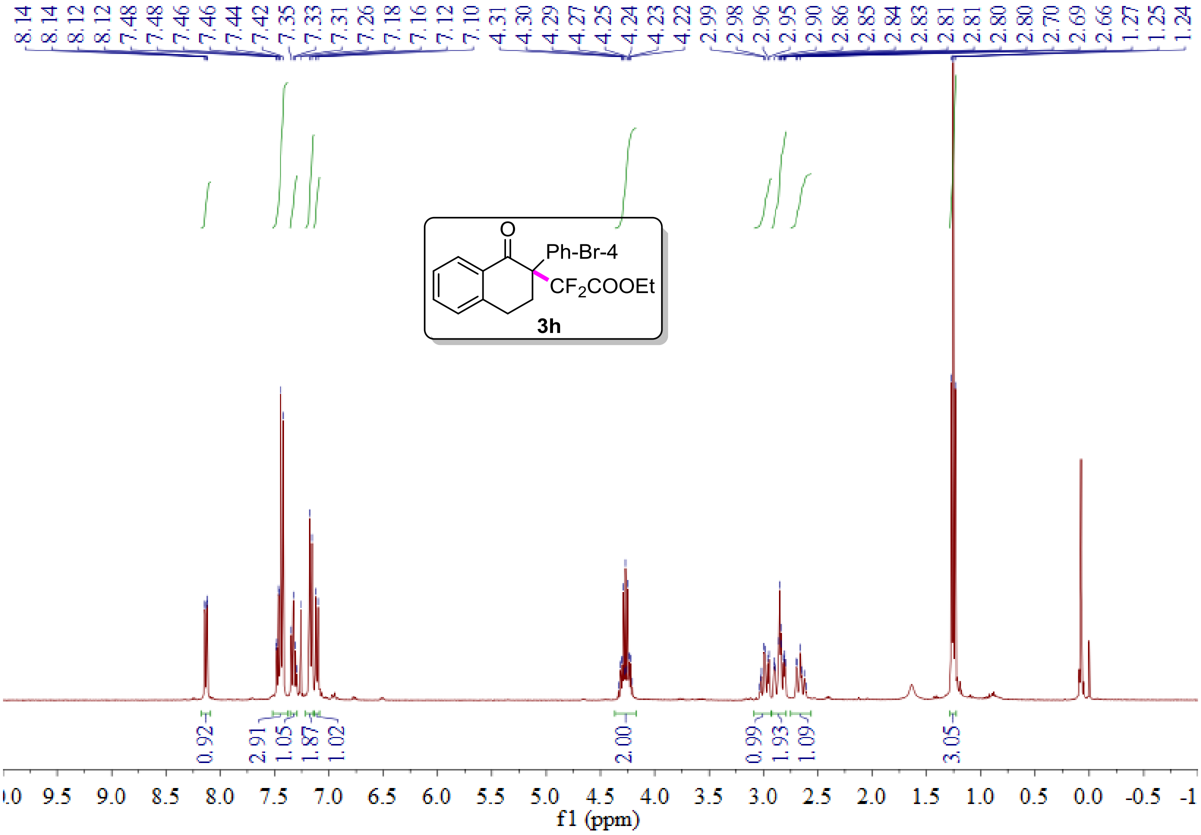

Supplementary Figure 22. $^{1}$H NMR of 3h

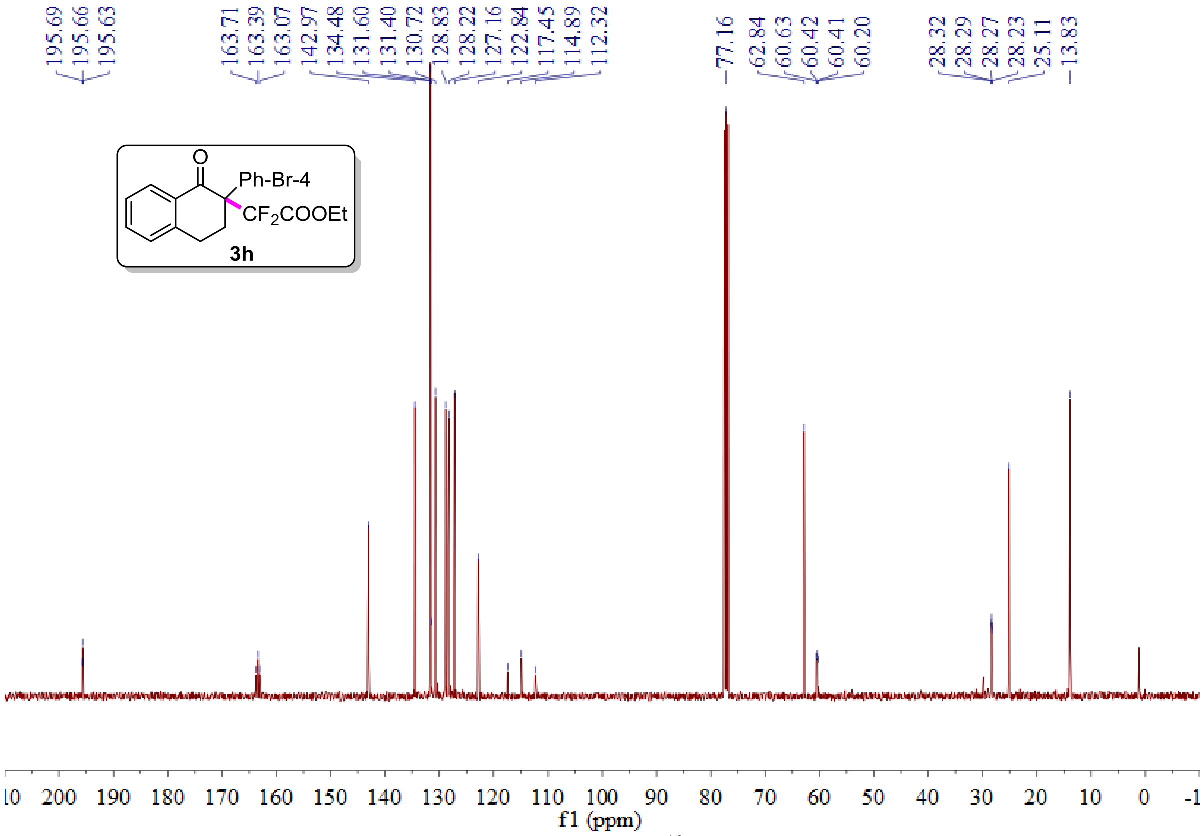

Supplementary Figure 23. $^{13}C$ NMR of 3h

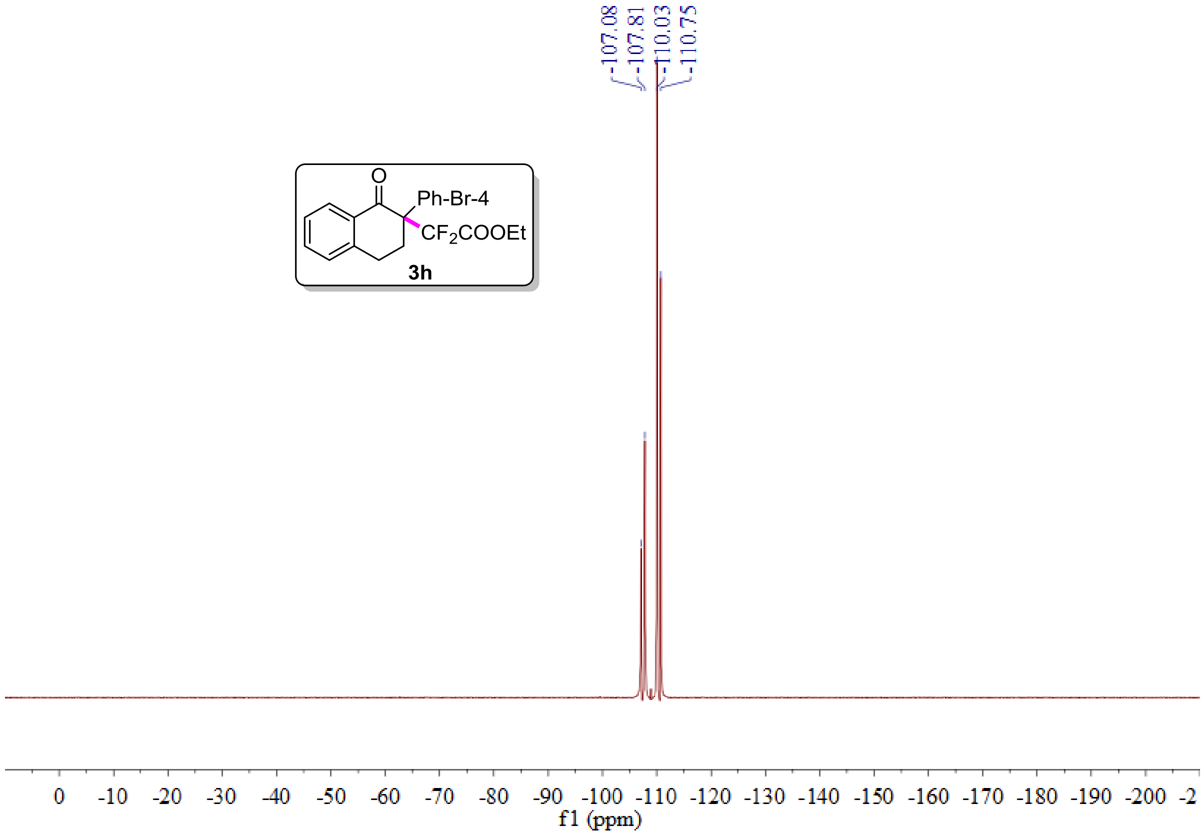

Supplementary Figure 24. $^{19}F$ NMR of 3h

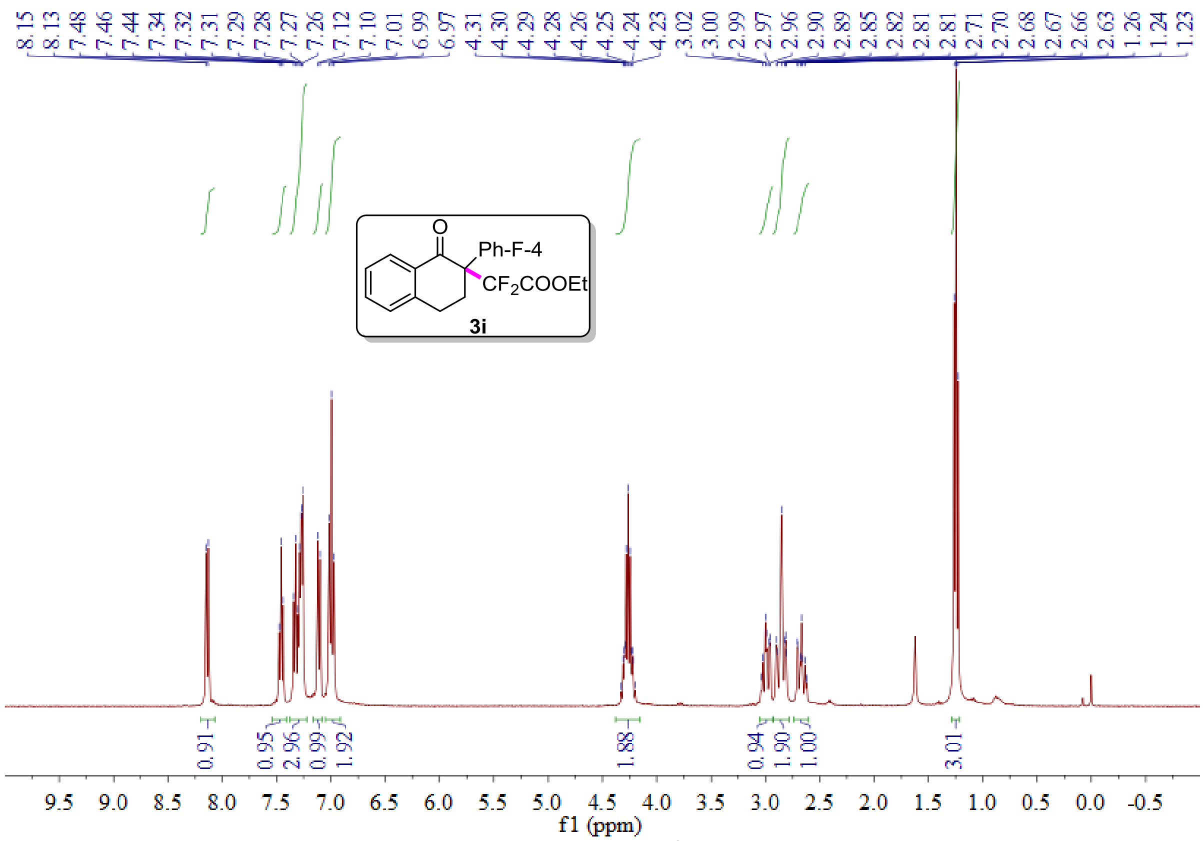

Supplementary Figure 25. $^{1}$H NMR of 3i

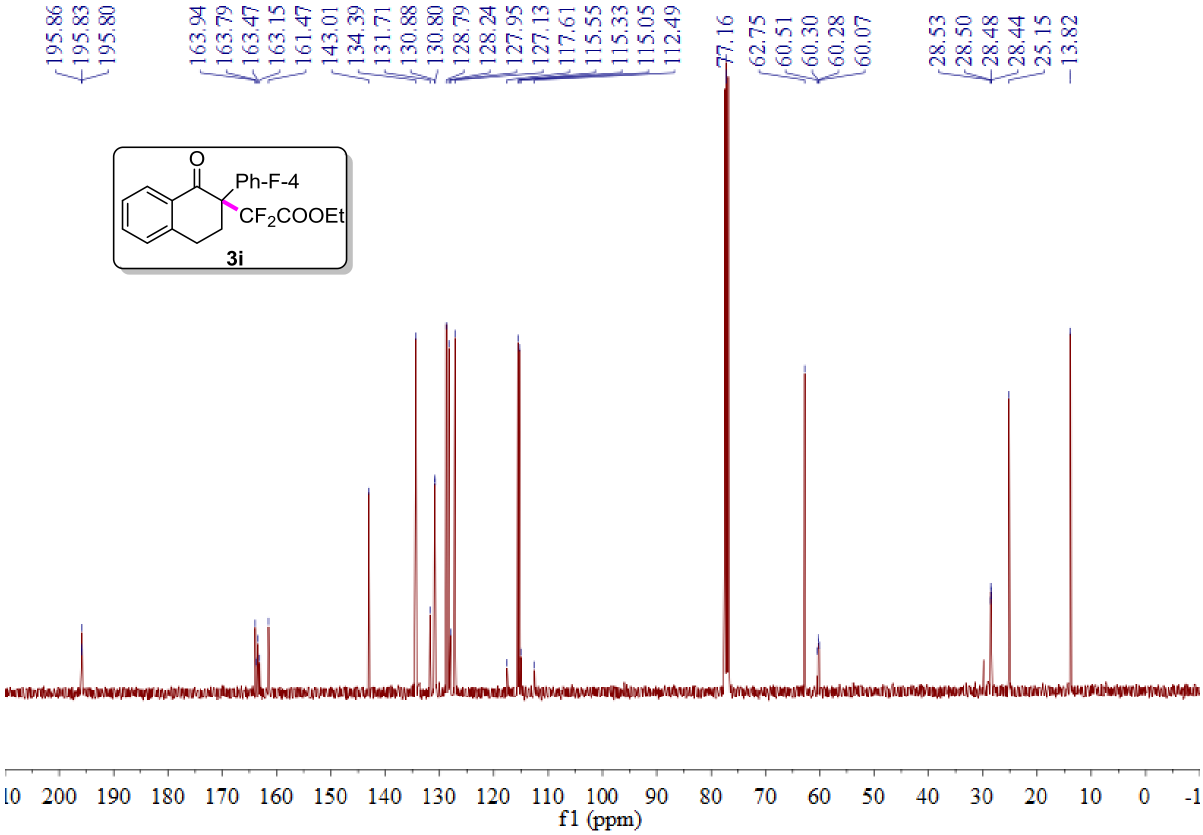

Supplementary Figure 26. $^{13}$C NMR of 3i

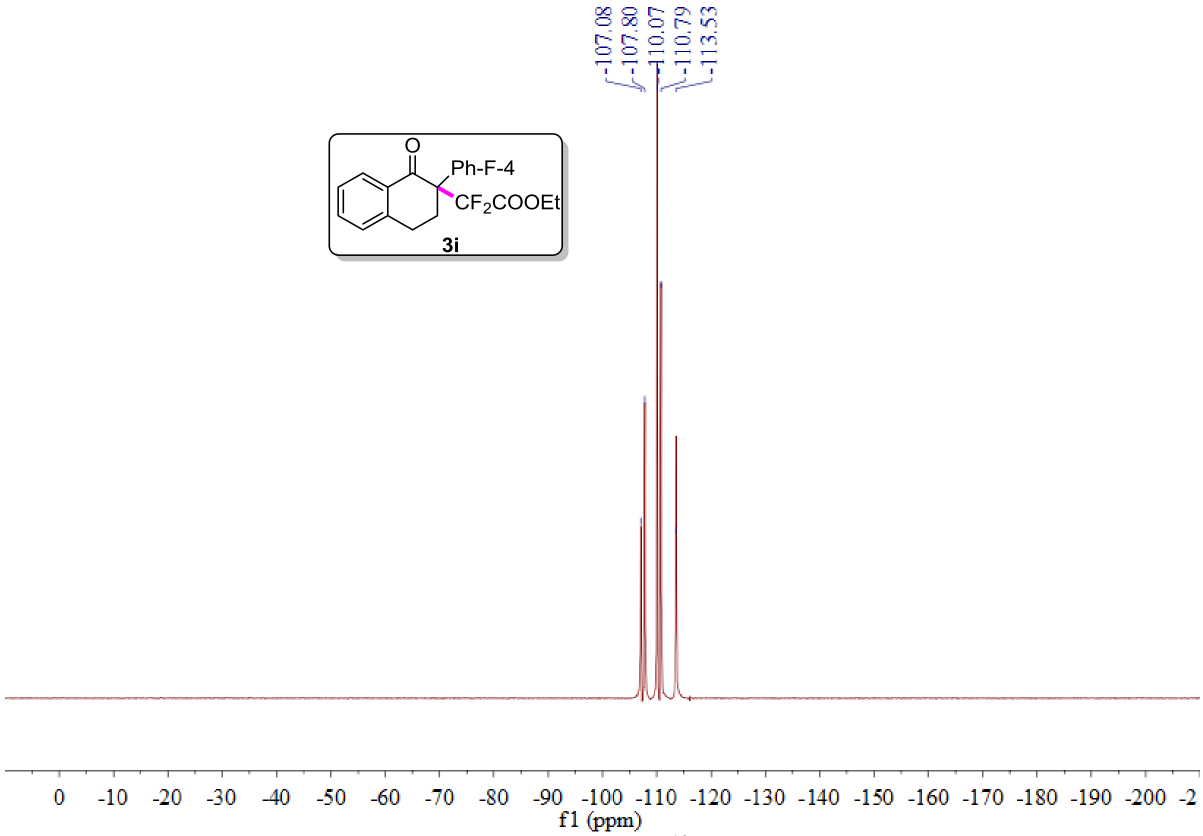

Supplementary Figure 27. $^{19}$F NMR of 3i

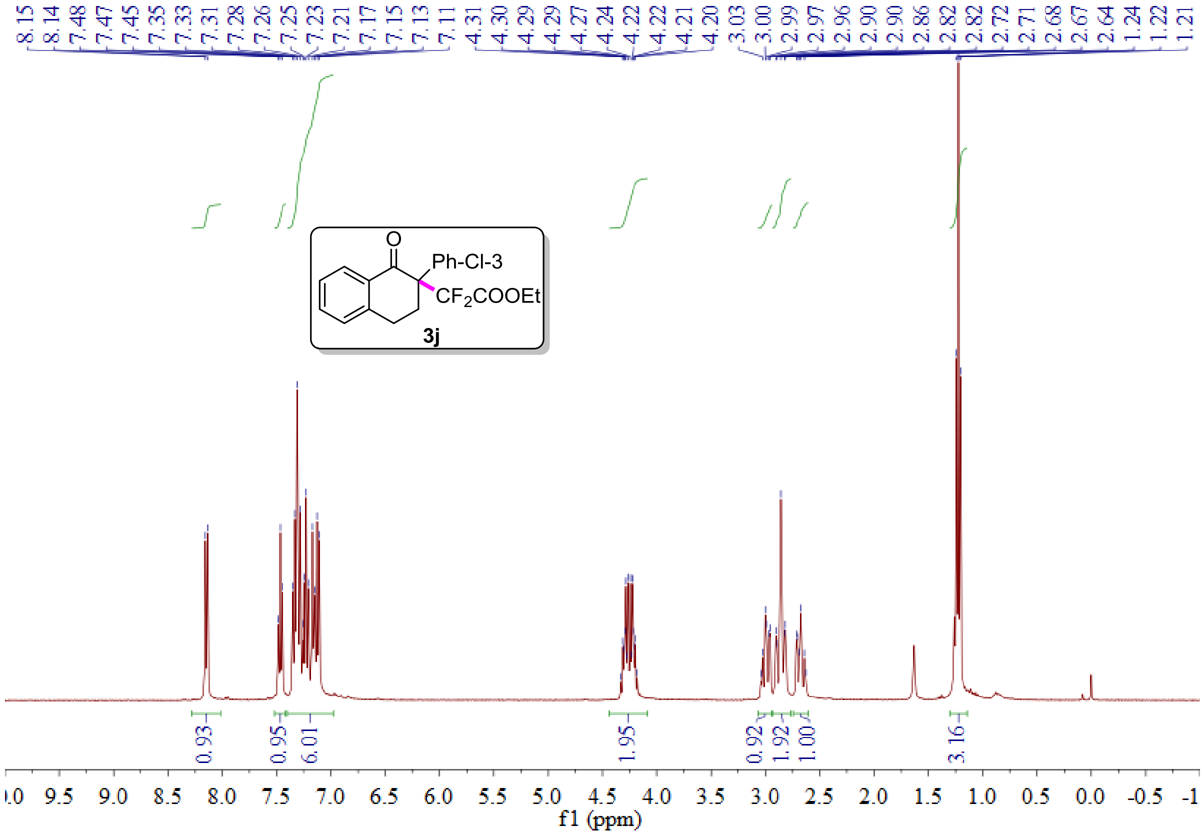

Supplementary Figure 28. $^{1}$H NMR of 3j

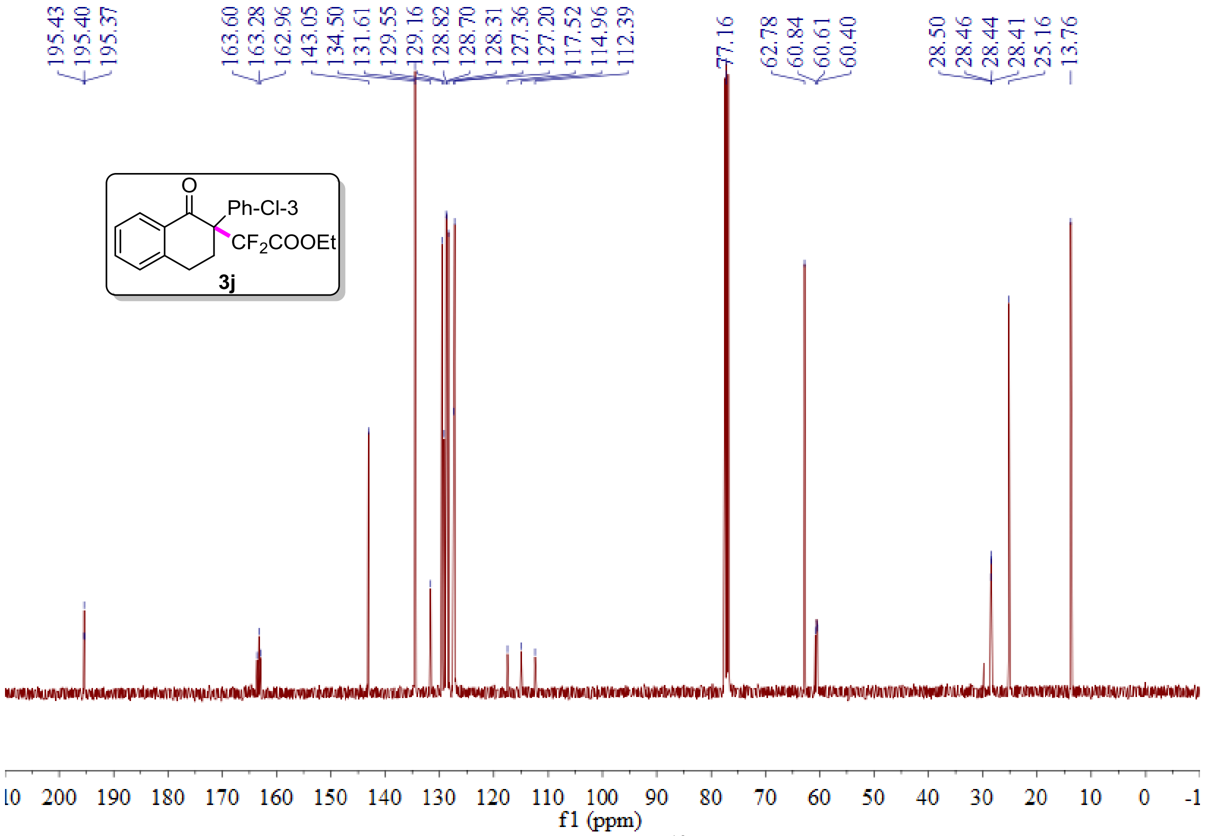


Supplementary Figure 29. $^{13}$C NMR of 3j

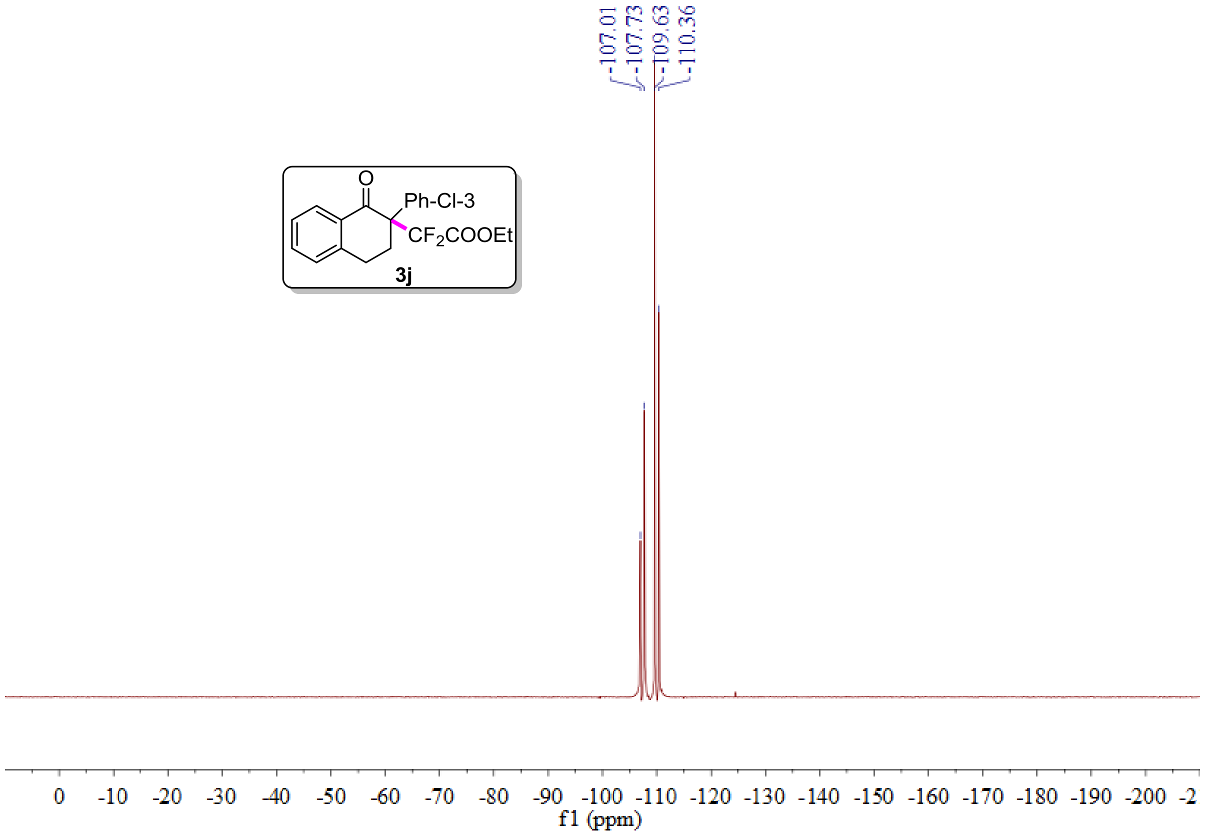


Supplementary Figure 30. $^{19}$F NMR of 3j

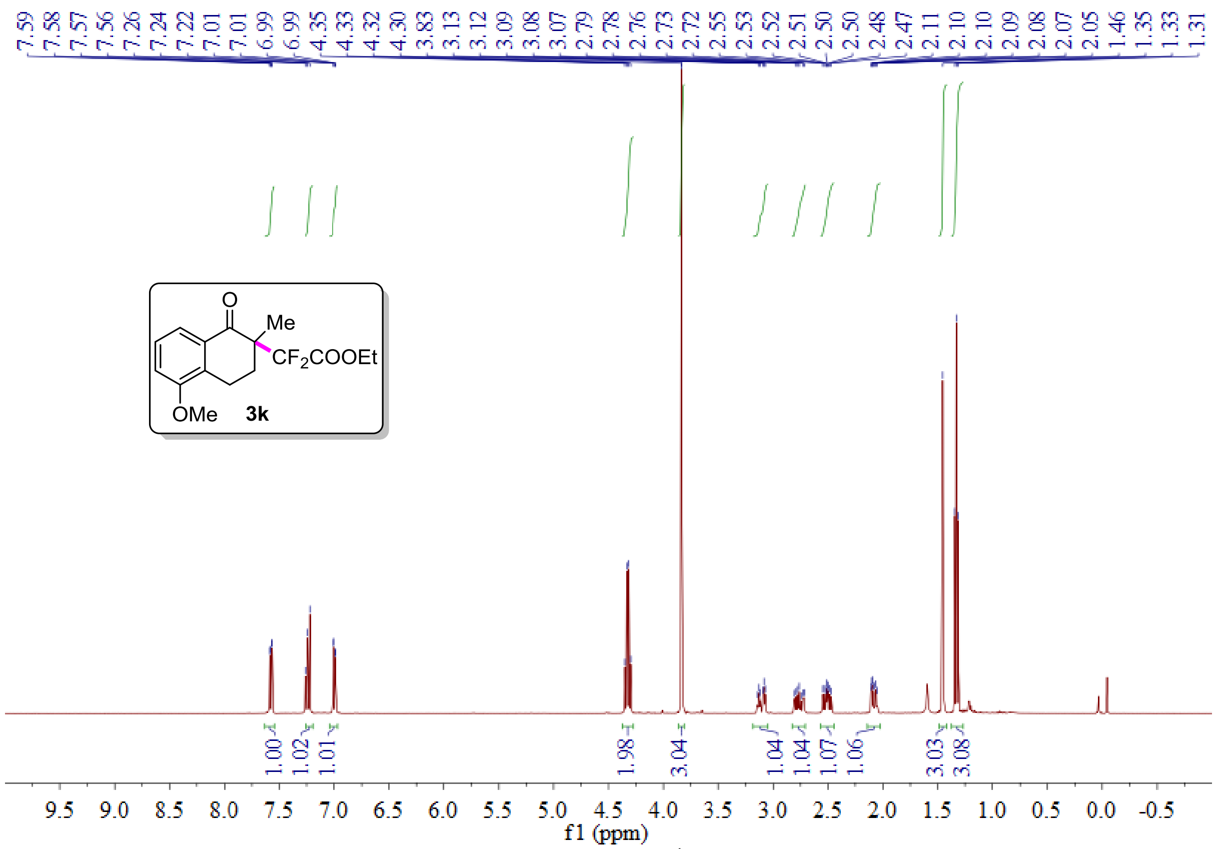

Supplementary Figure 31. $^{1}$H NMR of 3k

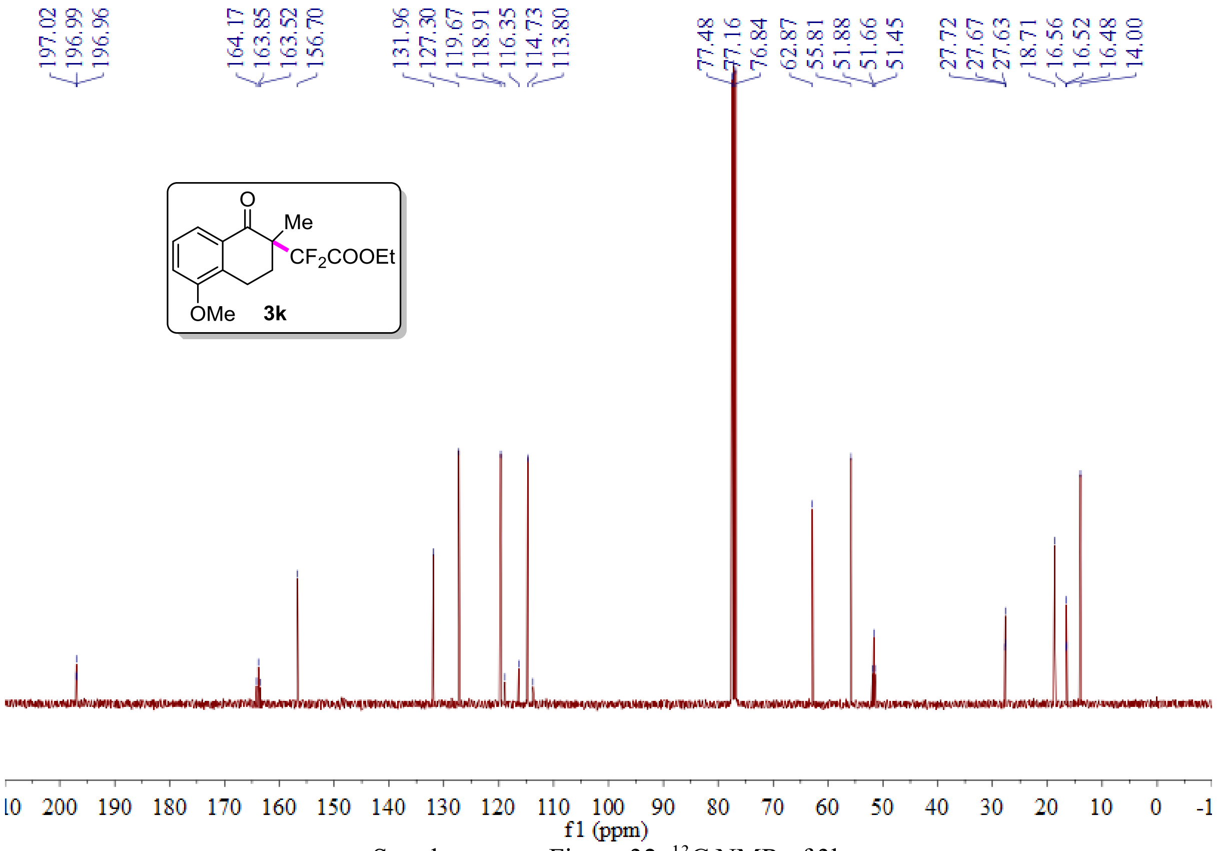

Supplementary Figure 32. $^{13}$C NMR of 3k

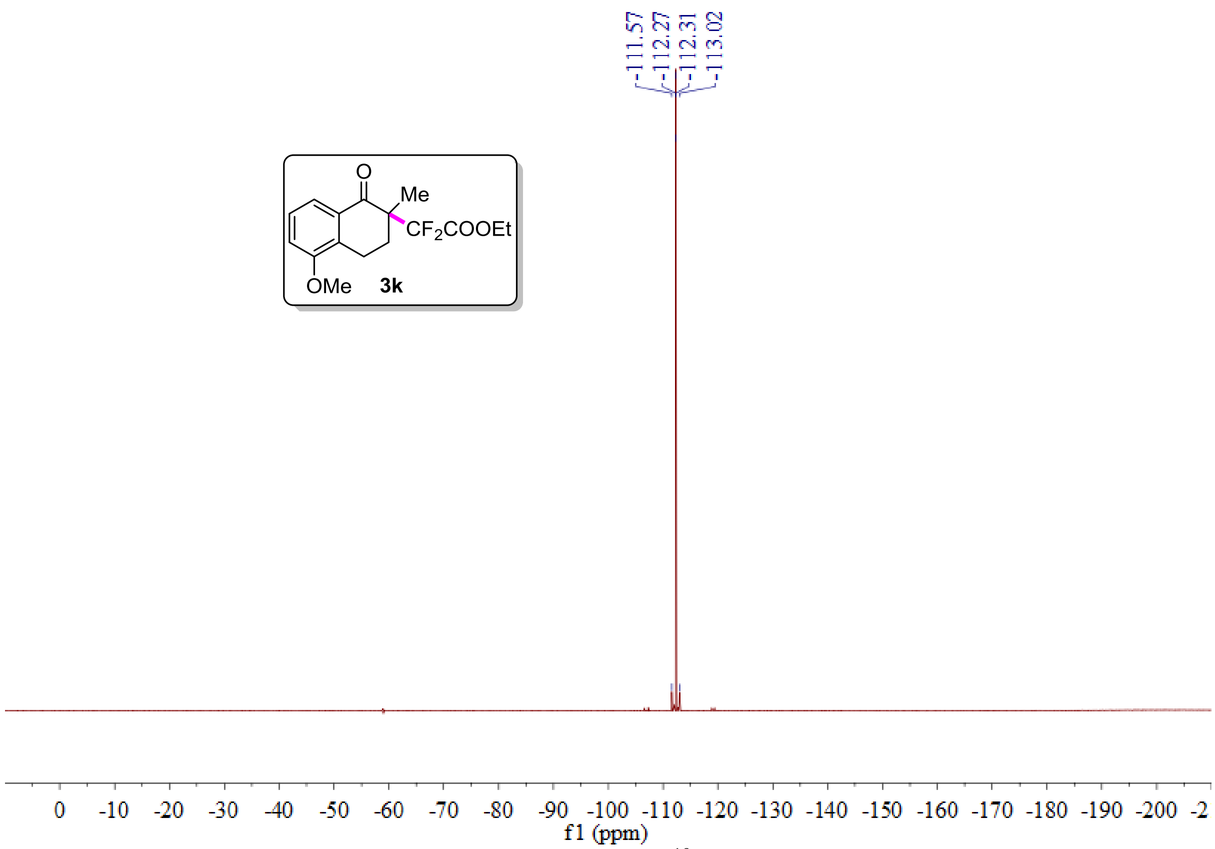

Supplementary Figure 33. $^{19}$F NMR of 3k

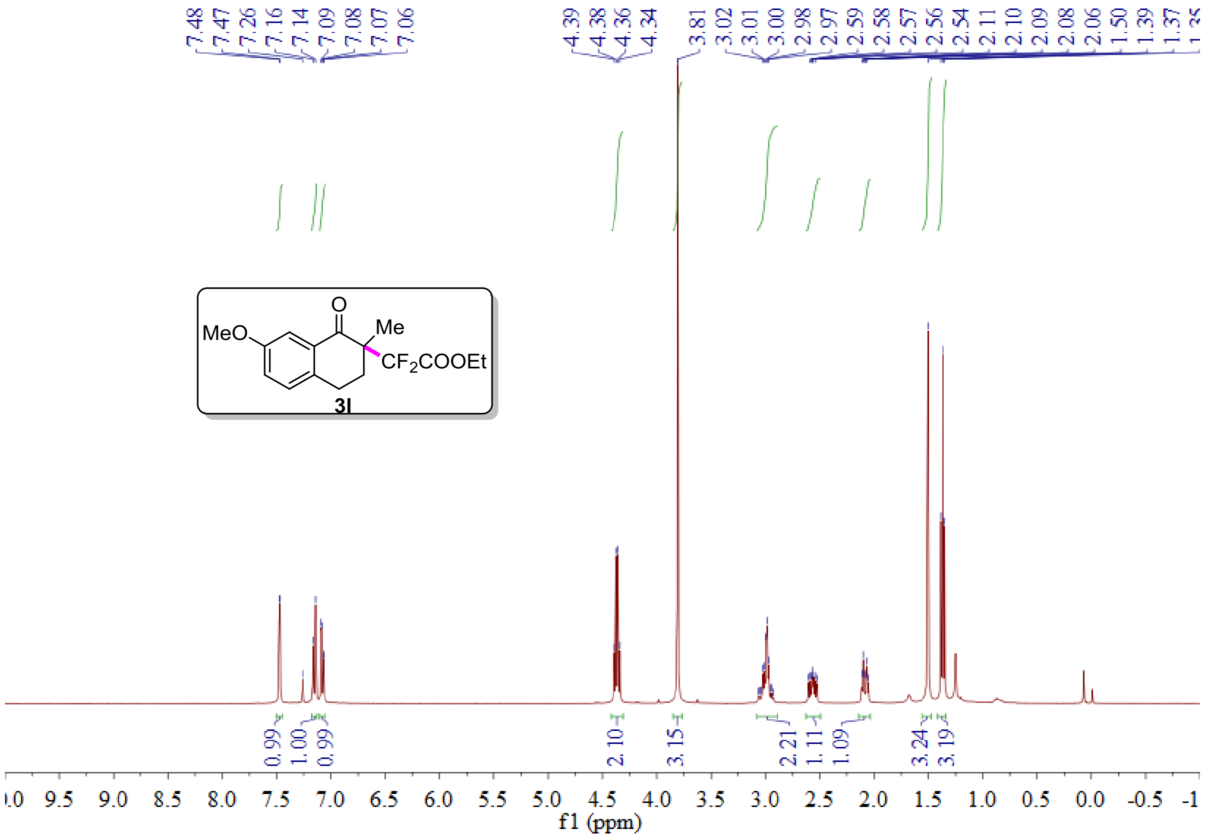

Supplementary Figure 34. $^{1}$H NMR of 3l

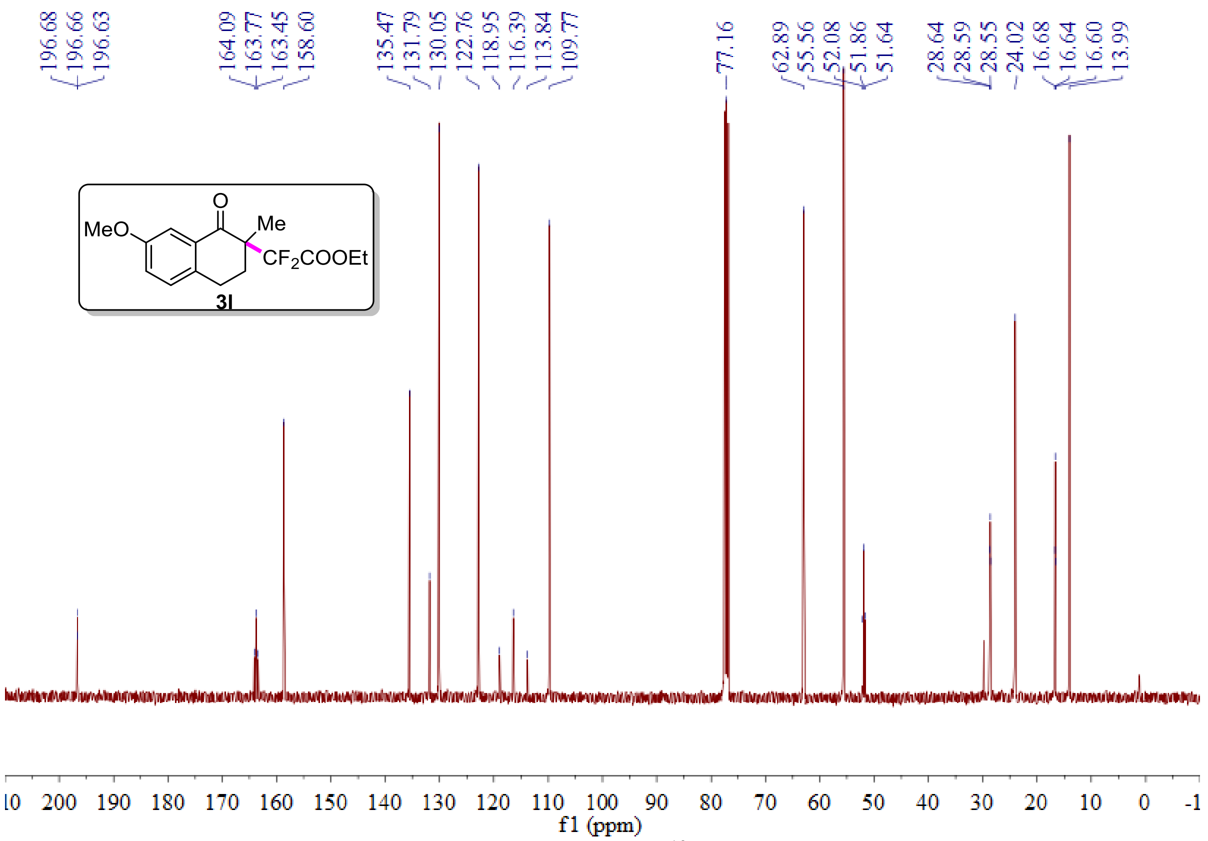

Supplementary Figure 35. $^{13}C$ NMR of 3l

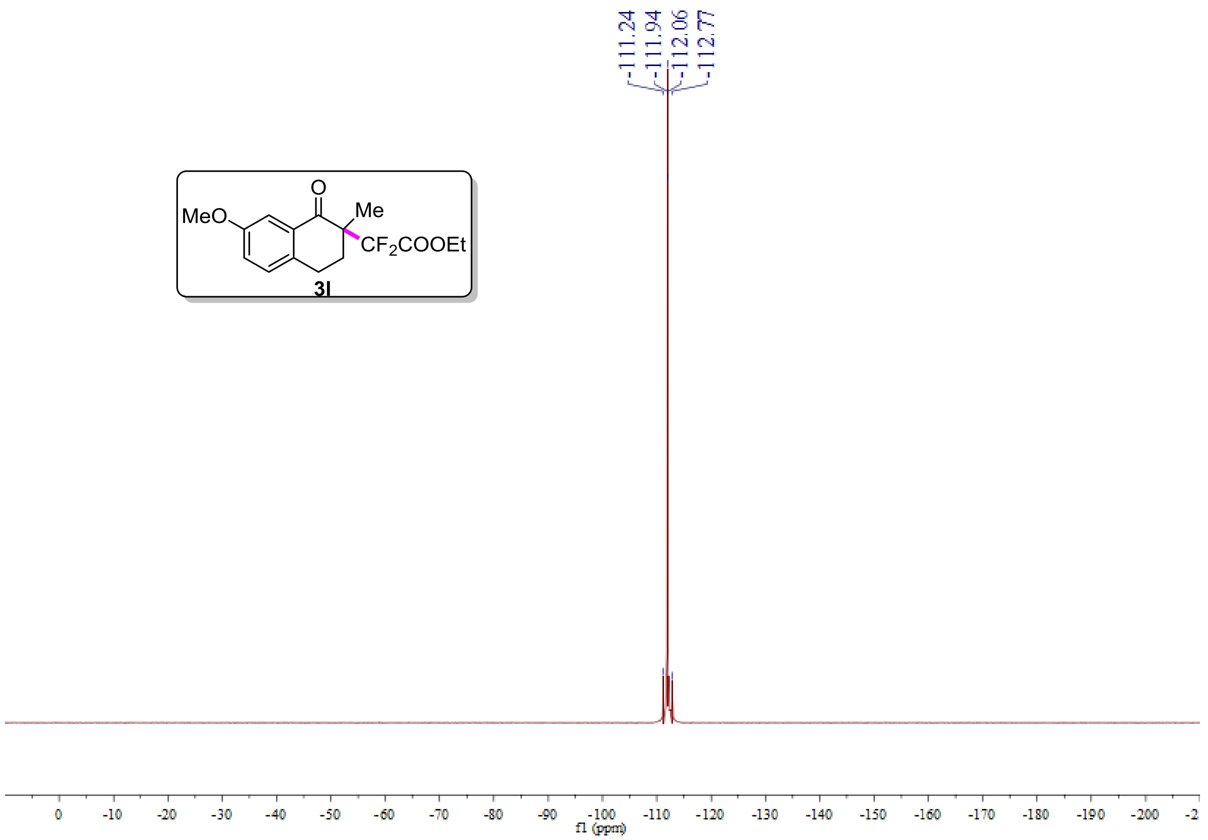

Supplementary Figure 36. $^{19}F$ NMR of 3l

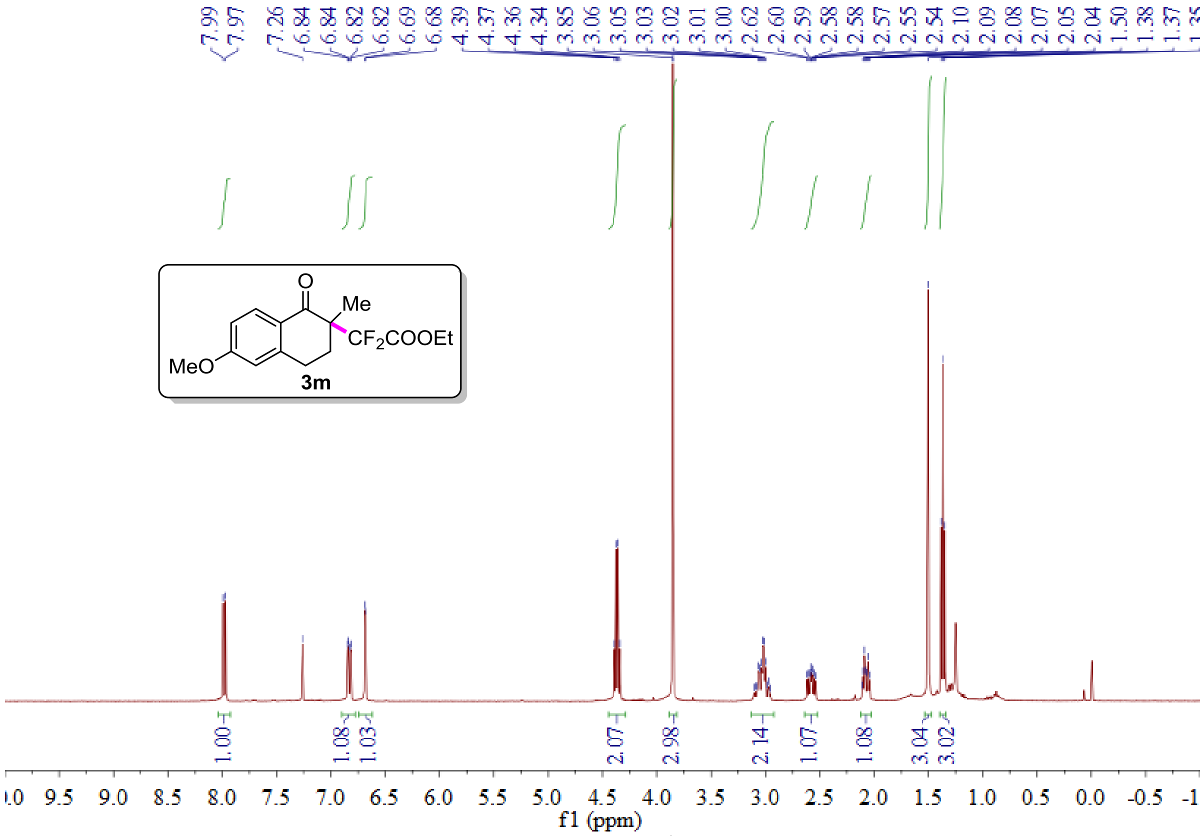


Supplementary Figure 37. $^{1}H$ NMR of 3m

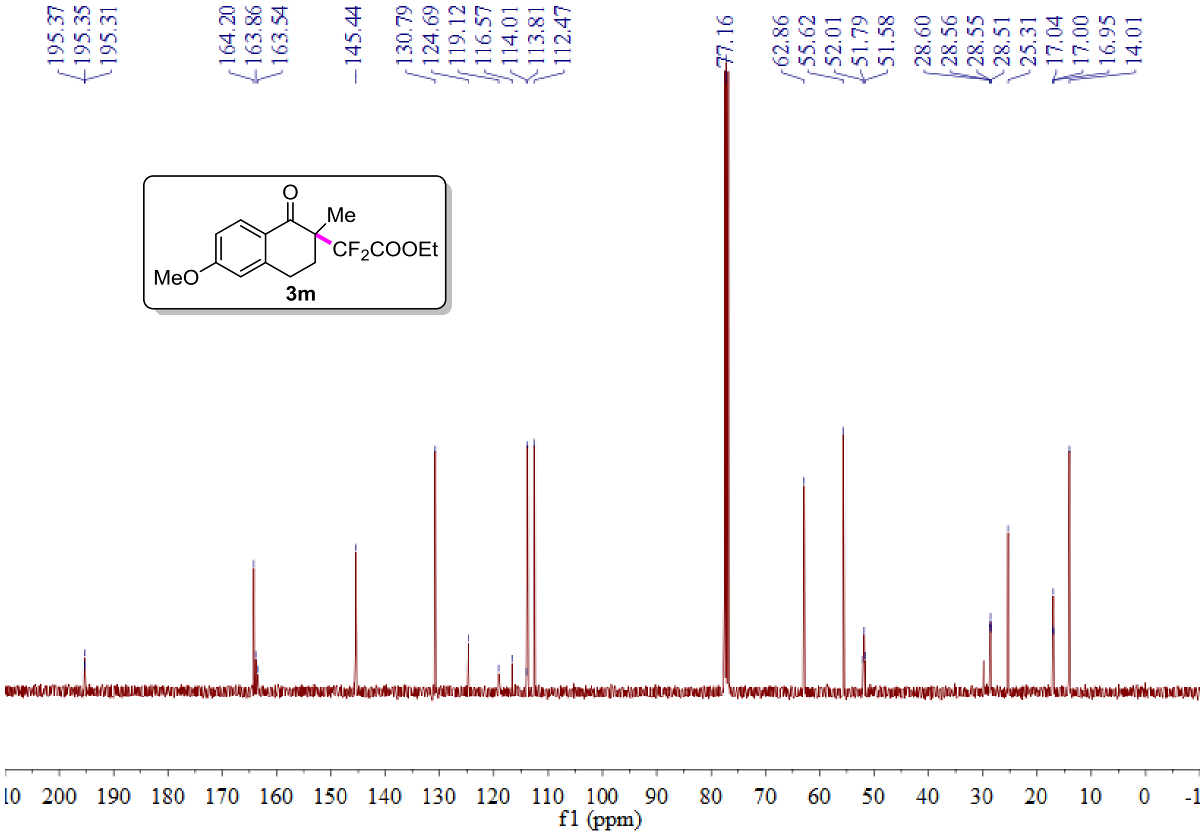


Supplementary Figure 38. $^{13}C$ NMR of 3m

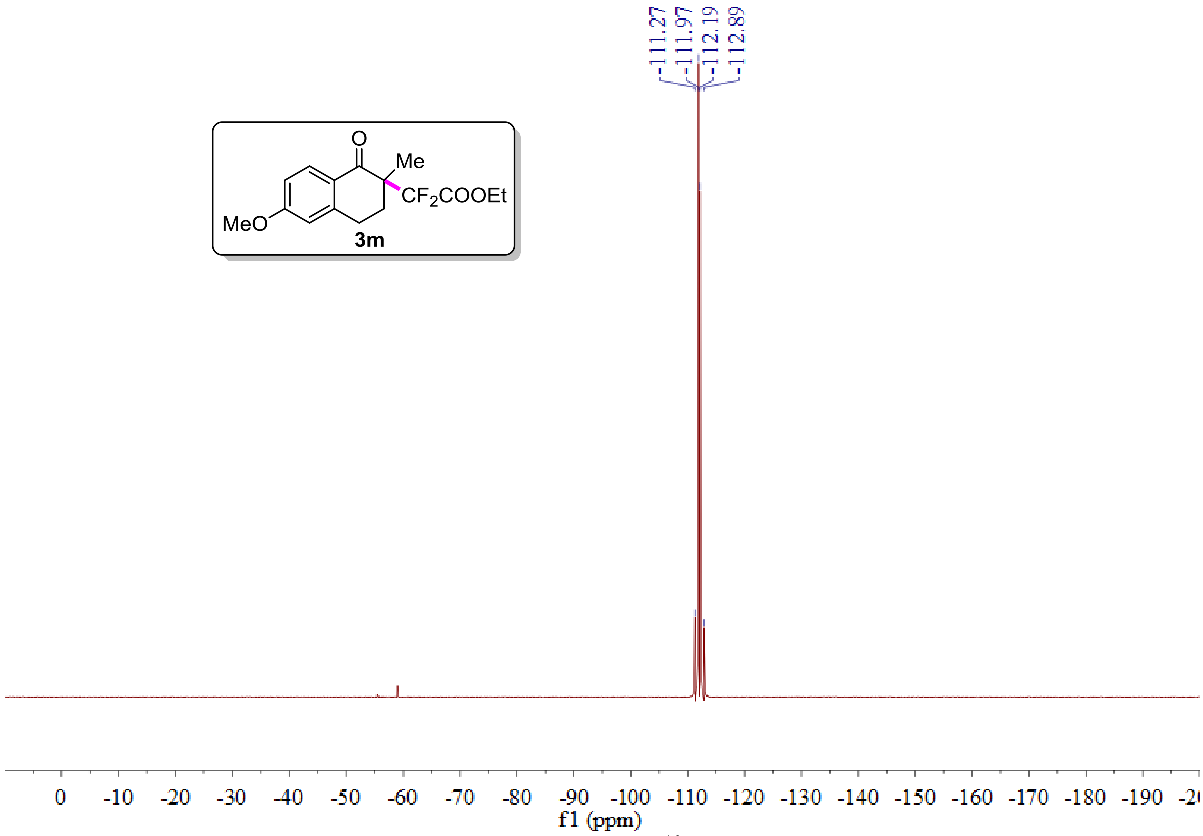


Supplementary Figure 39. $^{19}$F NMR of 3m

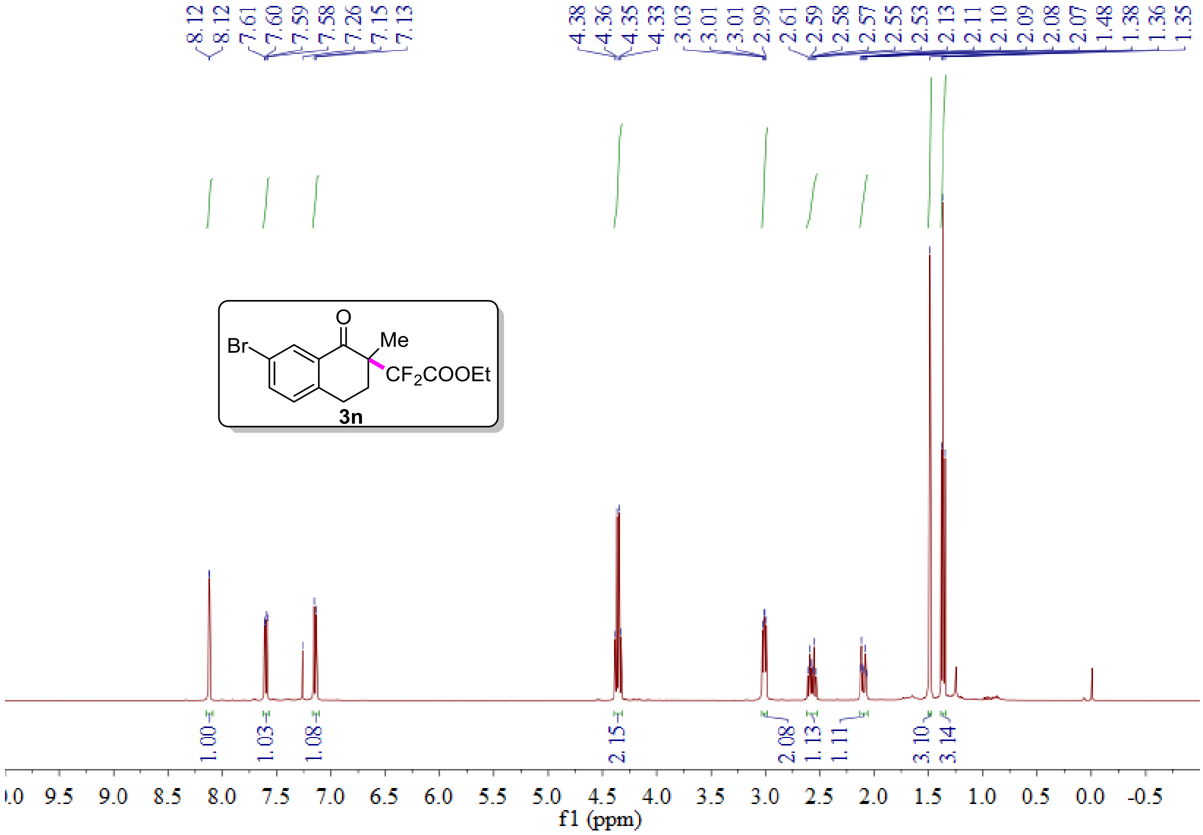


Supplementary Figure 40. $^{1}$H NMR of 3n

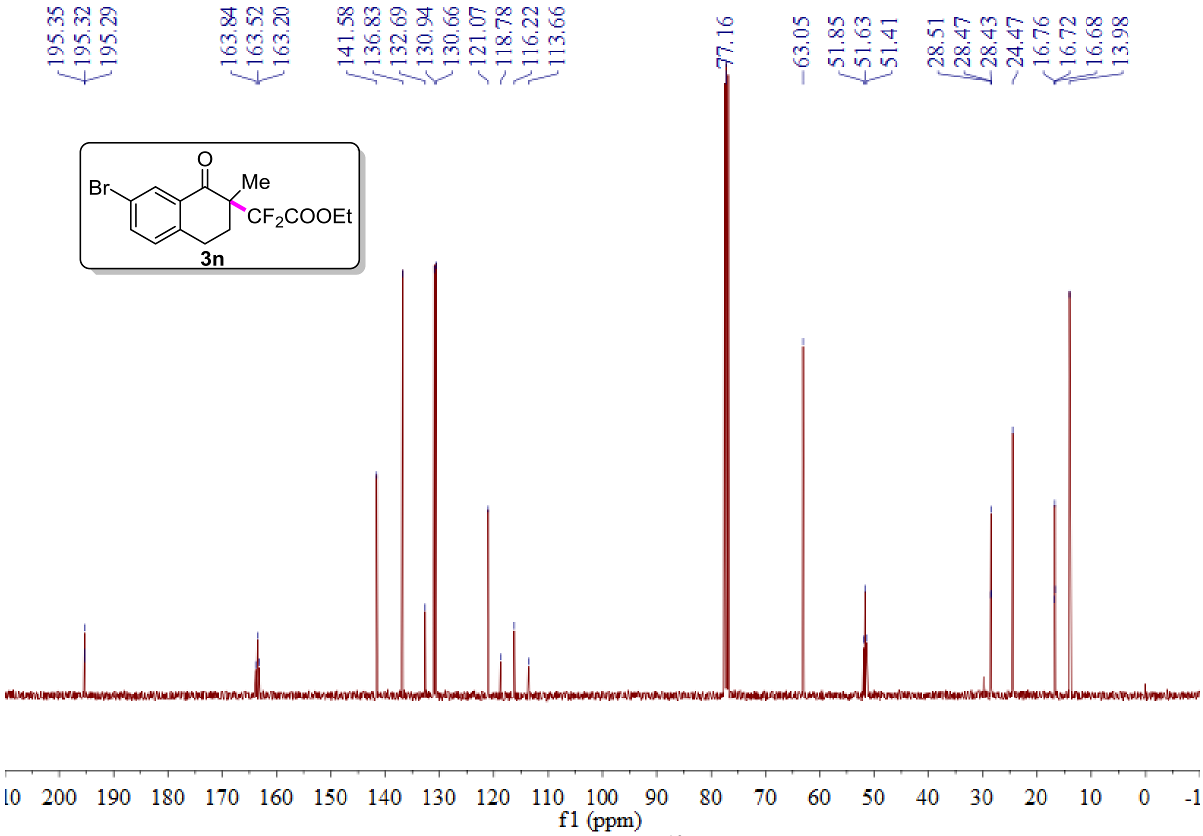


Supplementary Figure 41. $^{13}C$ NMR of 3n

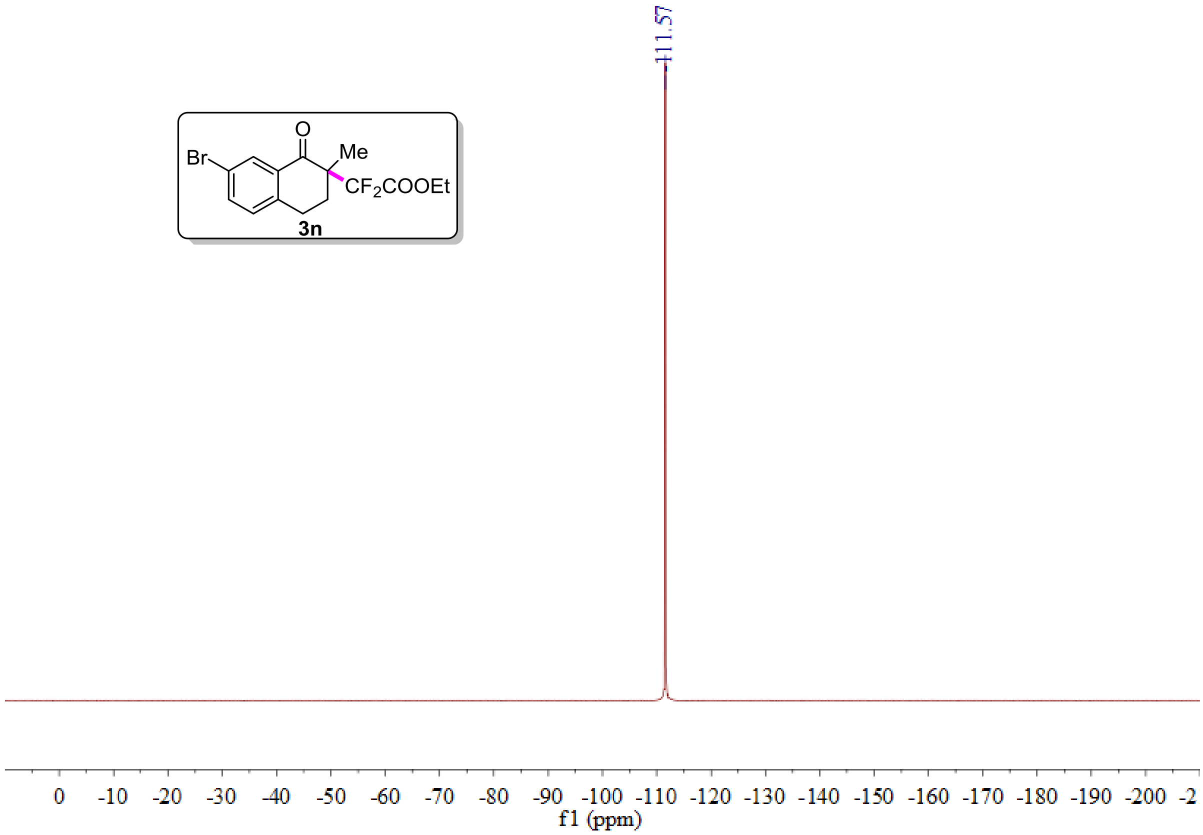


Supplementary Figure 42. $^{19}F$ NMR of 3n

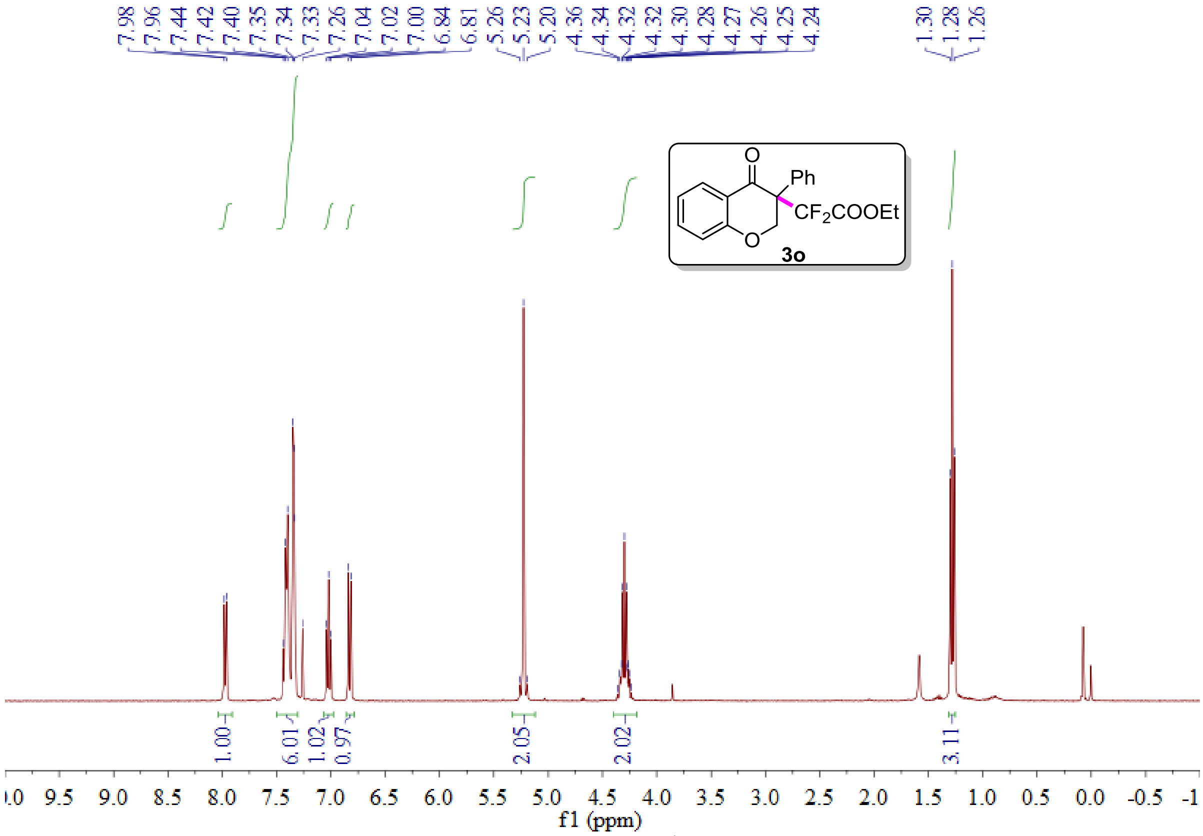


Supplementary Figure 43. $^{1}$H NMR of 3o

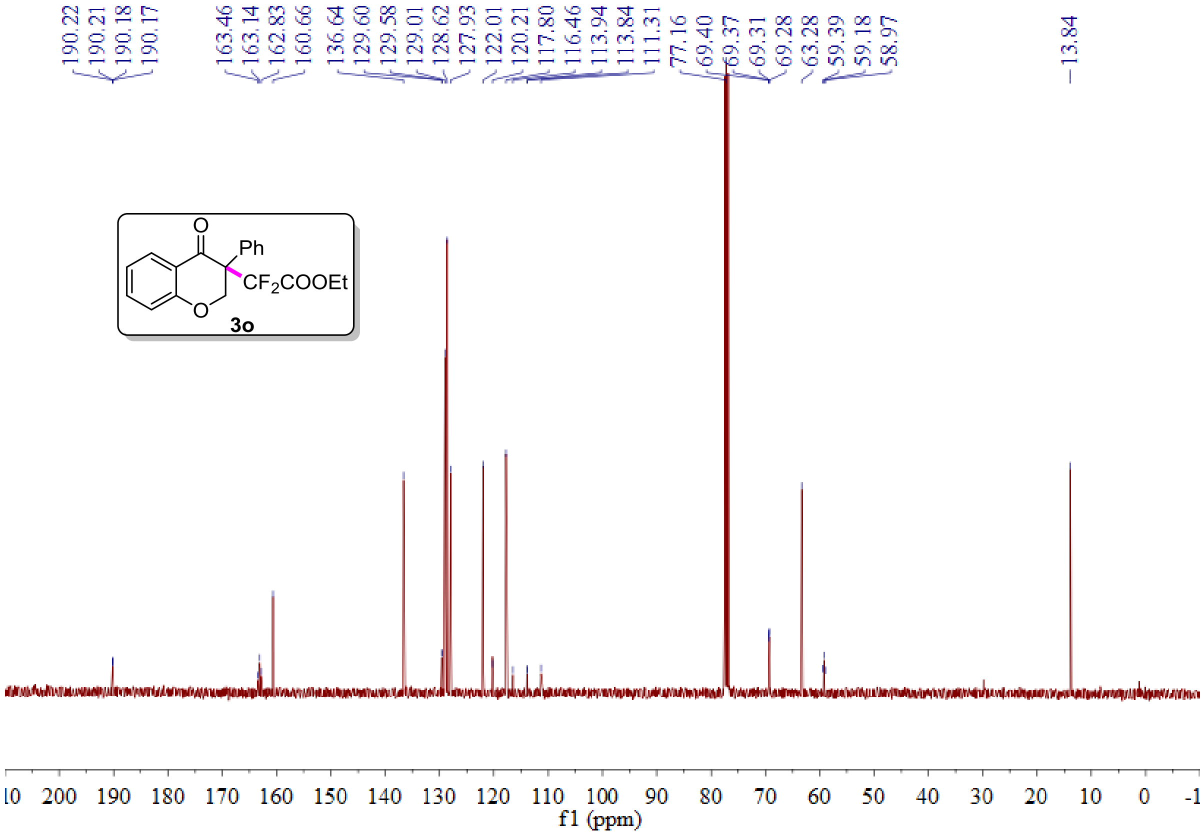


Supplementary Figure 44. $^{13}$C NMR of 3o

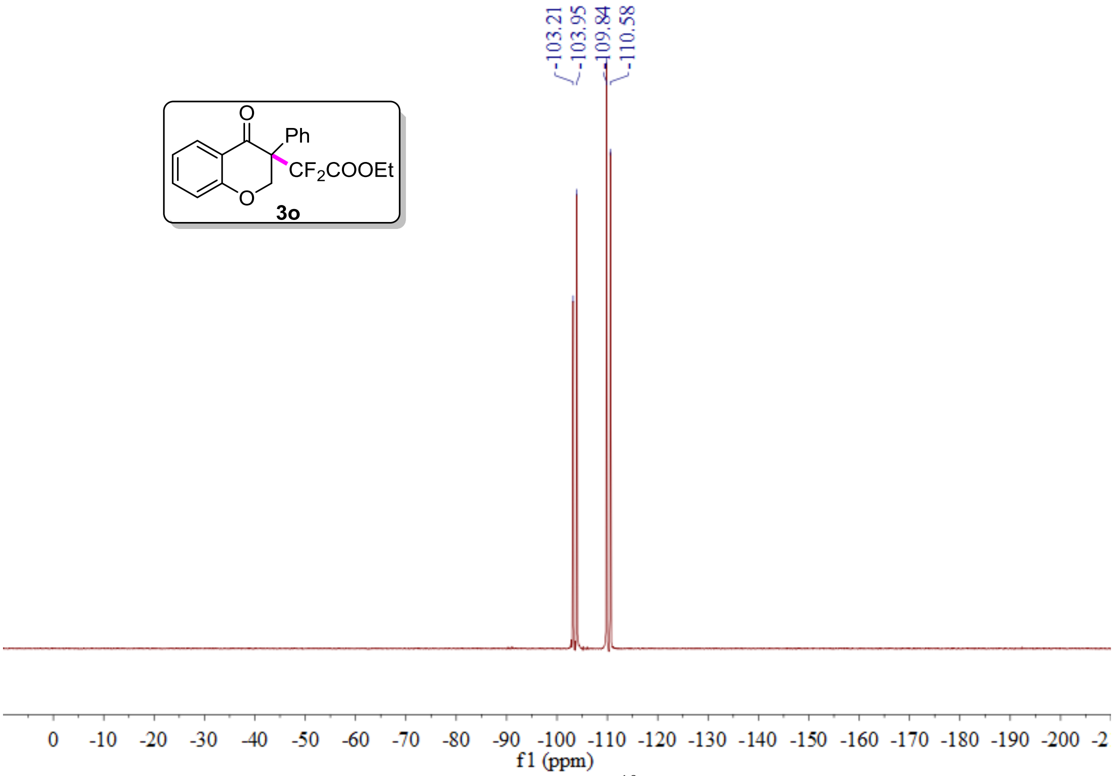


Supplementary Figure 45. $^{19}$F NMR of 3o

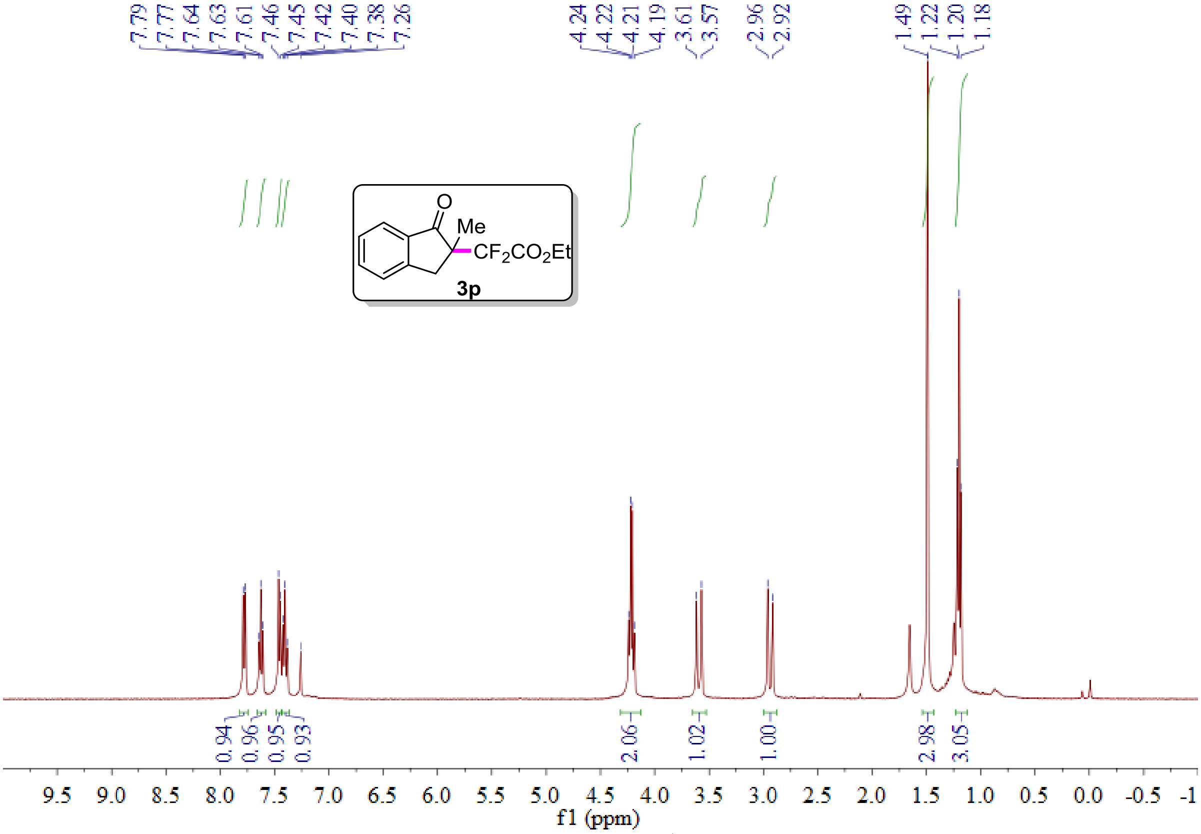


Supplementary Figure 46. $^{1}$H NMR of 3p

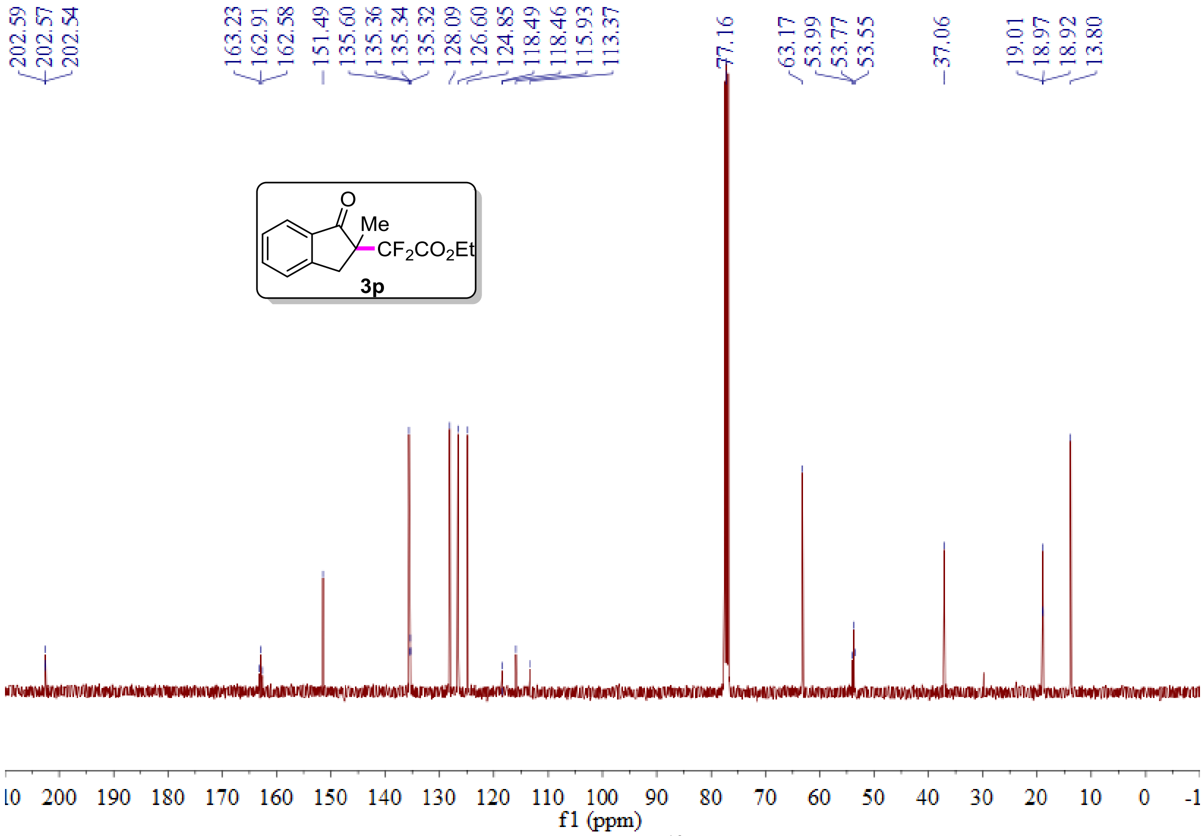


Supplementary Figure 47. $^{13}$C NMR of 3p

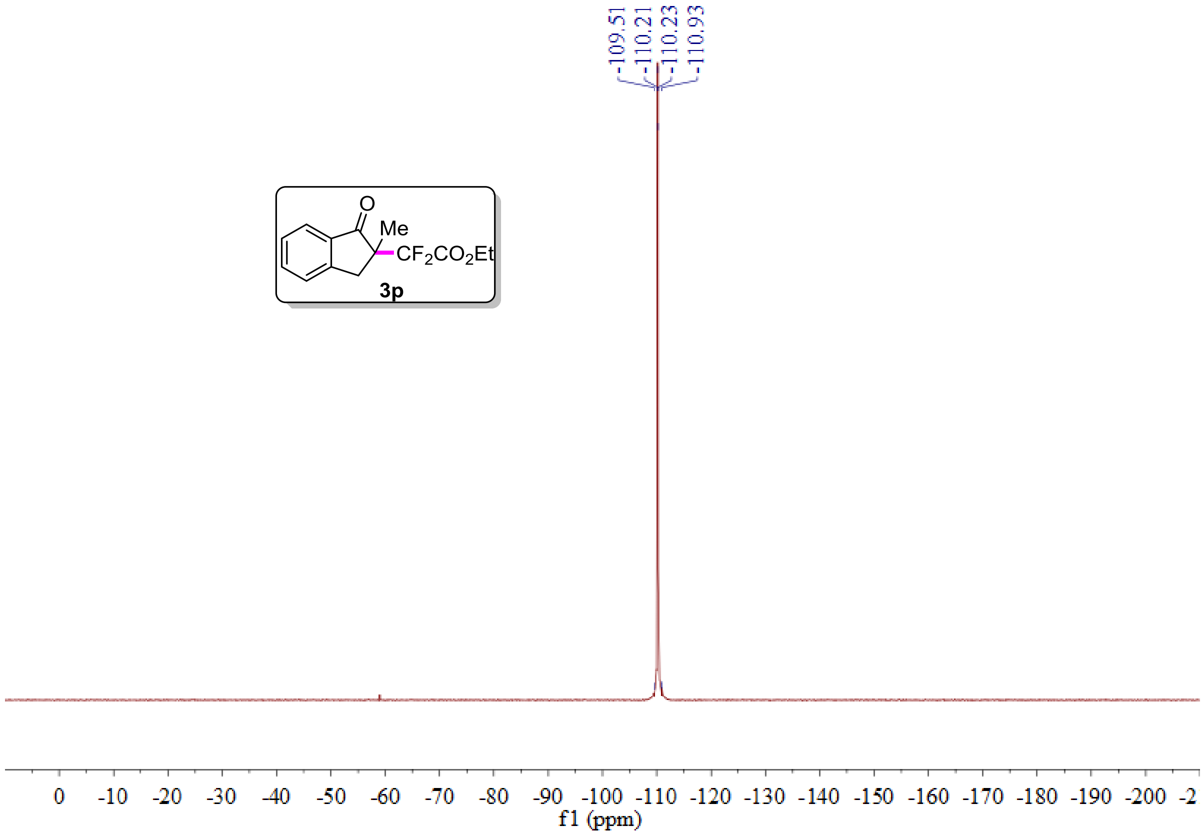


Supplementary Figure 48. $^{19}$F NMR of 3p

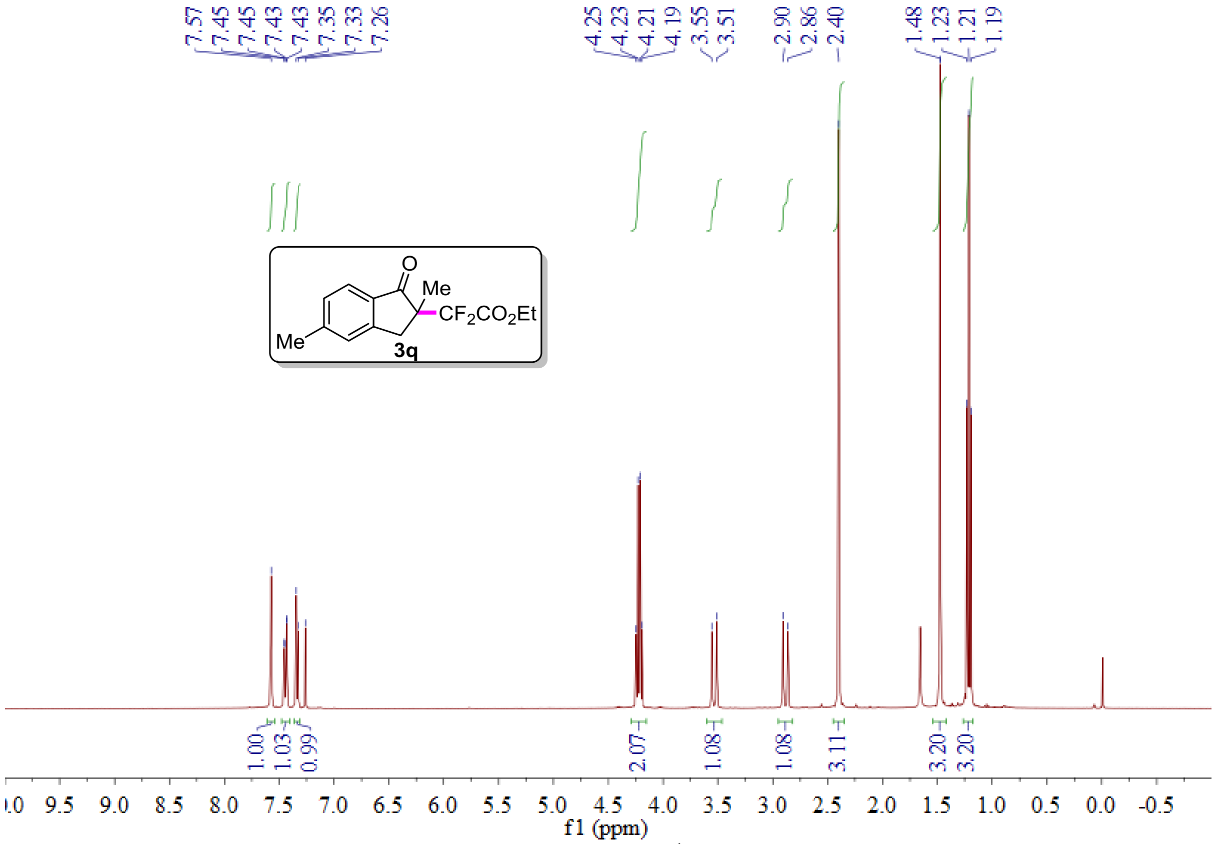


Supplementary Figure 49. $^{1}$H NMR of 3q

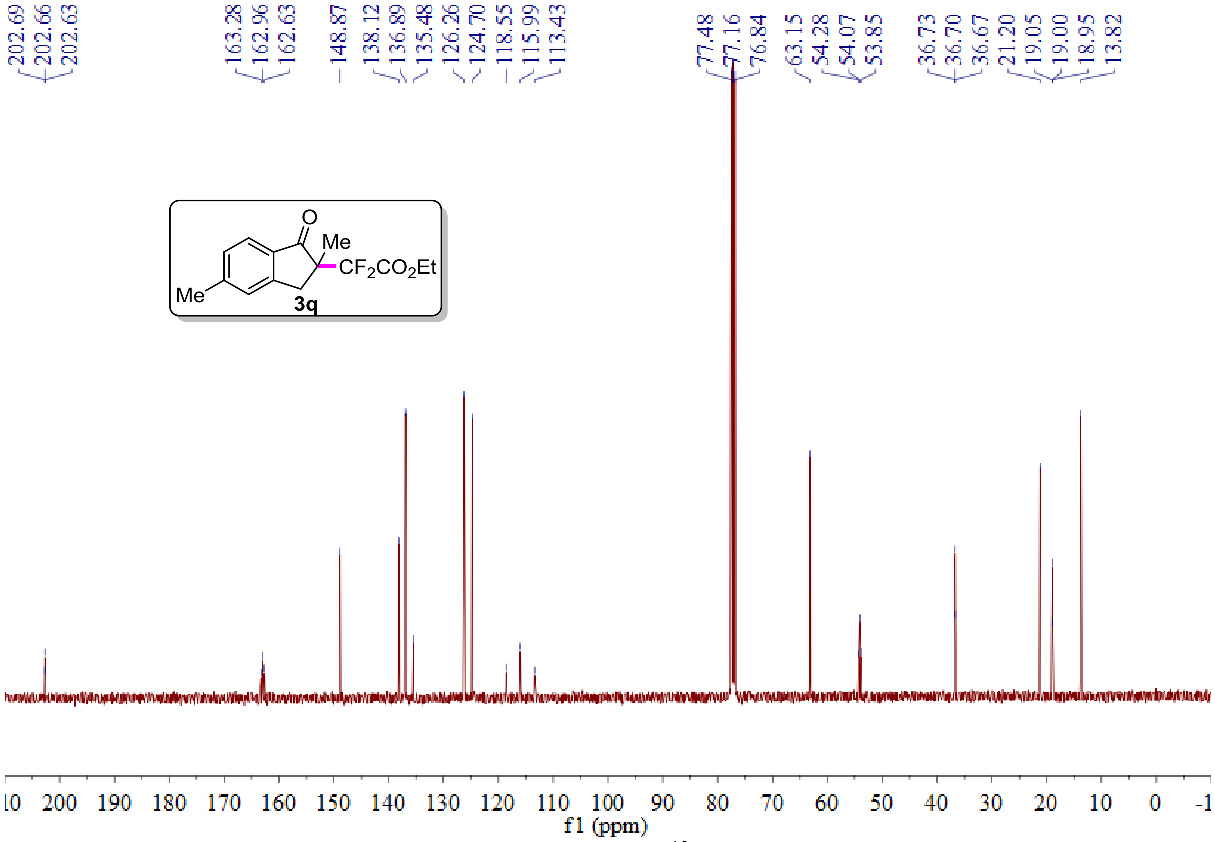


Supplementary Figure 50. $^{13}$C NMR of 3q

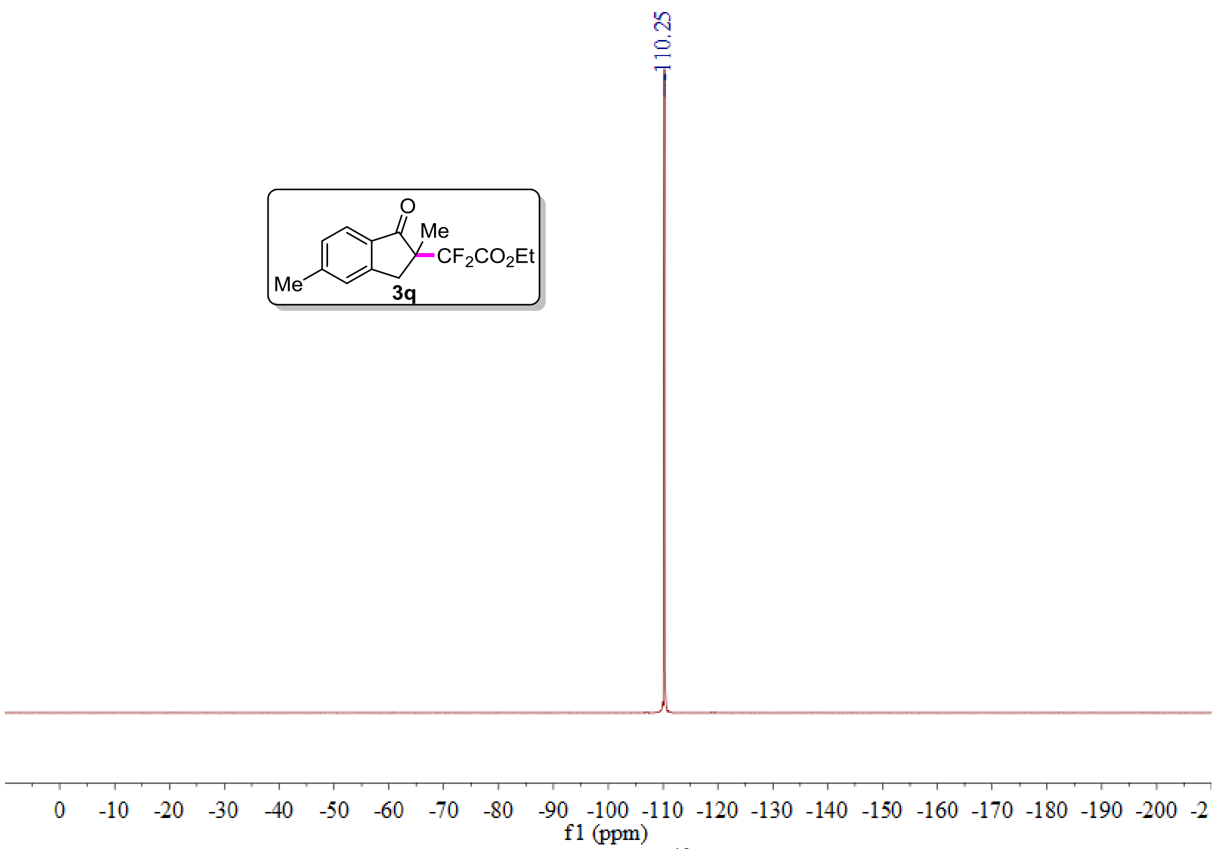


Supplementary Figure 51. $^{19}$F NMR of 3q

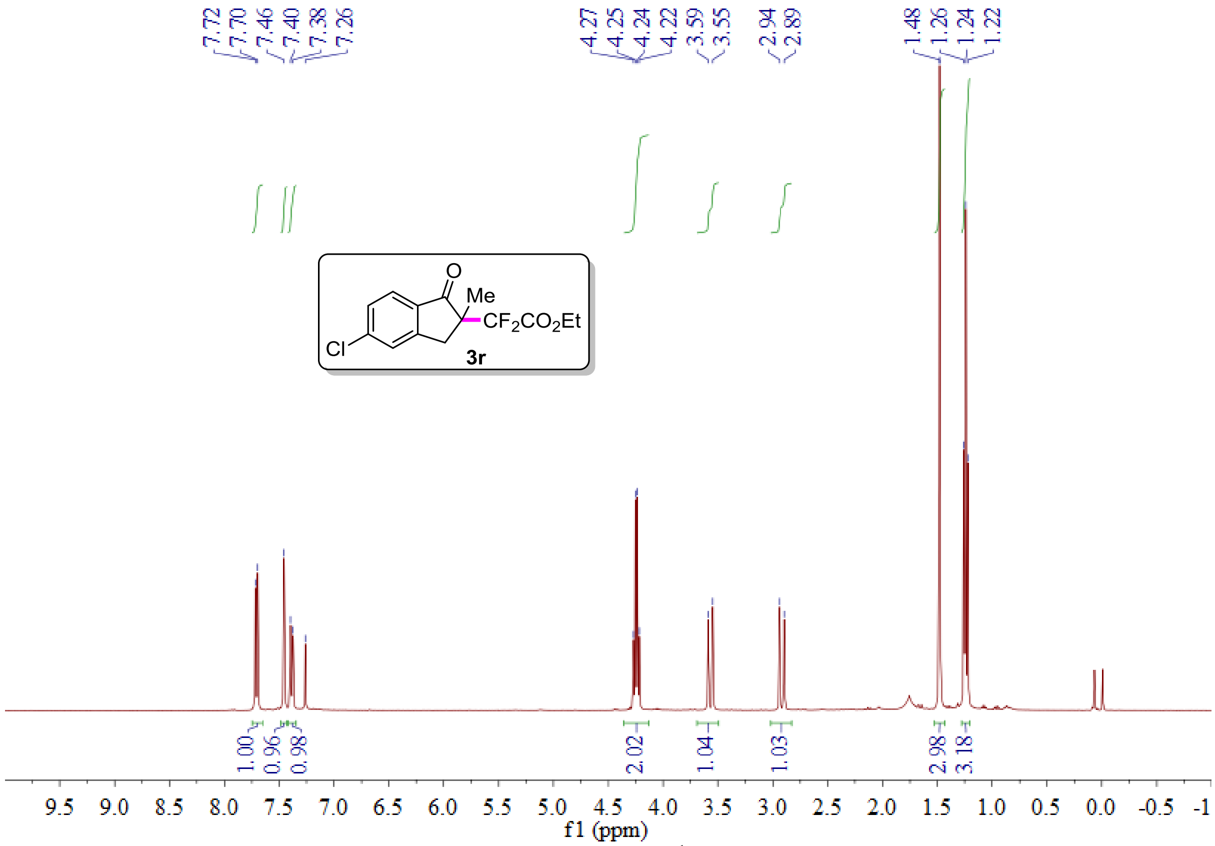


Supplementary Figure 52. $^{1}$H NMR of 3r

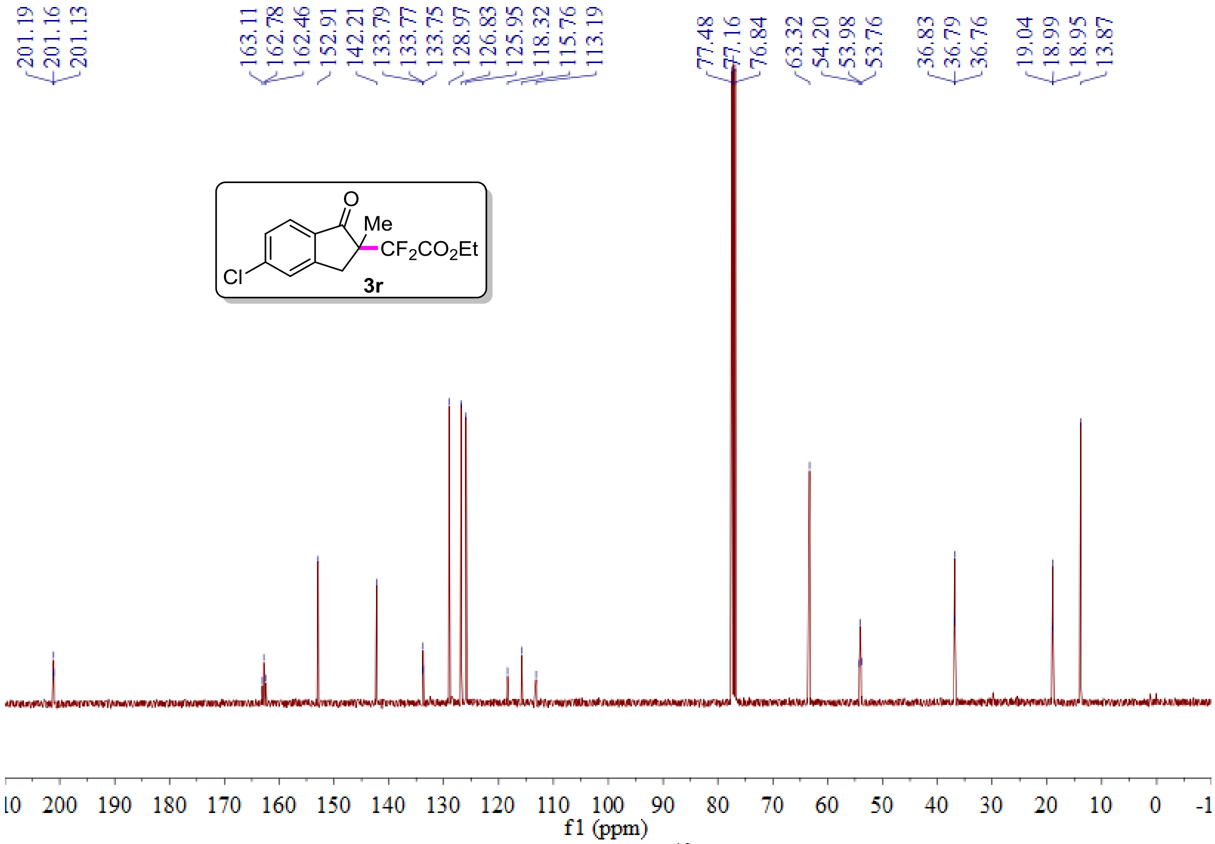


Supplementary Figure 53. $^{13}C$ NMR of 3r

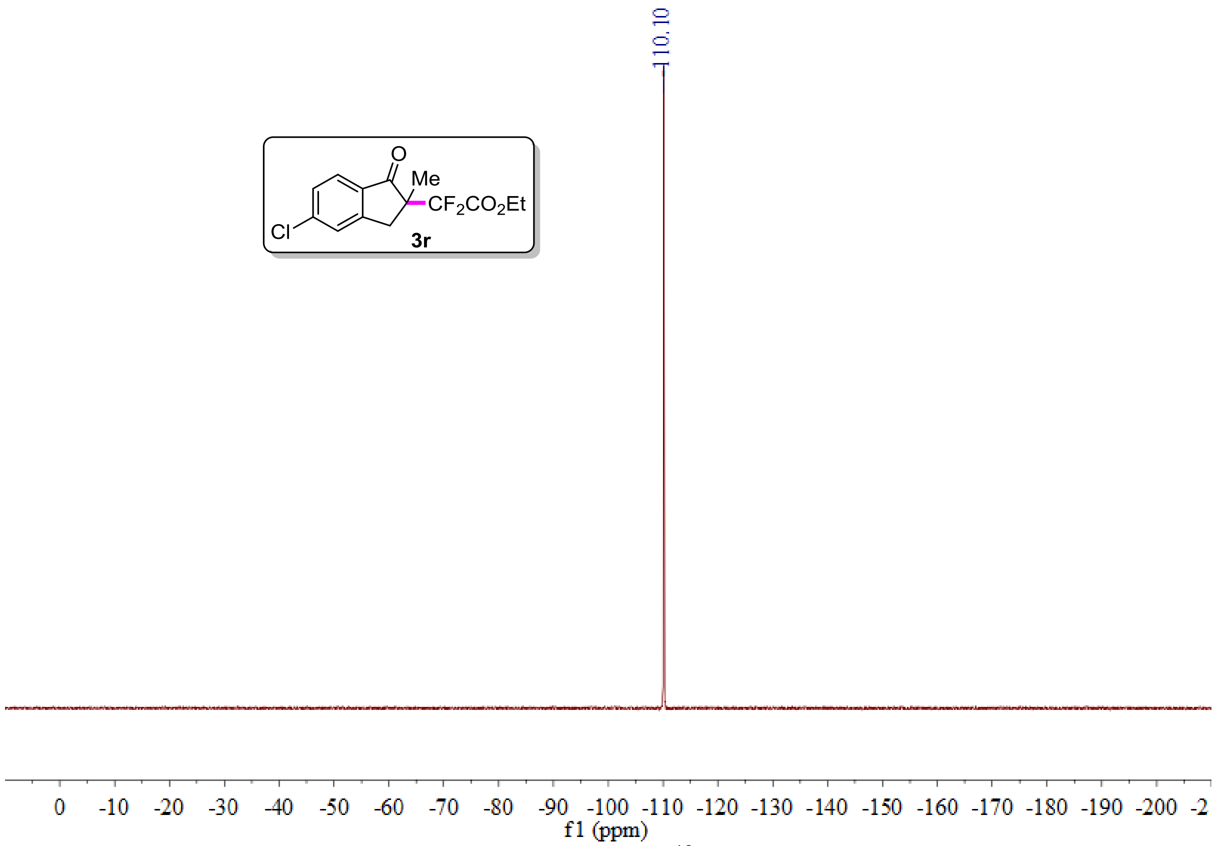


Supplementary Figure 54. $^{19}F$ NMR of 3r

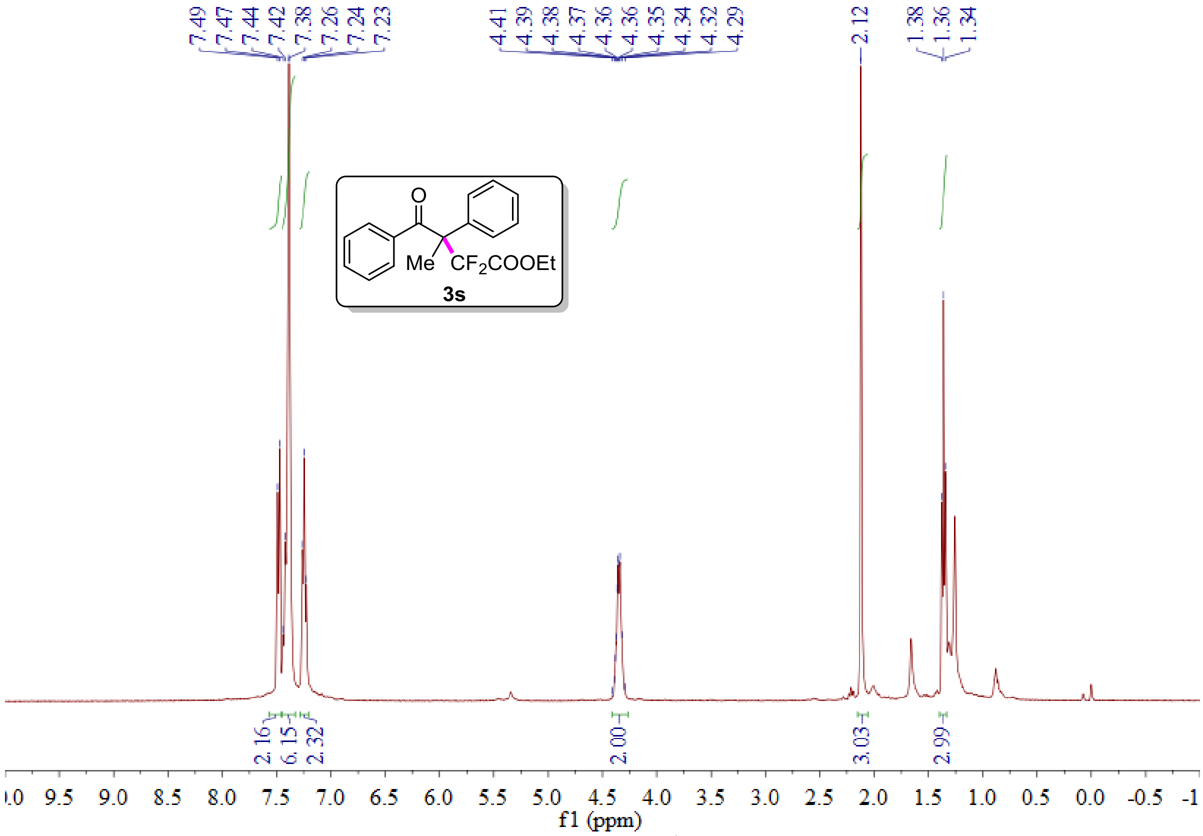


Supplementary Figure 55. $^{1}$H NMR of 3s

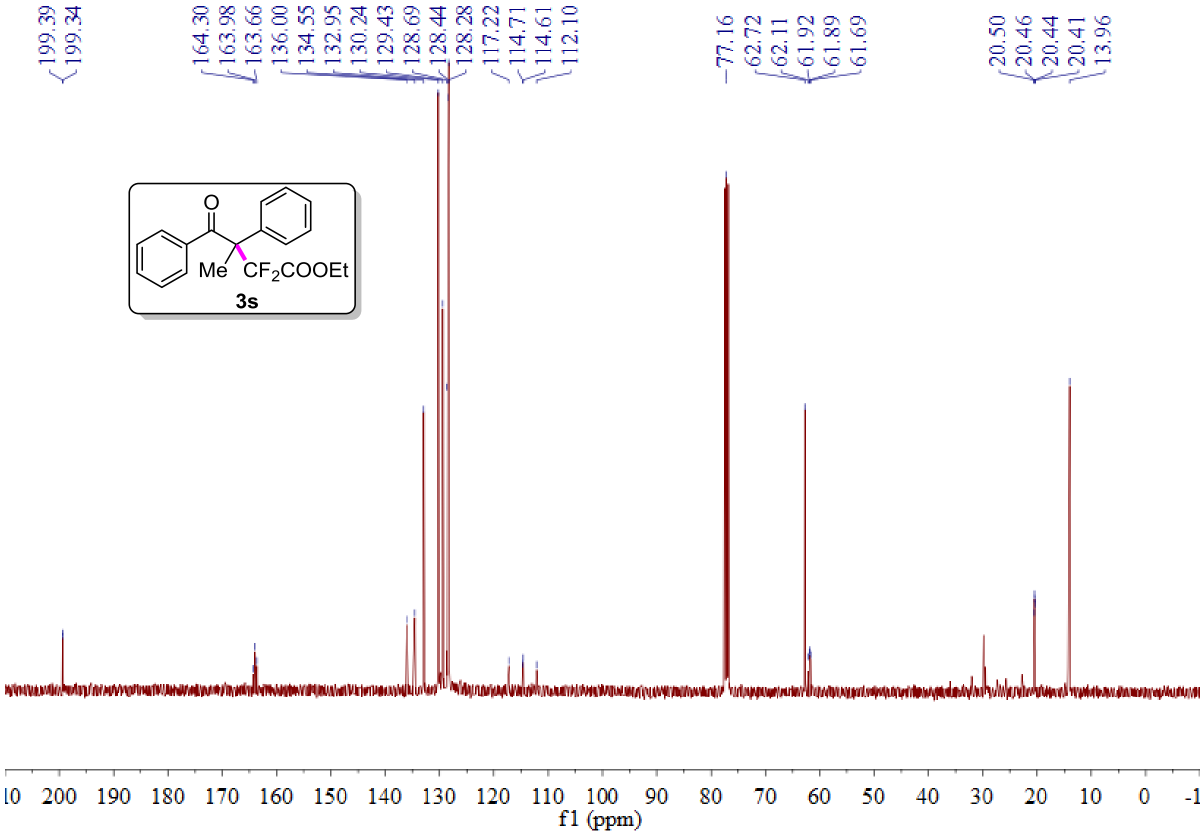


Supplementary Figure 56. $^{13}$C NMR of 3s

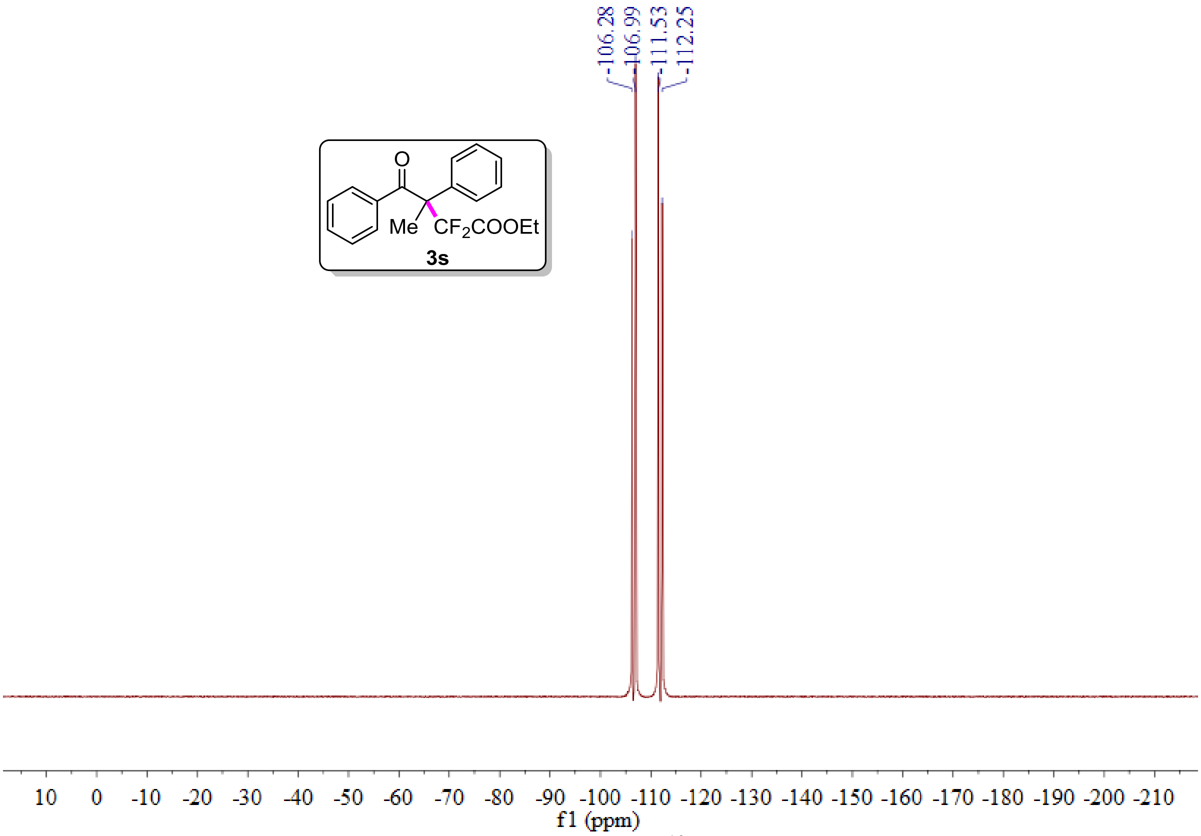


Supplementary Figure 57. $^{19}$F NMR of 3s

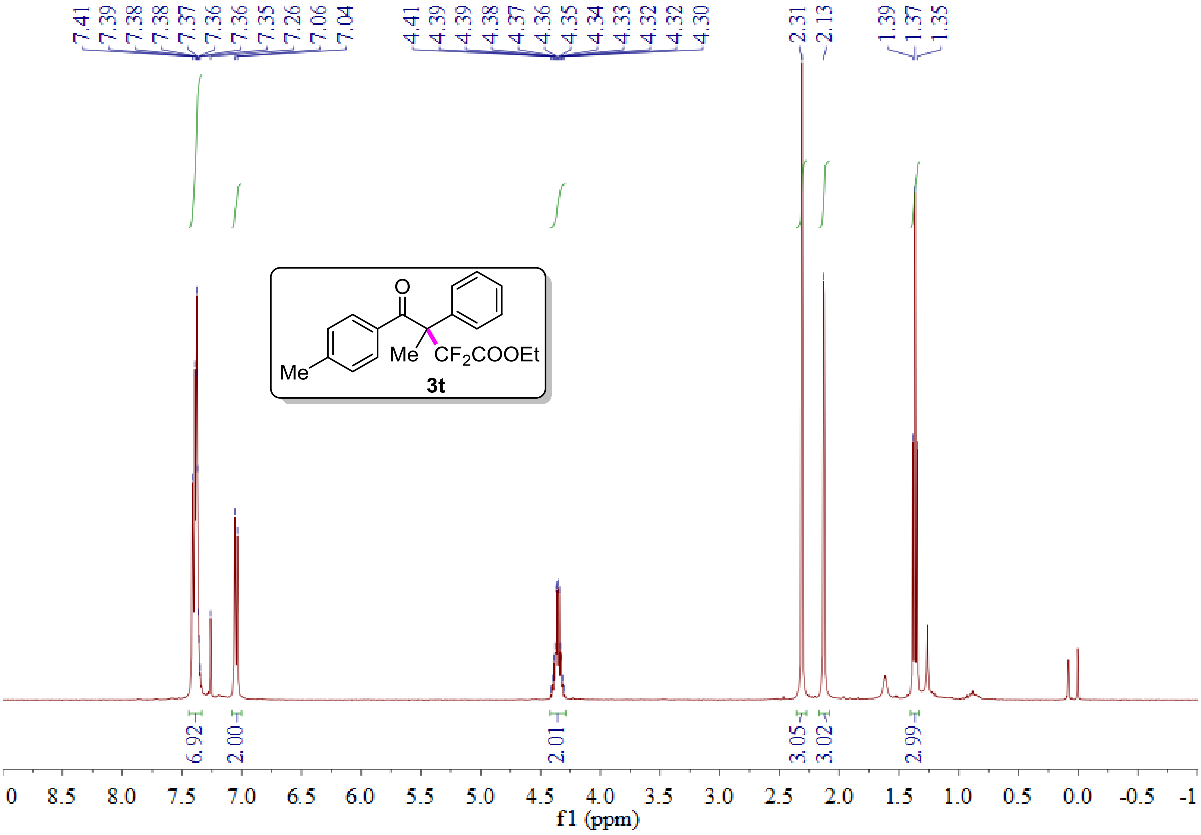


Supplementary Figure 58. $^{1}$H NMR of 3t

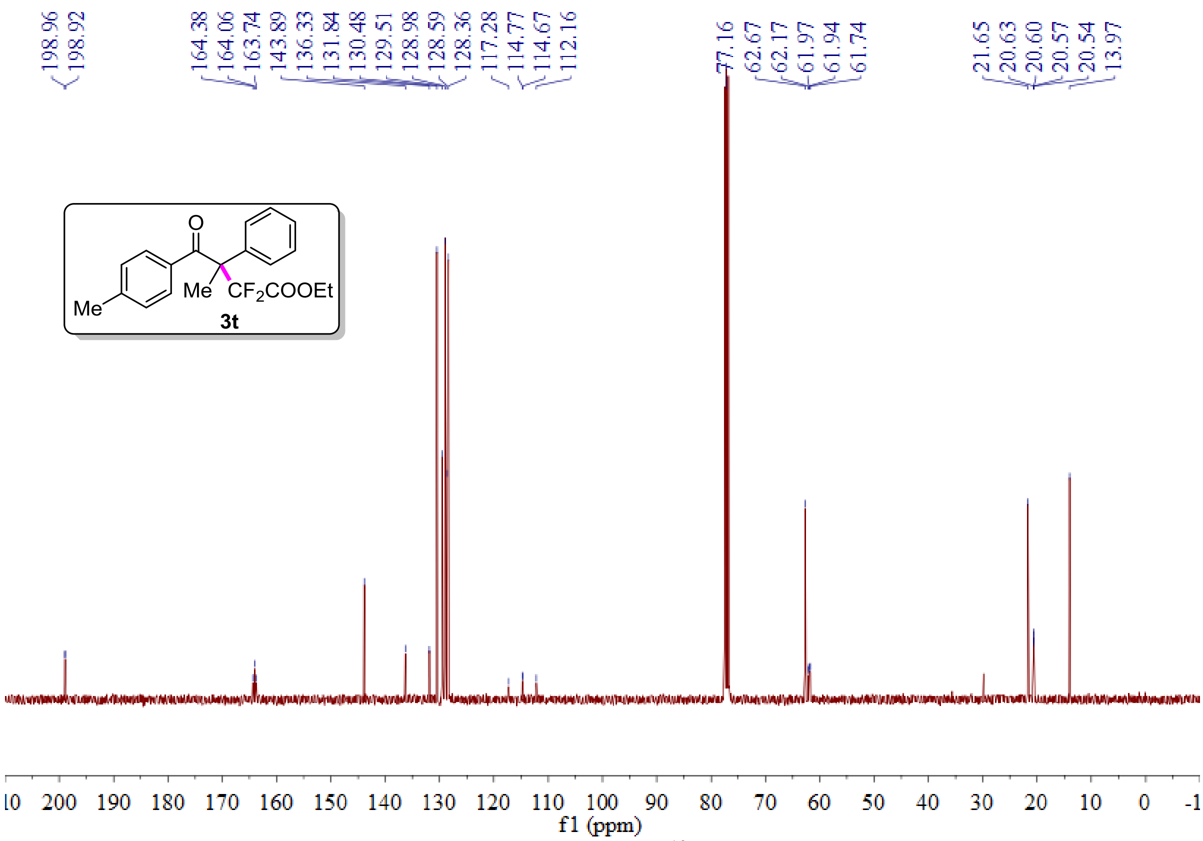


Supplementary Figure 59. $^{13}C$ NMR of 3t

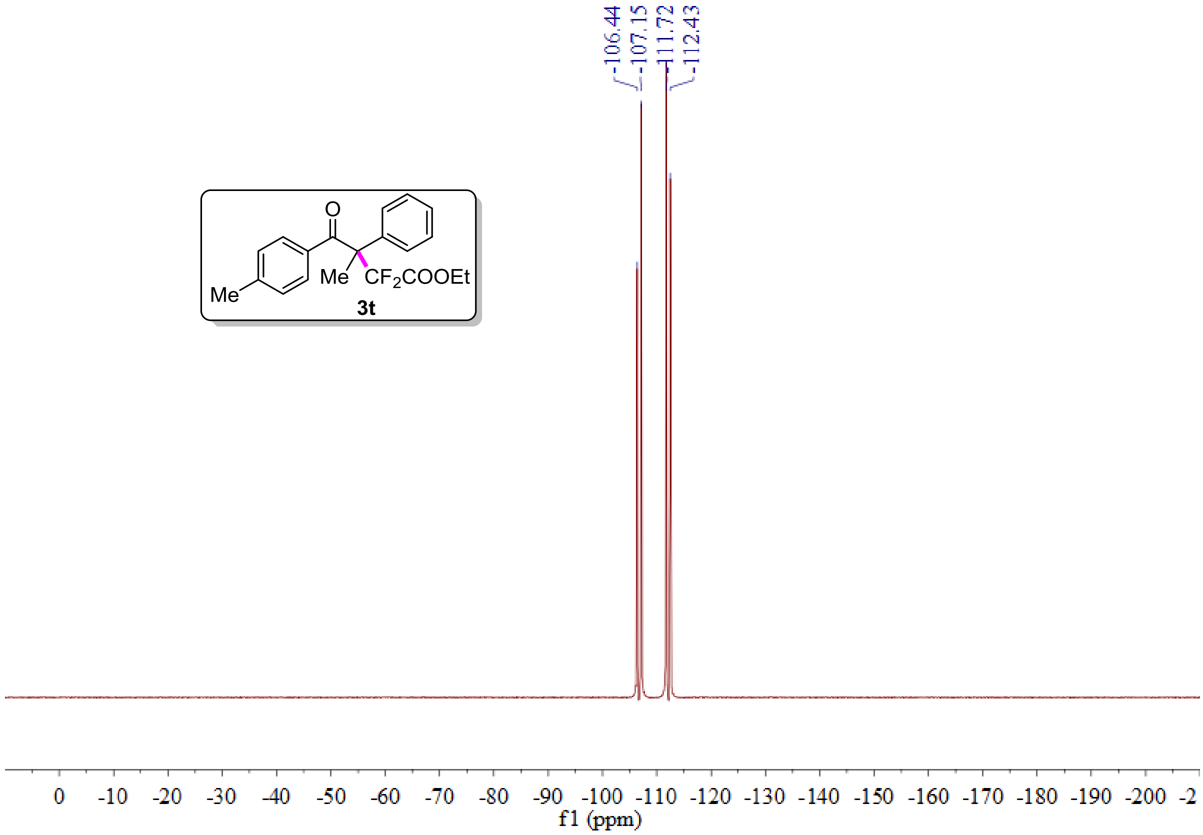


Supplementary Figure 60. $^{19}F$ NMR of 3t

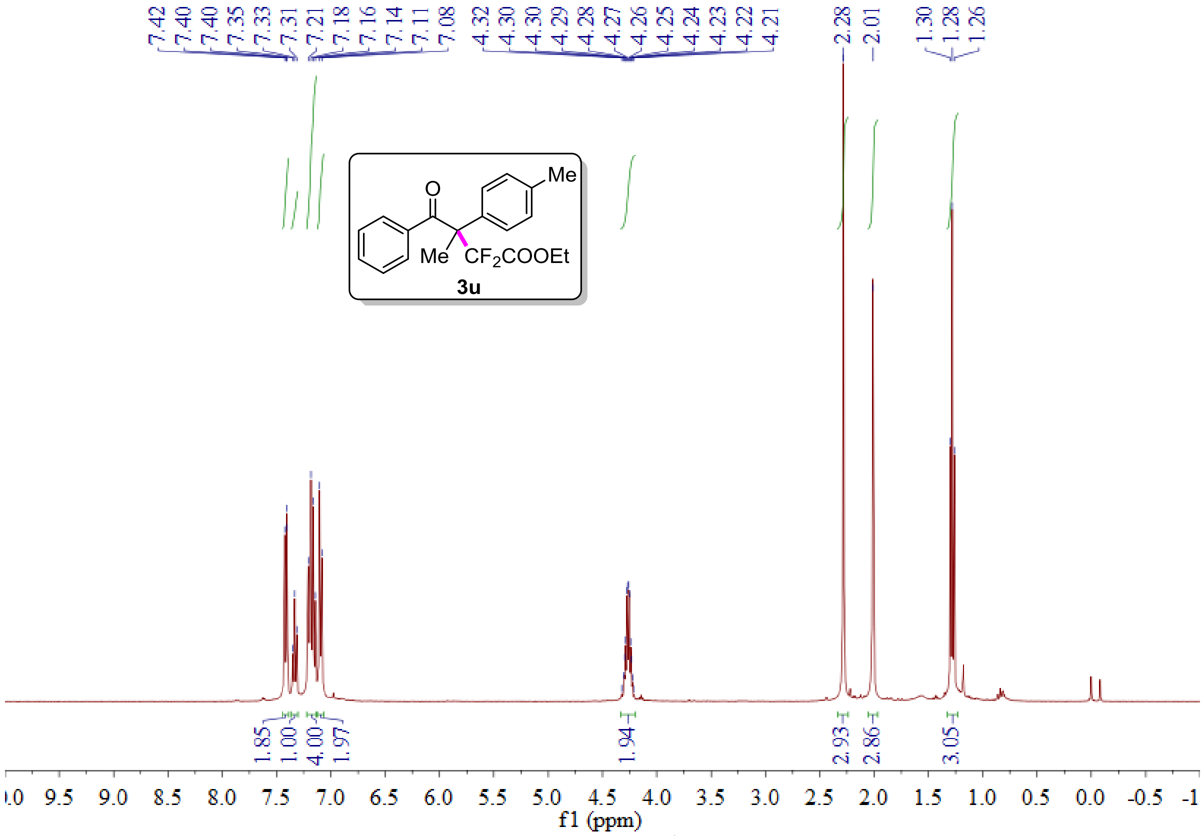

Supplementary Figure 61. $^{1}H$ NMR of 3u

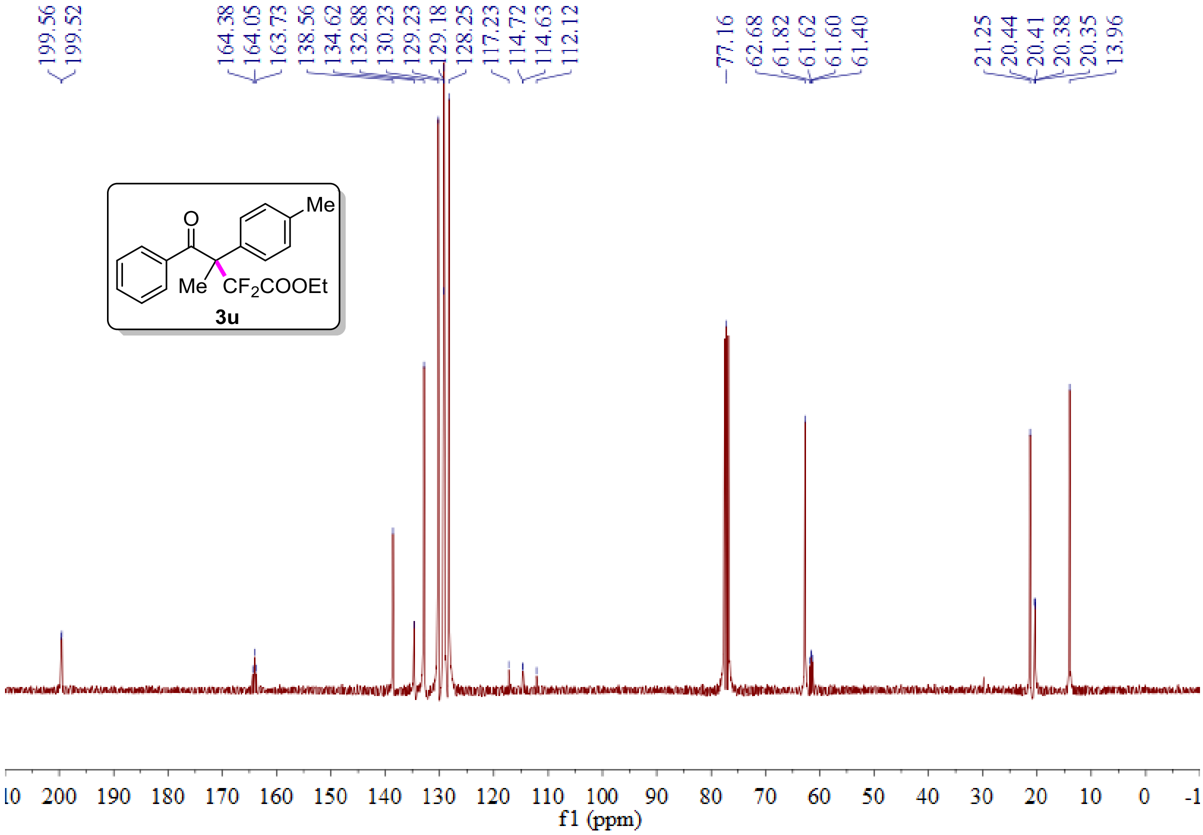

Supplementary Figure 62. $^{13}C$ NMR of 3u

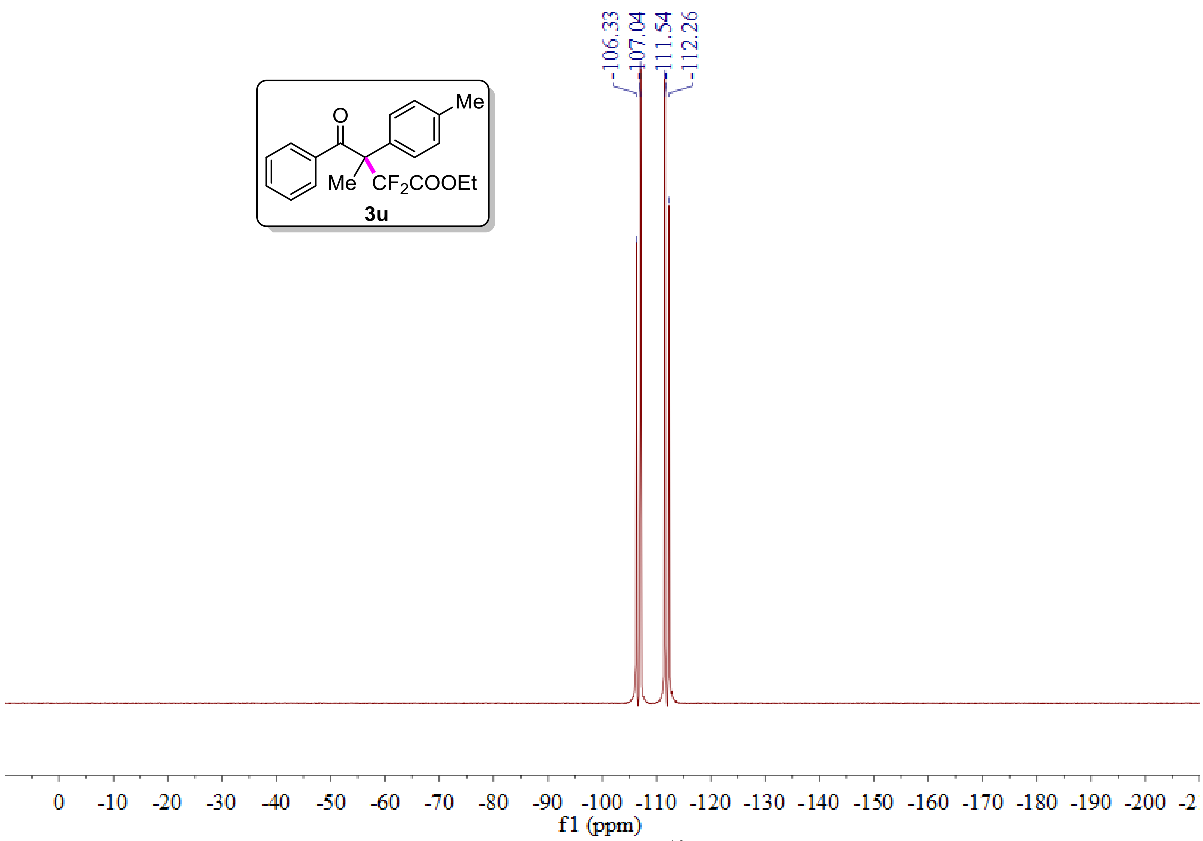


Supplementary Figure 63. $^{19}F$ NMR of 3u

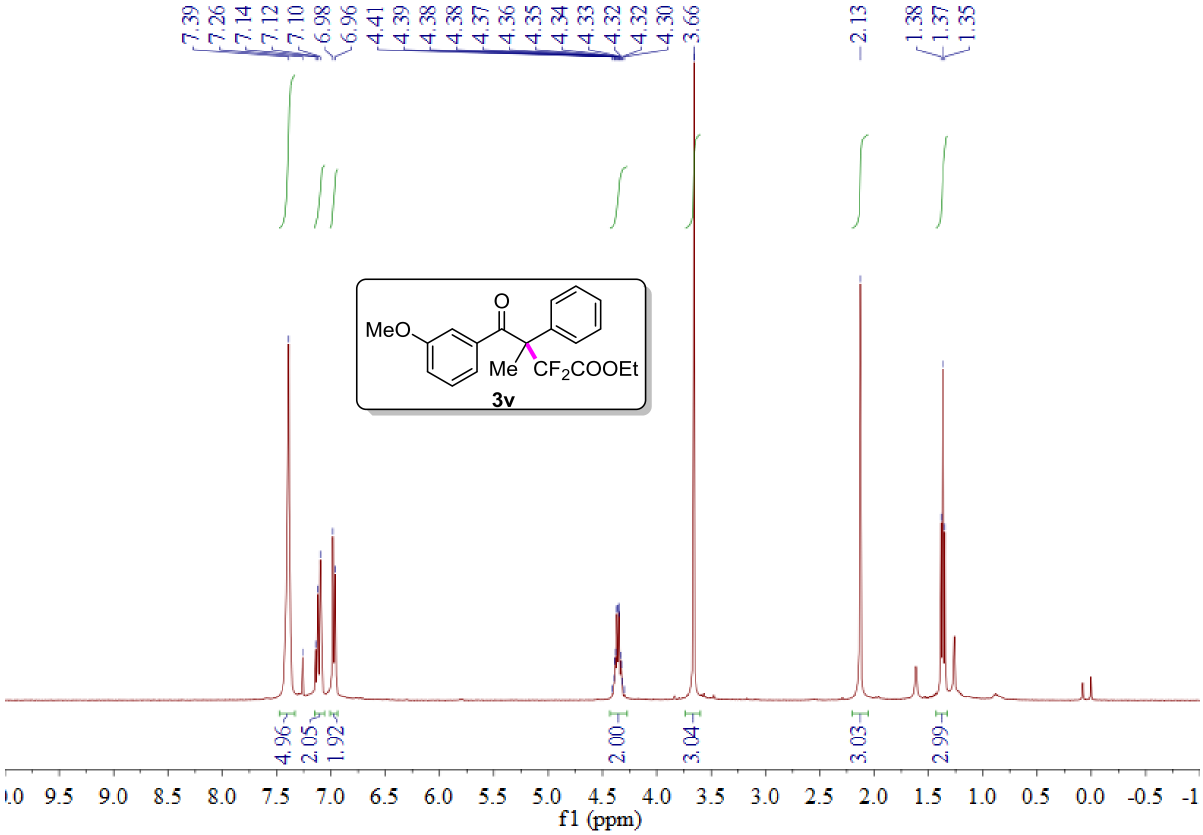


Supplementary Figure 64. $^{1}H$ NMR of 3v

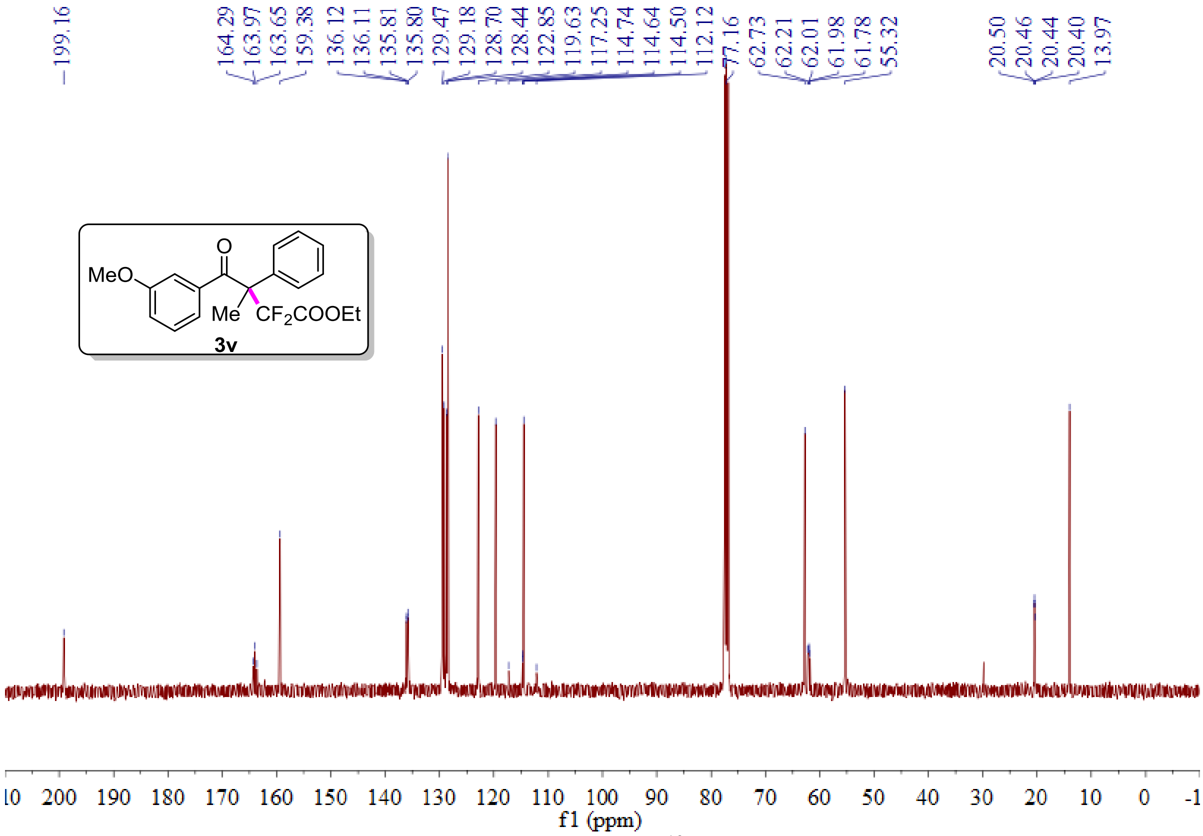

Supplementary Figure 65. $^{13}C$ NMR of 3v

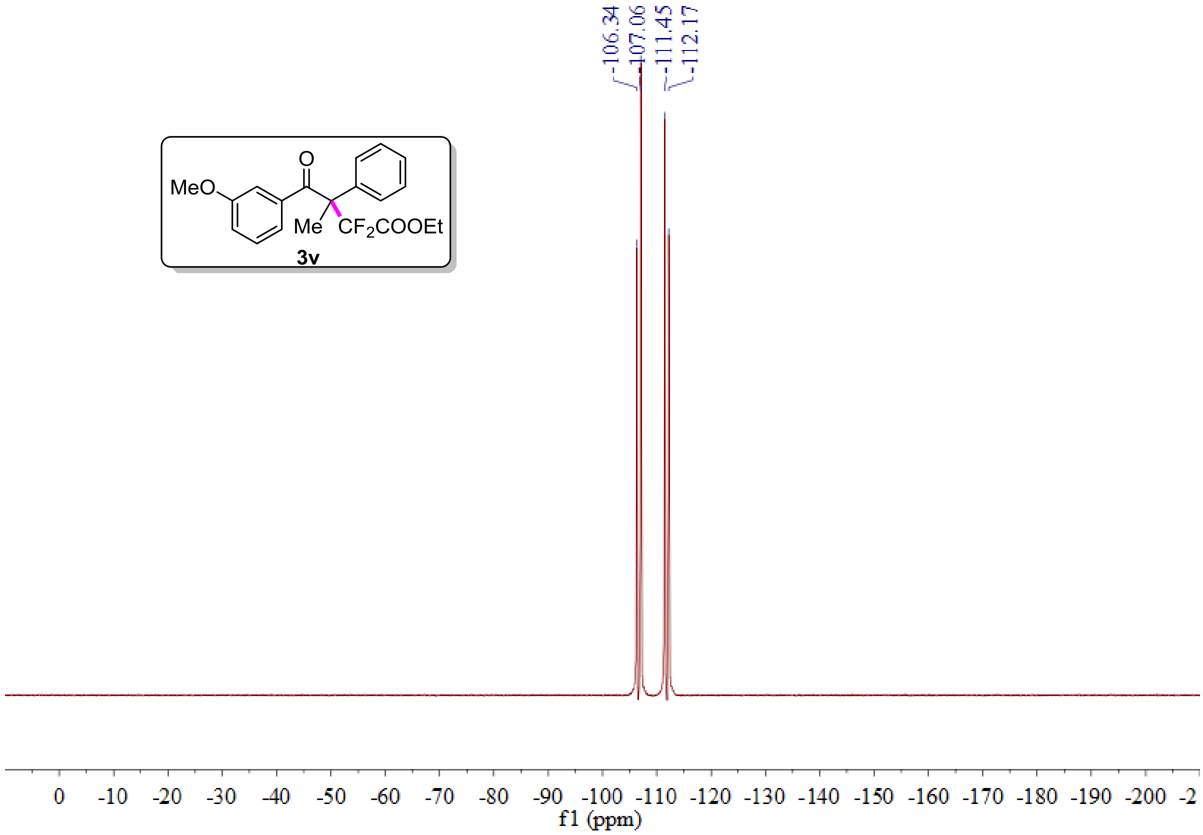

Supplementary Figure 66. $^{19}F$ NMR of 3v

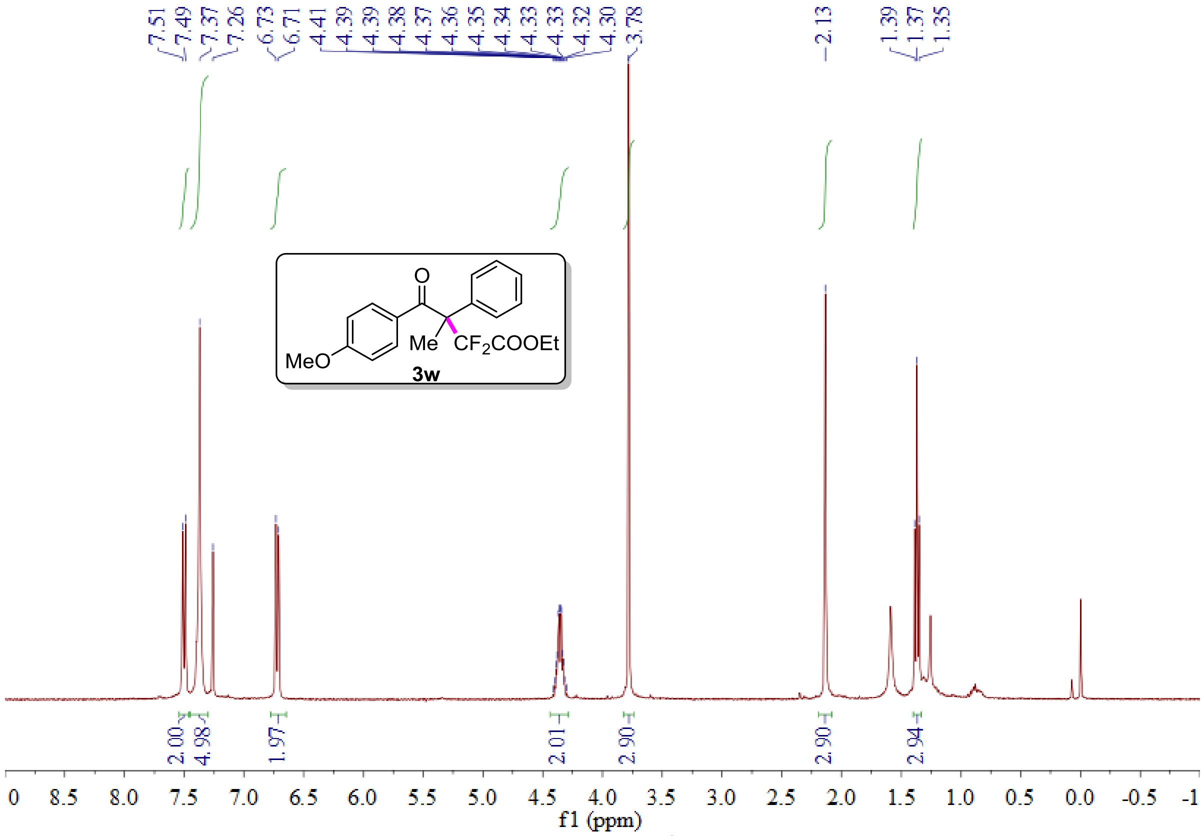

Supplementary Figure 67. $^{1}H$ NMR of 3w

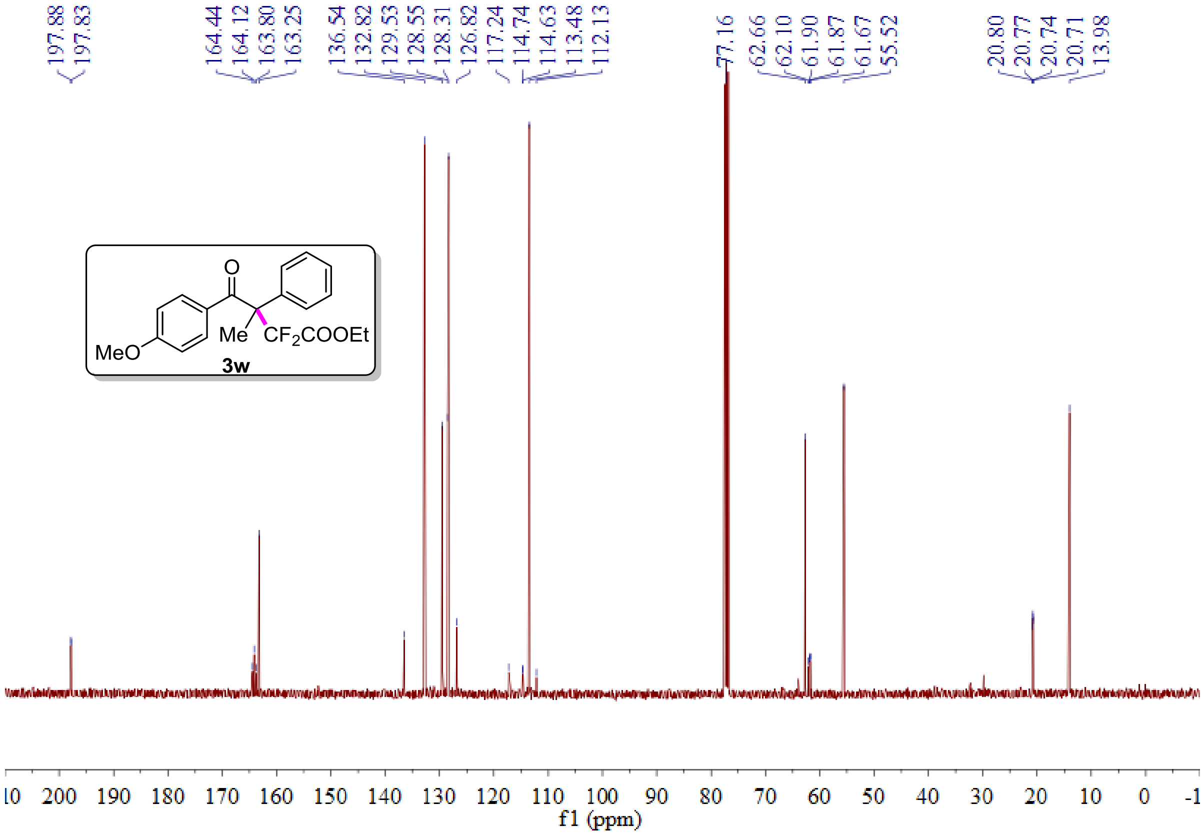

Supplementary Figure 68. $^{13}C$ NMR of 3w

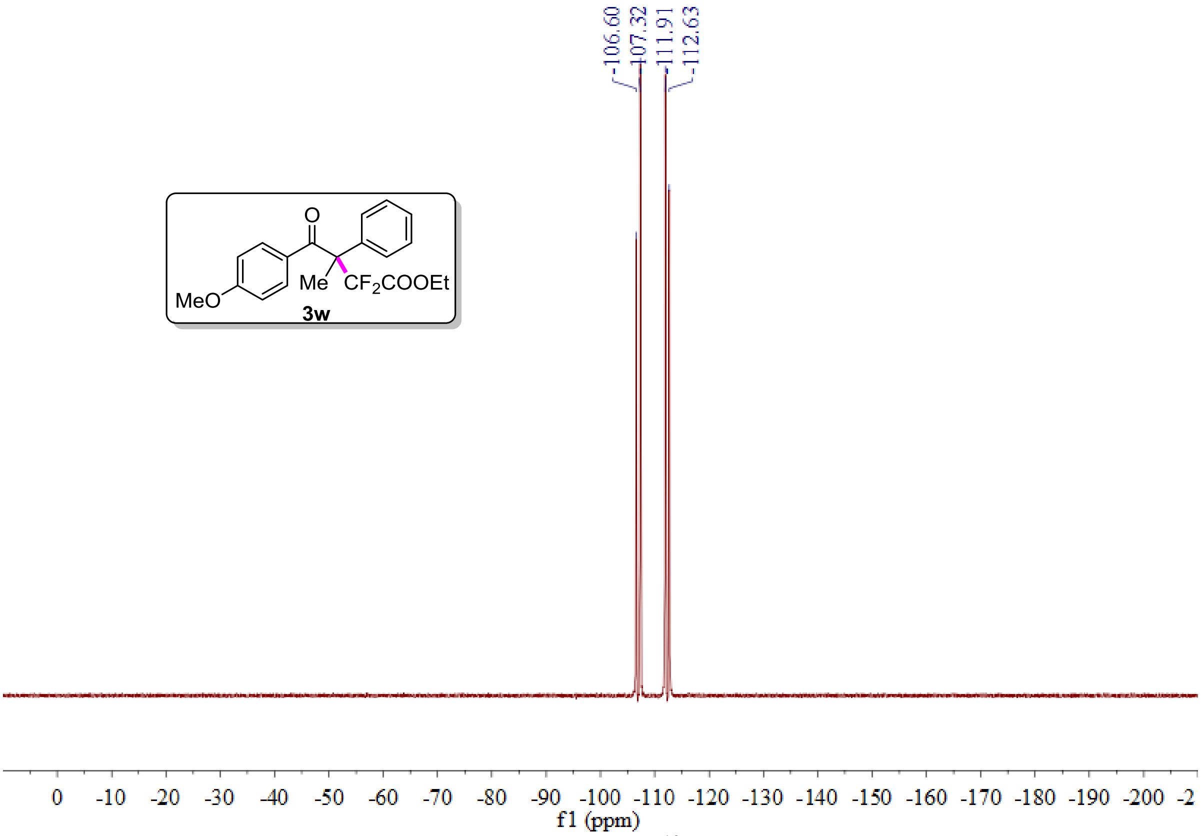


Supplementary Figure 69. $^{19}F$ NMR of 3w

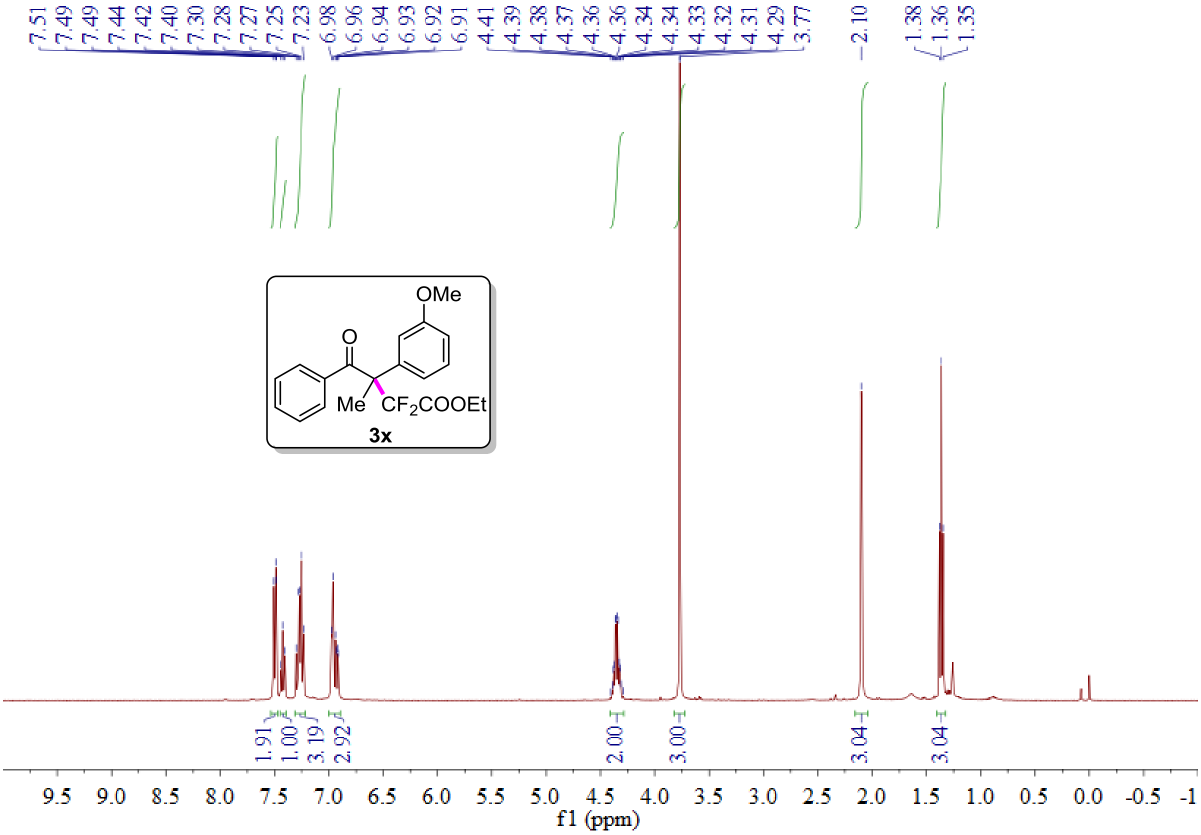


Supplementary Figure 70. $^{1}H$ NMR of 3x

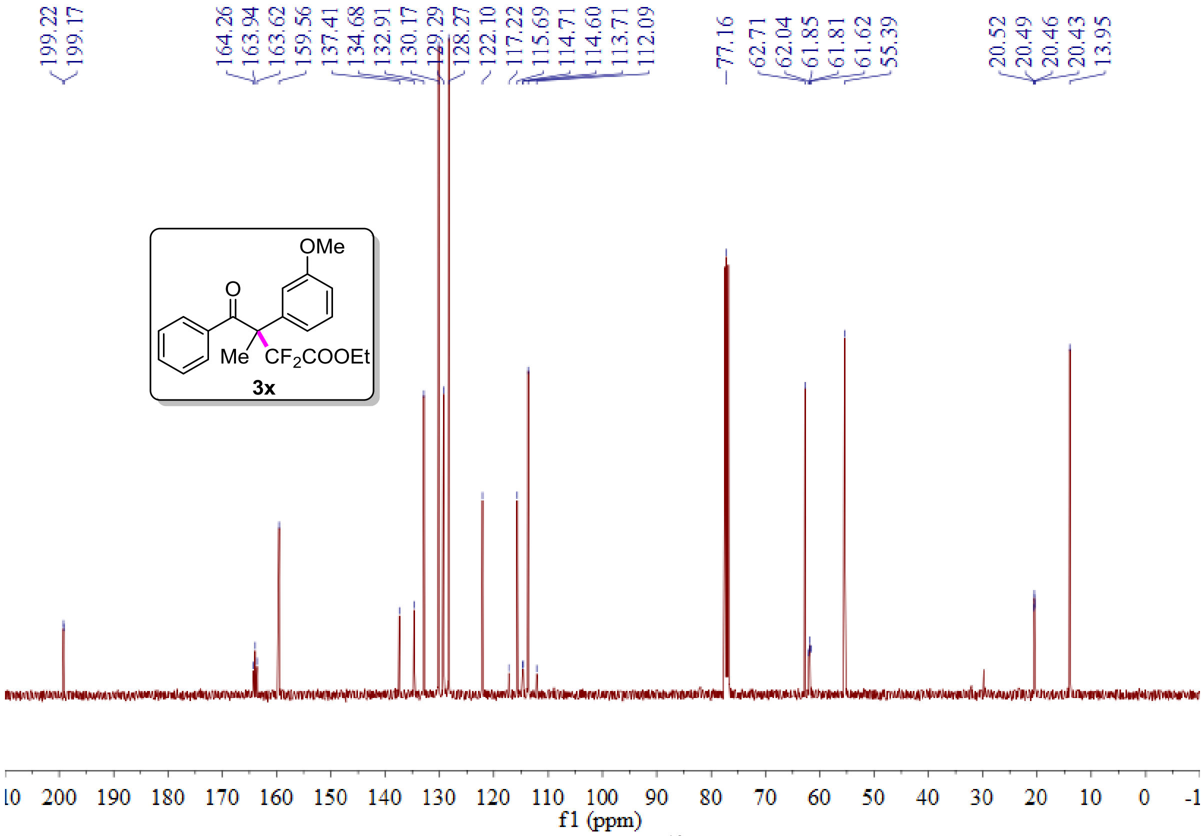


Supplementary Figure 71. $^{13}C$ NMR of 3x

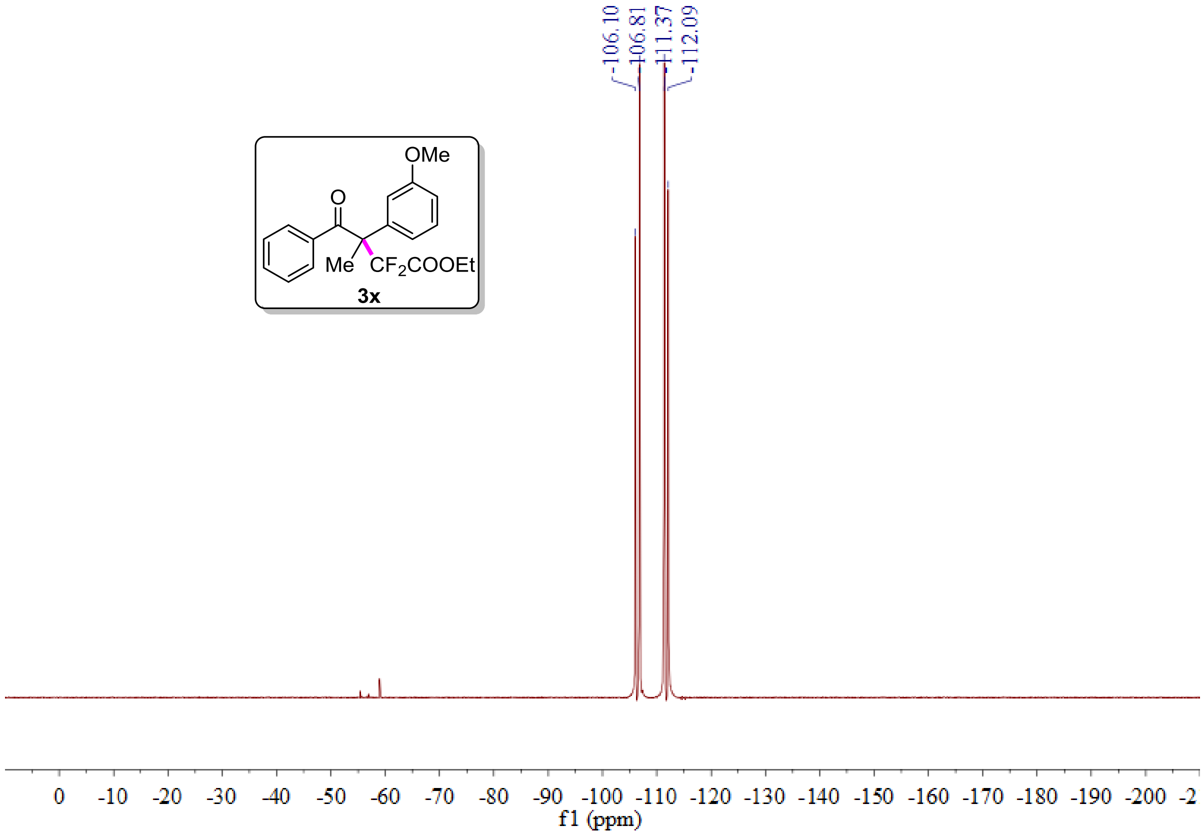


Supplementary Figure 72. $^{19}F$ NMR of 3x

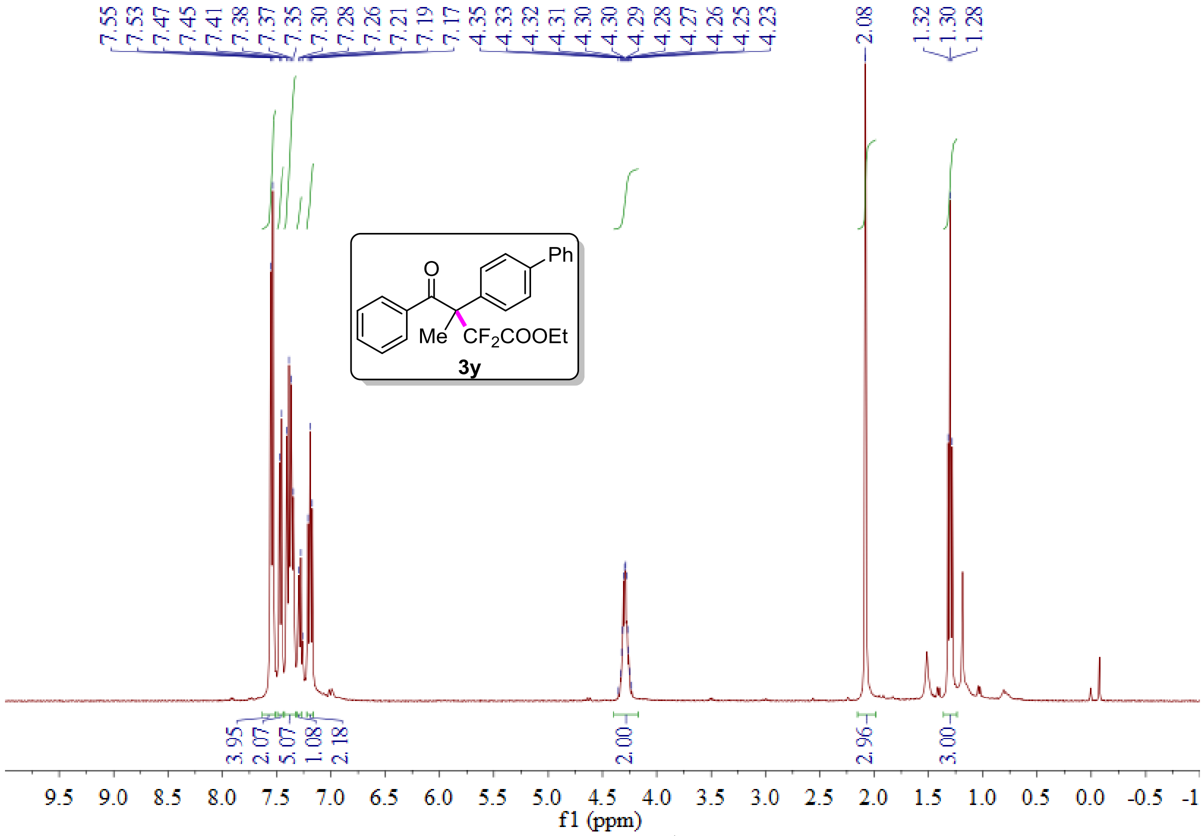


Supplementary Figure 73. $^{1}H$ NMR of 3y

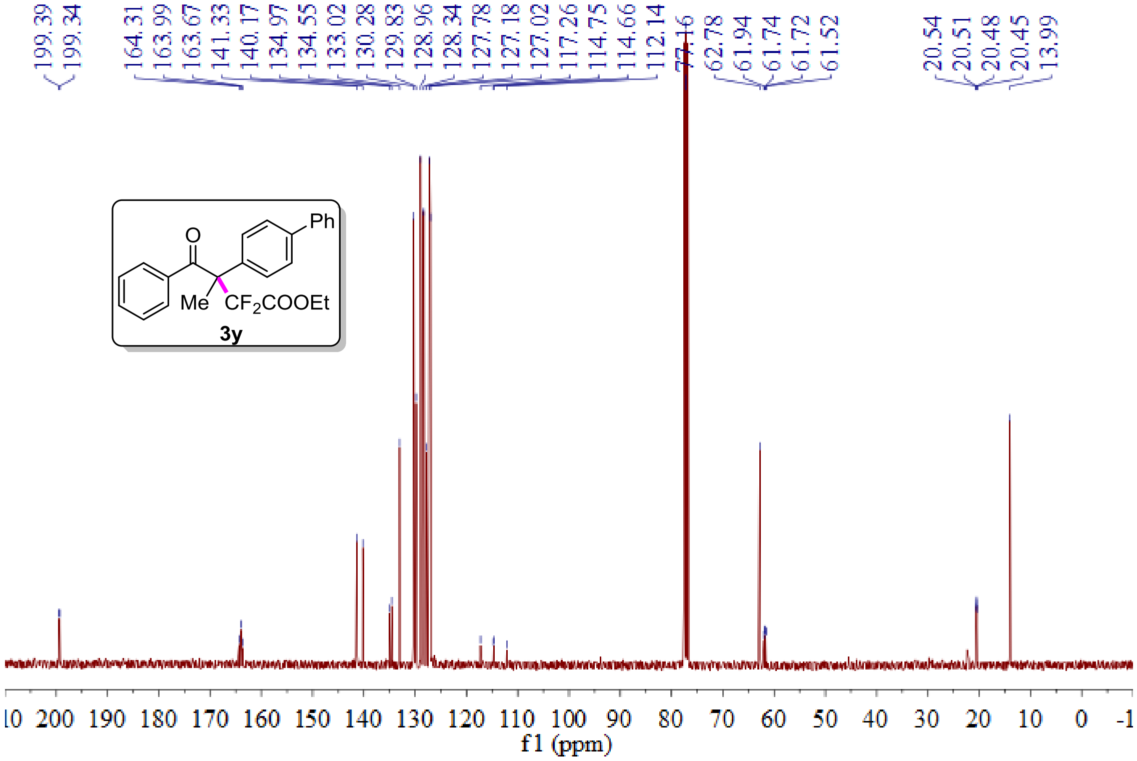


Supplementary Figure 74. $^{13}C$ NMR of 3y

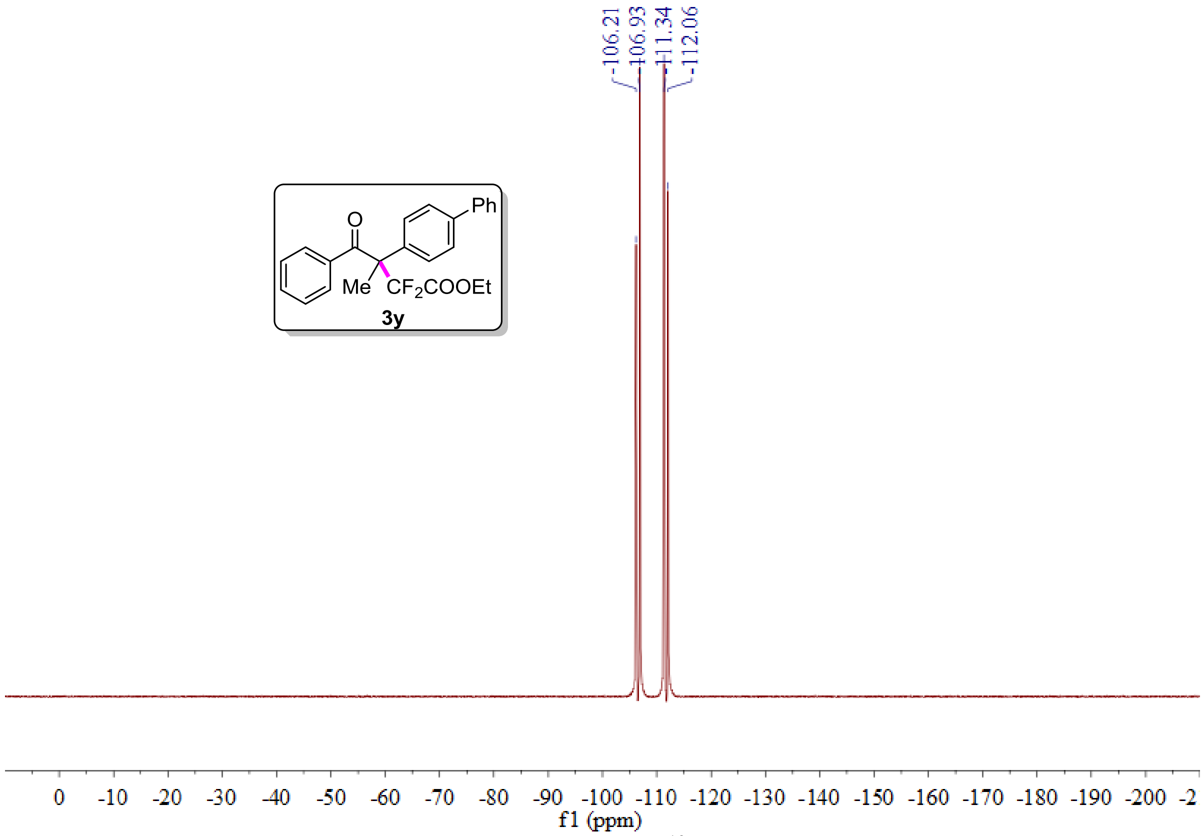


Supplementary Figure 75. $^{19}$F NMR of 3y

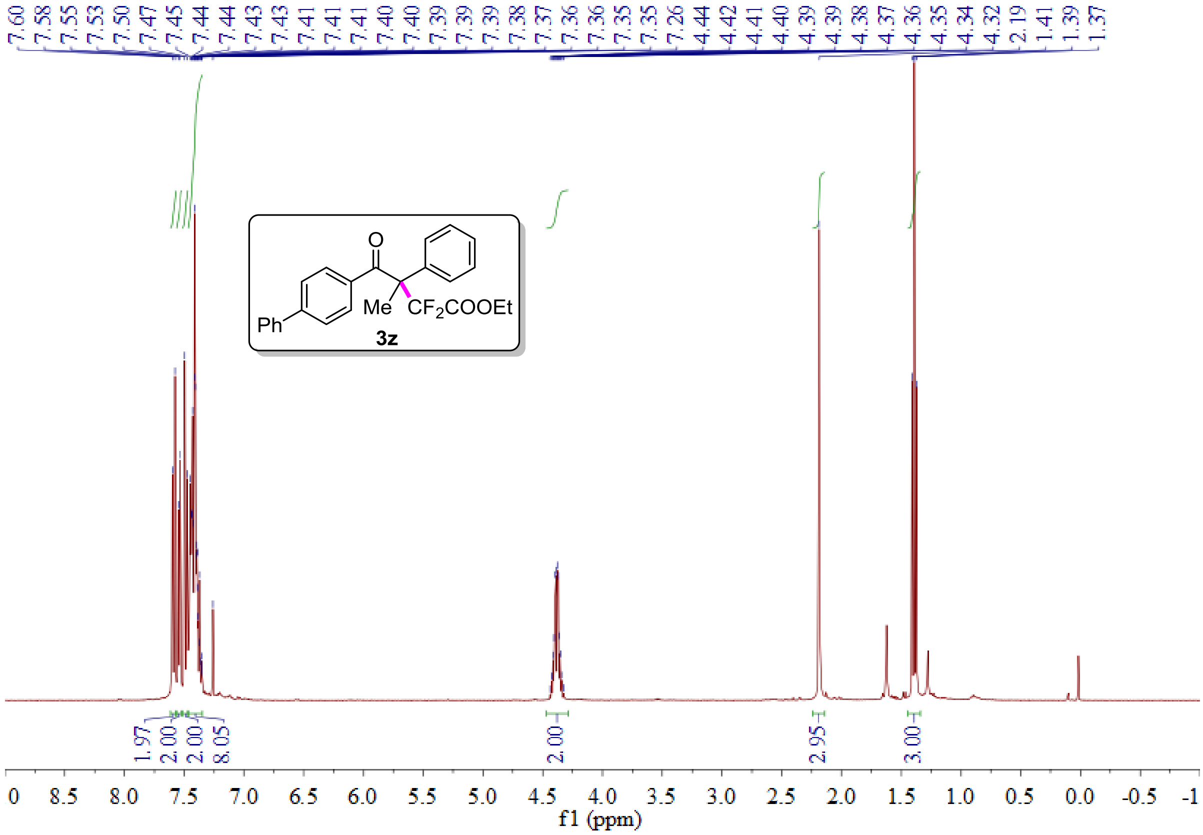


Supplementary Figure 76. $^{1}$H NMR of 3z

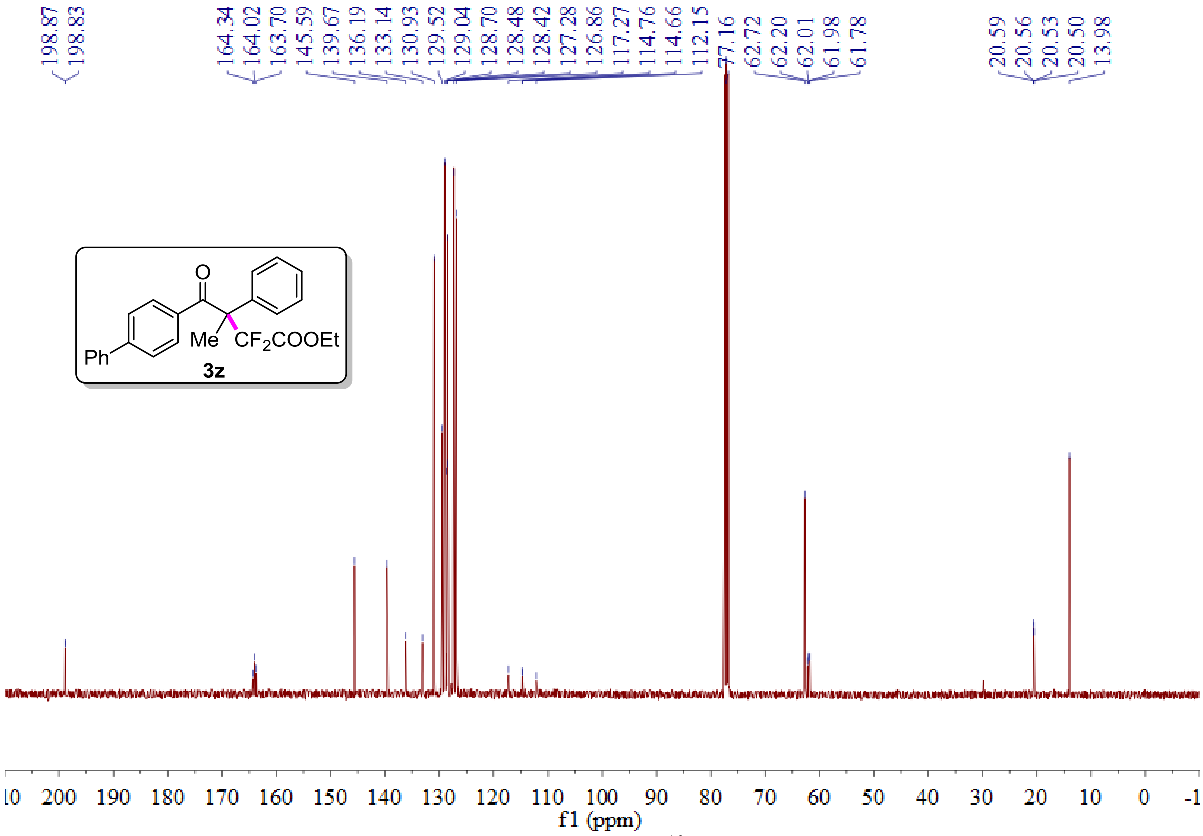


Supplementary Figure 77. $^{13}C$ NMR of 3z

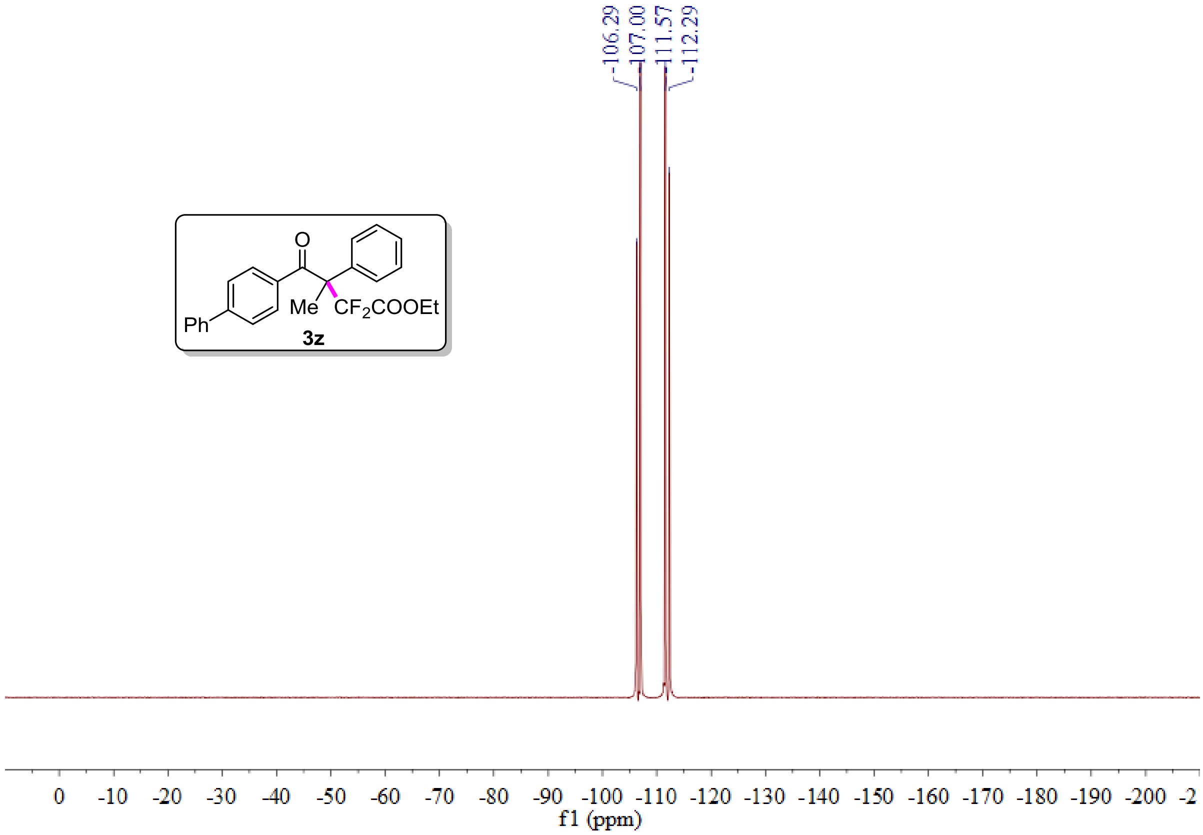


Supplementary Figure 78. $^{19}F$ NMR of 3z

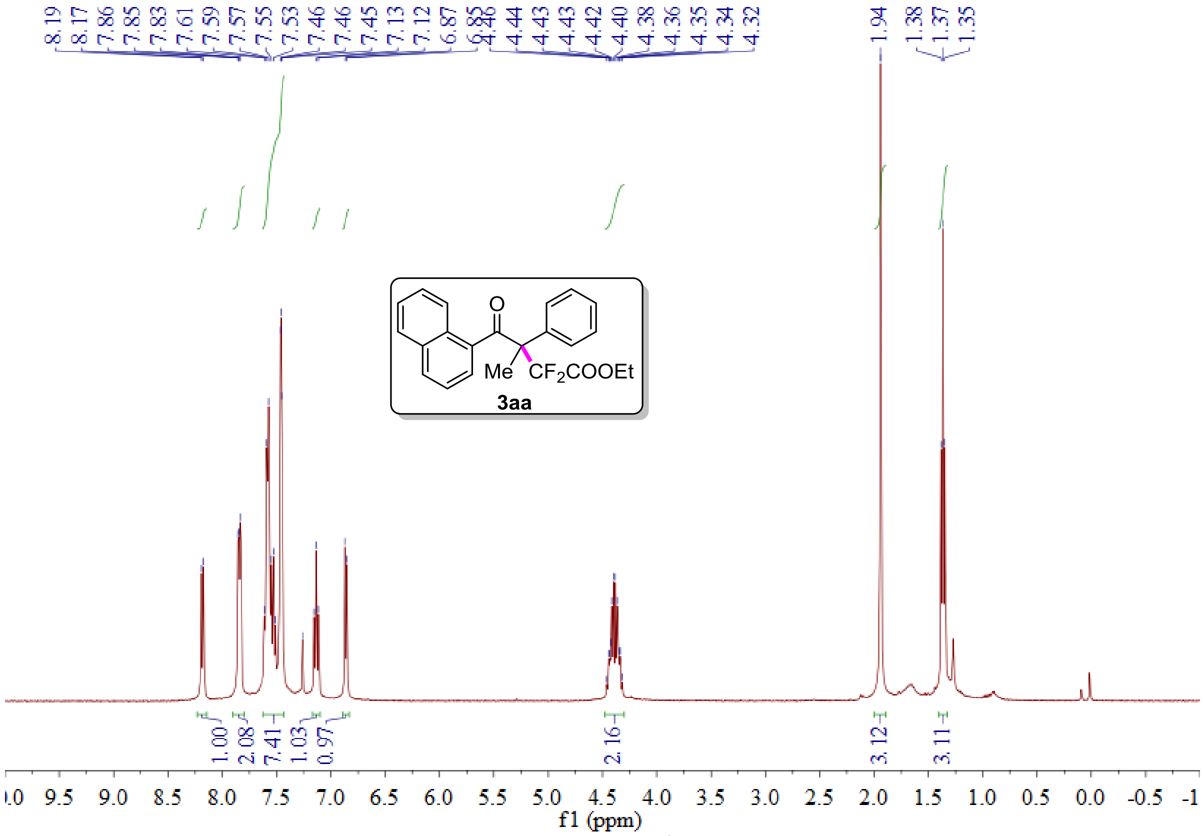

Supplementary Figure 79. $^{1}$H NMR of 3aa

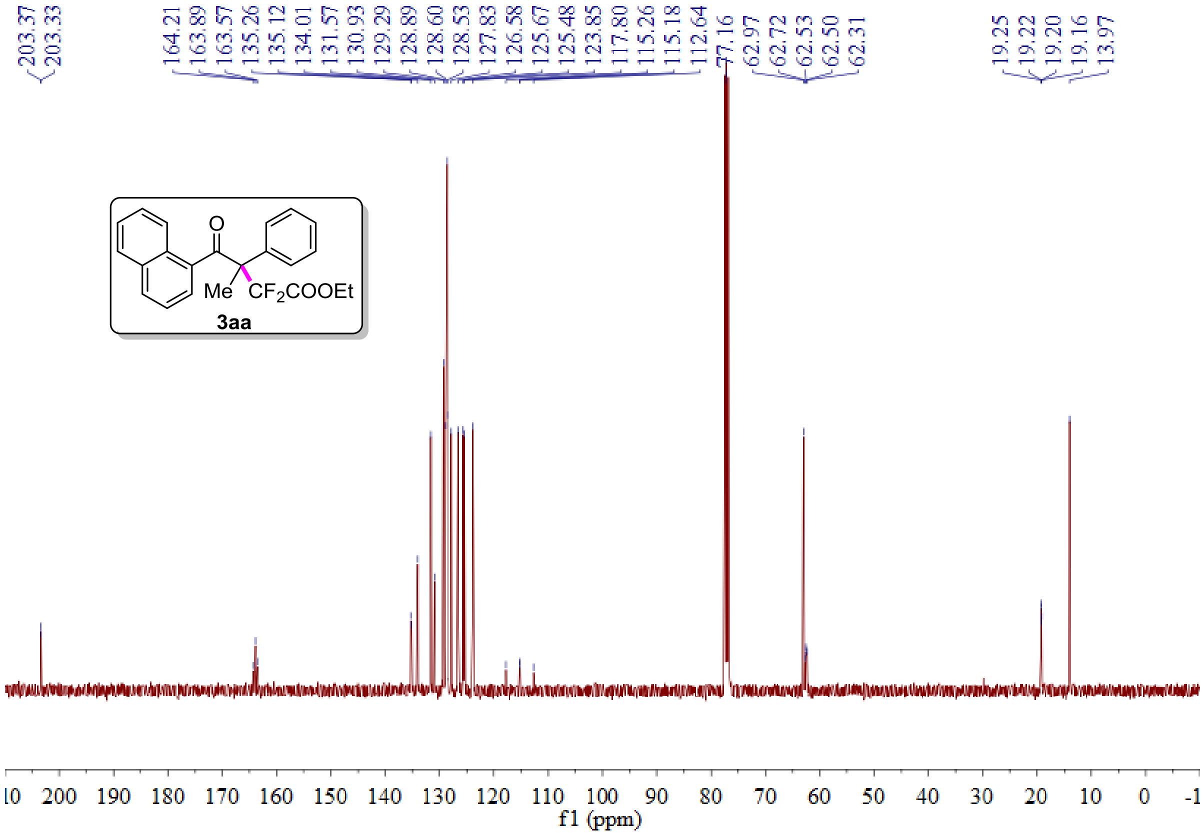

Supplementary Figure 80. $^{13}$C NMR of 3aa

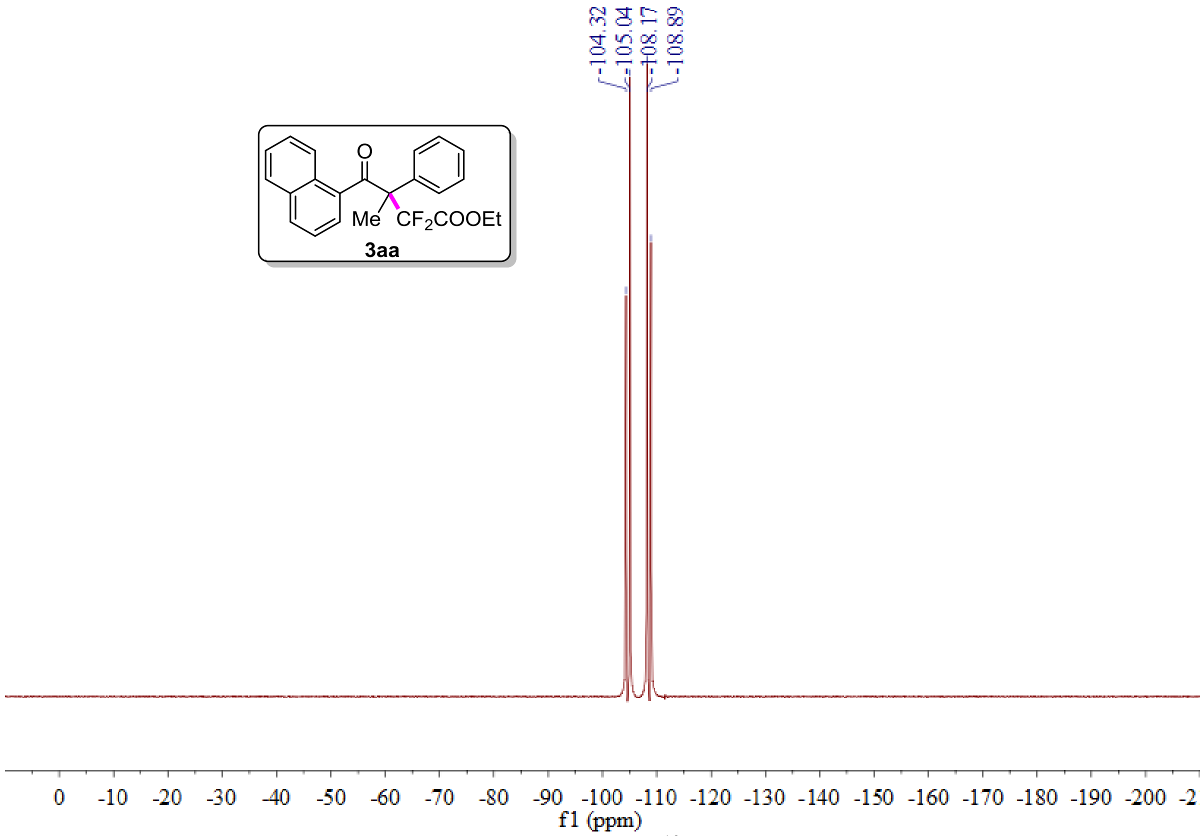


Supplementary Figure 81. $^{19}$F NMR of 3aa

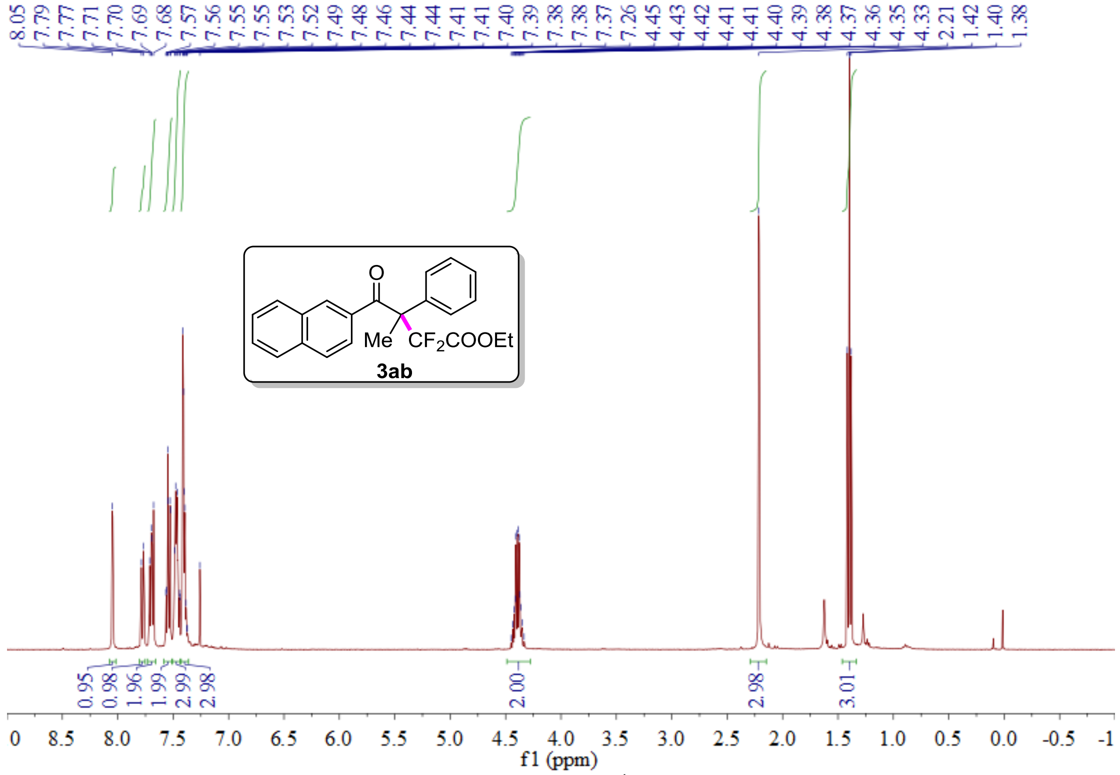


Supplementary Figure 82. $^{1}$H NMR of 3ab

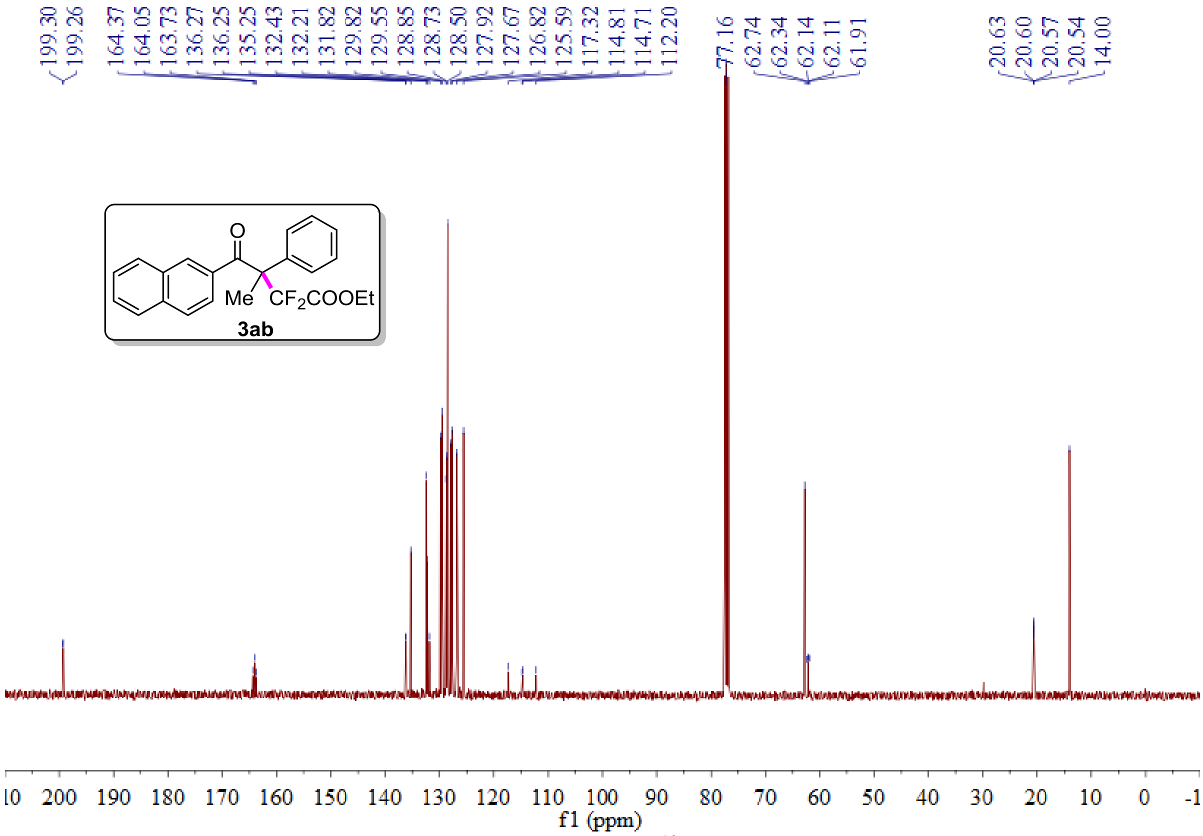


Supplementary Figure 83. $^{13}C$ NMR of 3ab

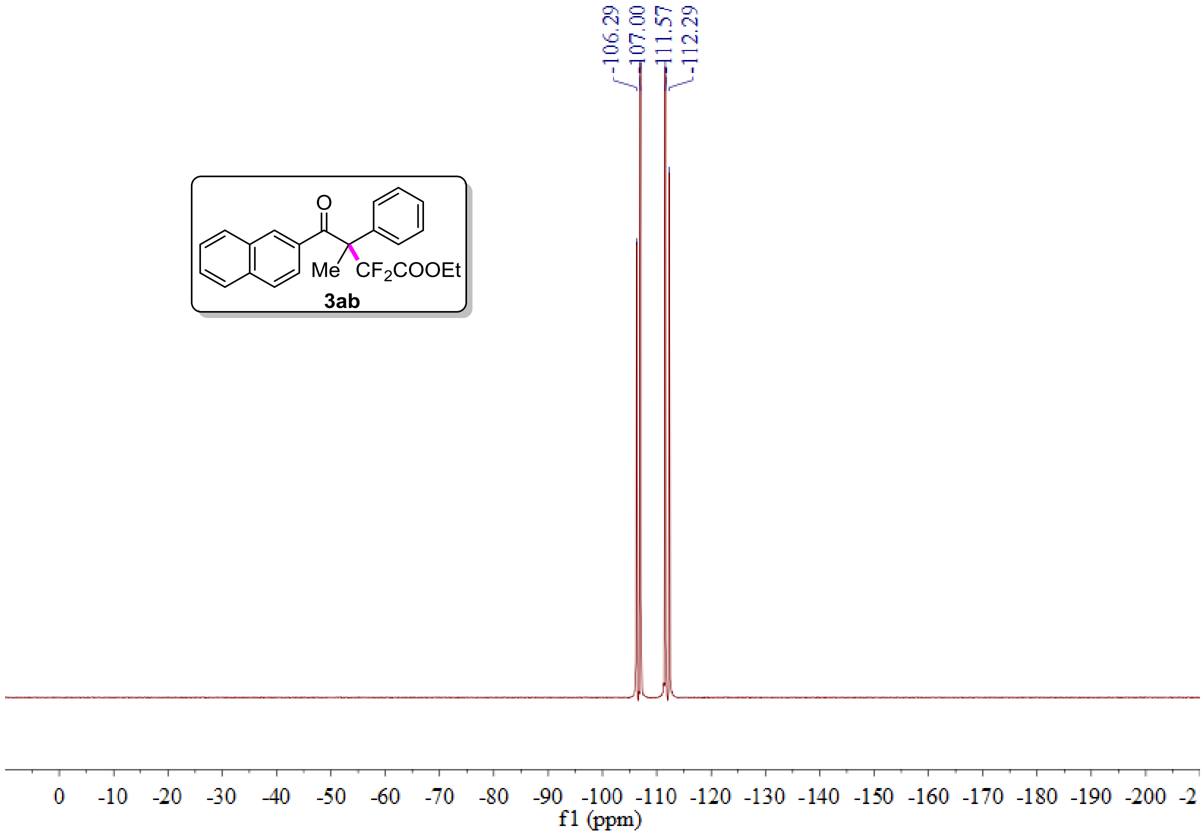


Supplementary Figure 84. $^{19}F$ NMR of 3ab

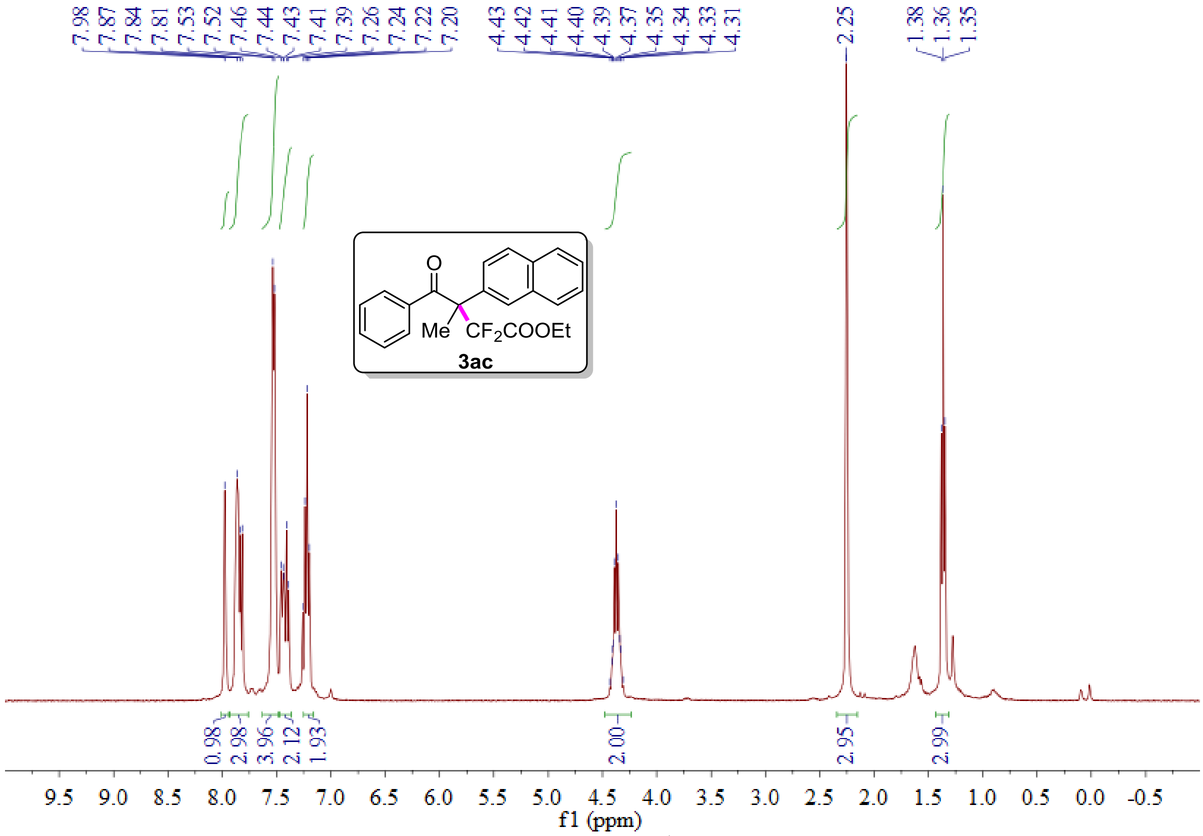


Supplementary Figure 85. $^{1}$H NMR of 3ac

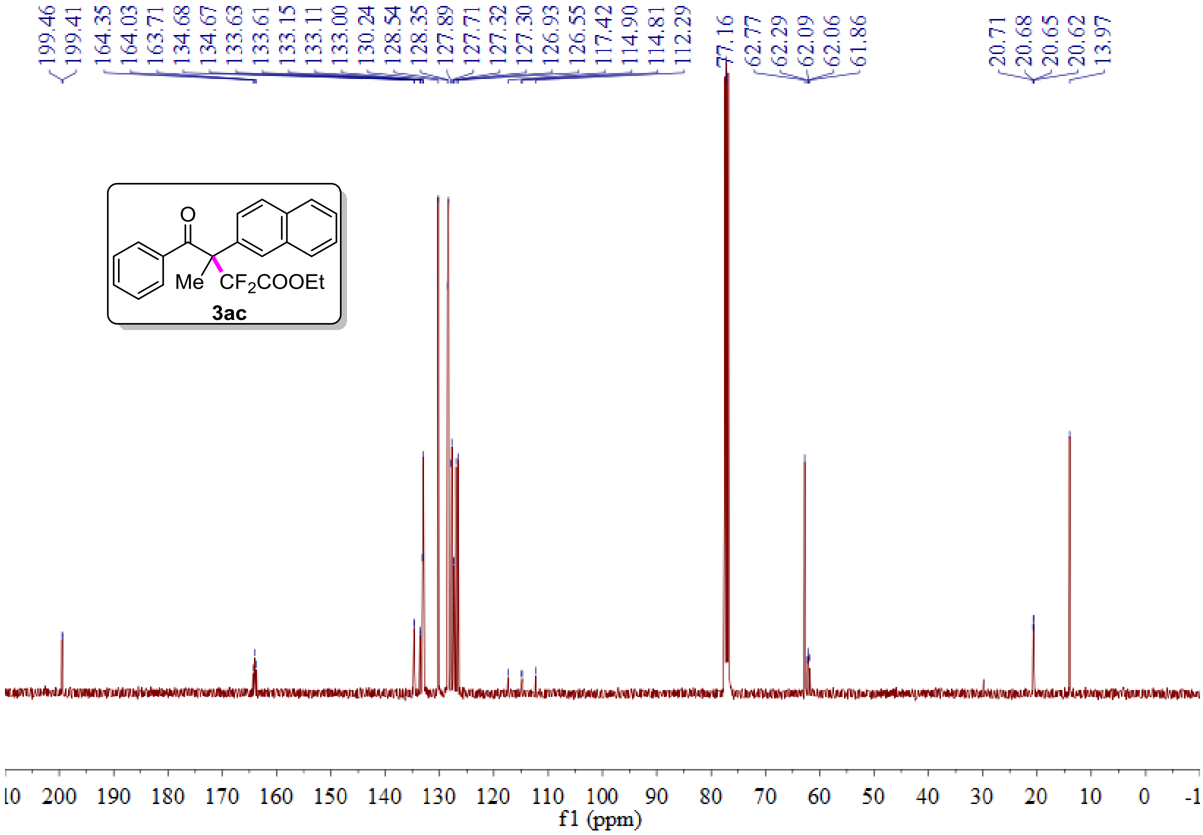


Supplementary Figure 86. $^{13}$C NMR of 3ac

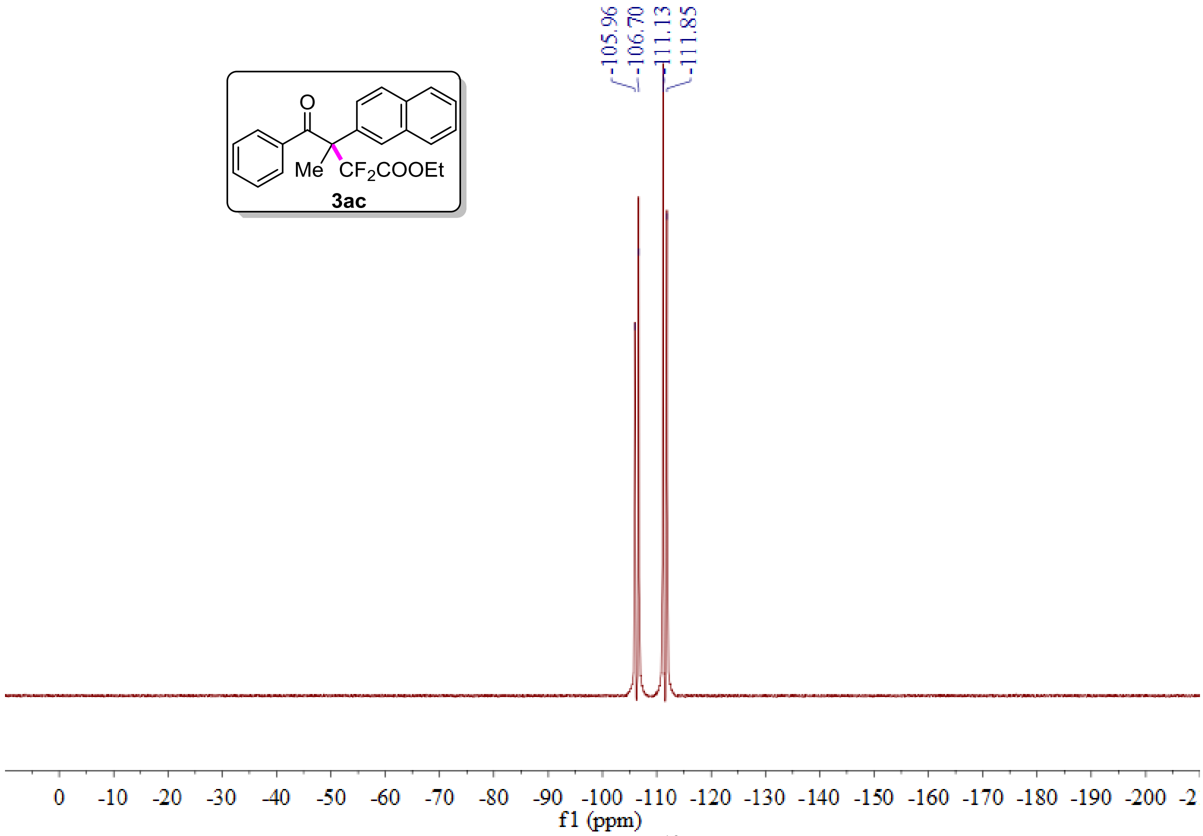


Supplementary Figure 87. $^{19}$F NMR of 3ac

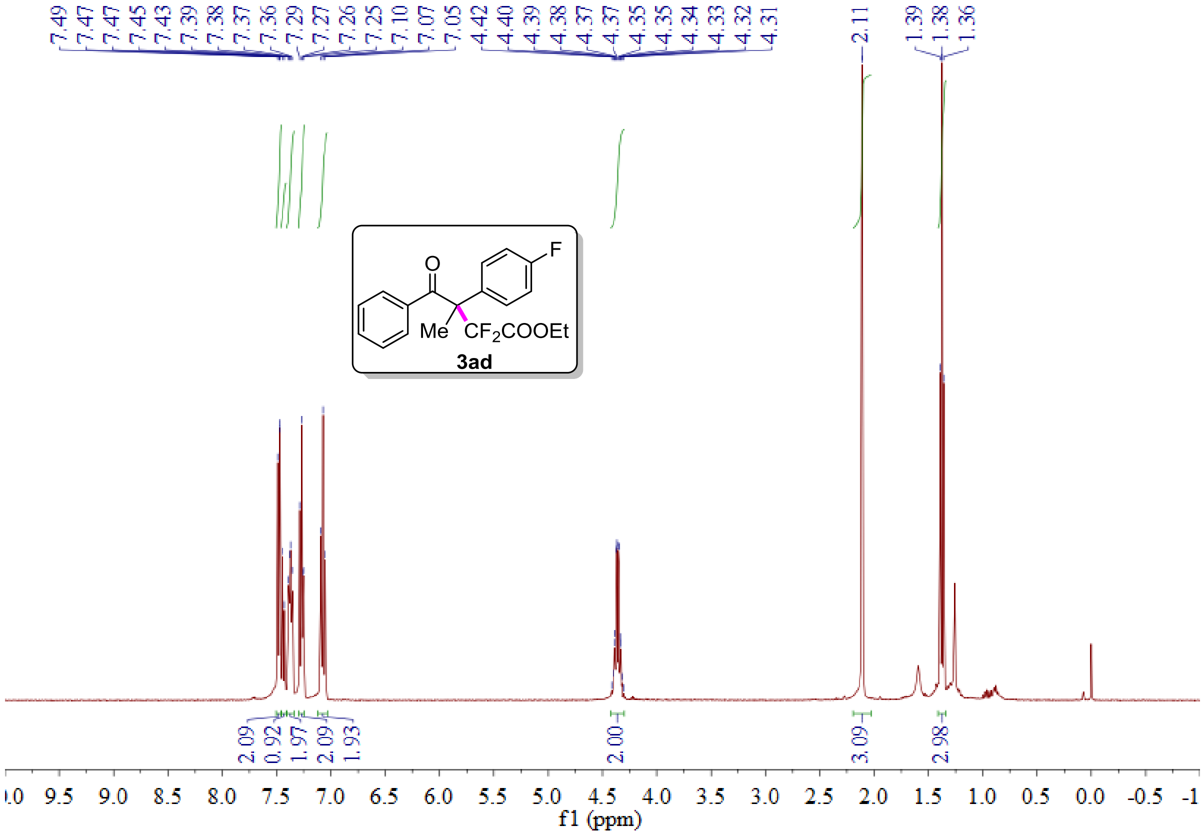


Supplementary Figure 88. $^{1}$H NMR of 3ad

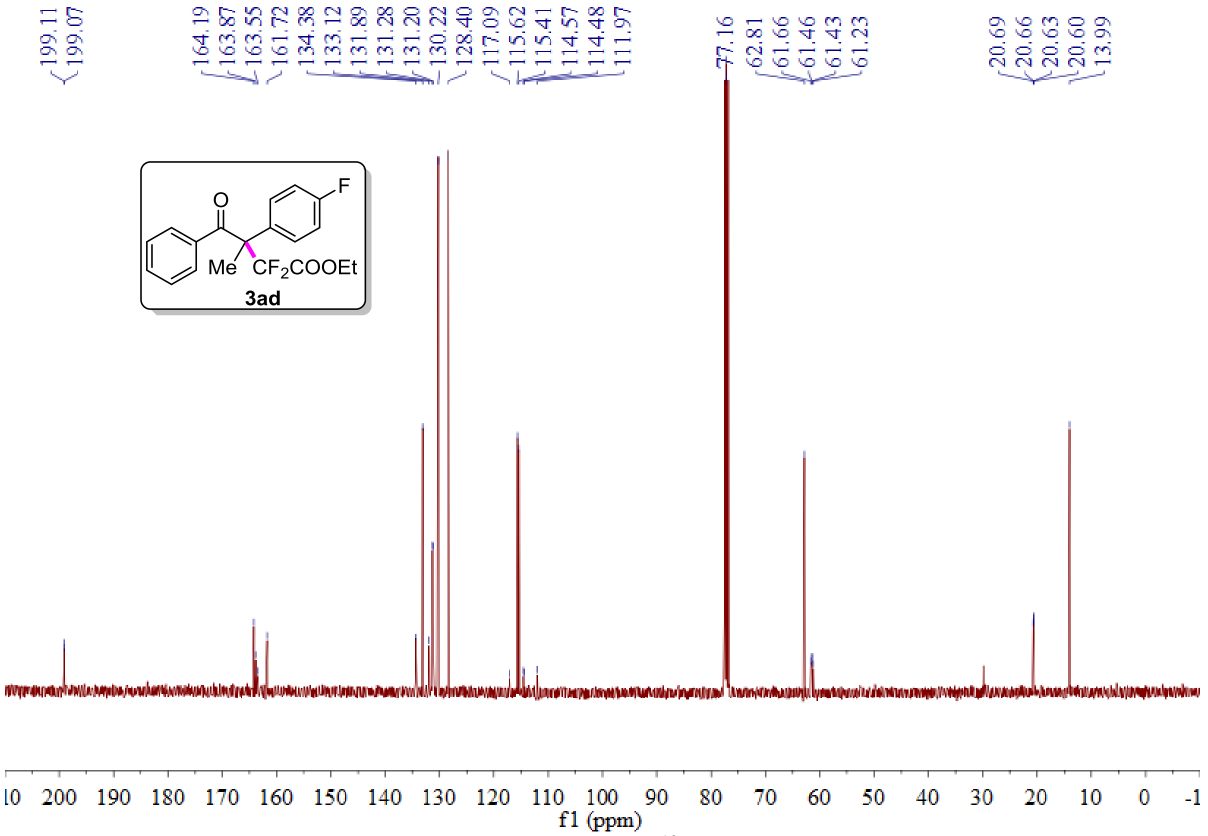

Supplementary Figure 89. $^{13}C$ NMR of 3ad

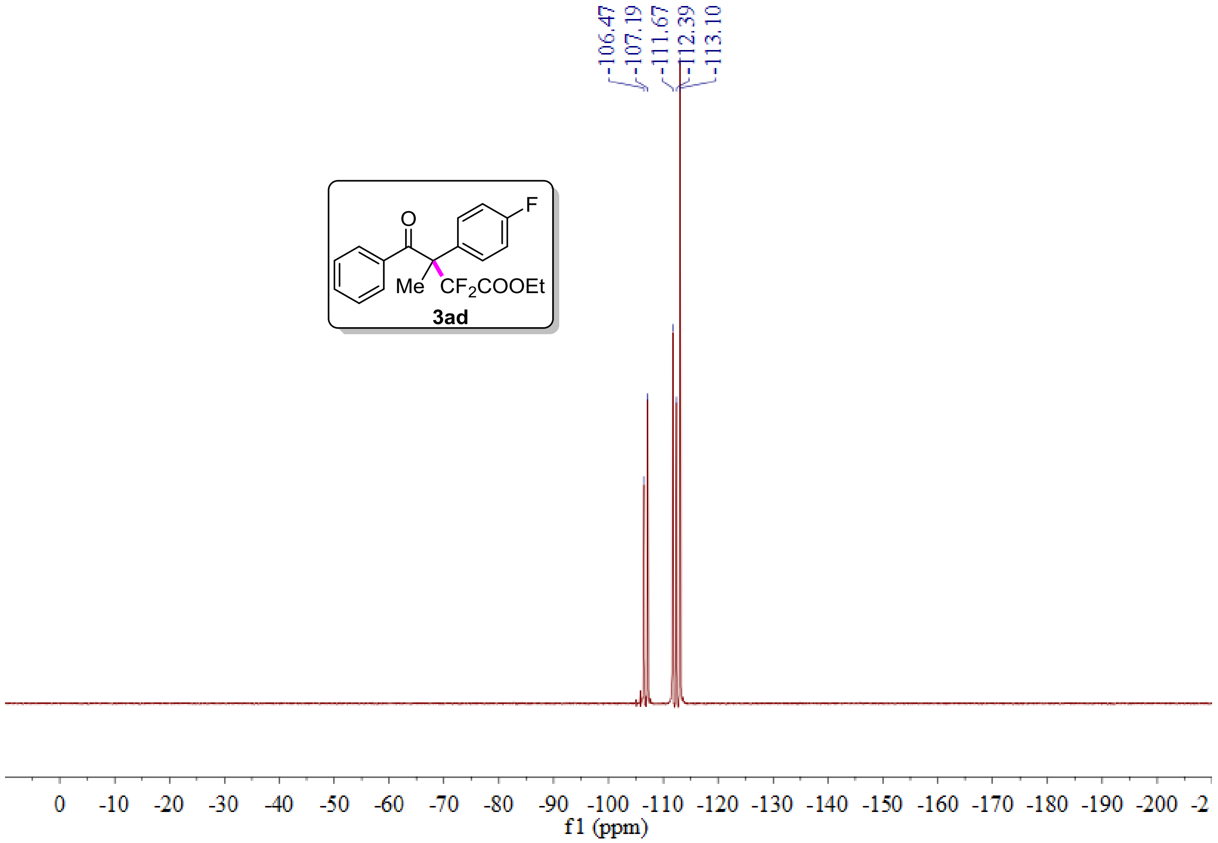

Supplementary Figure 90. $^{19}F$ NMR of 3ad

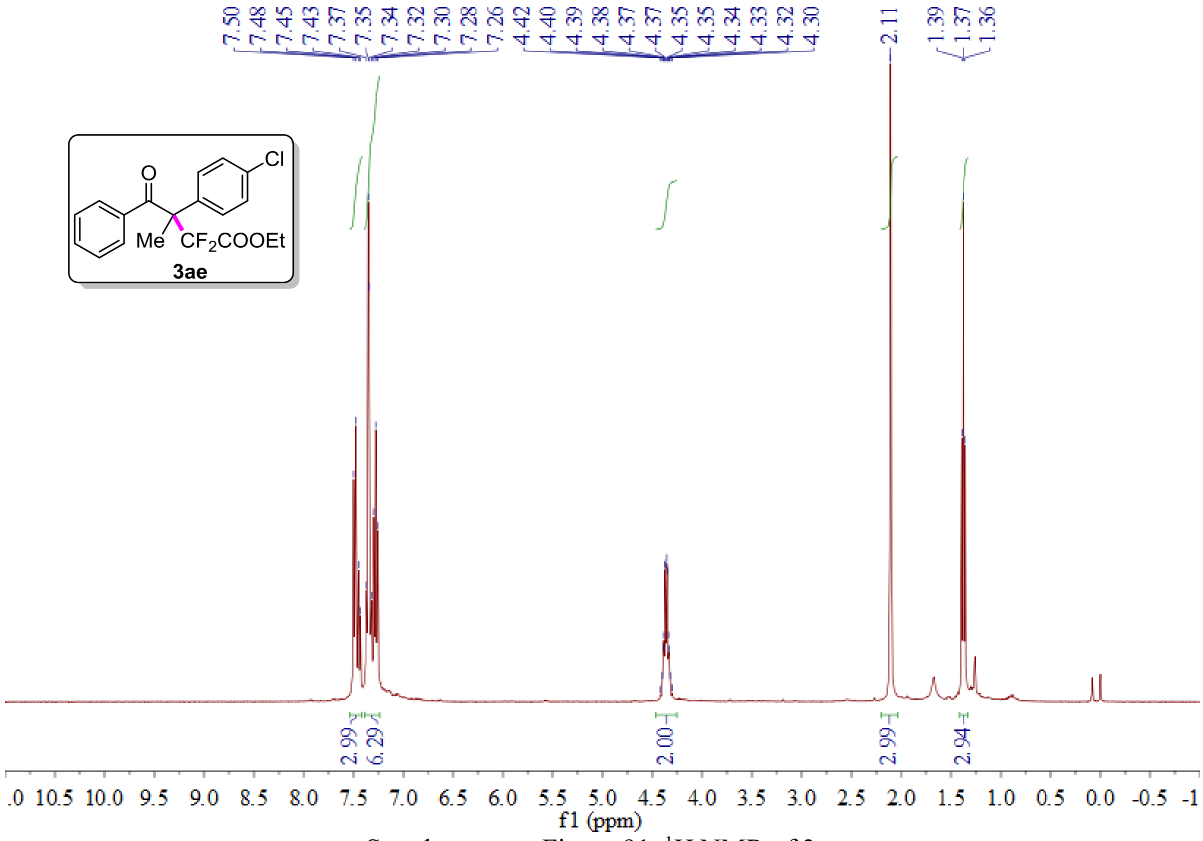


Supplementary Figure 91. $^{1}$H NMR of 3ae

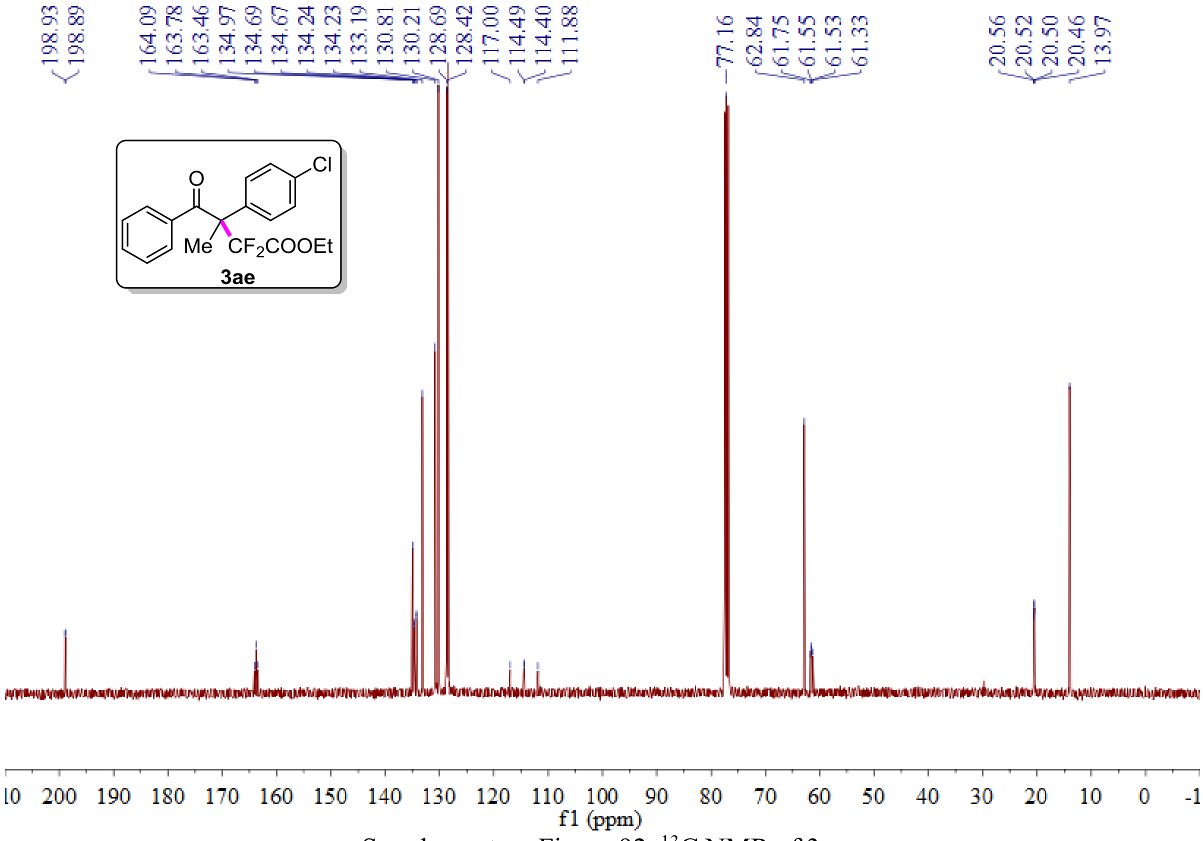


Supplementary Figure 92. $^{13}$C NMR of 3ae

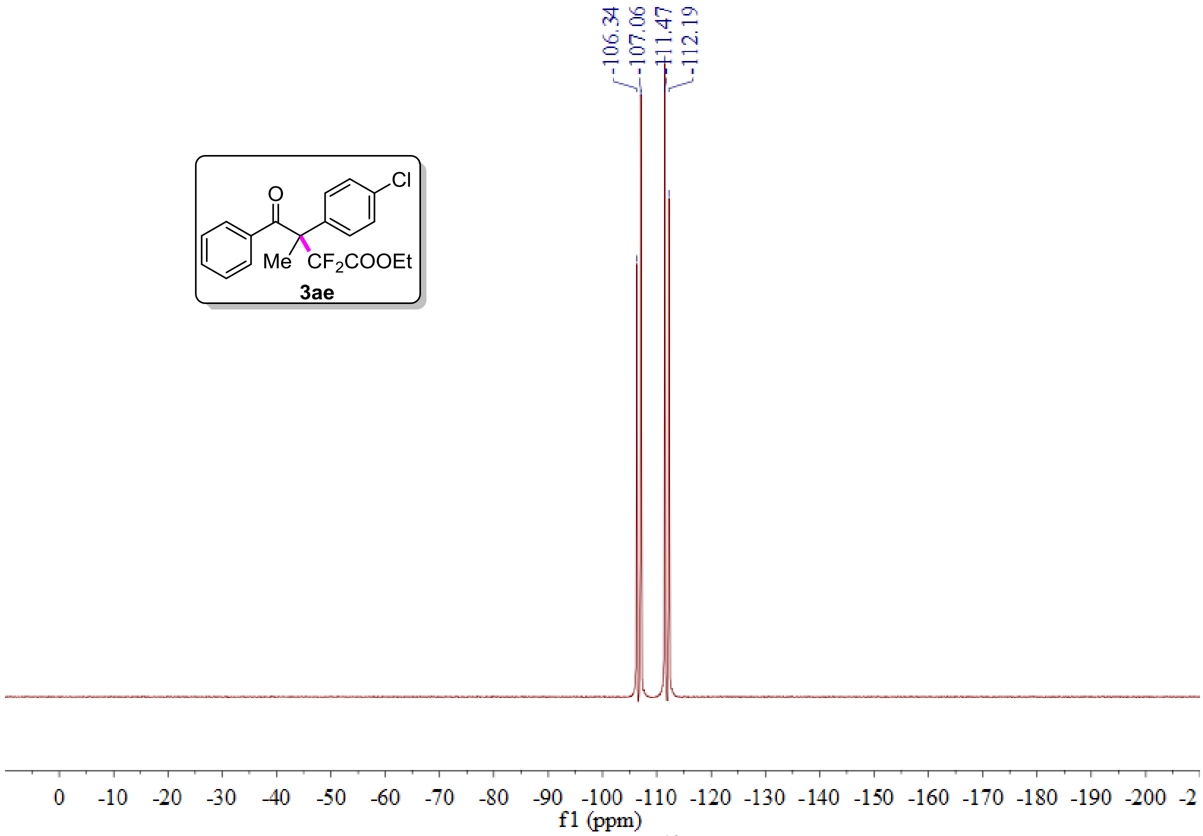

Supplementary Figure 93. $^{19}F$ NMR of 3ae

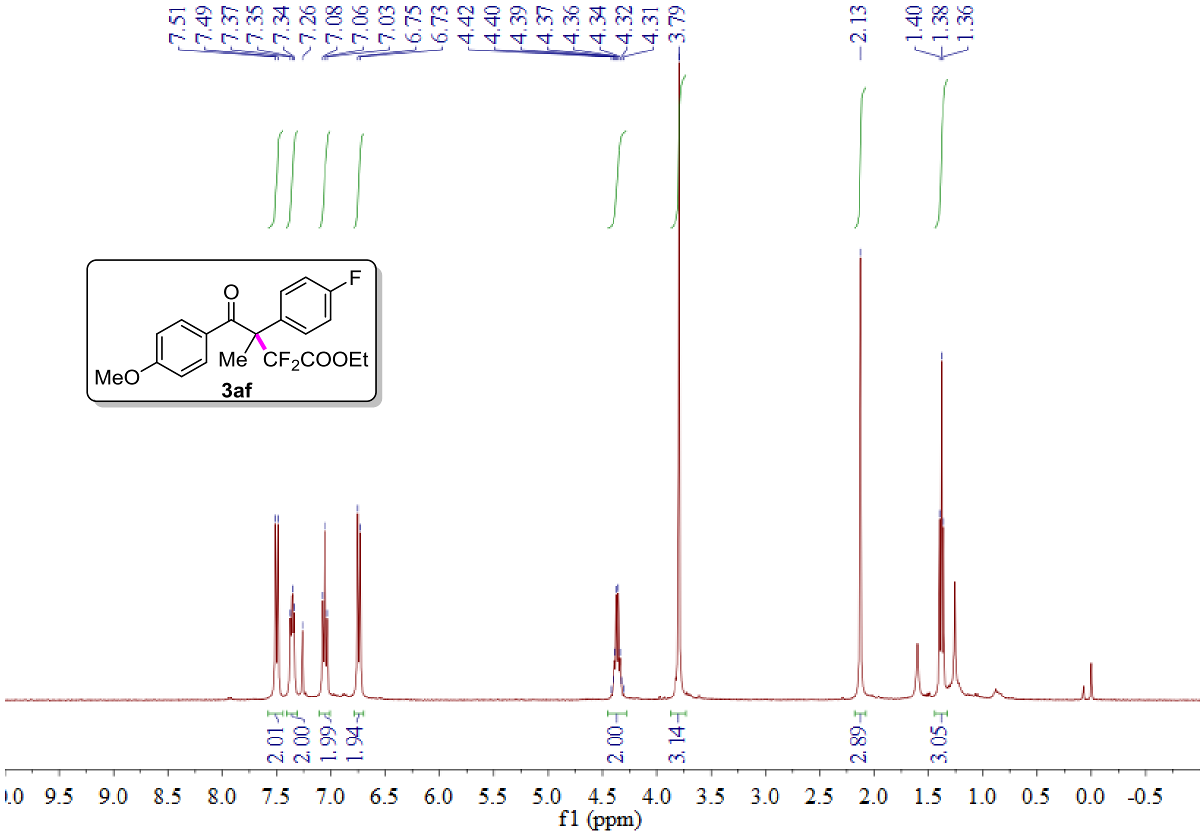

Supplementary Figure 94. $^{1}H$ NMR of 3af

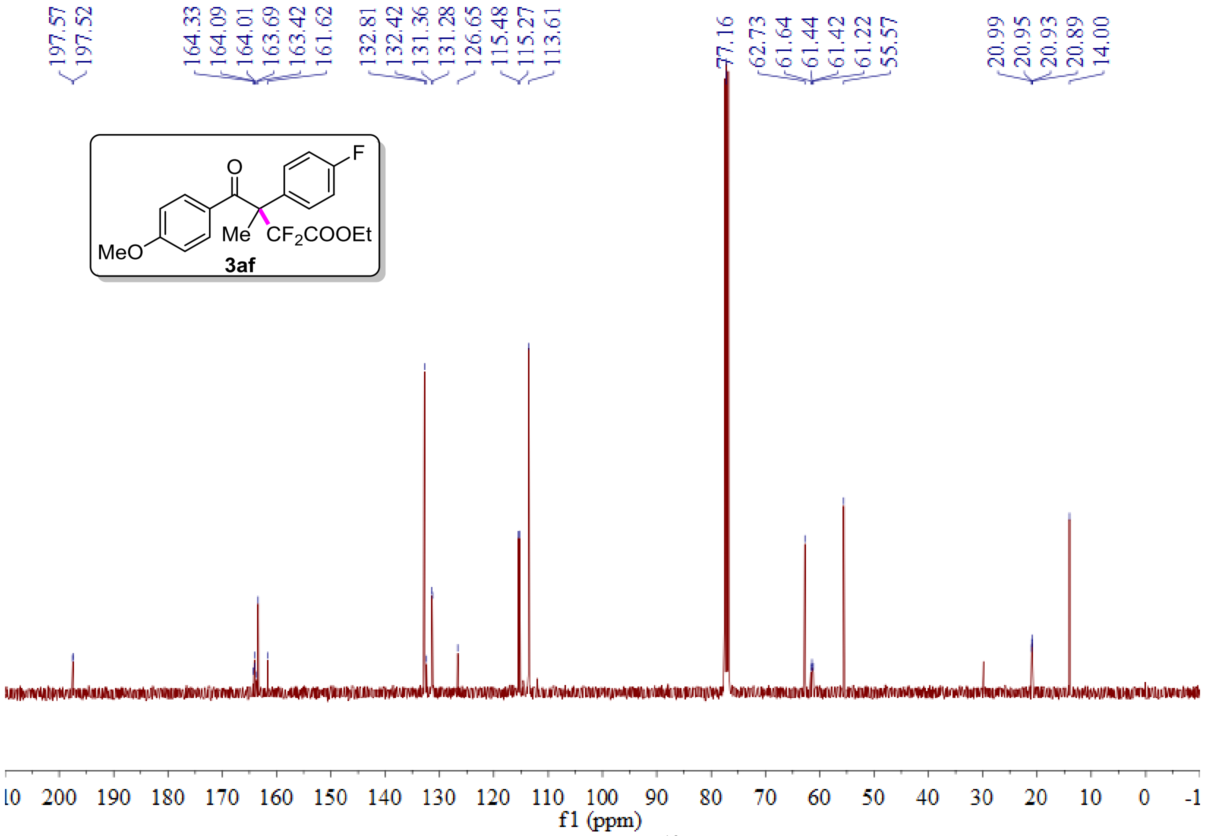


Supplementary Figure 95. $^{13}C$ NMR of 3af

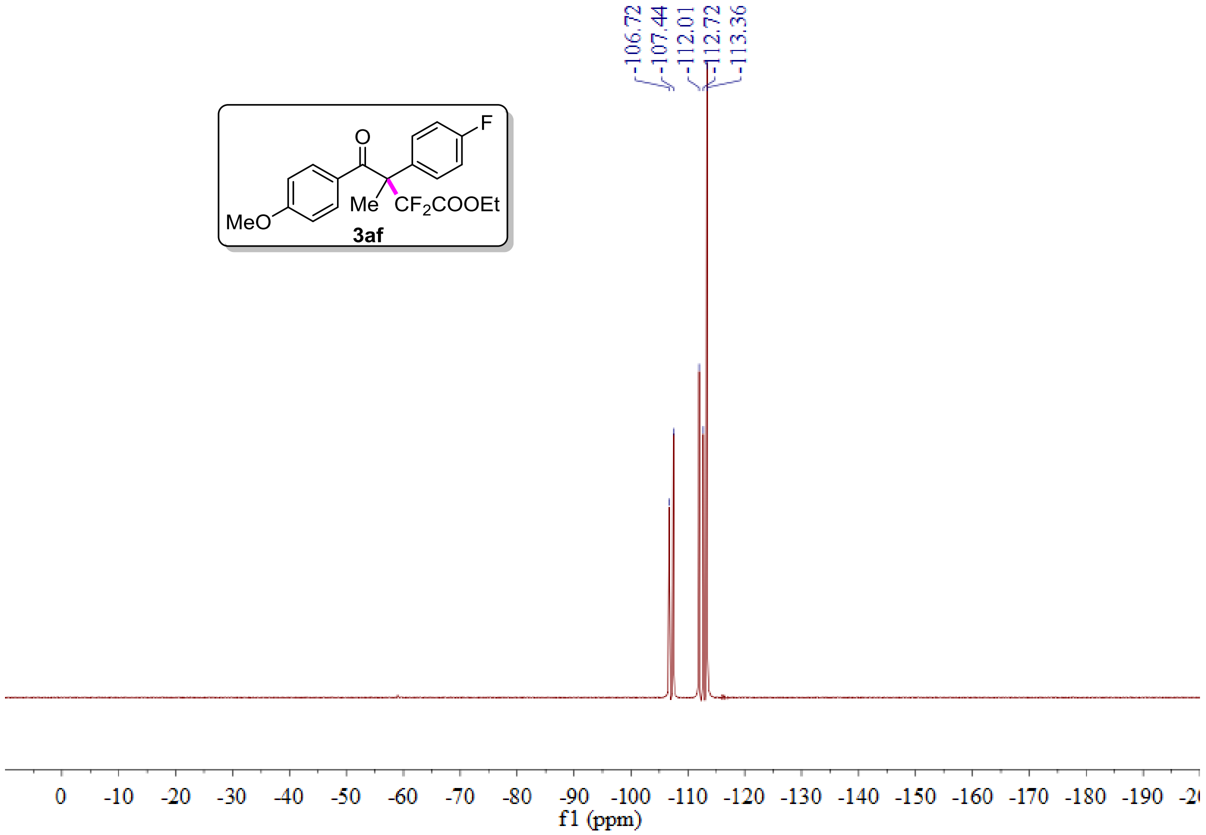


Supplementary Figure 96. $^{19}F$ NMR of 3af

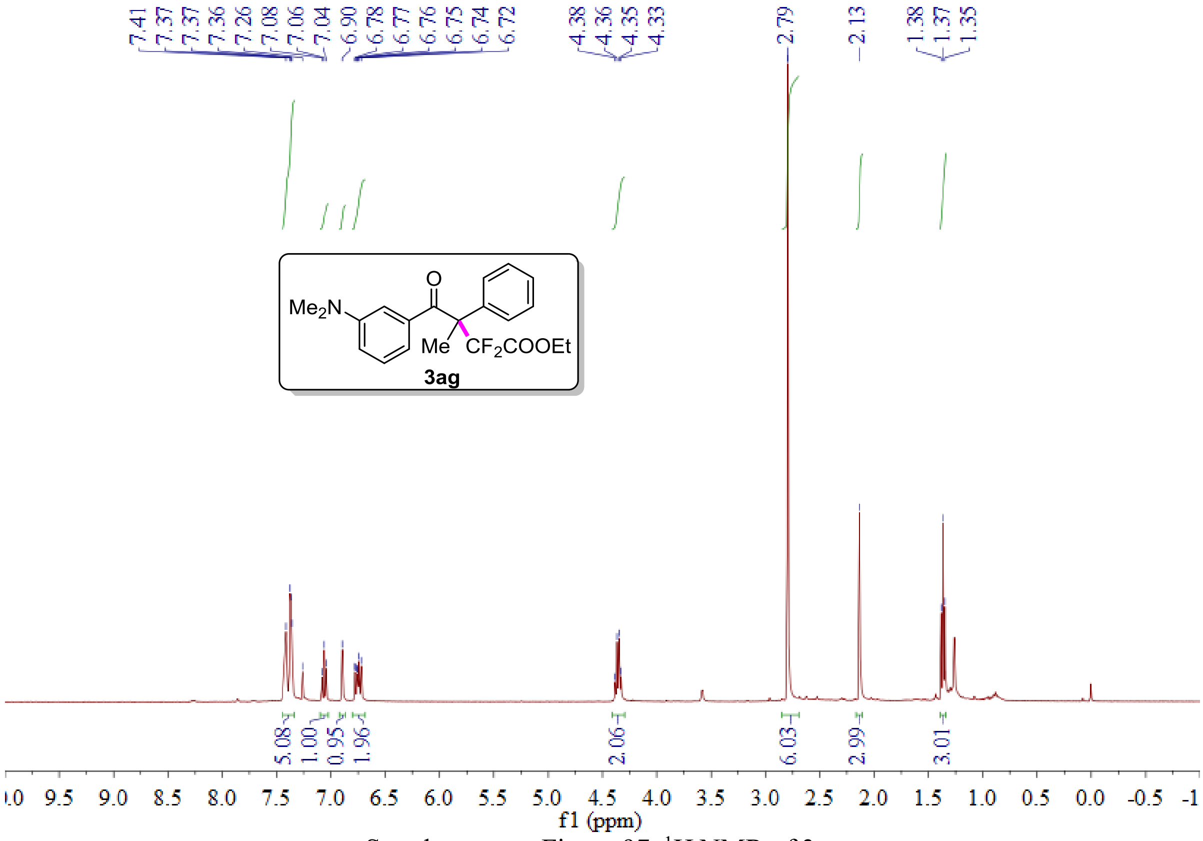

Supplementary Figure 97. $^{1}H$ NMR of 3ag

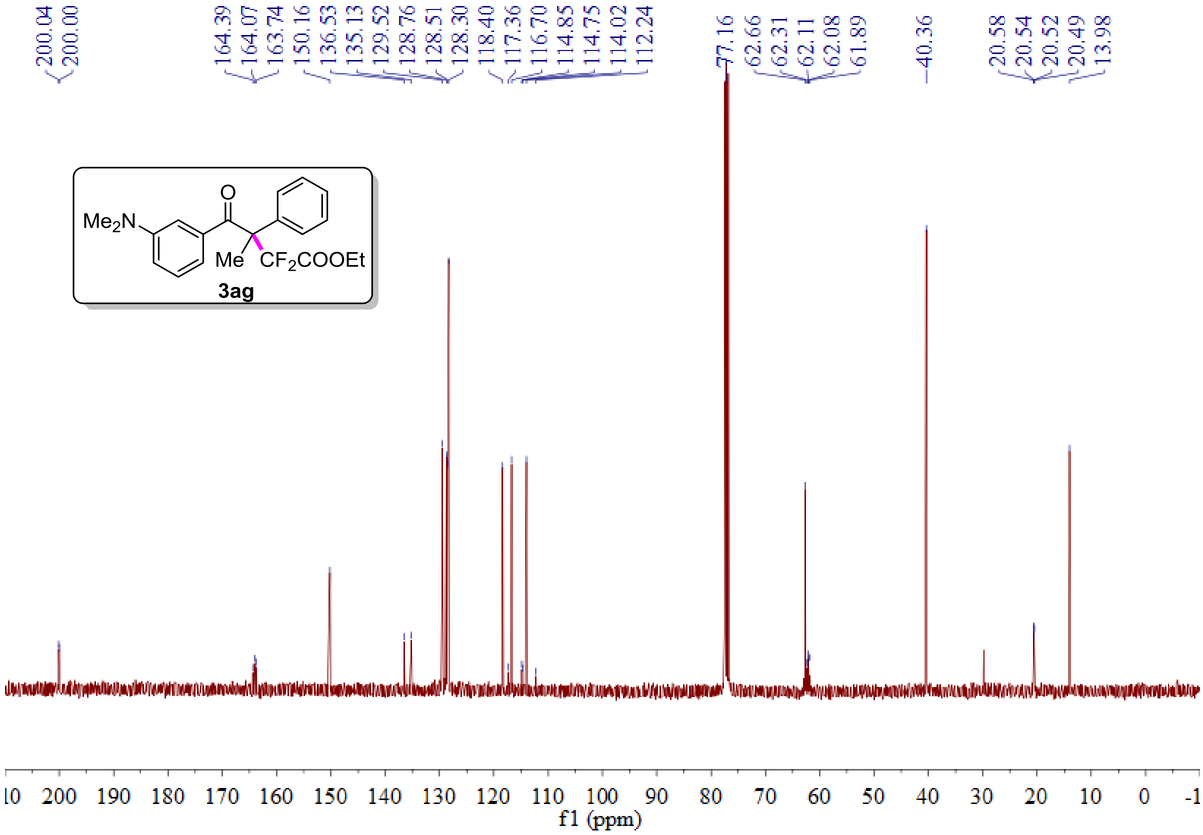

Supplementary Figure 98. $^{13}C$ NMR of 3ag

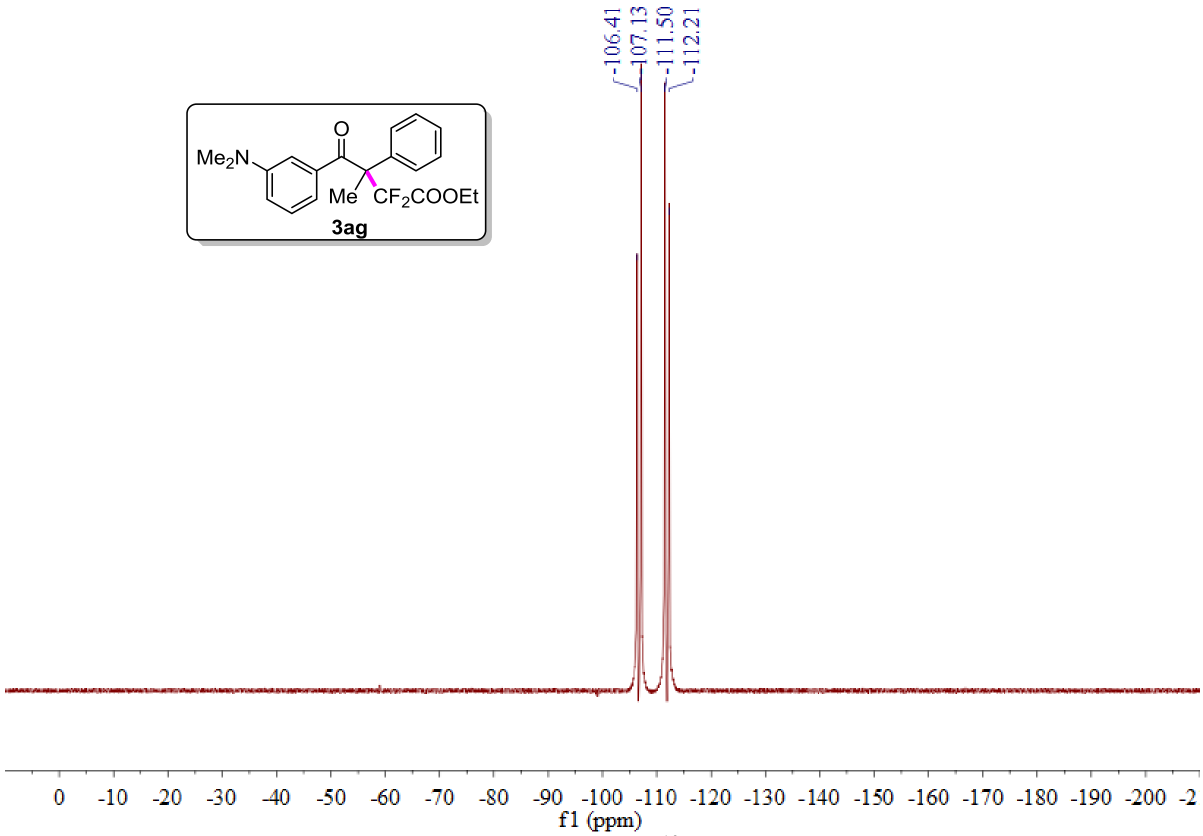

Supplementary Figure 99. $^{19}F$ NMR of 3ag

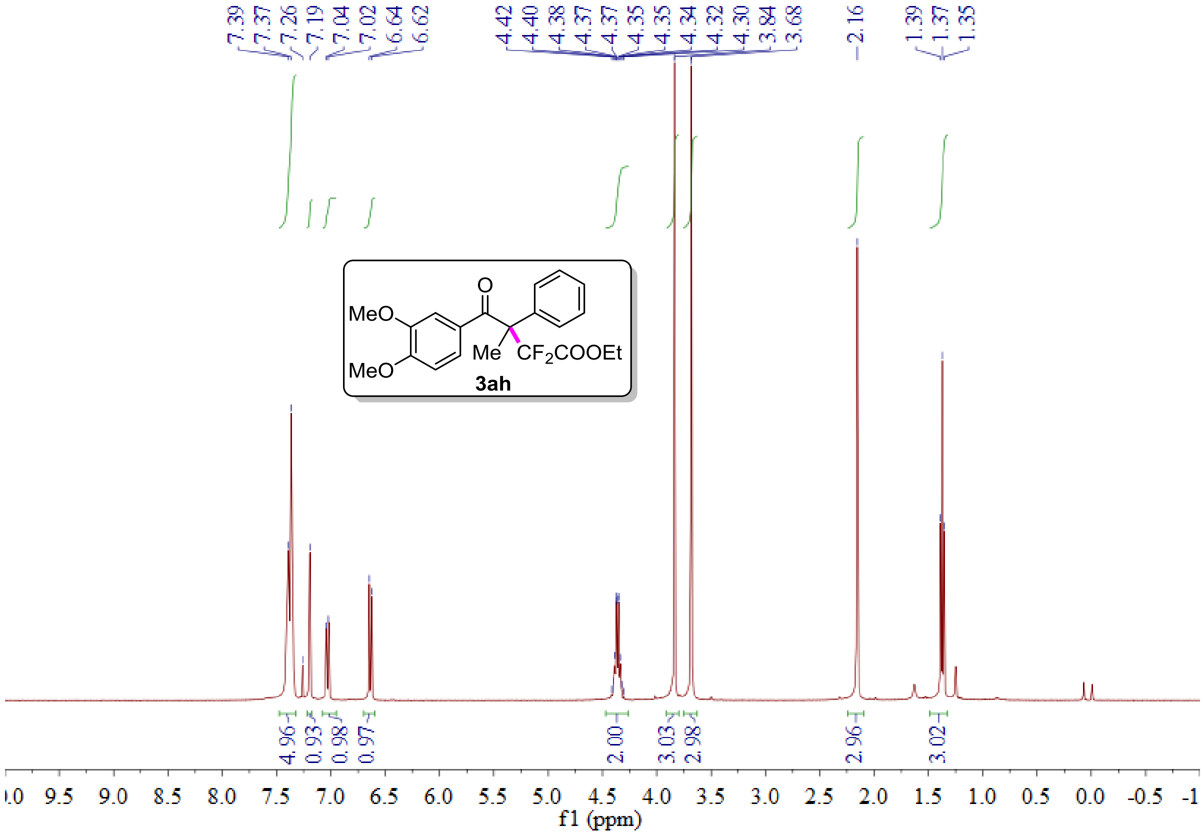

Supplementary Figure 100. $^{1}H$ NMR of 3ah

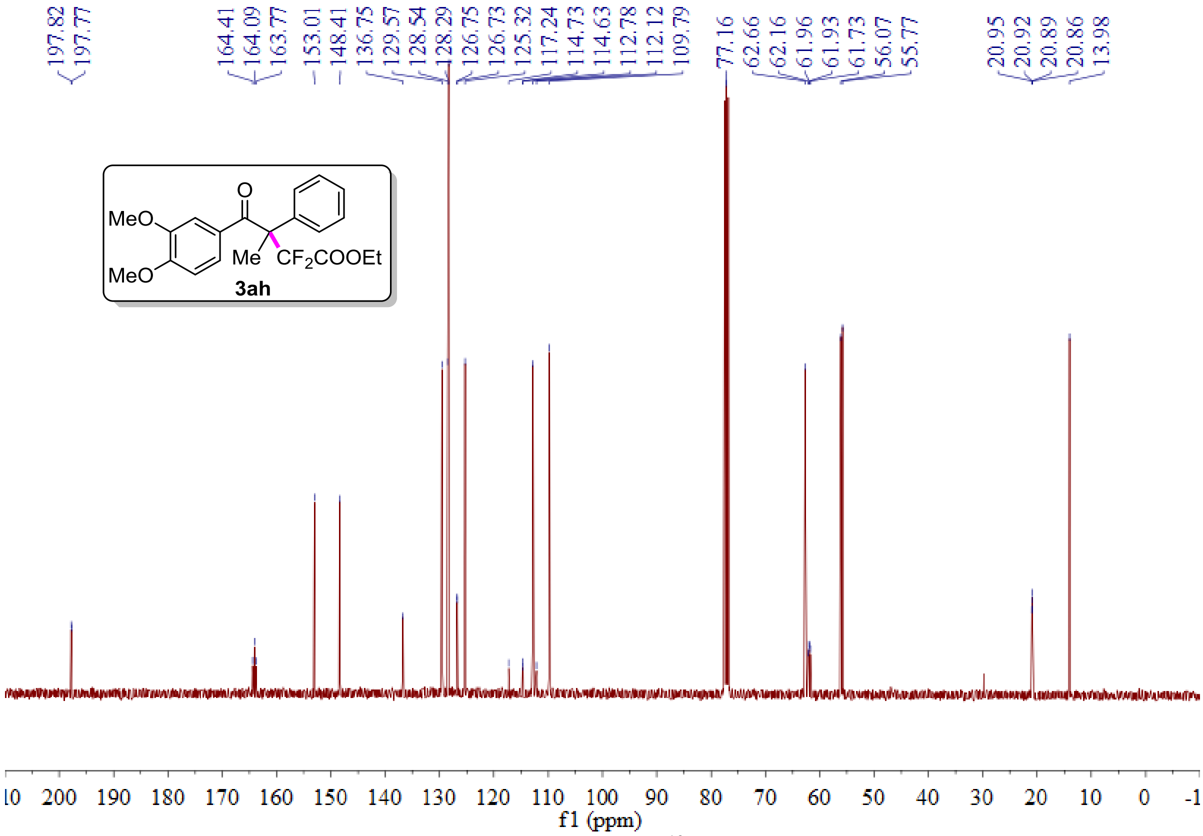


Supplementary Figure 101. $^{13}$C NMR of 3ah

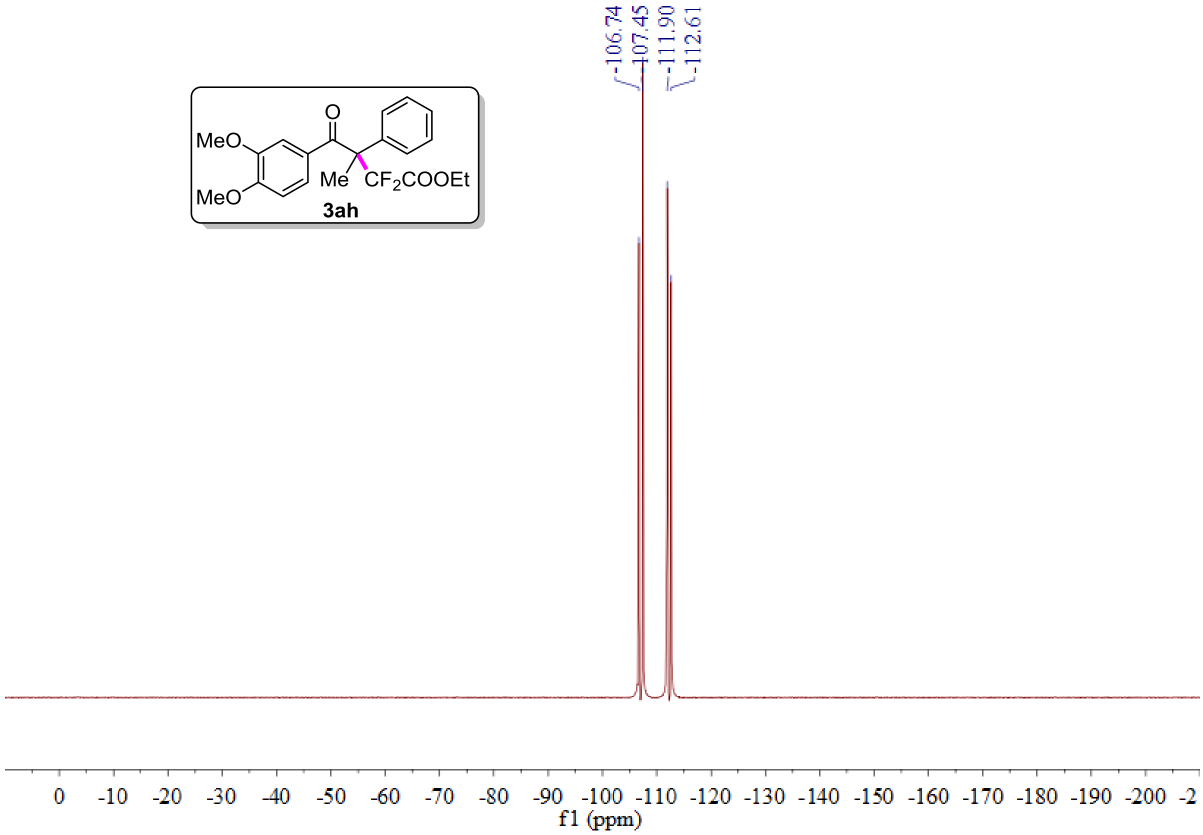


Supplementary Figure 102. $^{19}$F NMR of 3ah

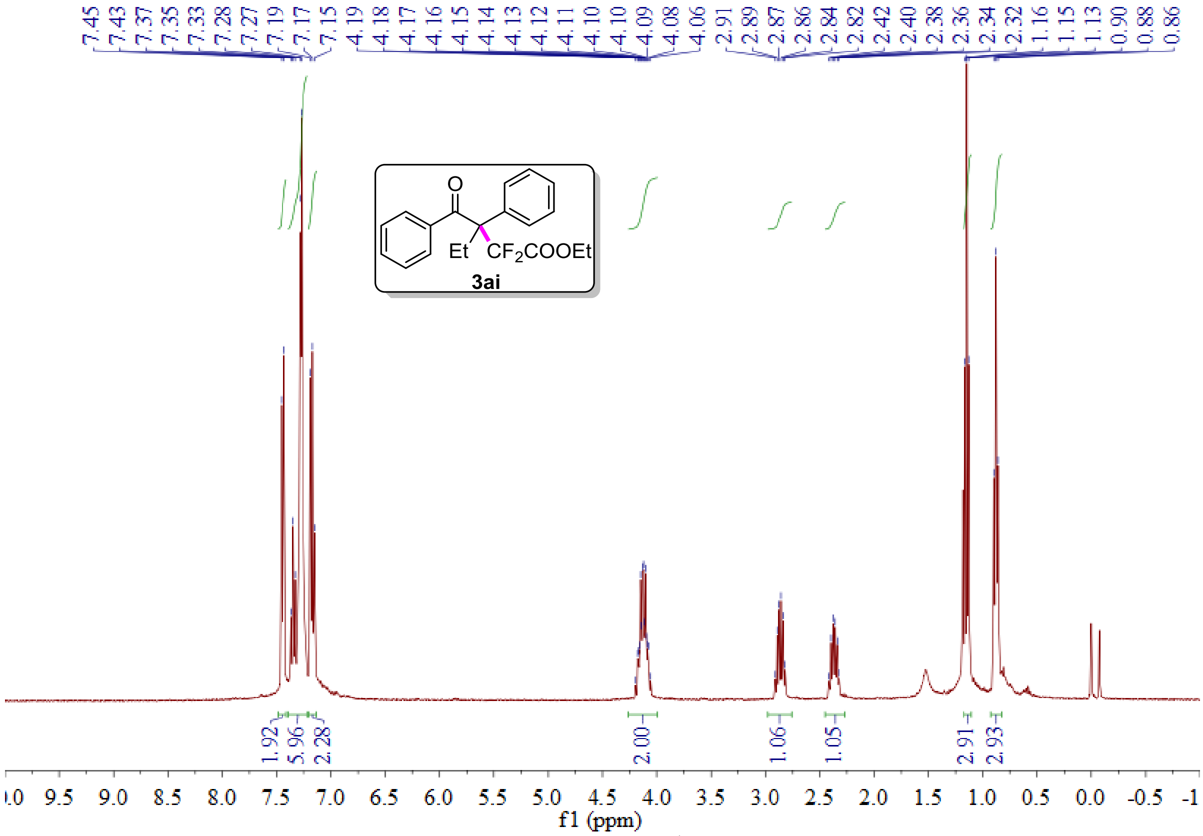

Supplementary Figure 103. $^{1}$H NMR of 3ai

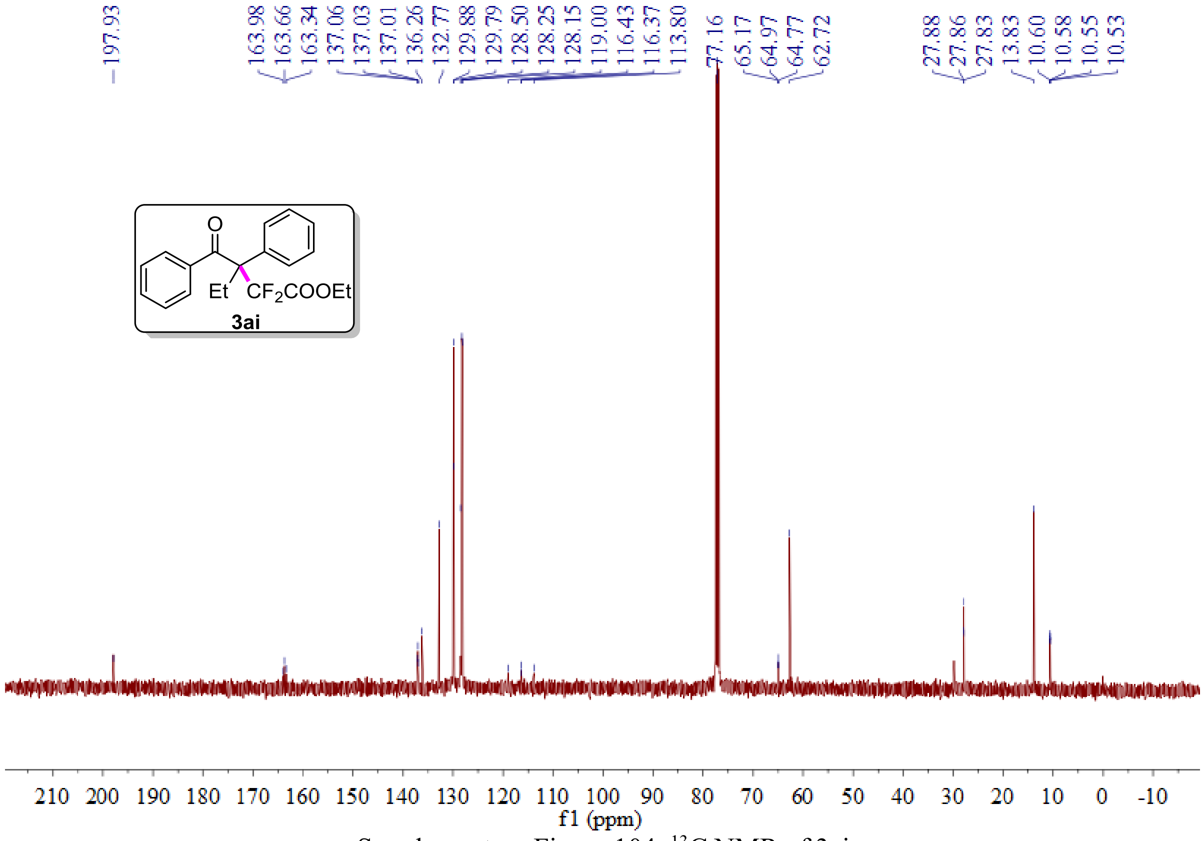

Supplementary Figure 104. $^{13}$C NMR of 3ai

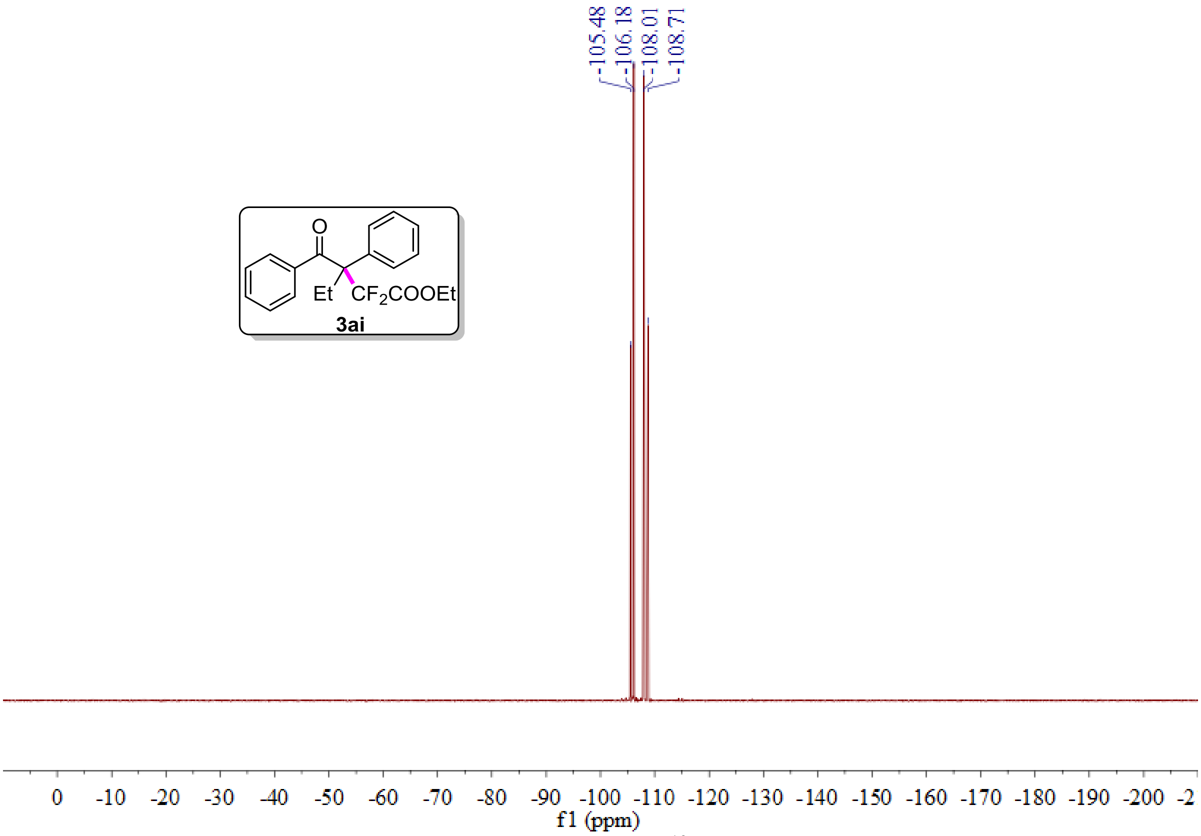


Supplementary Figure 105. $^{19}$F NMR of 3ai

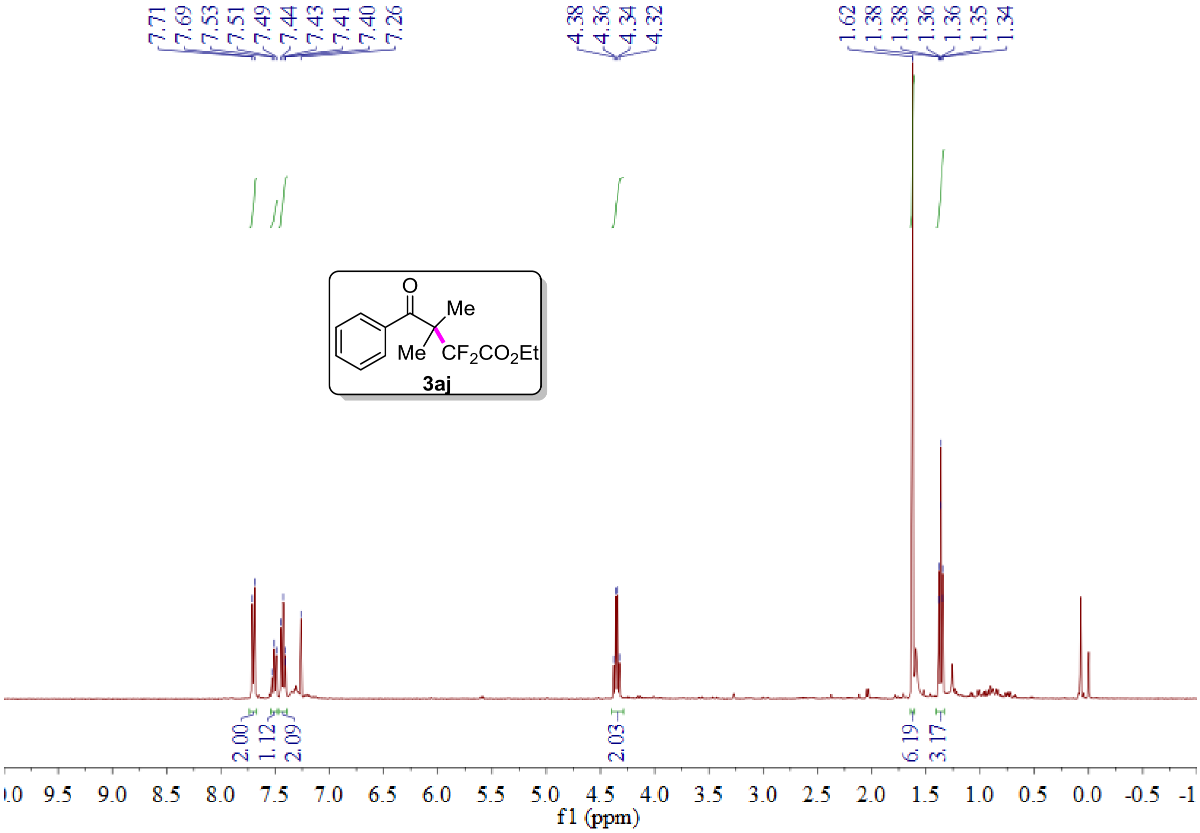


Supplementary Figure 106. $^{1}$H NMR of 3aj

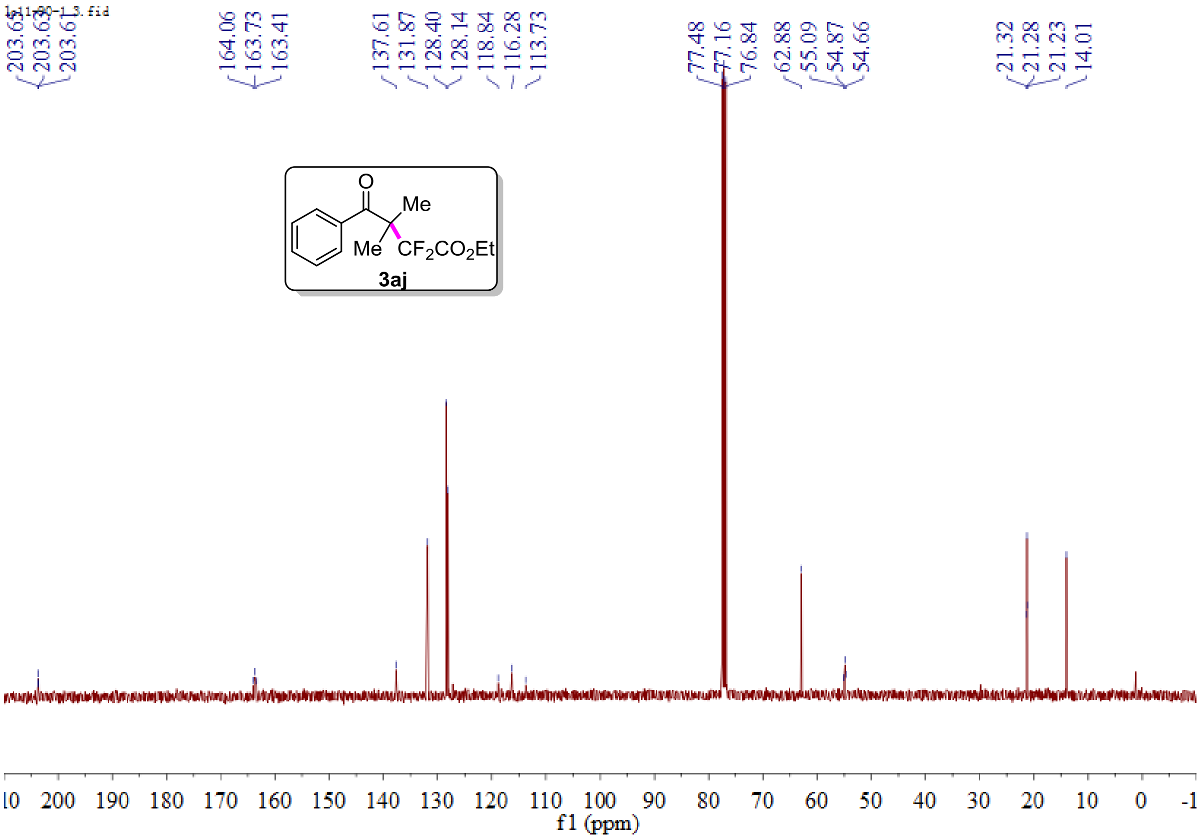


Supplementary Figure 107. $^{13}C$ NMR of 3aj

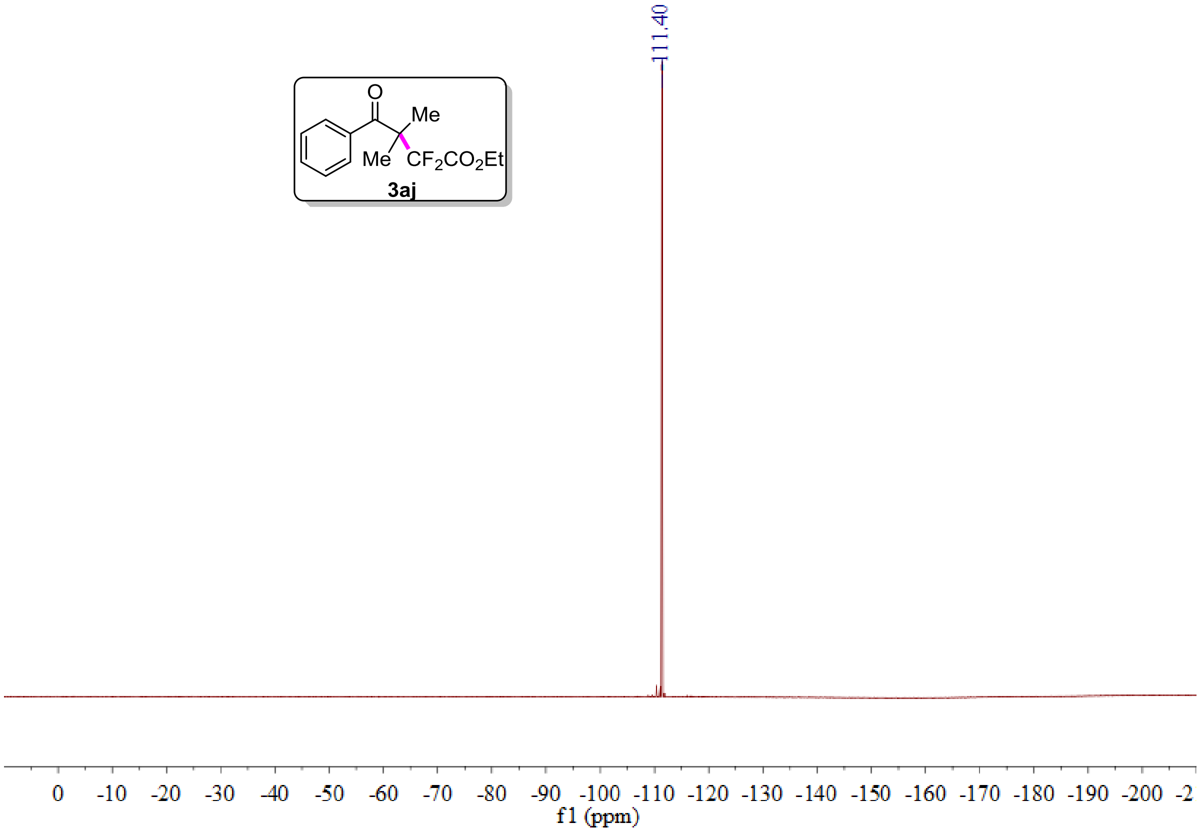


Supplementary Figure 108. $^{19}F$ NMR of 3aj

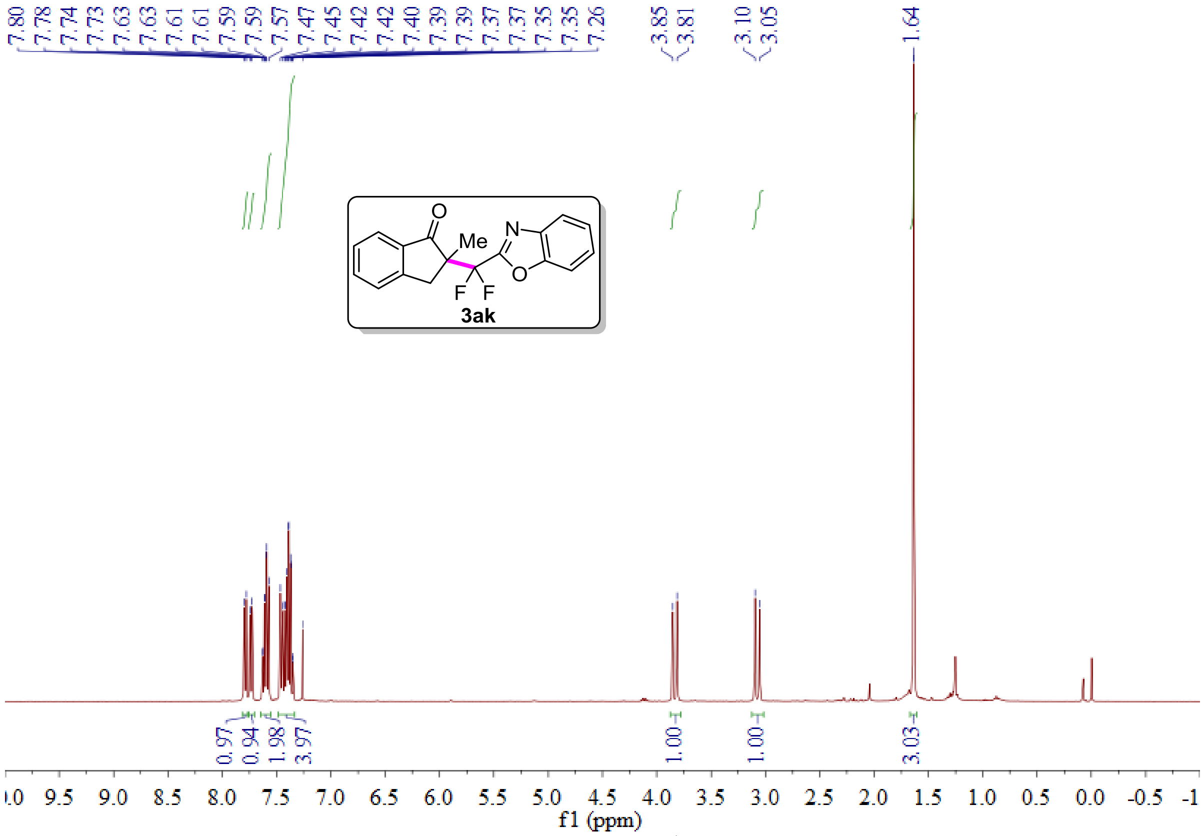


Supplementary Figure 109. $^{1}$H NMR of 3ak

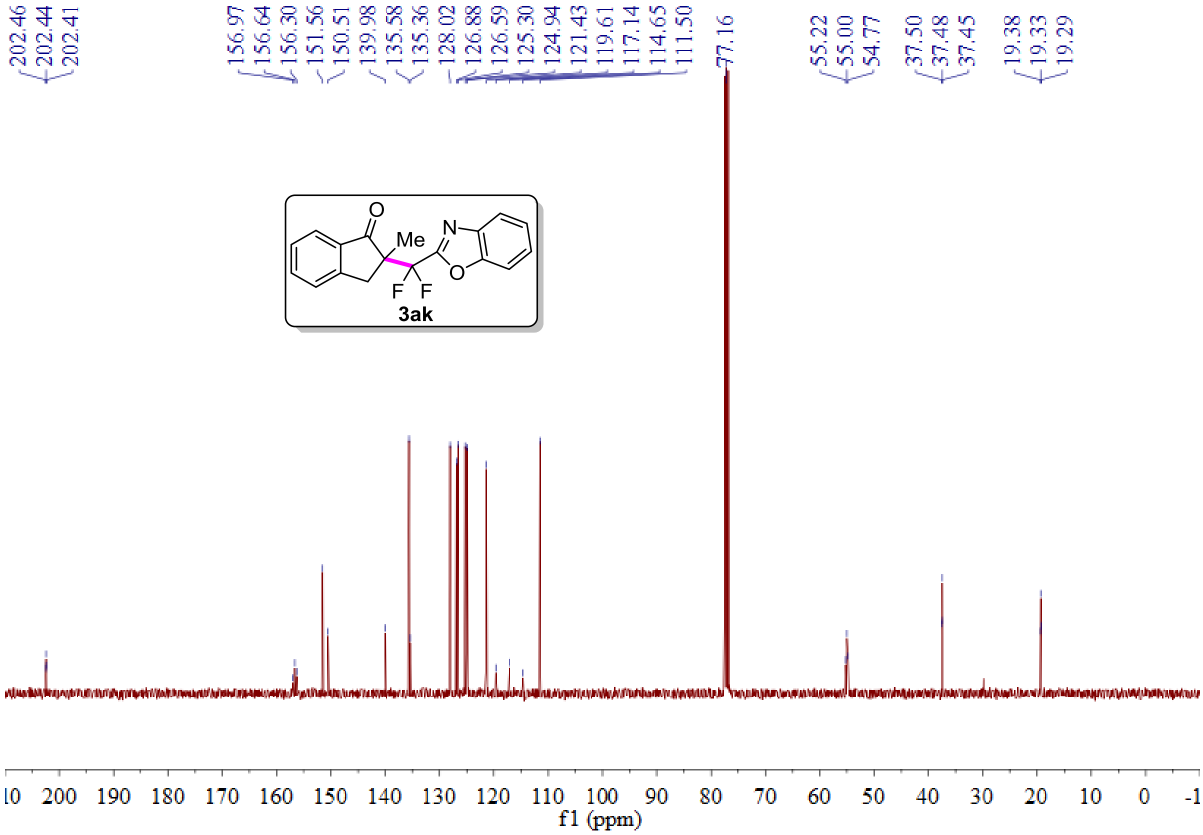


Supplementary Figure 110. $^{13}$C NMR of 3ak

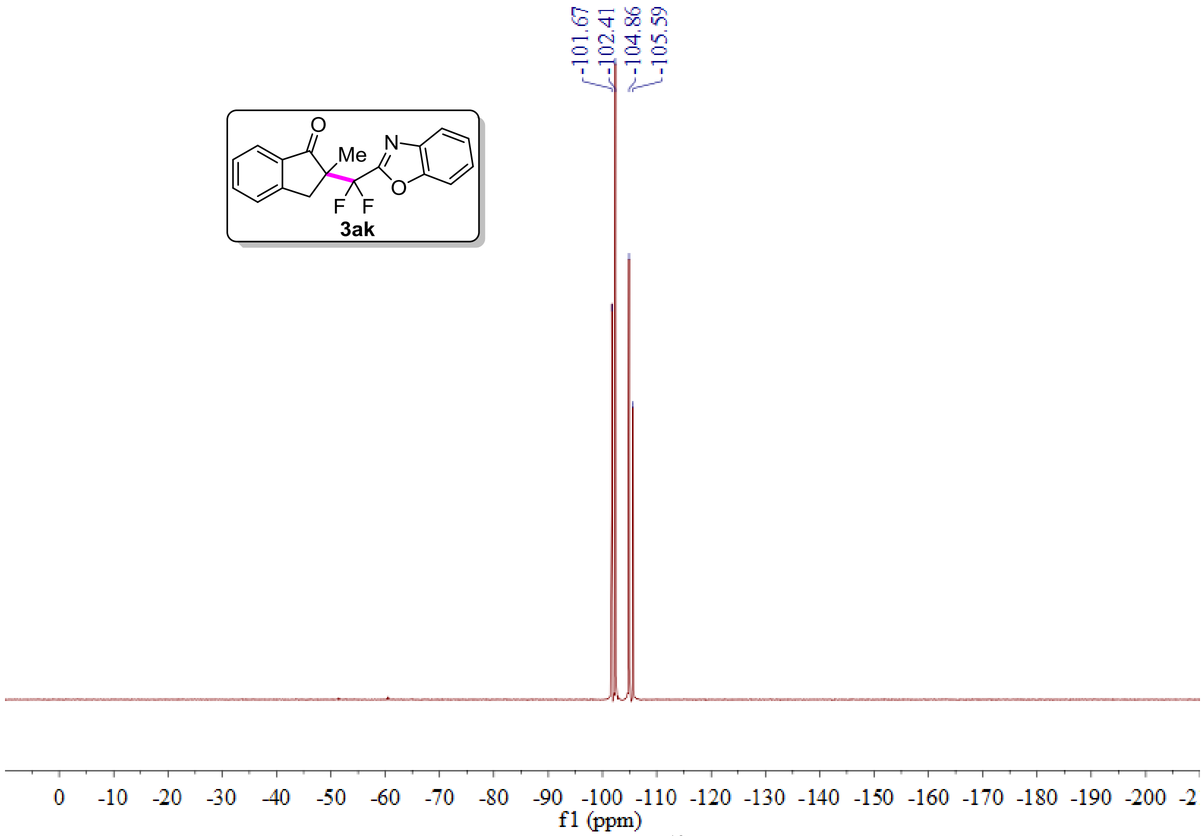


Supplementary Figure 111. $^{19}F$ NMR of 3ak

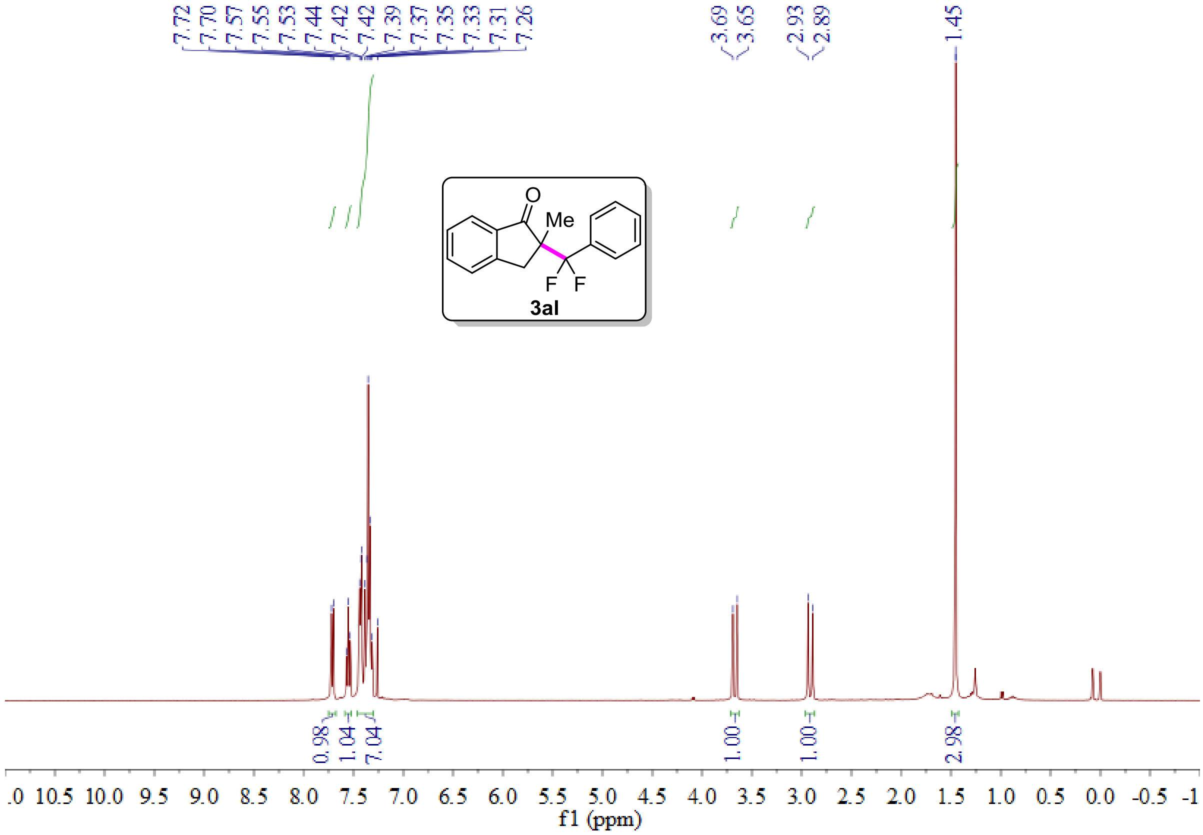


Supplementary Figure 112. $^{1}H$ NMR of 3al

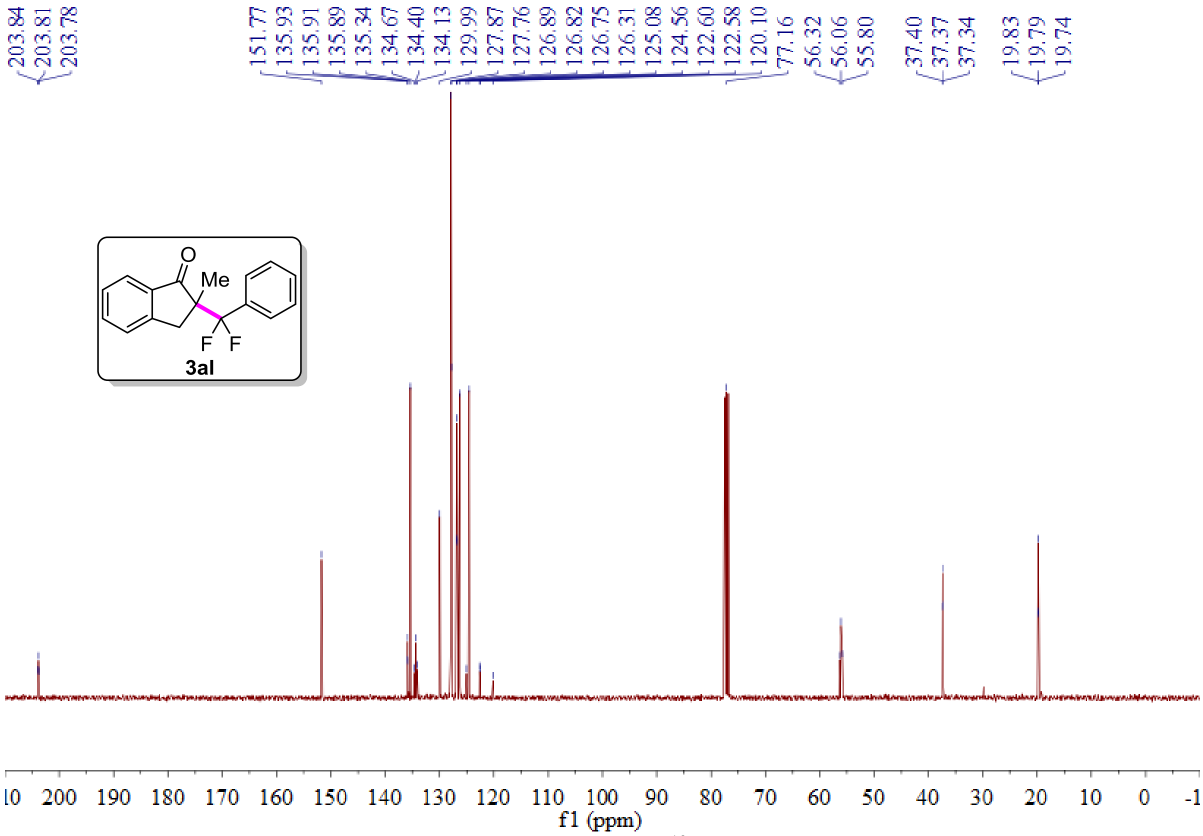


Supplementary Figure 113. $^{13}C$ NMR of 3al

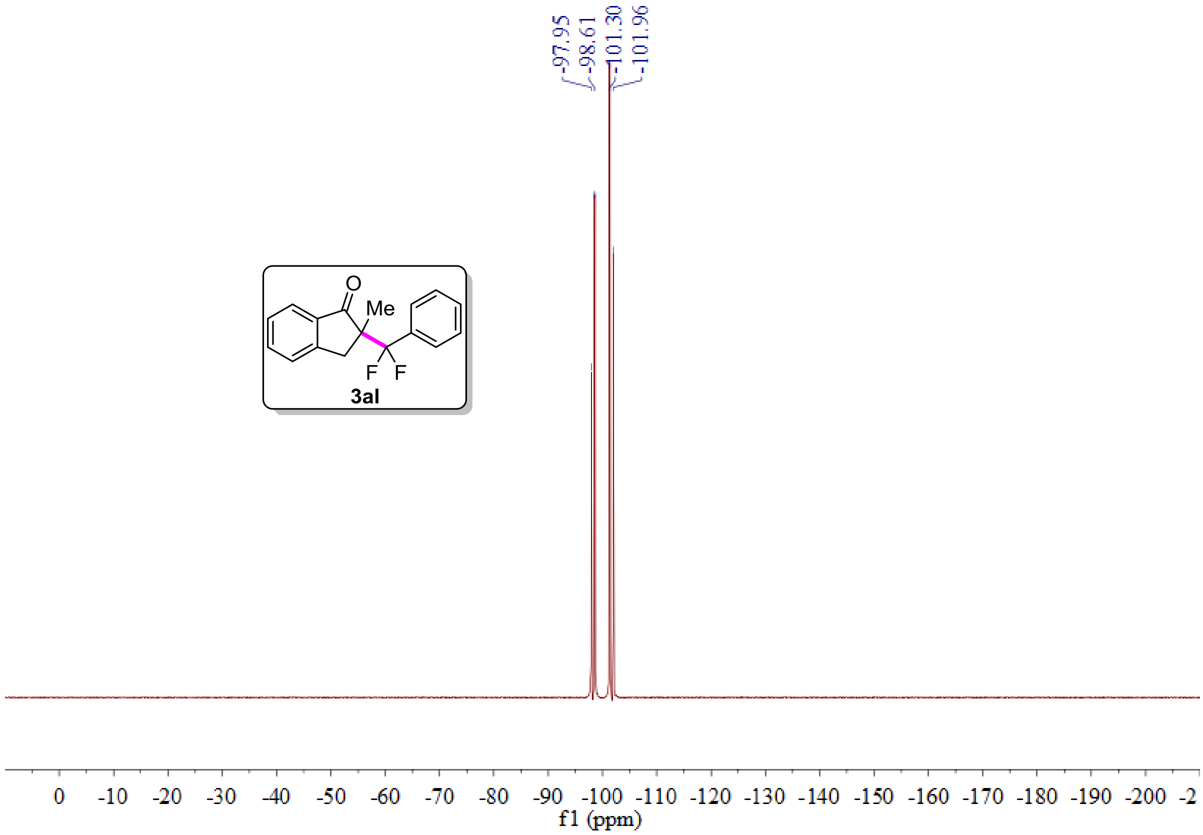


Supplementary Figure 114. $^{19}F$ NMR of 3al

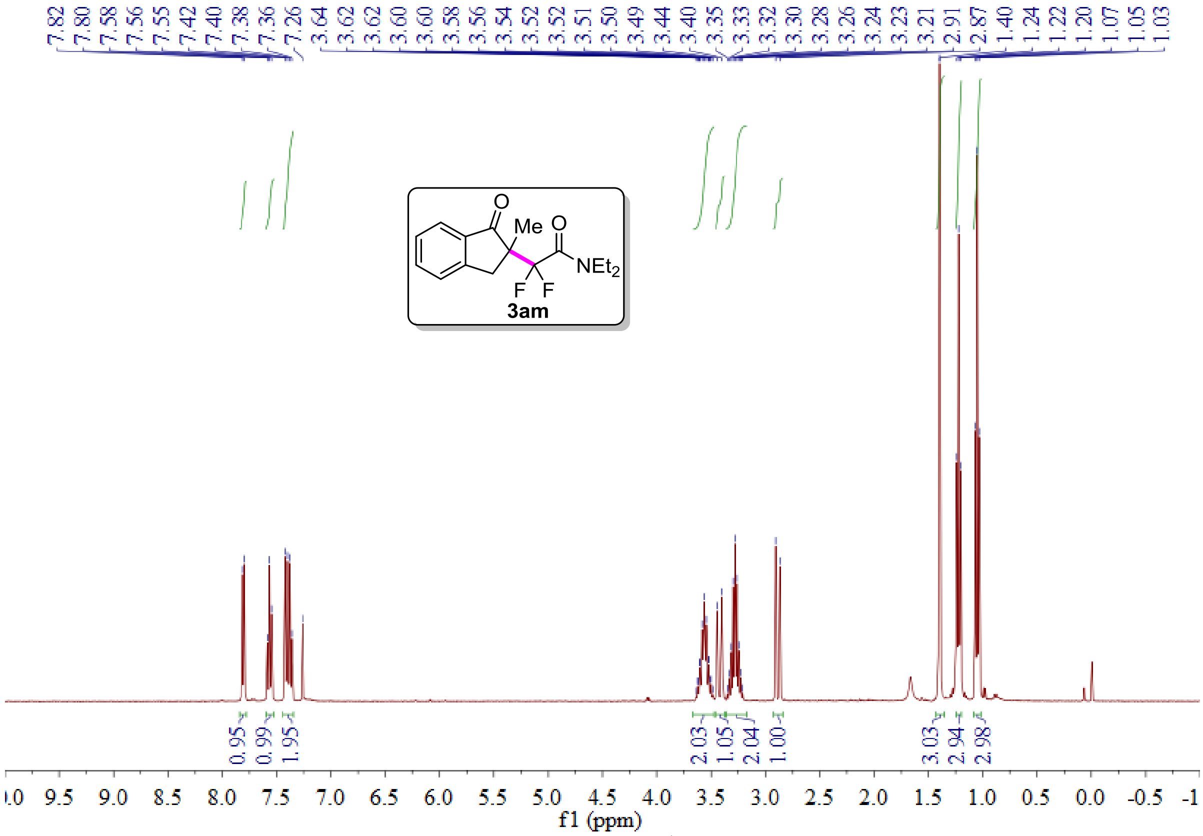

Supplementary Figure 115. $^{1}$H NMR of 3am

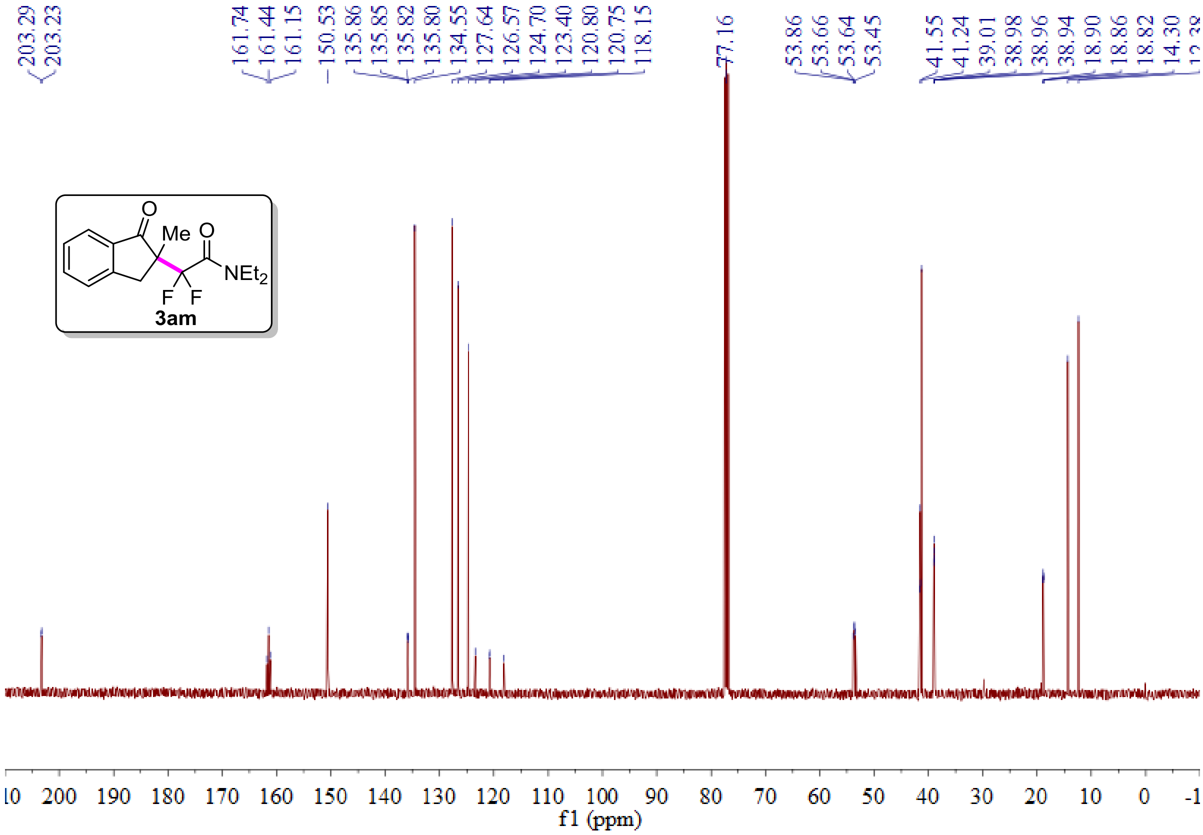

Supplementary Figure 116. $^{13}$C NMR of 3am

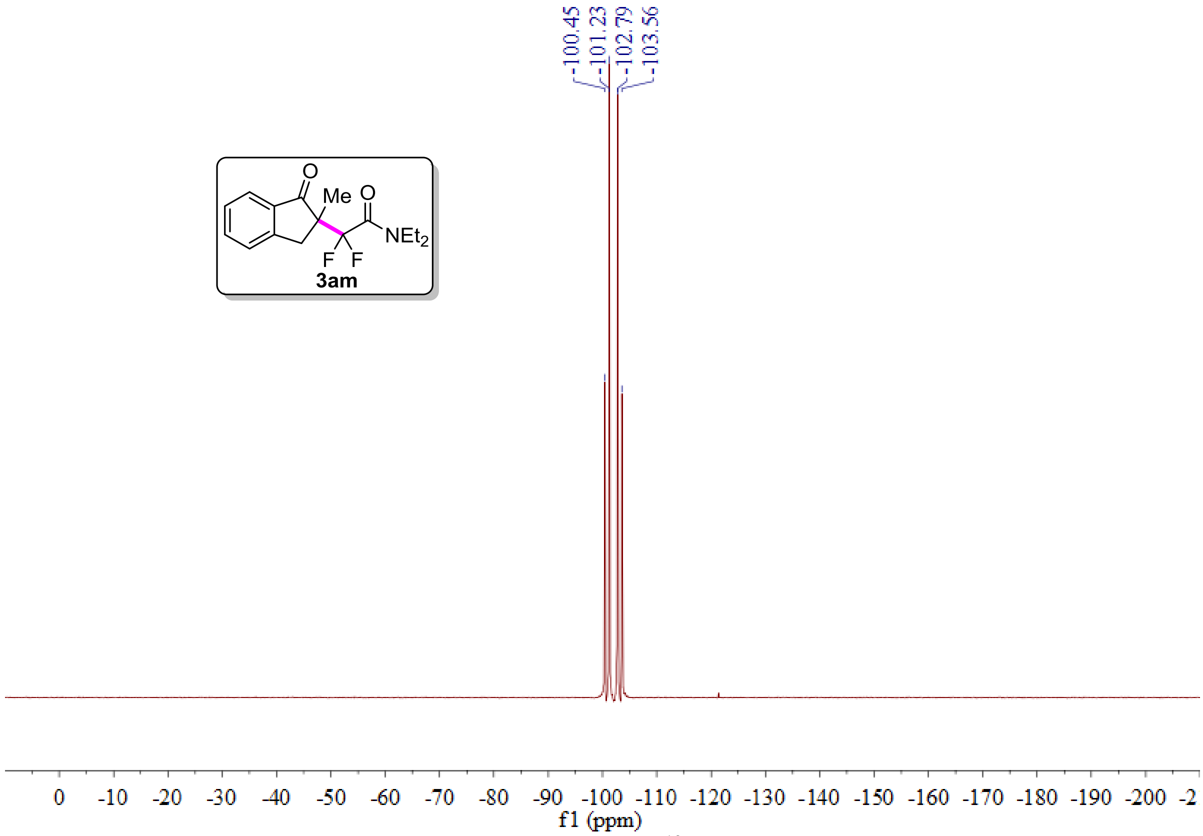


Supplementary Figure 117. $^{19}$F NMR of 3am

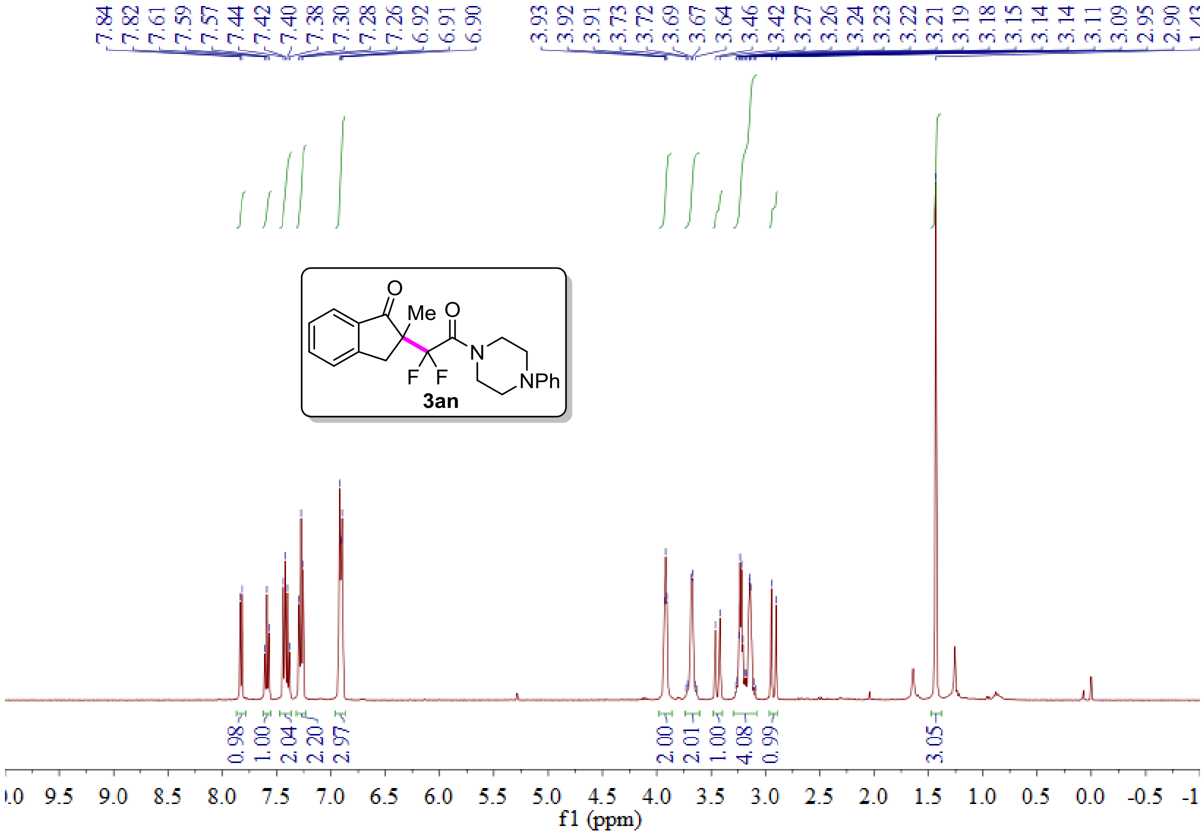


Supplementary Figure 118. $^{1}$H NMR of 3an

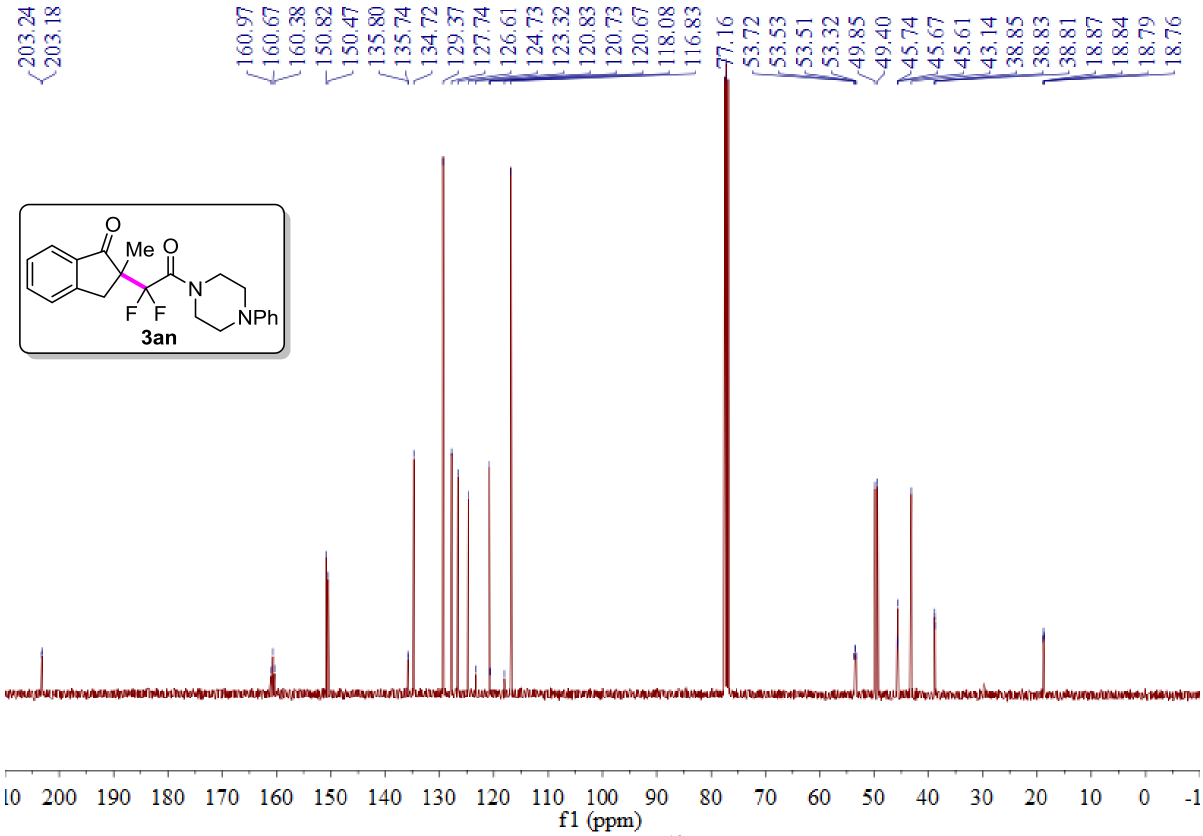


Supplementary Figure 119. $^{13}$C NMR of 3an

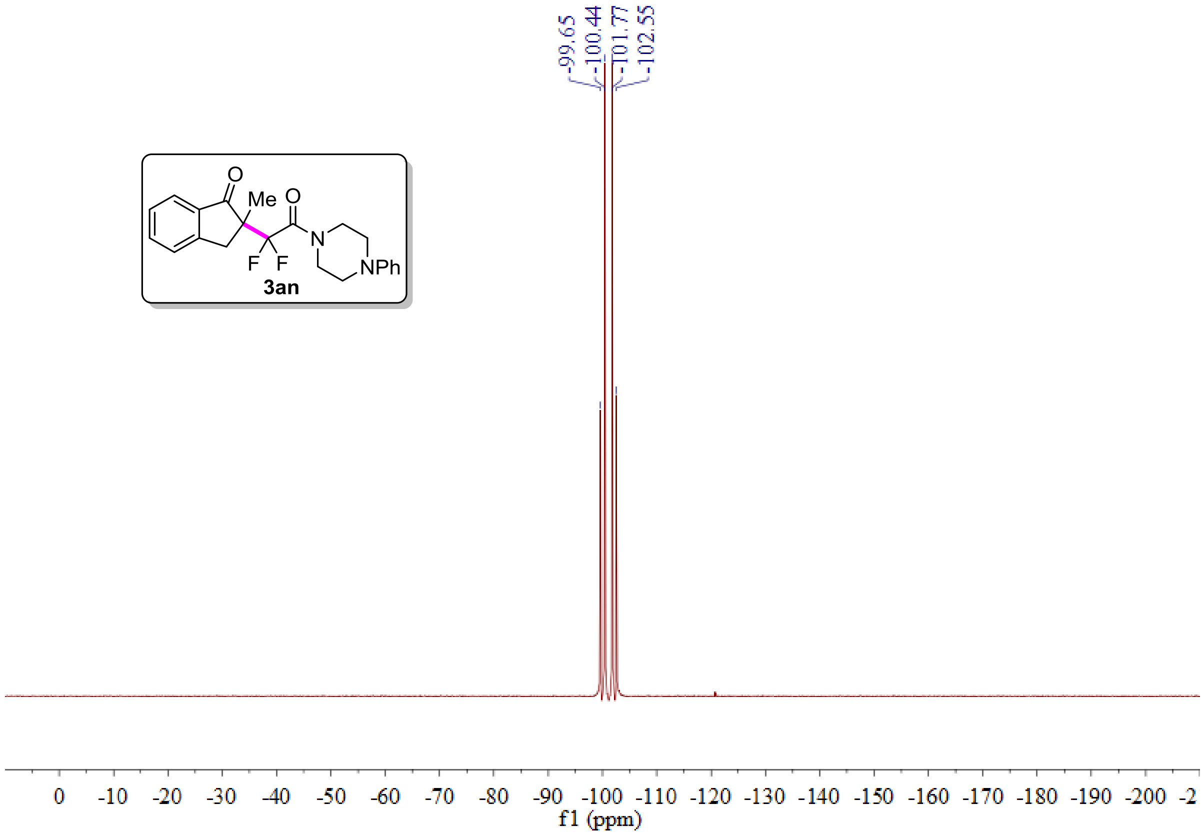


Supplementary Figure 120. $^{19}$F NMR of 3an

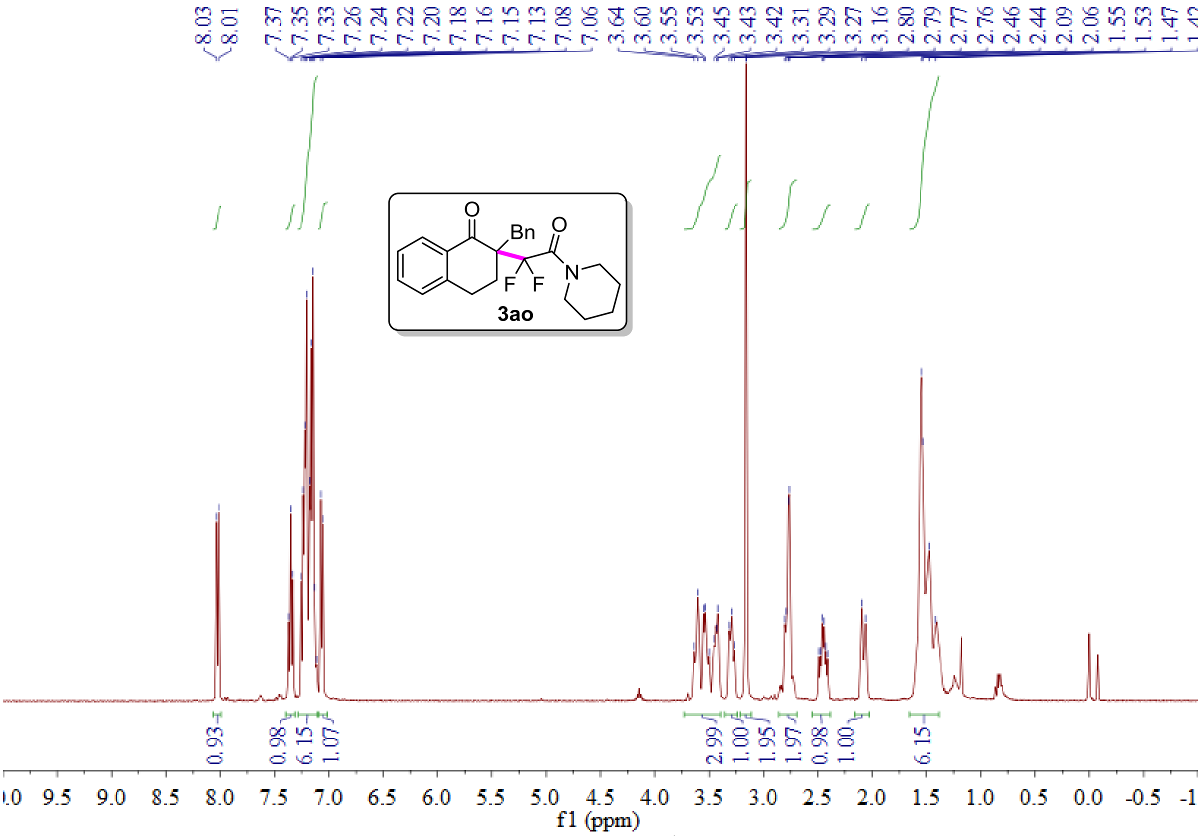


Supplementary Figure 121. $^{1}$H NMR of 3ao

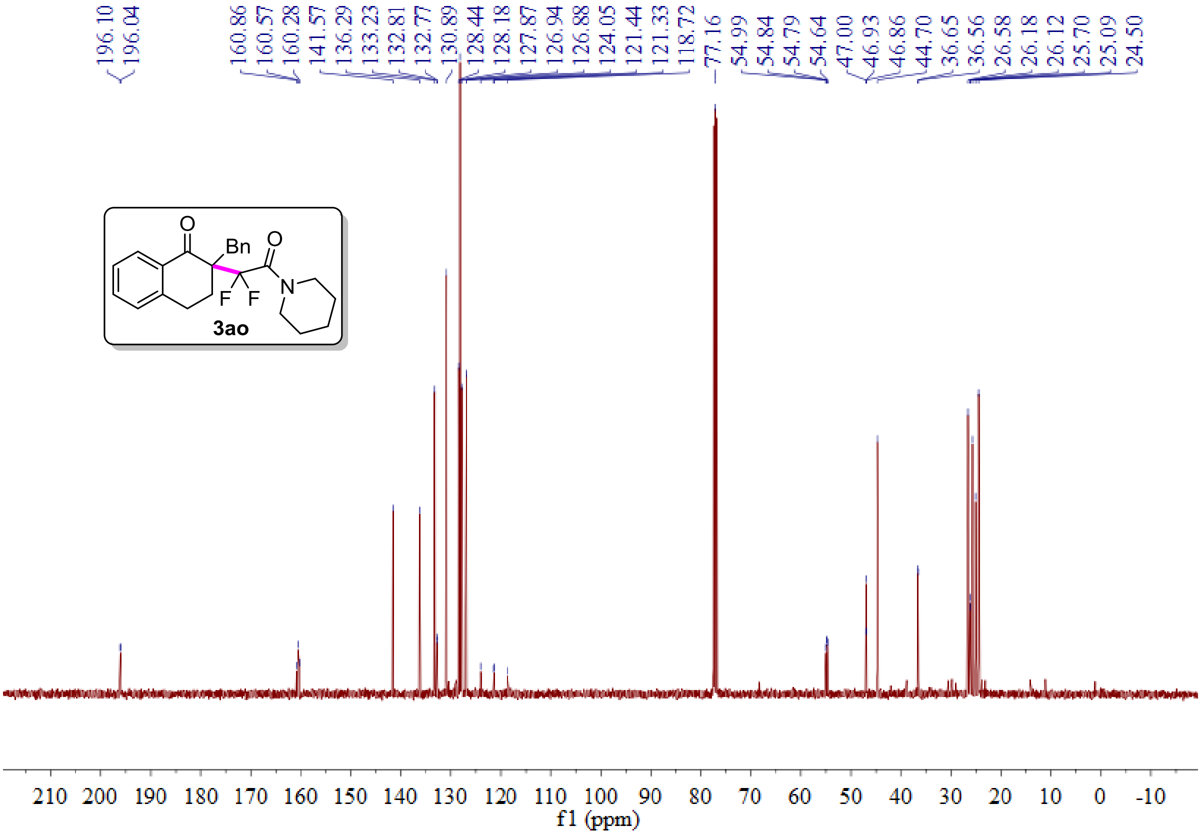


Supplementary Figure 122. $^{13}$C NMR of 3ao

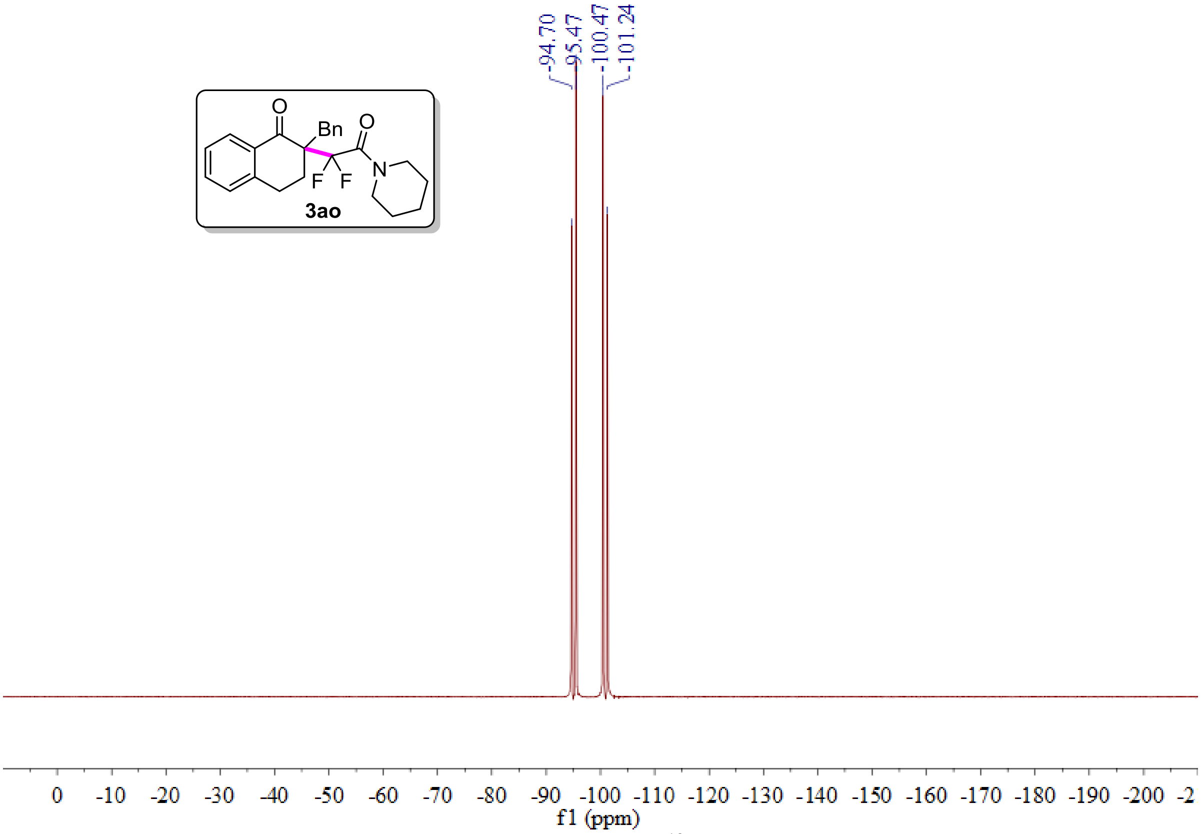


Supplementary Figure 123. $^{19}$F NMR of 3ao

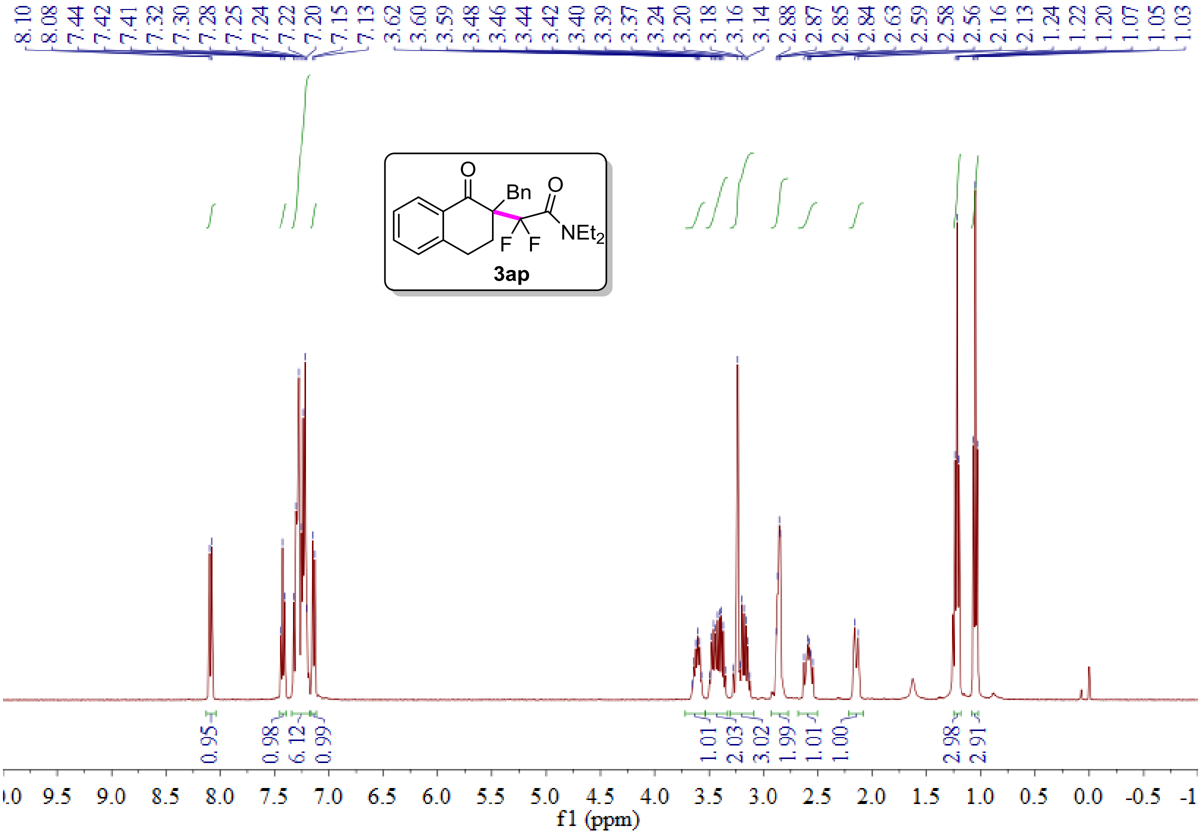


Supplementary Figure 124. $^{1}$H NMR of 3ap

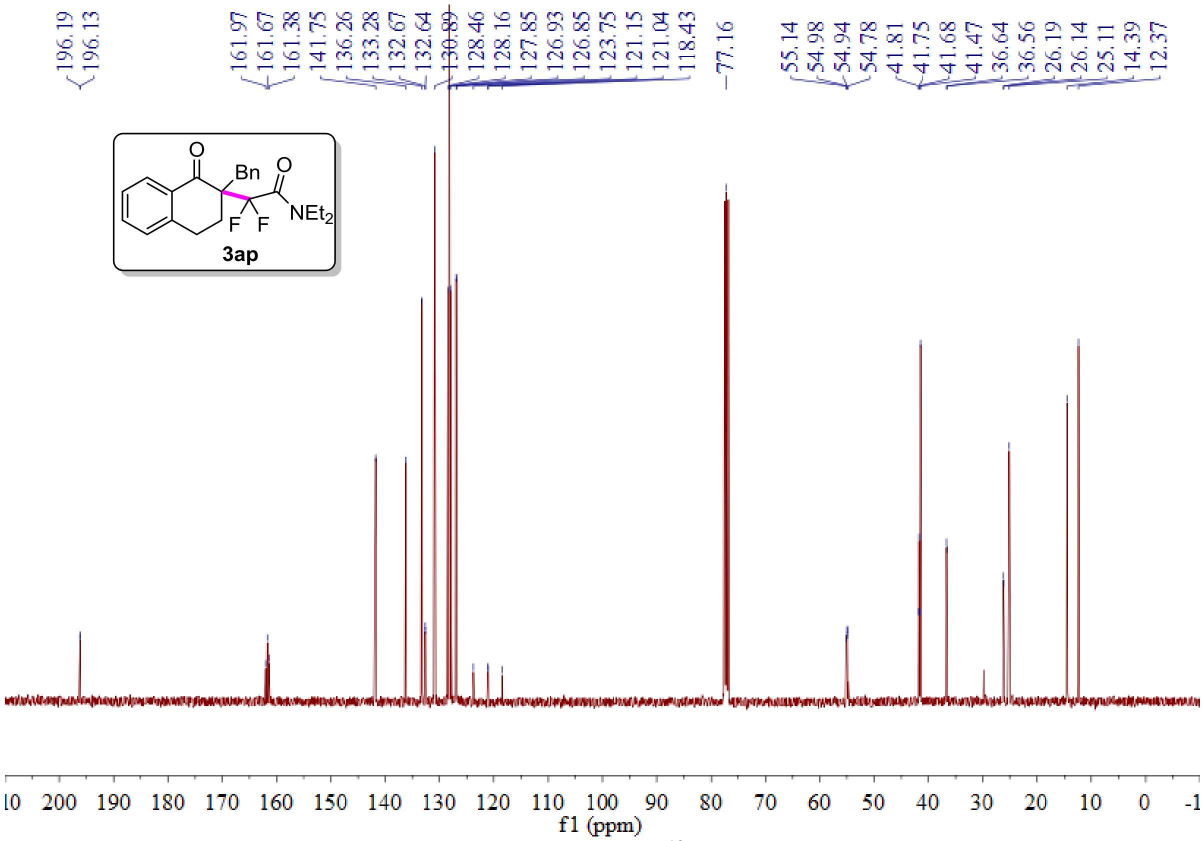


Supplementary Figure 125. $^{13}$C NMR of 3ap

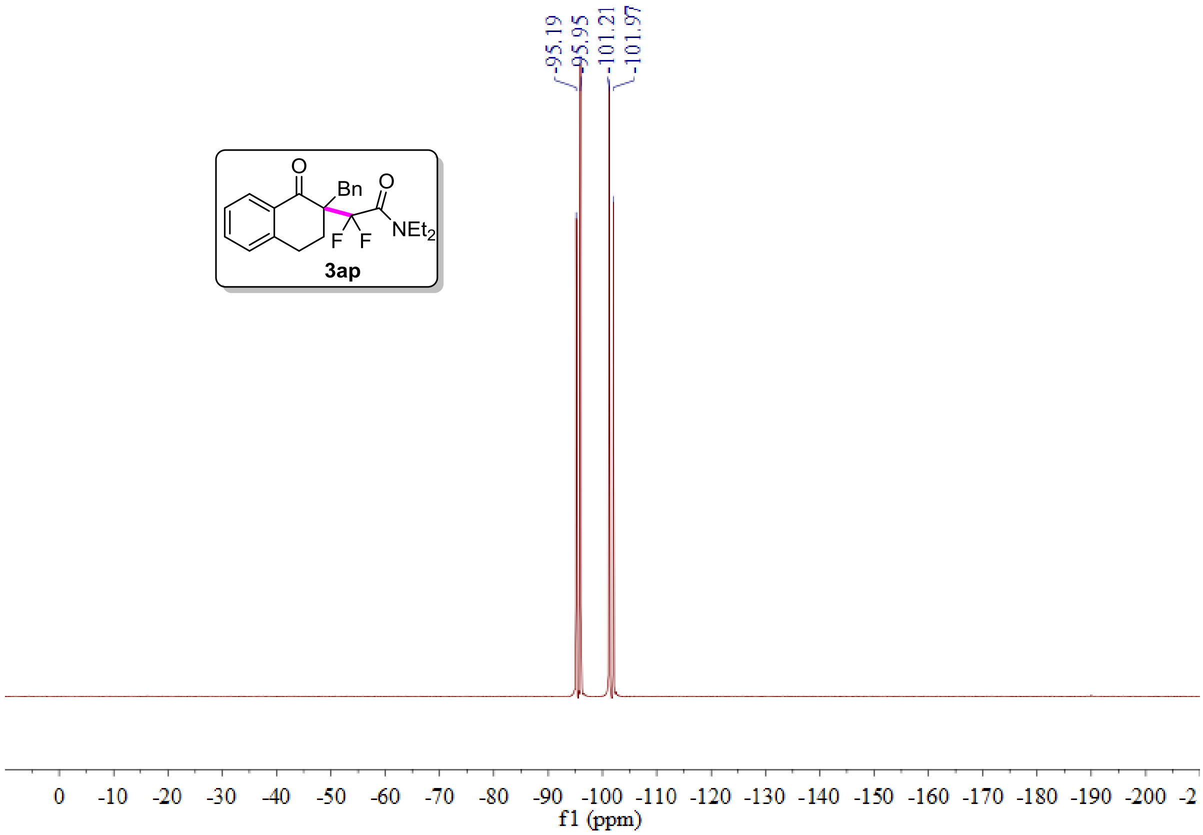


Supplementary Figure 126. $^{19}$F NMR of 3ap

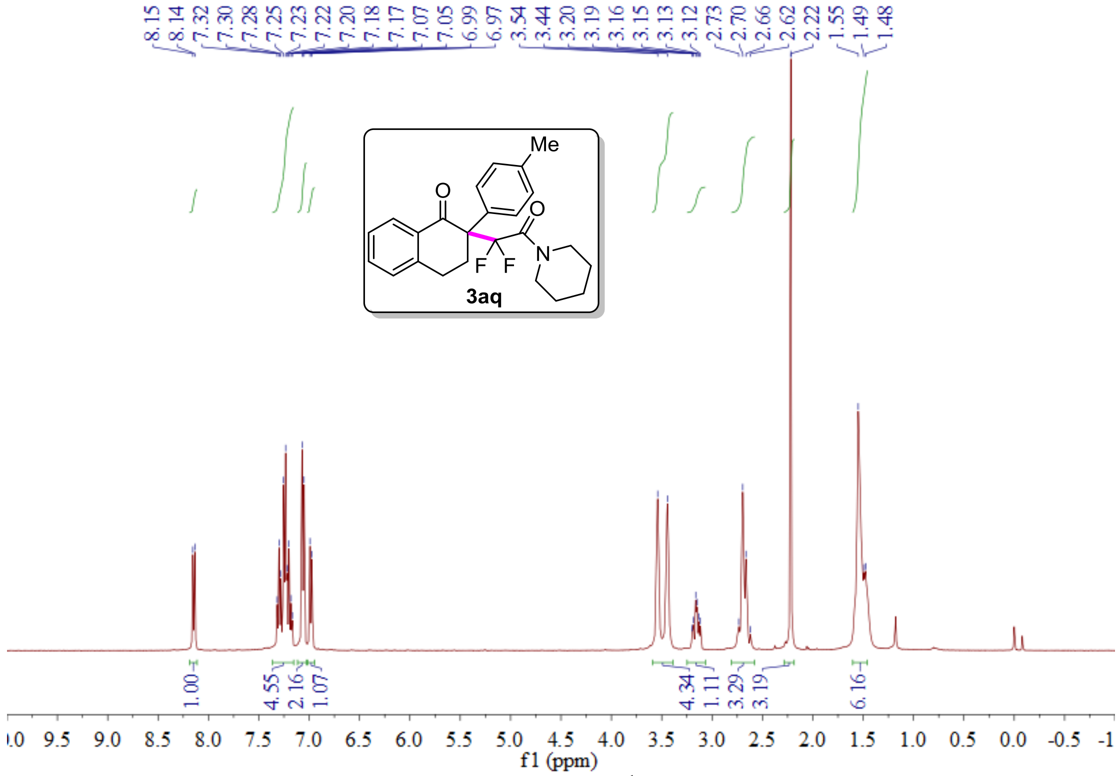

Supplementary Figure 127. $^{1}H$ NMR of 3aq

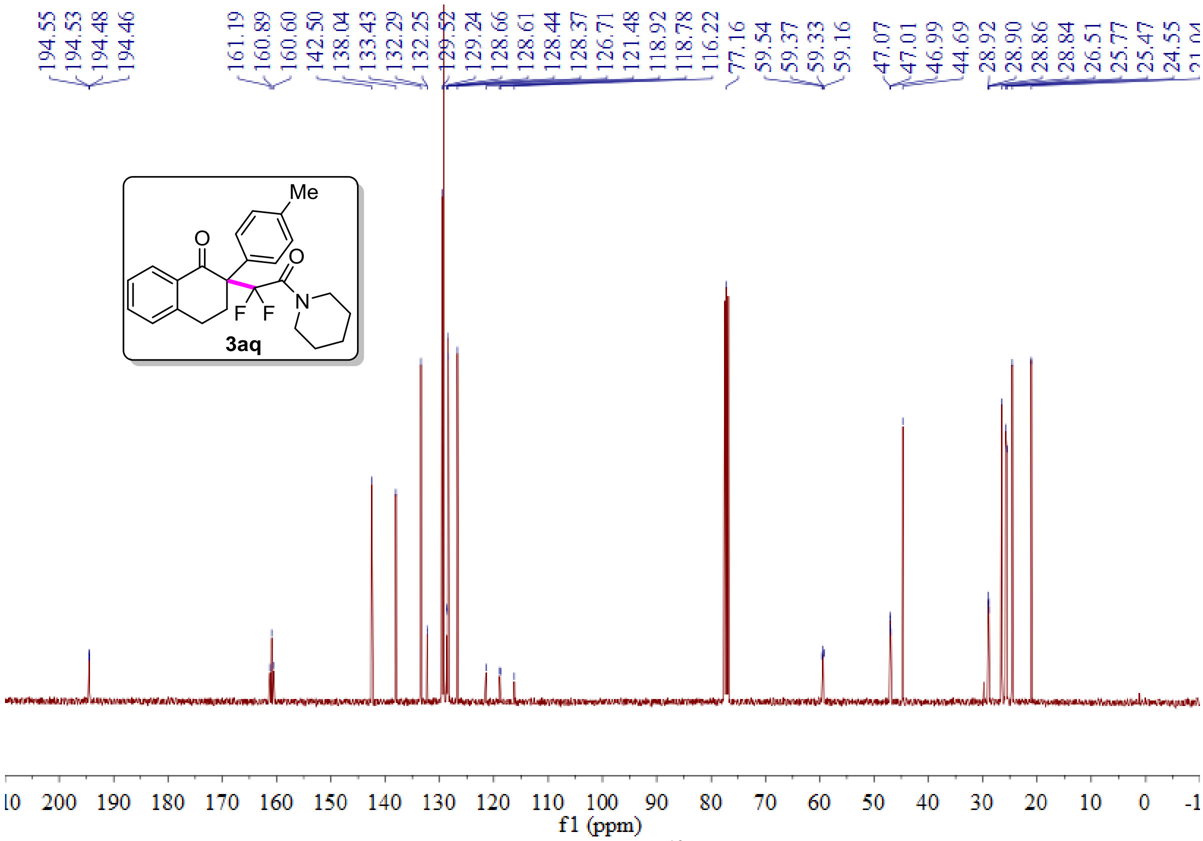

Supplementary Figure 128. $^{13}C$ NMR of 3aq

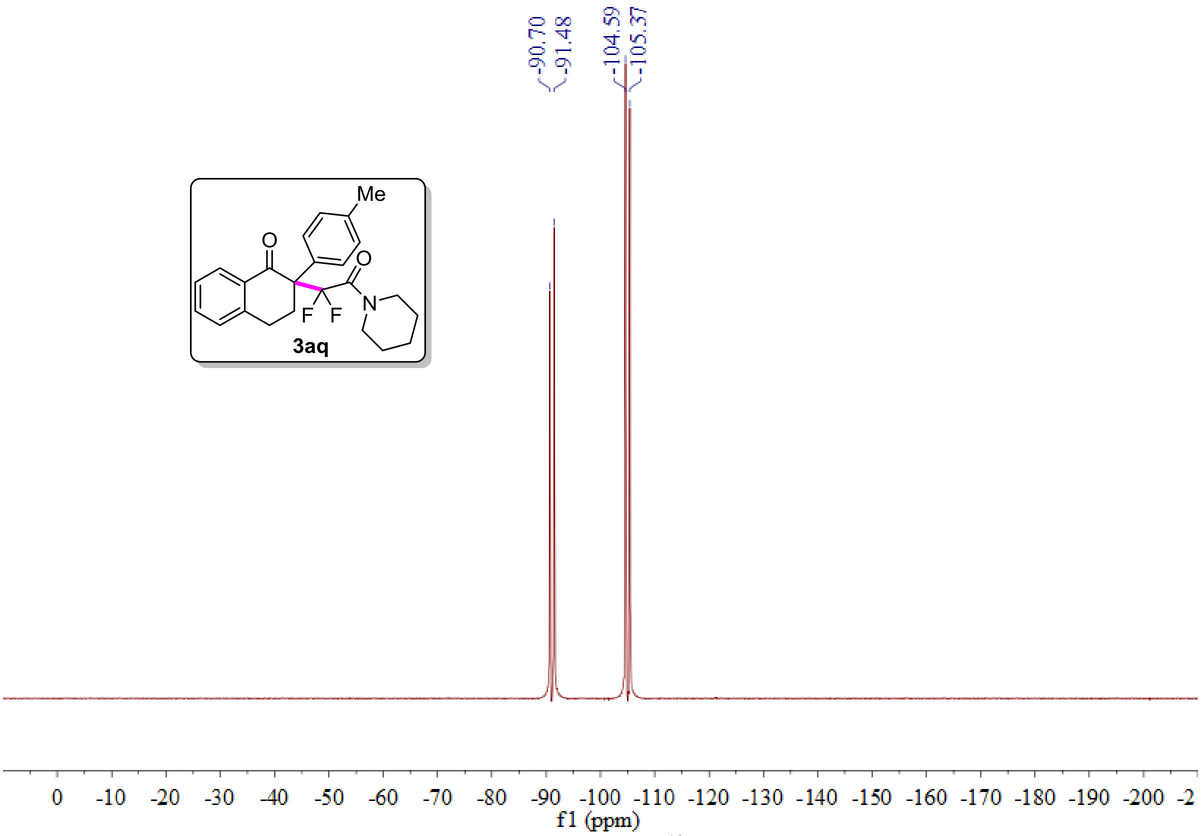


Supplementary Figure 129. $^{19}$F NMR of 3aq

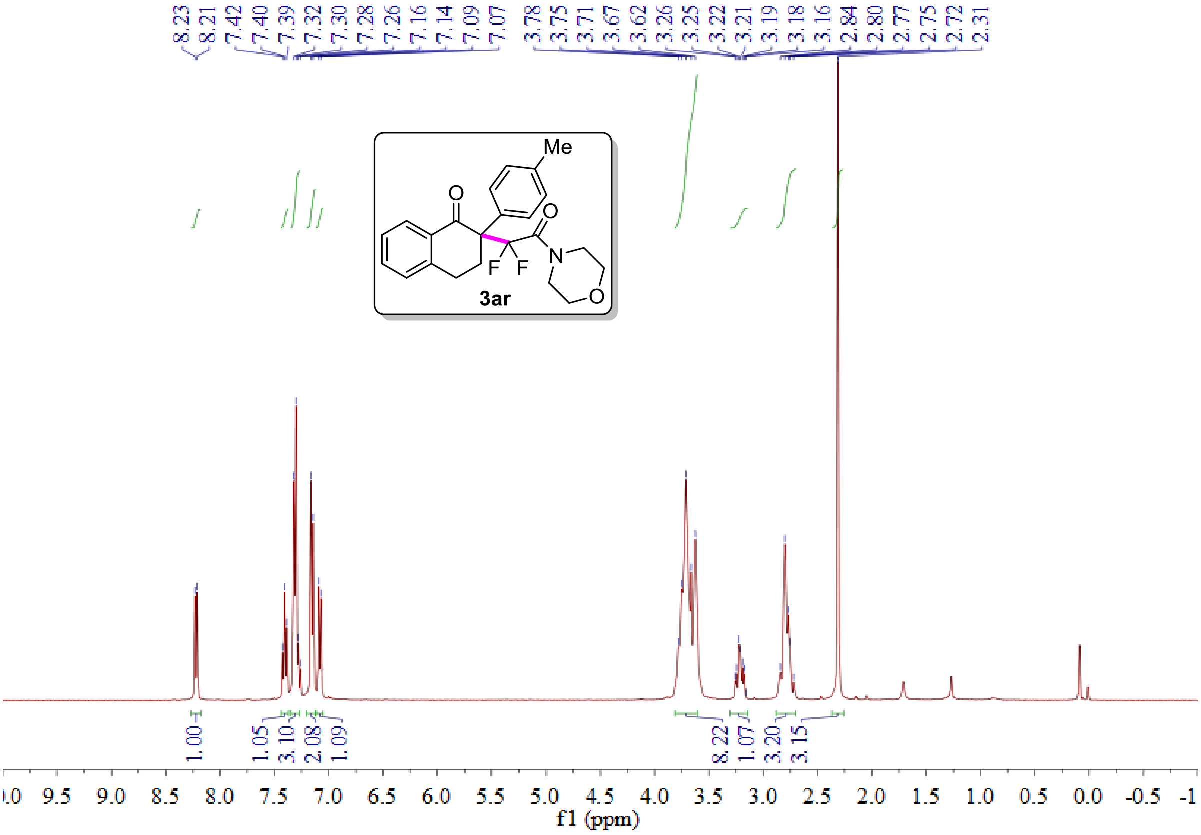


Supplementary Figure 130. $^{1}$H NMR of 3ar

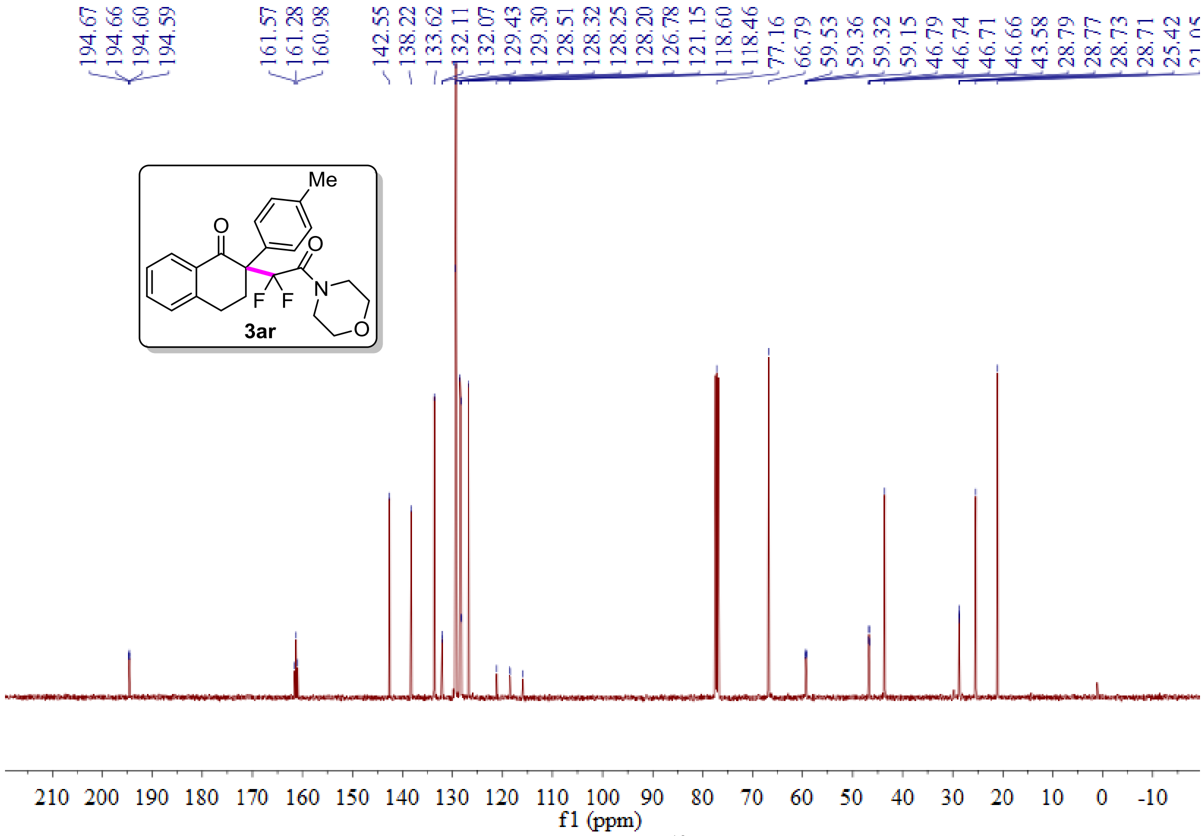


Supplementary Figure 131. $^{13}C$ NMR of 3ar

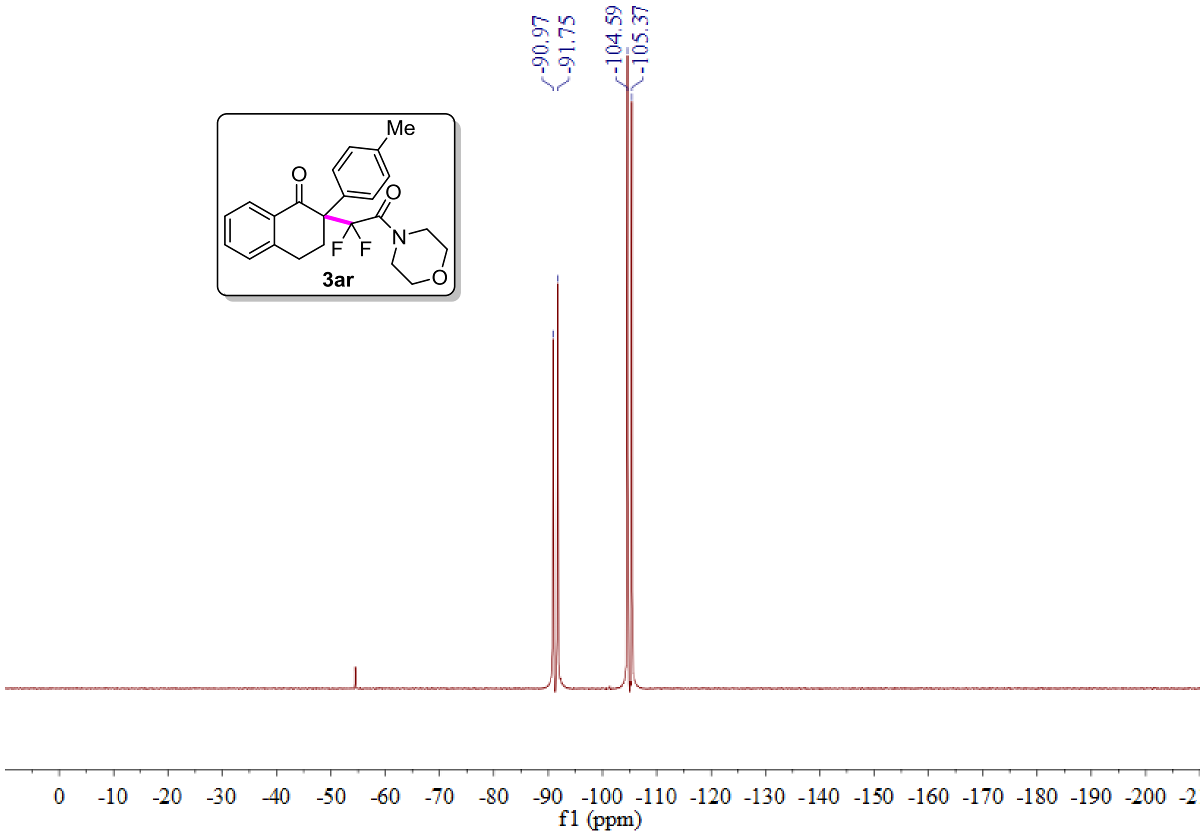


Supplementary Figure 132. $^{19}F$ NMR of 3ar

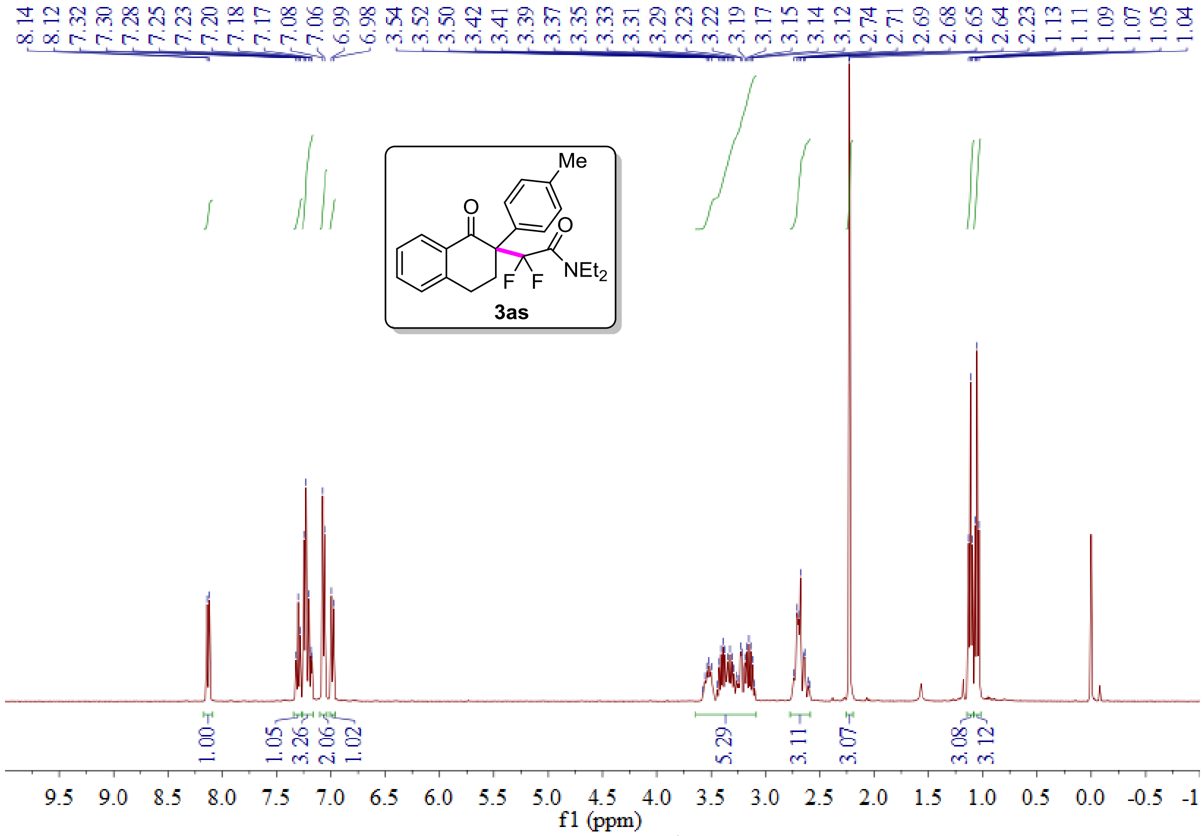

Supplementary Figure 133. $^{1}$H NMR of 3as

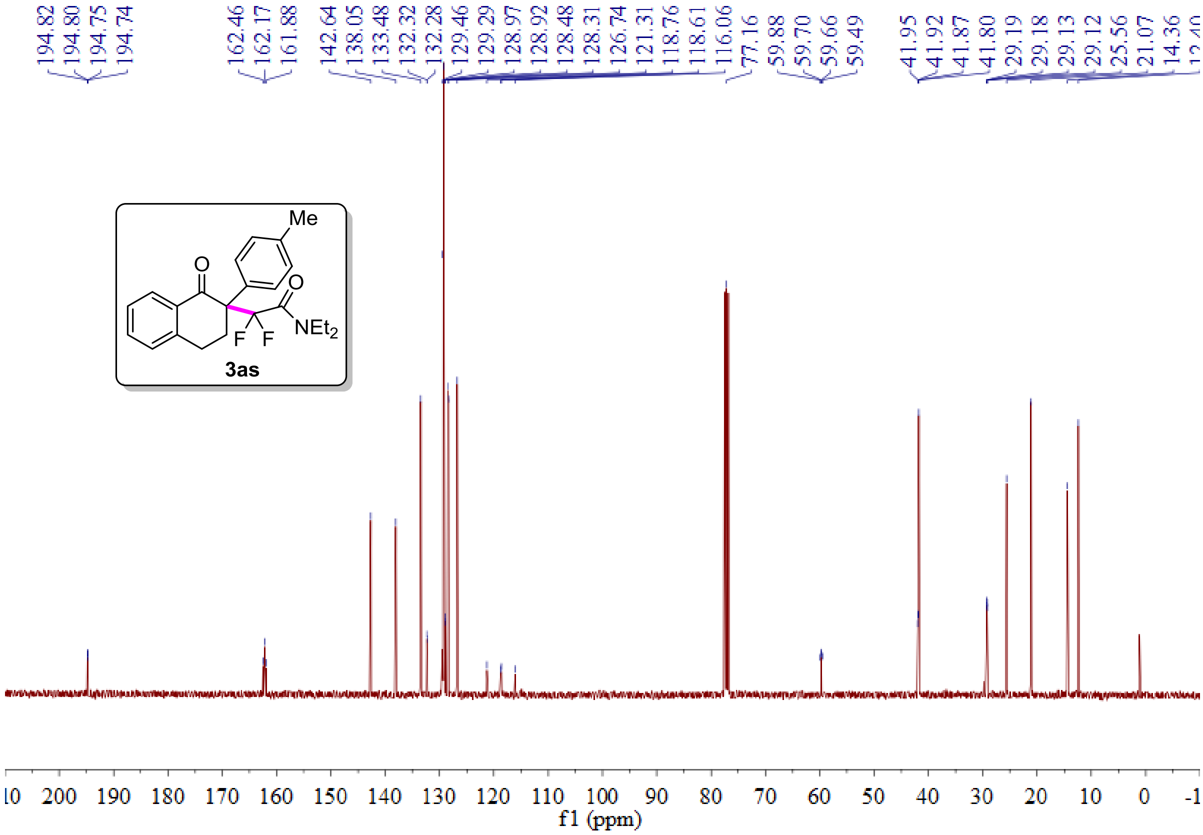

Supplementary Figure 134. $^{13}$C NMR of 3as

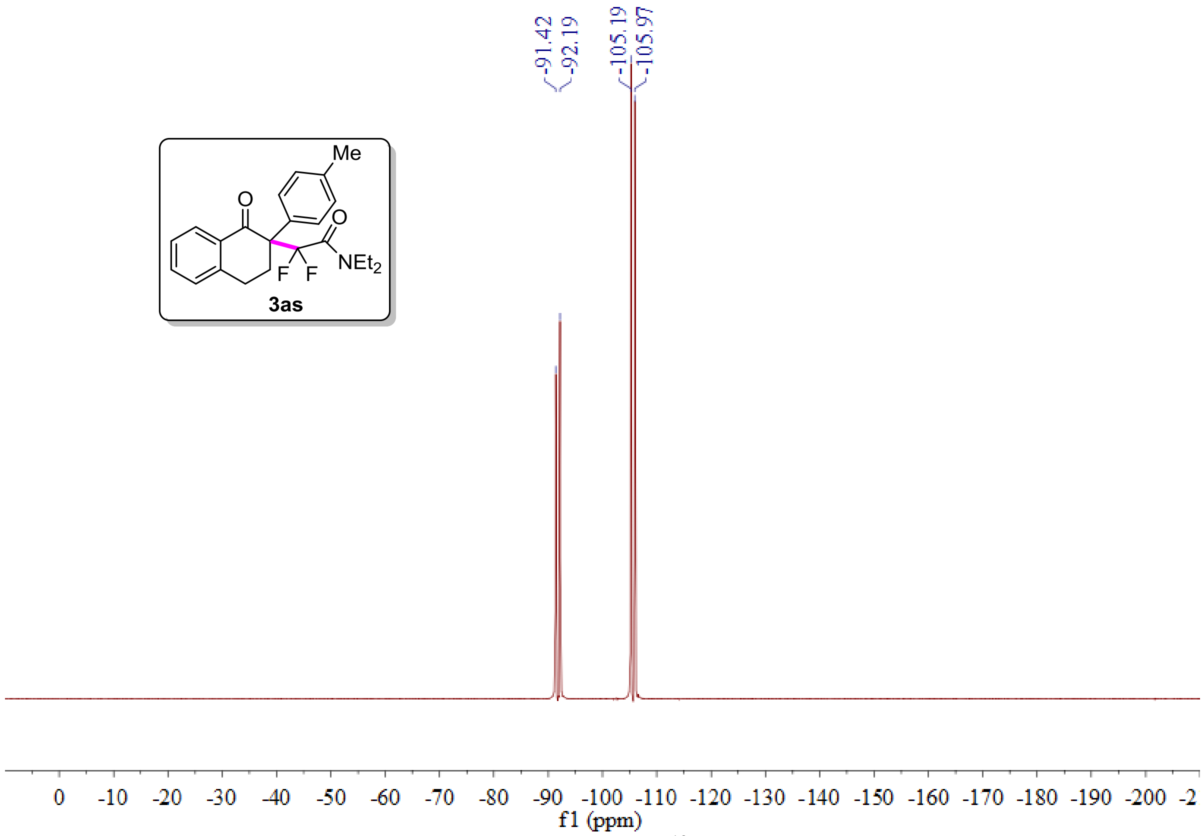


Supplementary Figure 135. $^{19}$F NMR of 3as

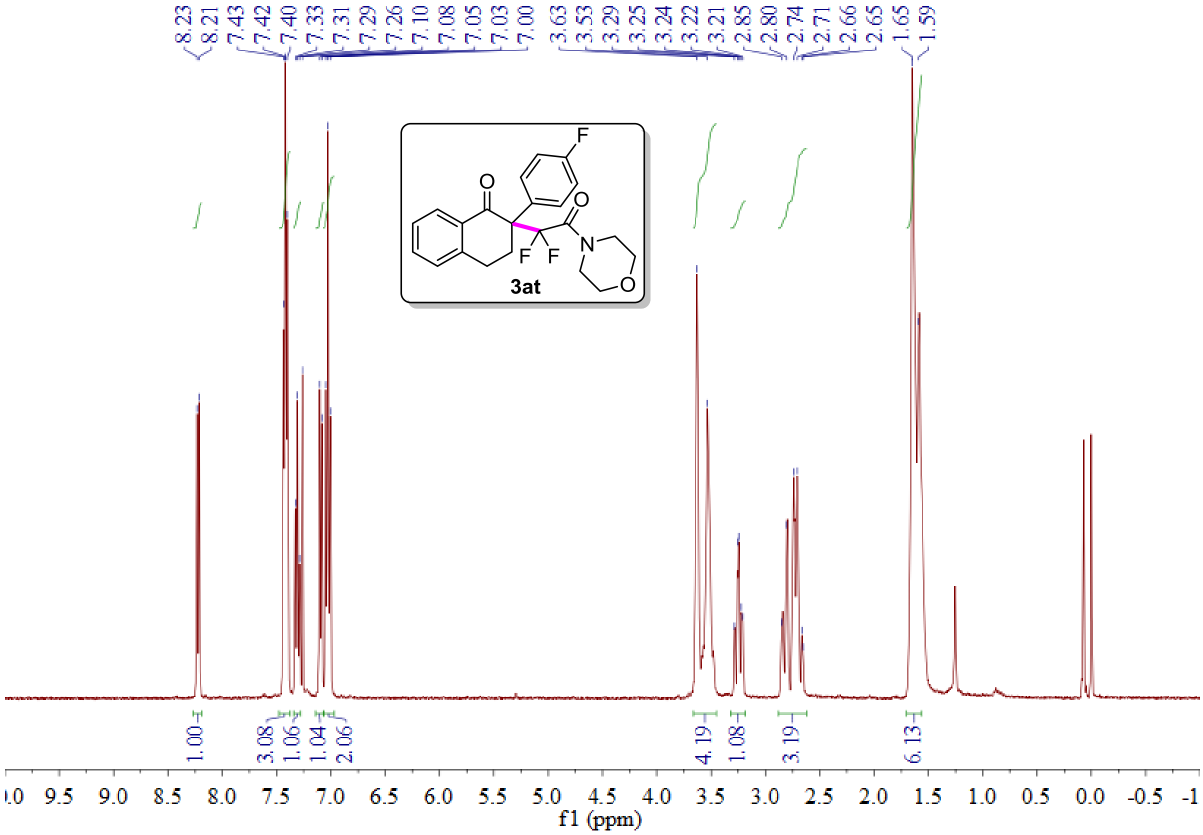


Supplementary Figure 136. $^{1}$H NMR of 3at

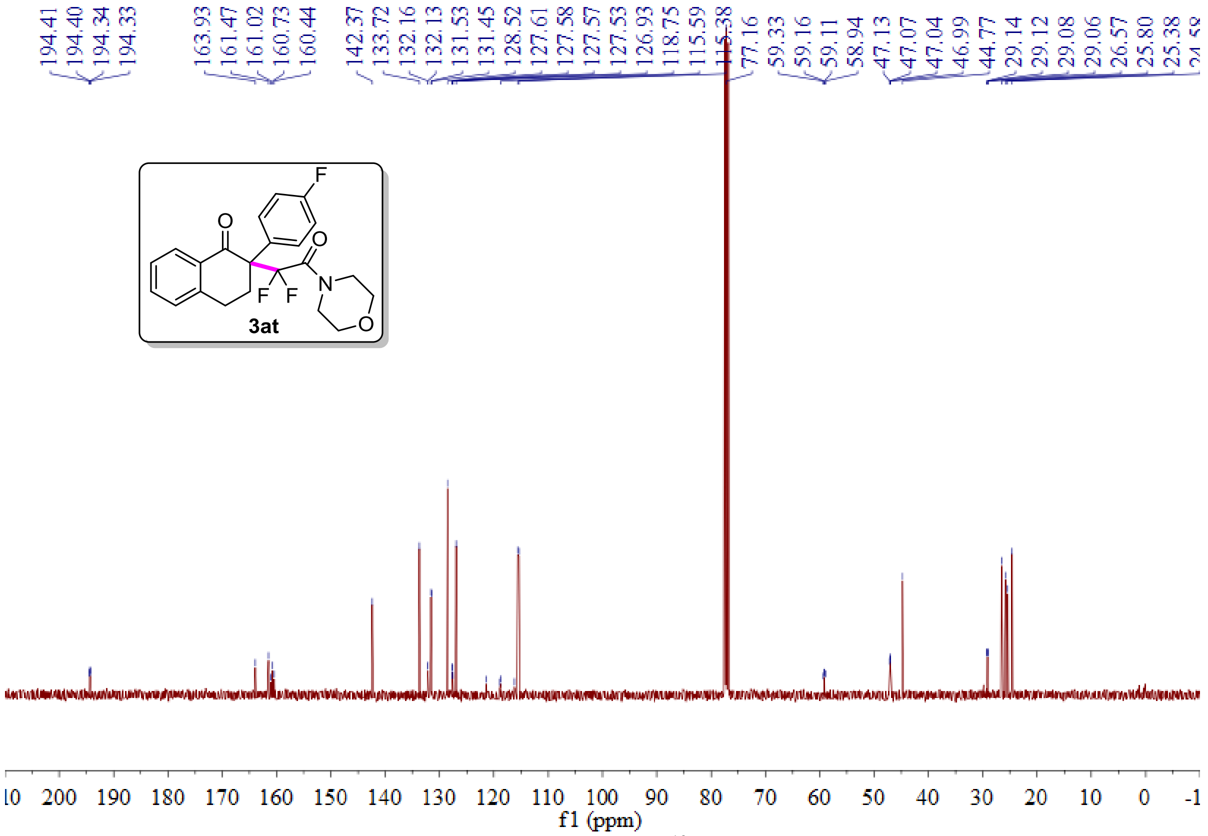


Supplementary Figure 137. $^{13}$C NMR of 3at

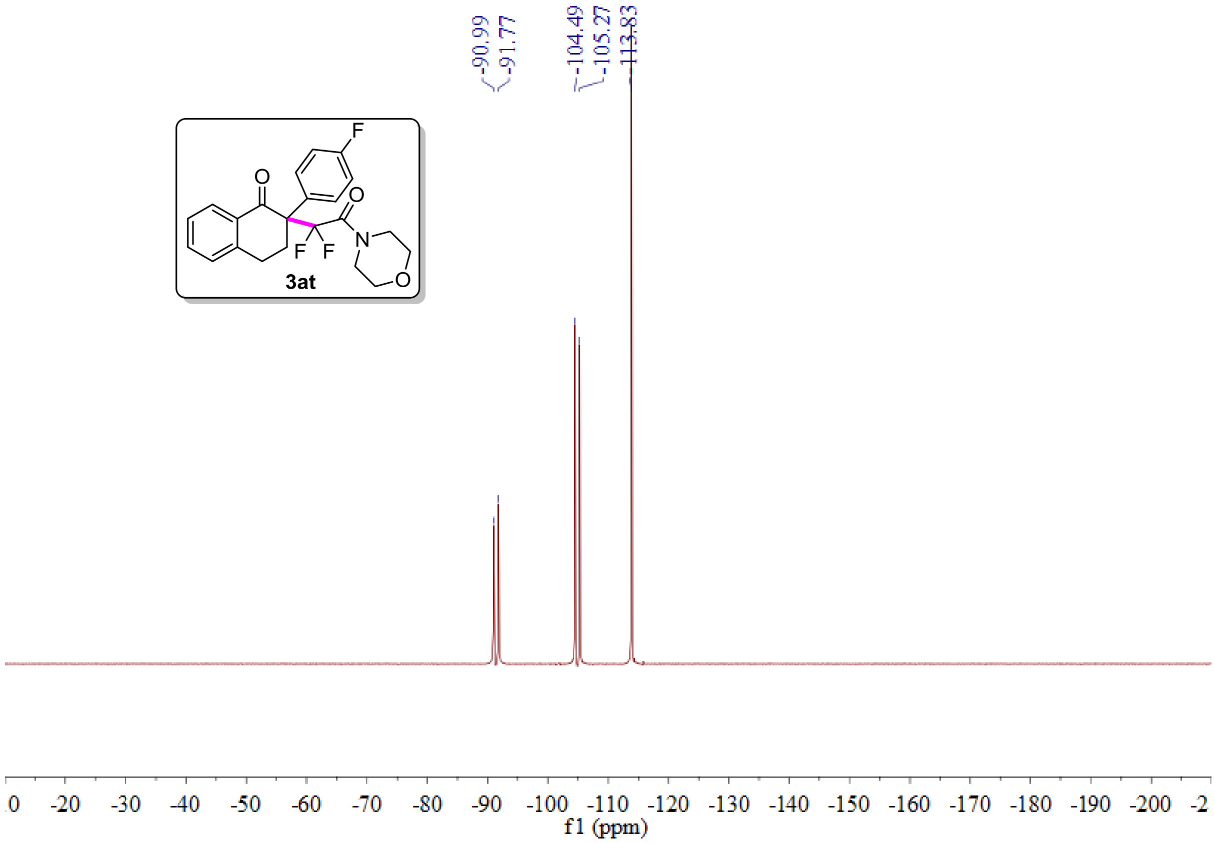


Supplementary Figure 138. $^{19}$F NMR of 3at

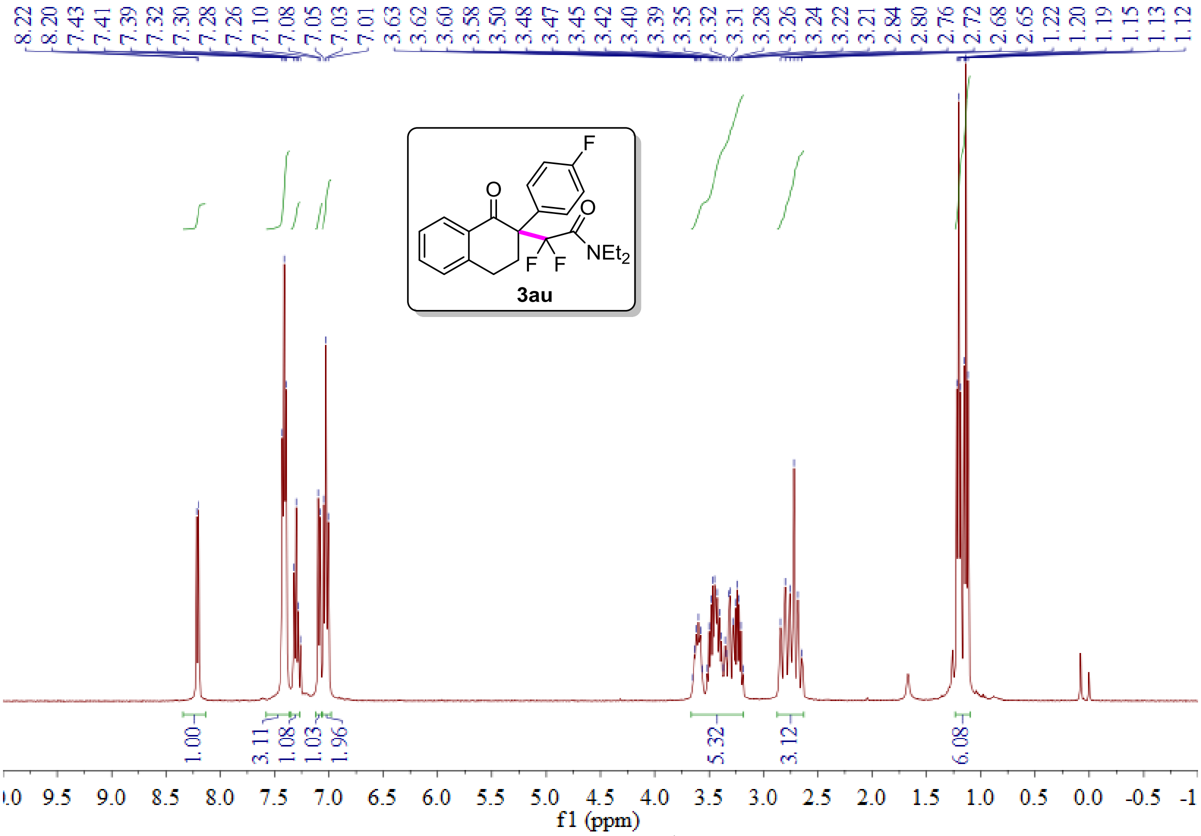

Supplementary Figure 139. $^{1}$H NMR of 3au

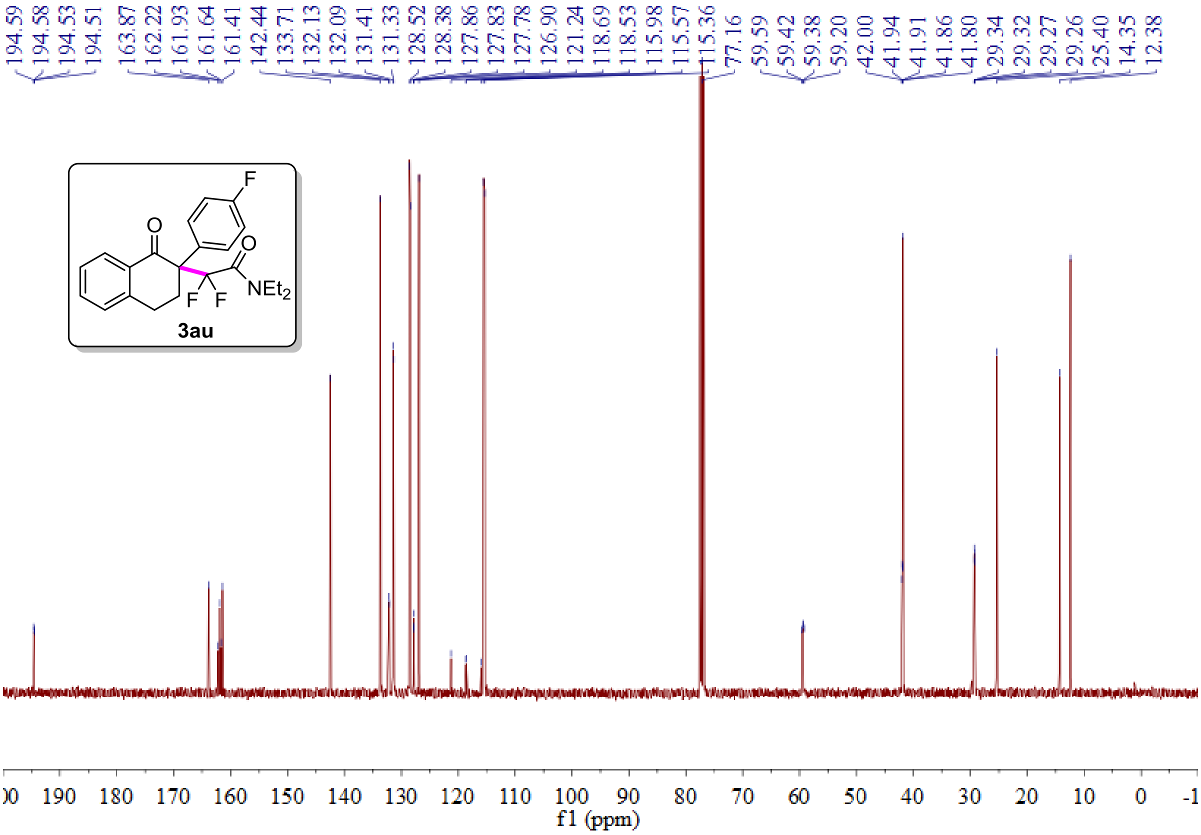

Supplementary Figure 140. $^{13}$C NMR of 3au

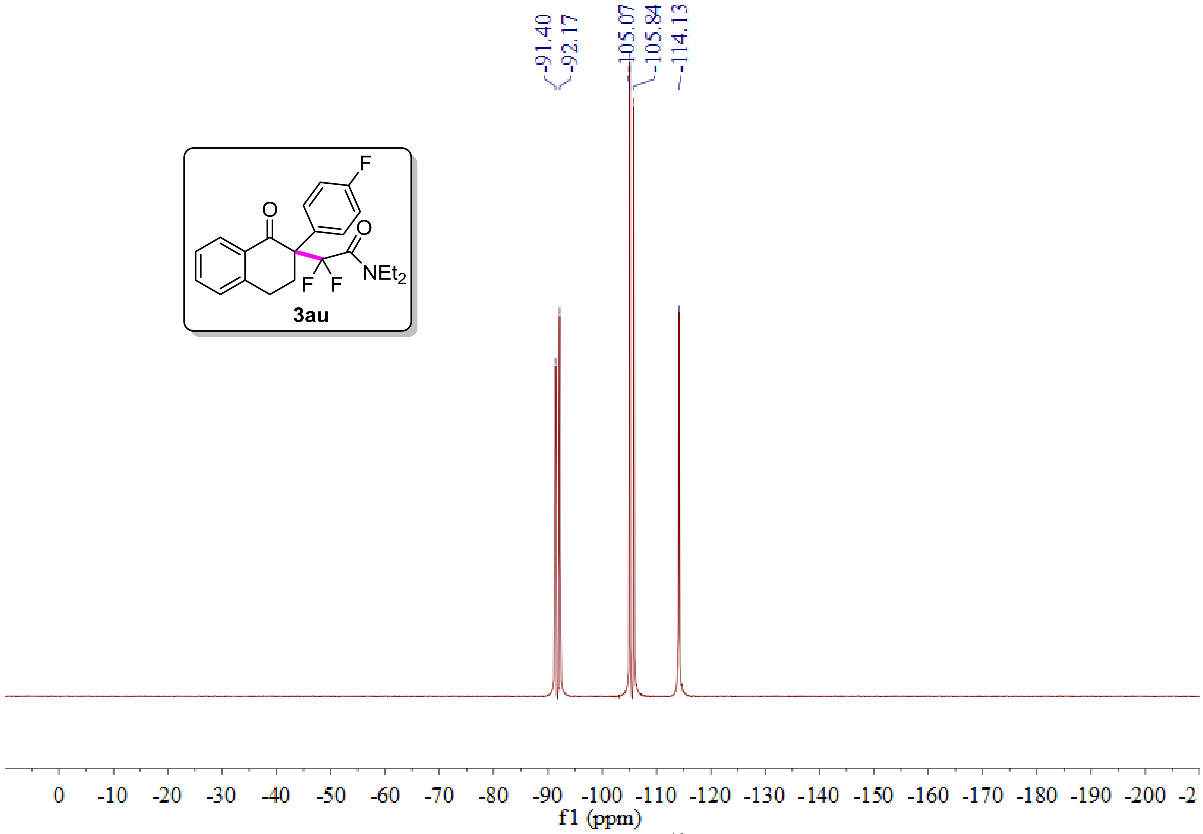


Supplementary Figure 141. $^{19}F$ NMR of 3au

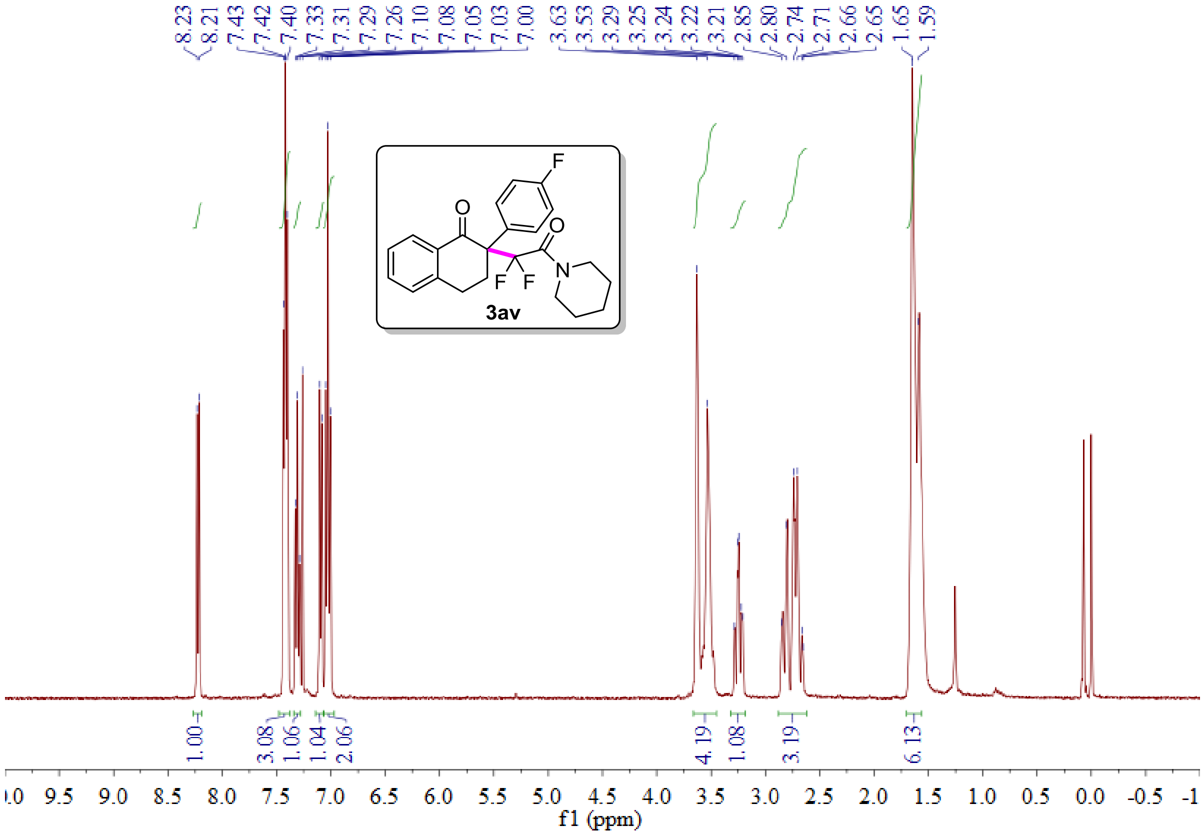


Supplementary Figure 142. $^{1}H$ NMR of 3av

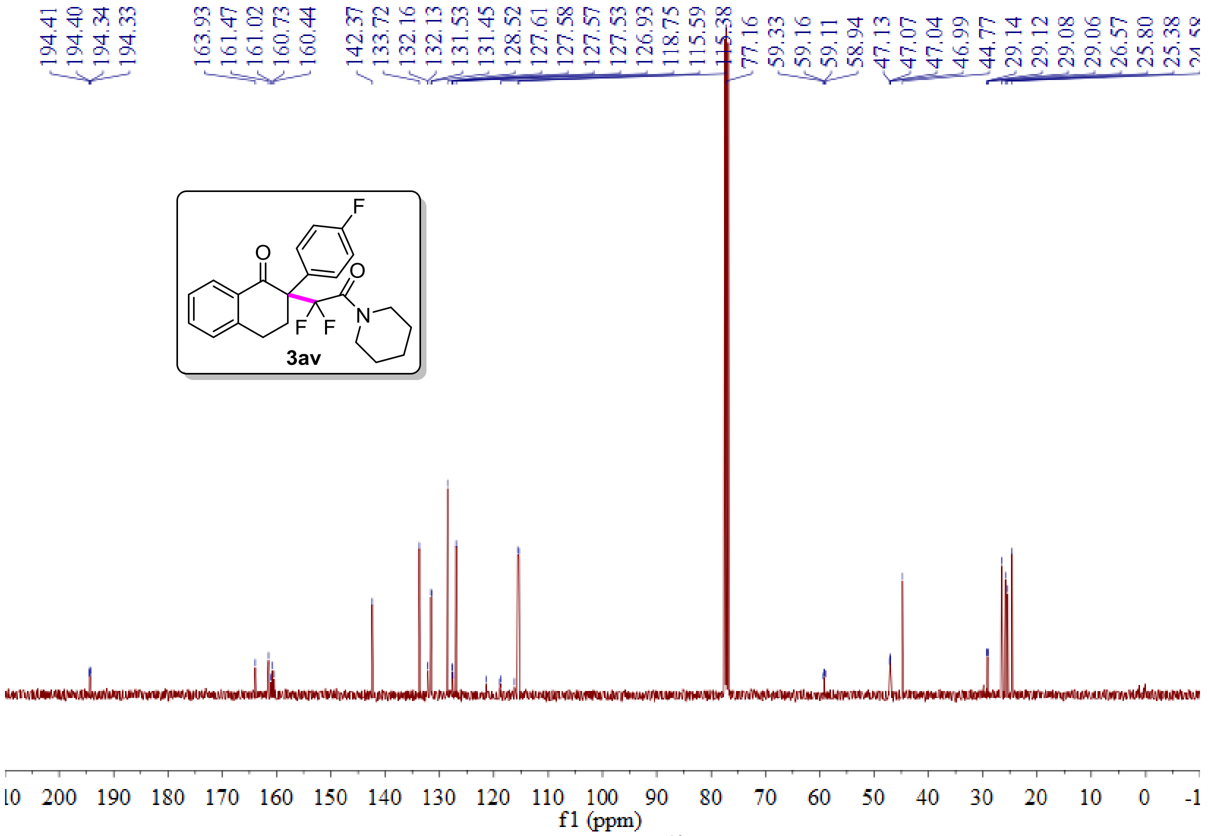

Supplementary Figure 143. $^{13}C$ NMR of 3av

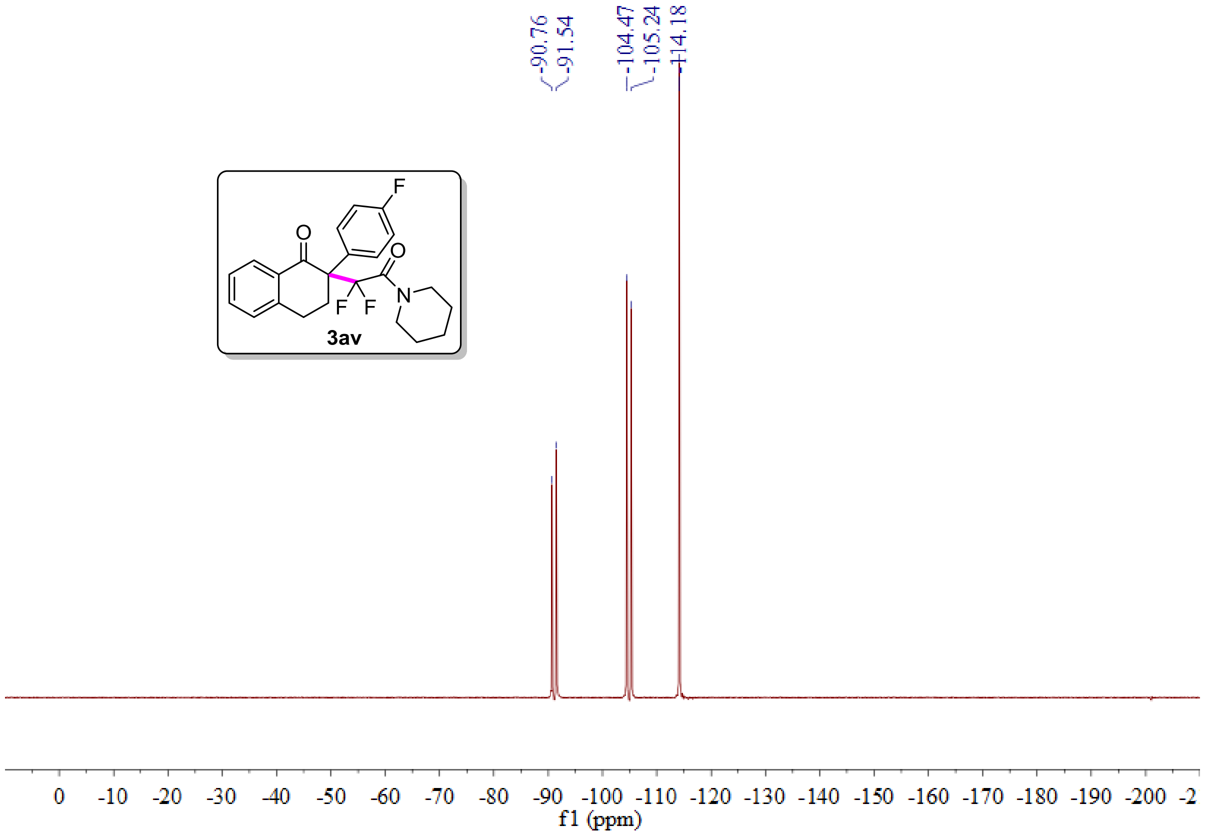

Supplementary Figure 144. $^{19}F$ NMR of 3av

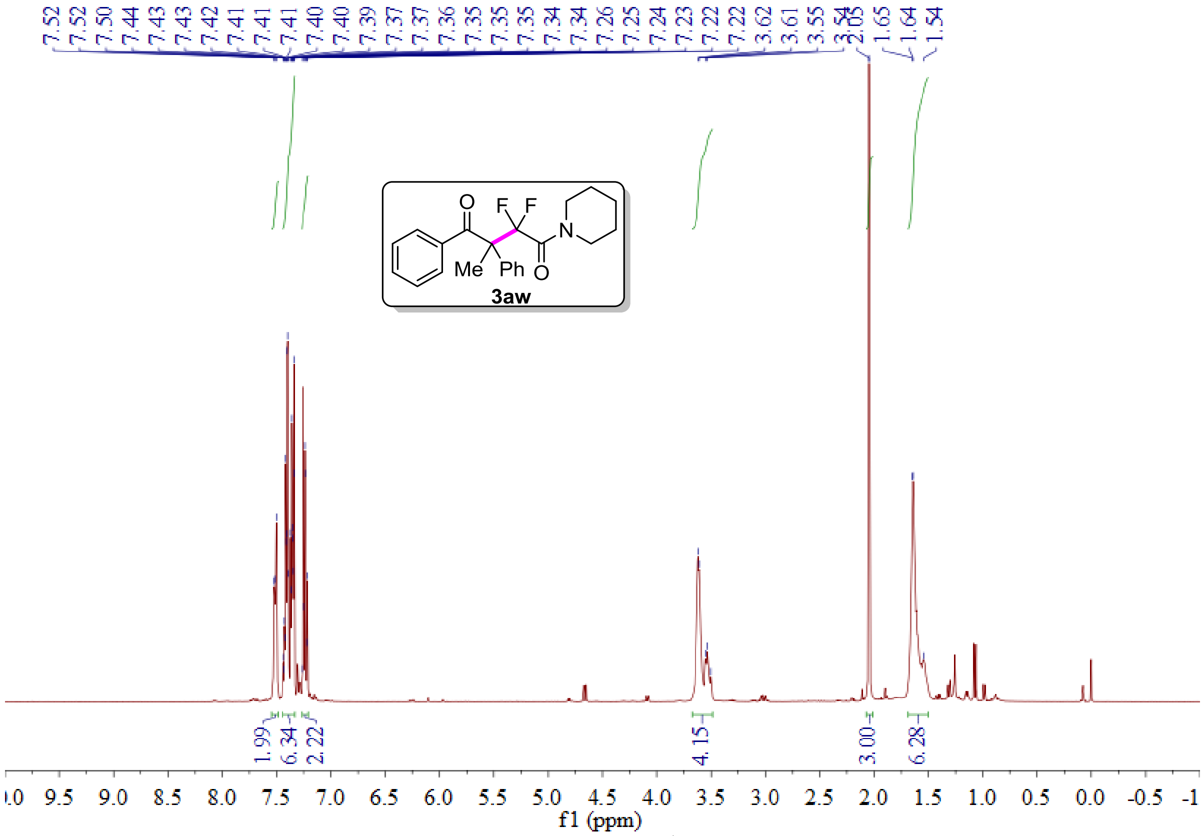

Supplementary Figure 145. $^{1}$H NMR of 3aw

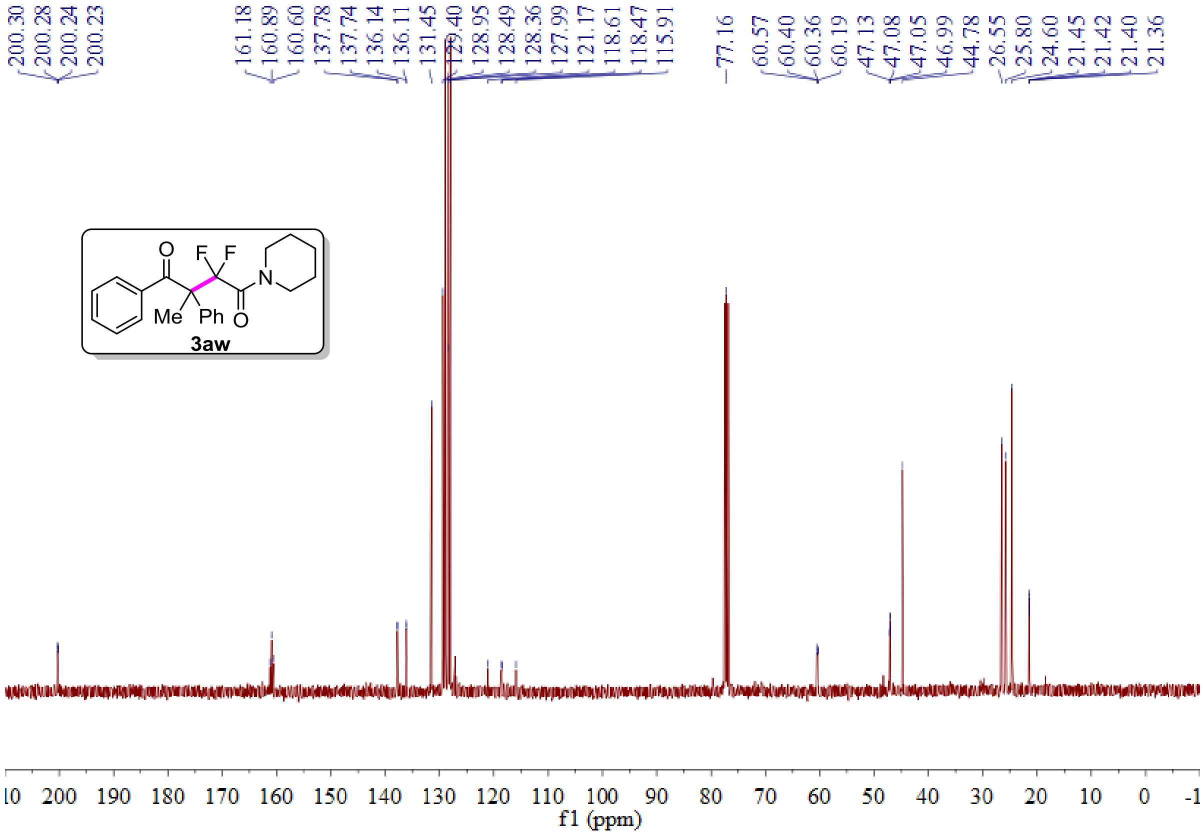

Supplementary Figure 146. $^{13}$C NMR of 3aw

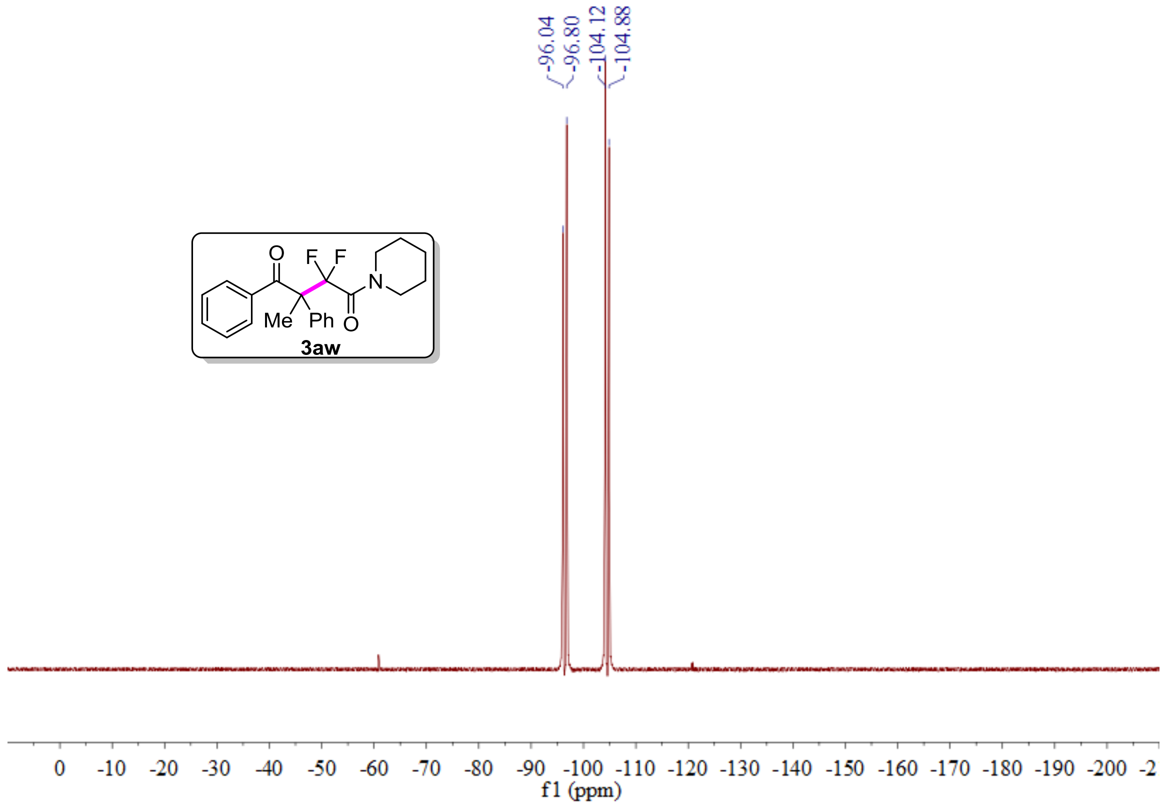


Supplementary Figure 147. $^{19}F$ NMR of 3aw

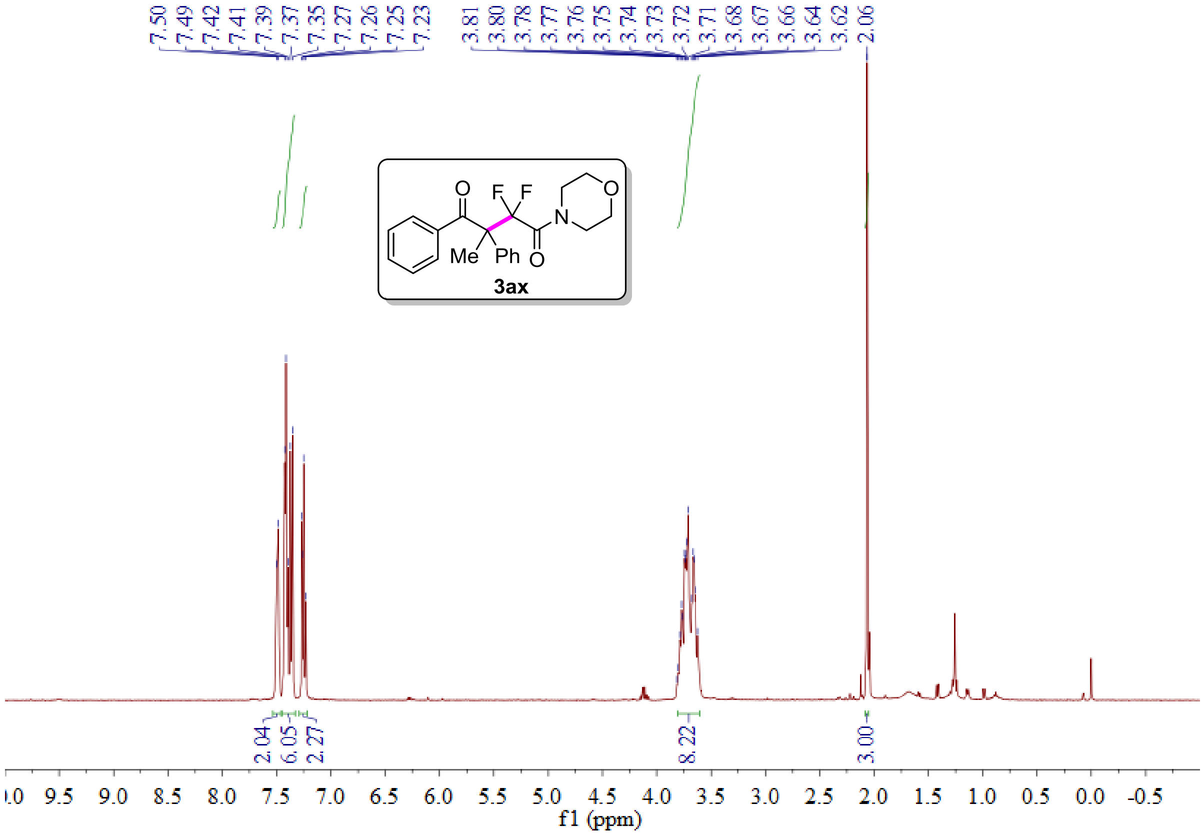


Supplementary Figure 148. $^{1}H$ NMR of 3ax

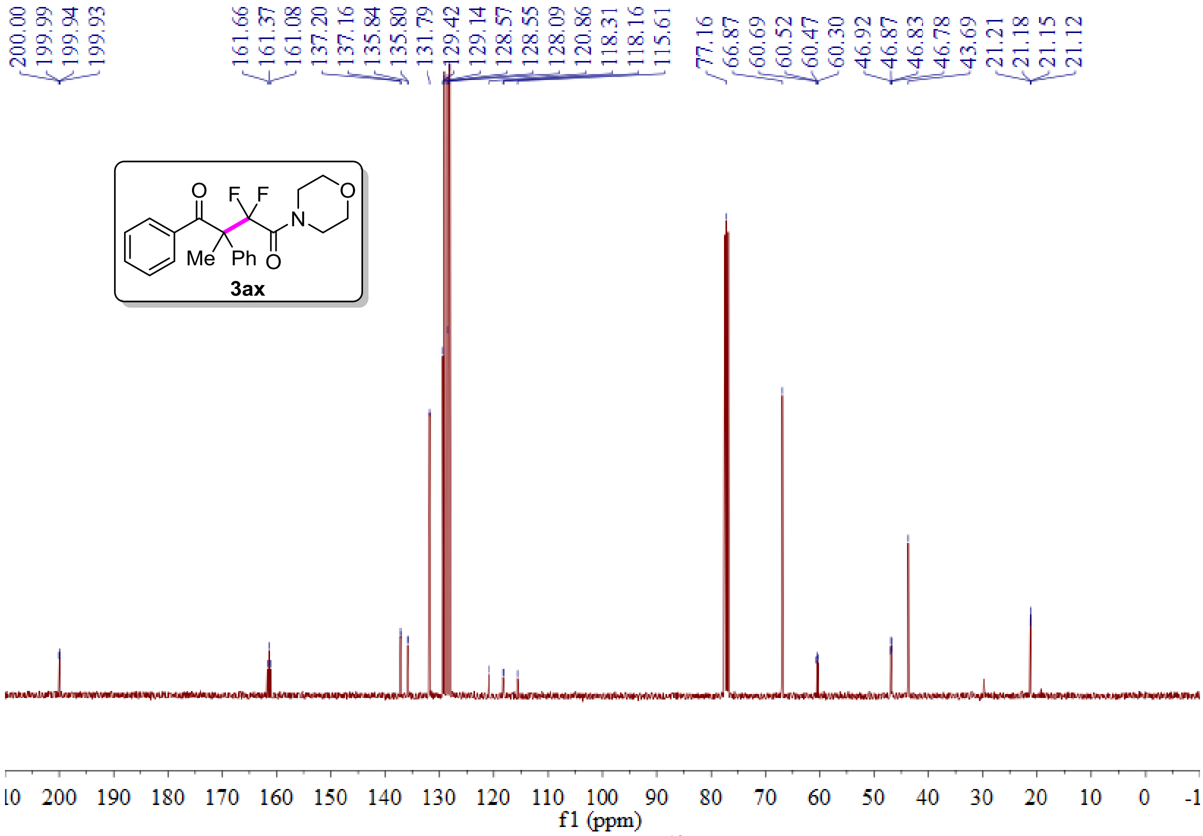


Supplementary Figure 149. $^{13}$C NMR of 3ax

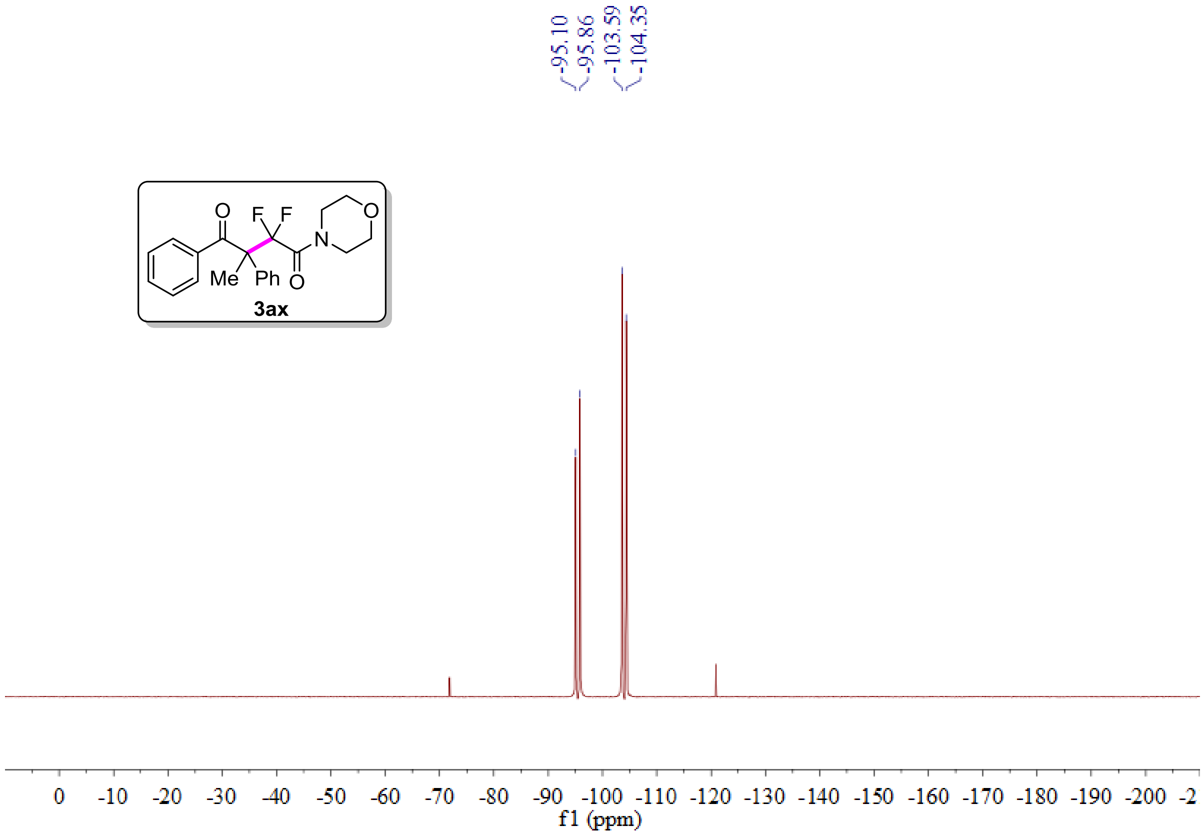


Supplementary Figure 150. $^{19}$F NMR of 3ax

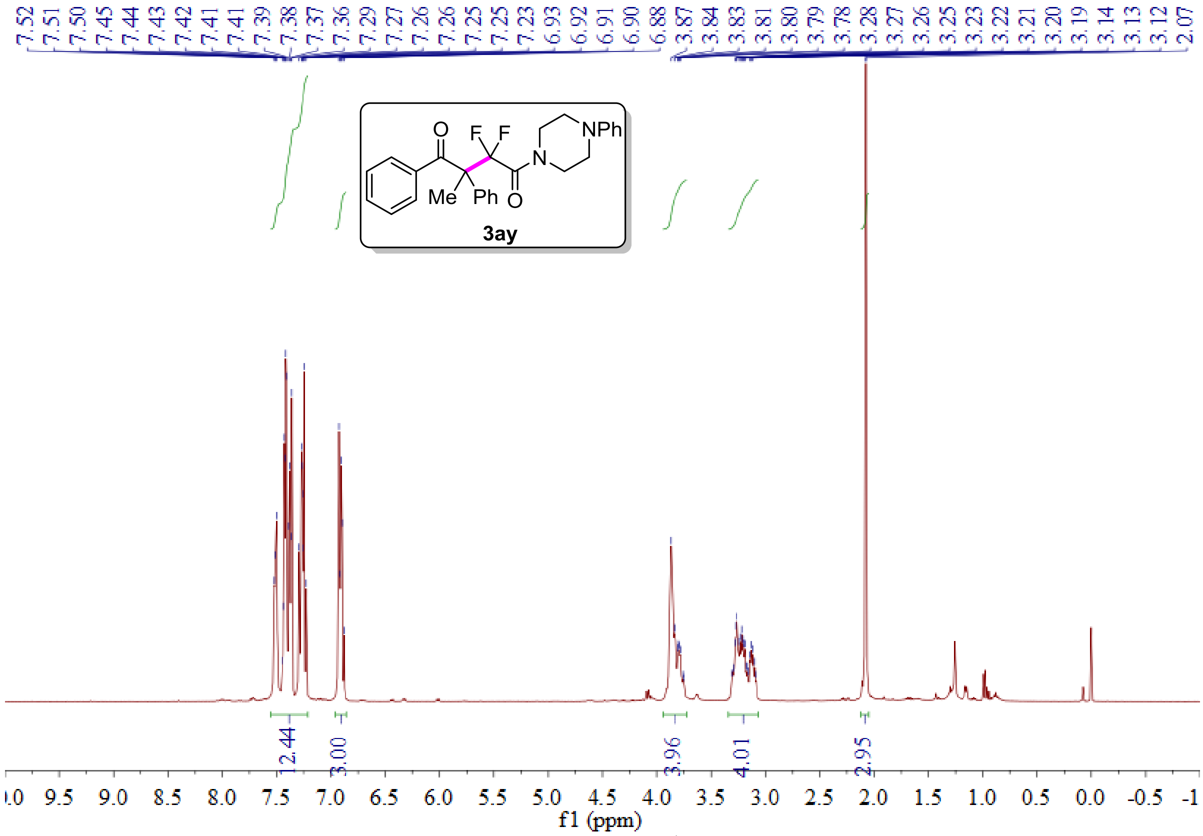

Supplementary Figure 151. $^{1}$H NMR of 3ay

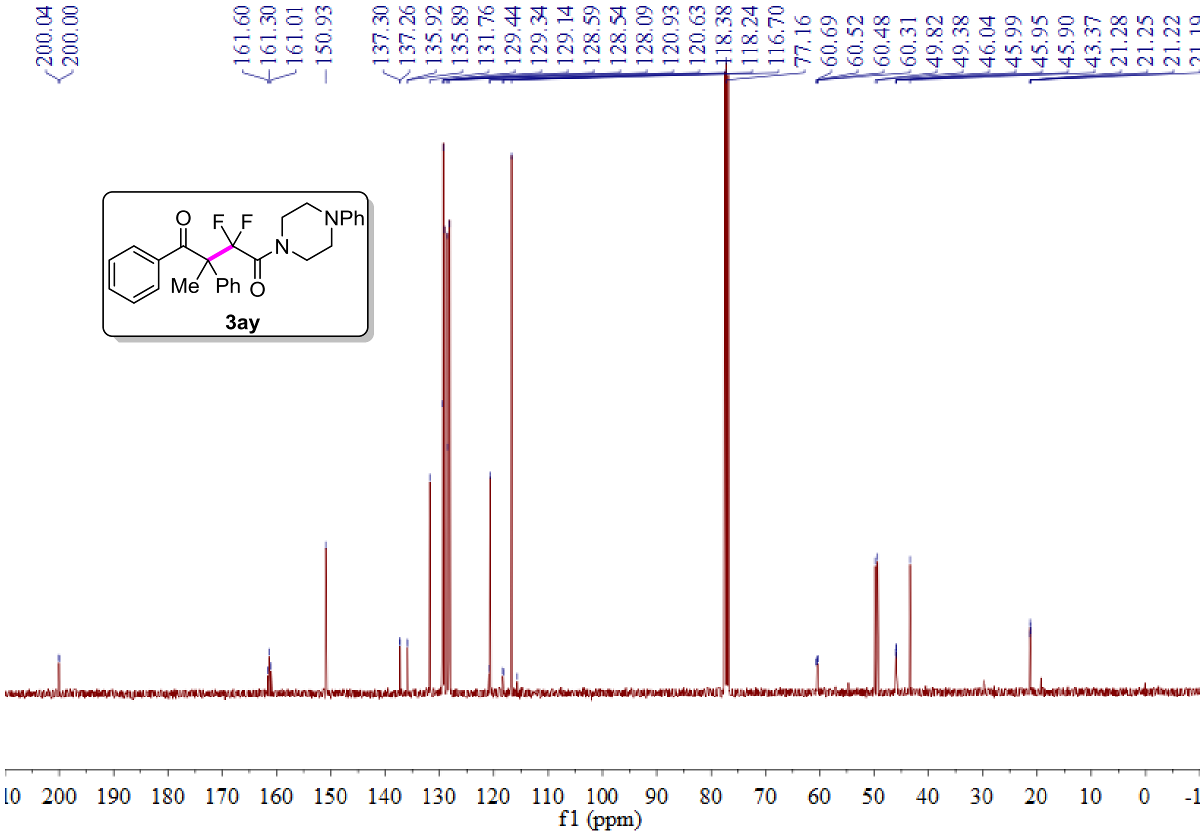

Supplementary Figure 152. $^{13}$C NMR of 3ay

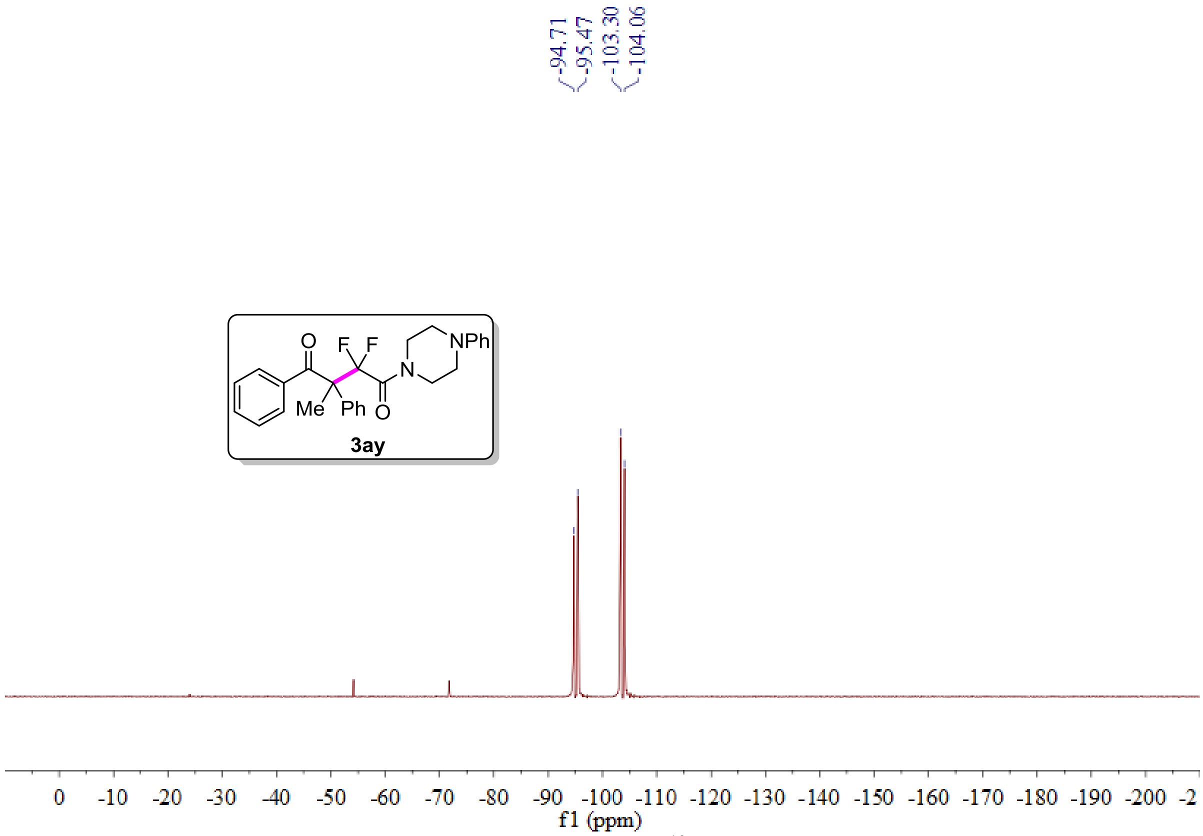


Supplementary Figure 153. $^{19}F$ NMR of 3ay

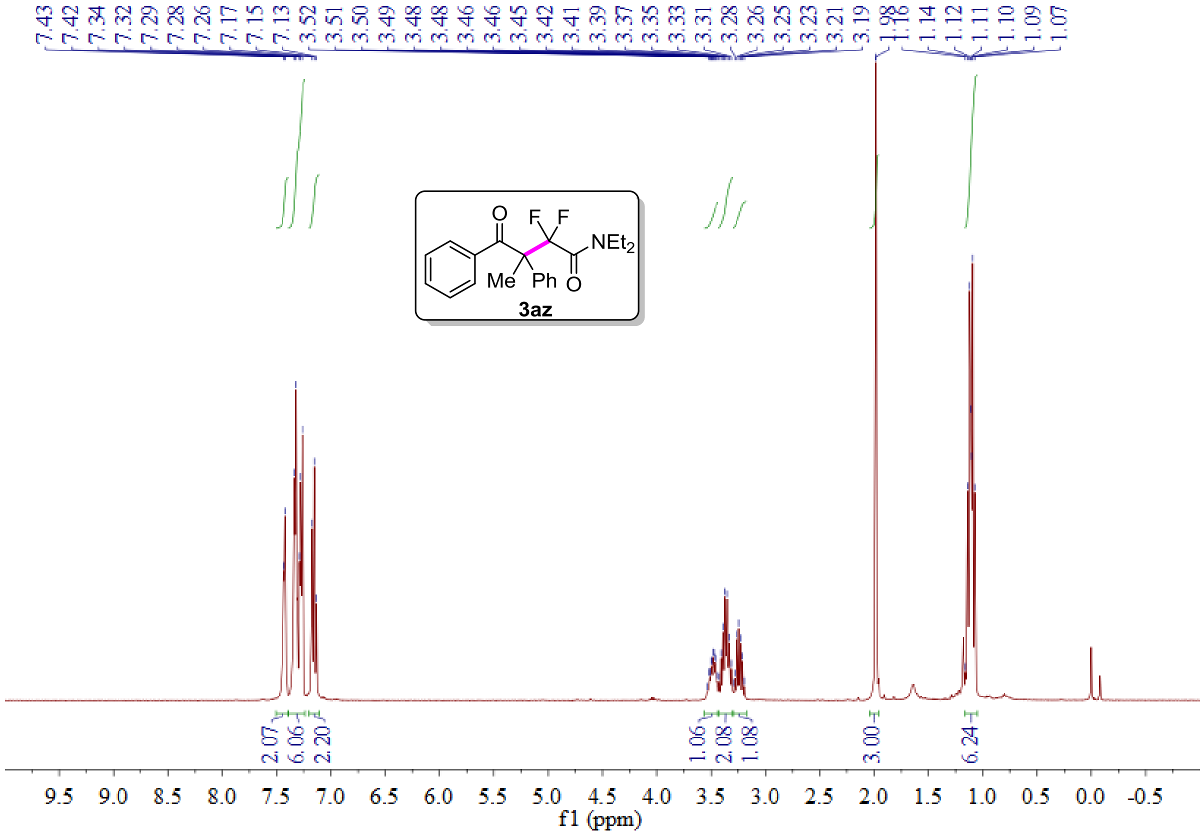


Supplementary Figure 154. $^{1}H$ NMR of 3az

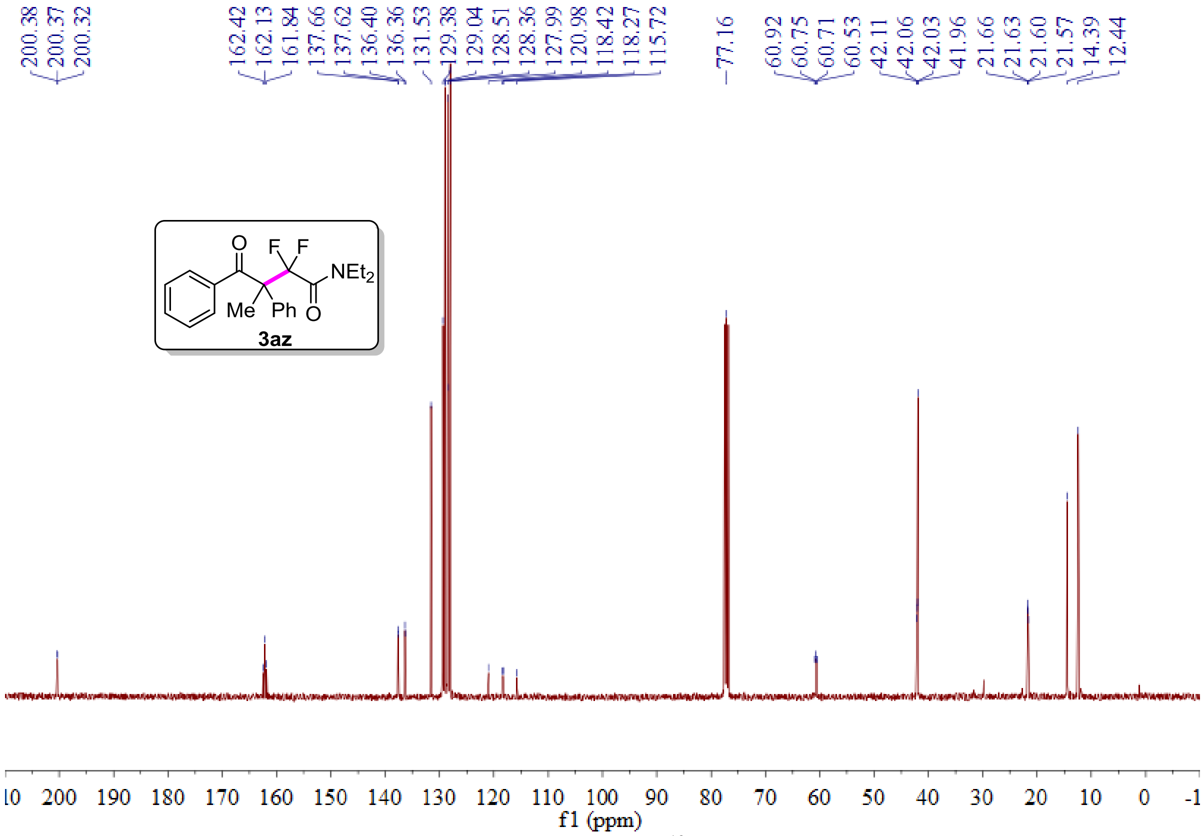


Supplementary Figure 155. $^{13}$C NMR of 3az

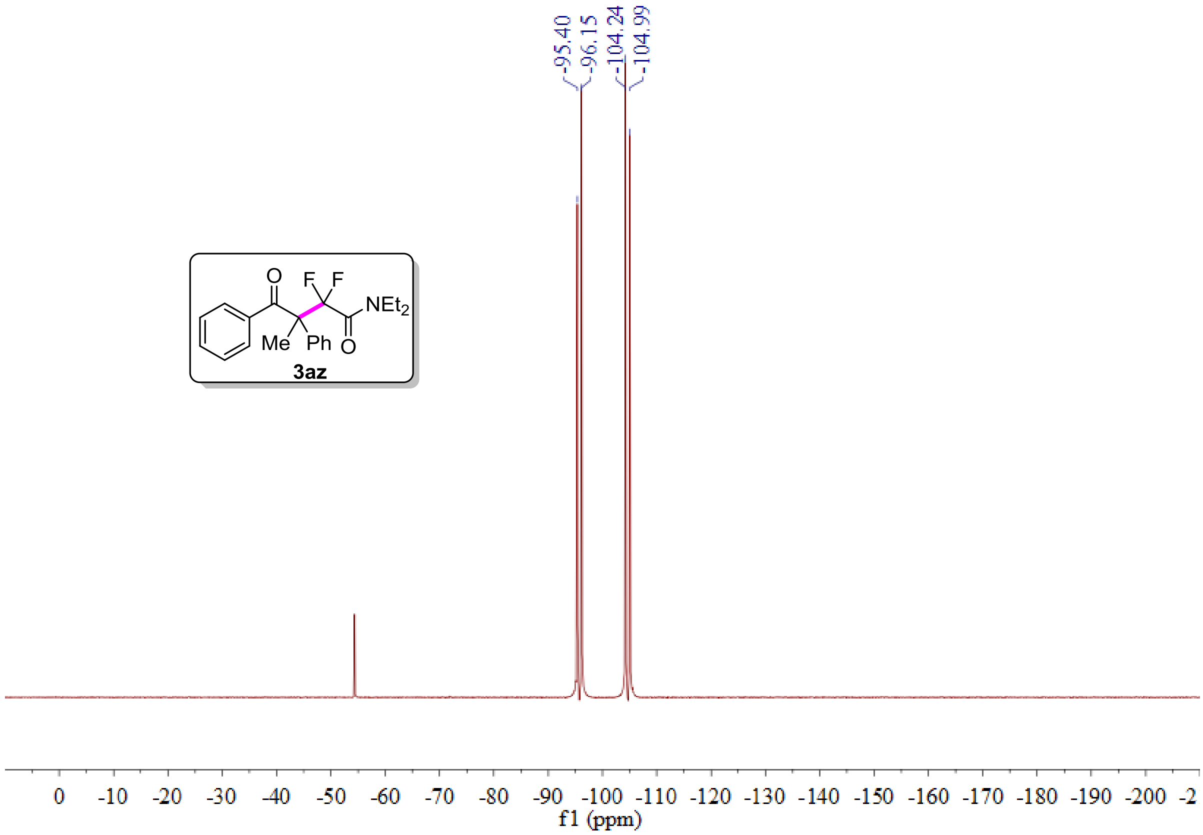


Supplementary Figure 156. $^{19}$F NMR of 3az

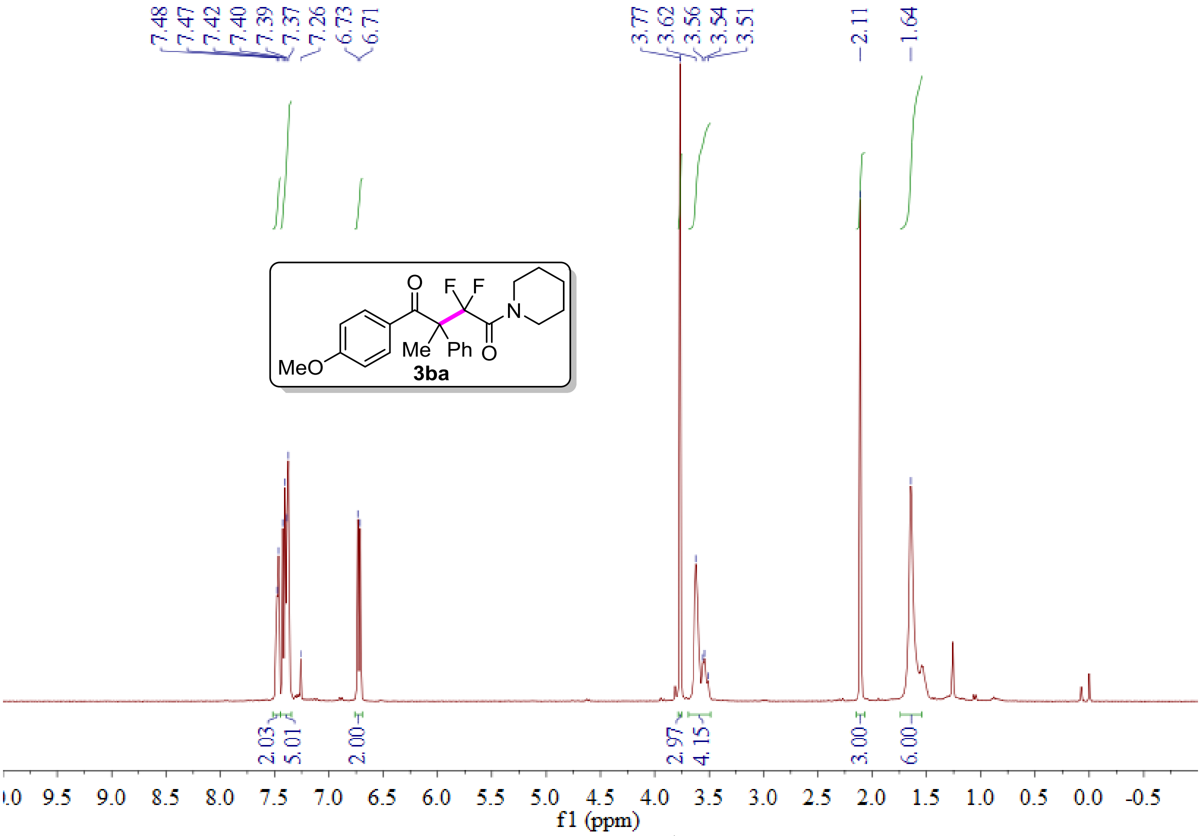


Supplementary Figure 157. $^{1}H$ NMR of 3ba

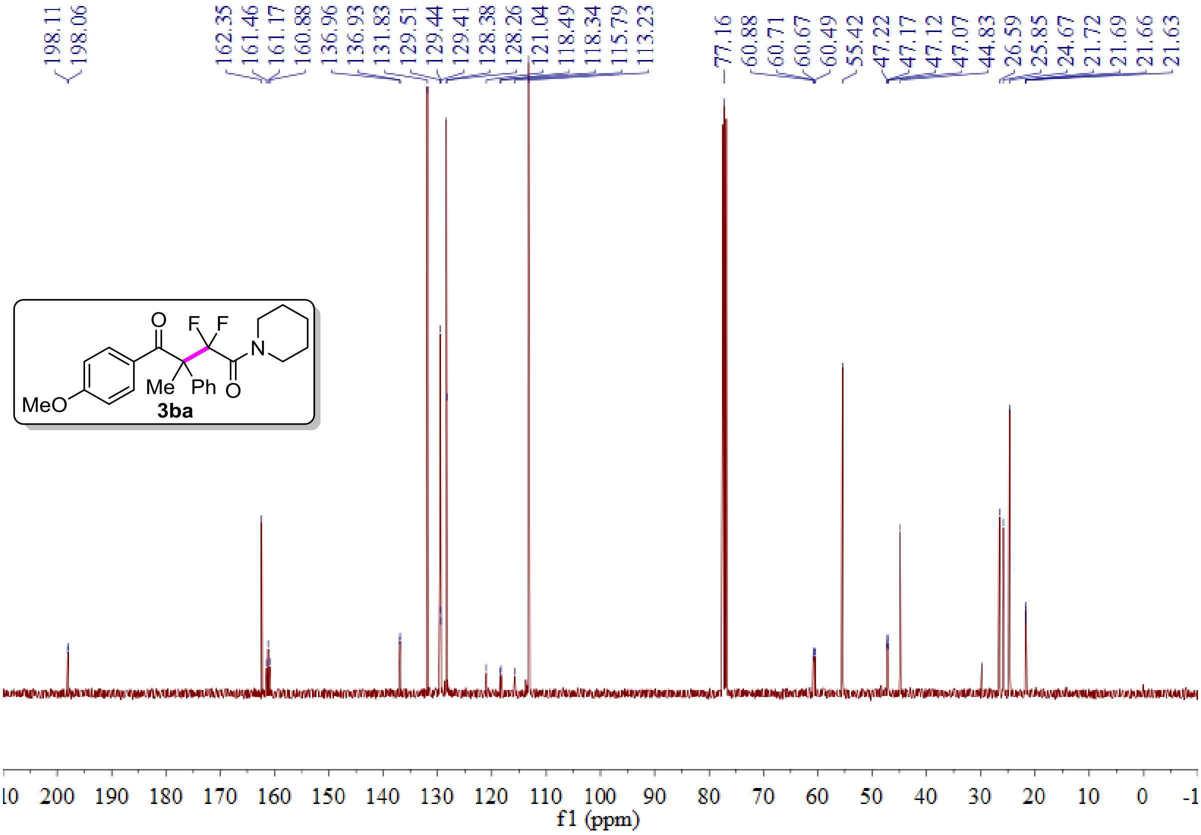


Supplementary Figure 158. $^{13}C$ NMR of 3ba

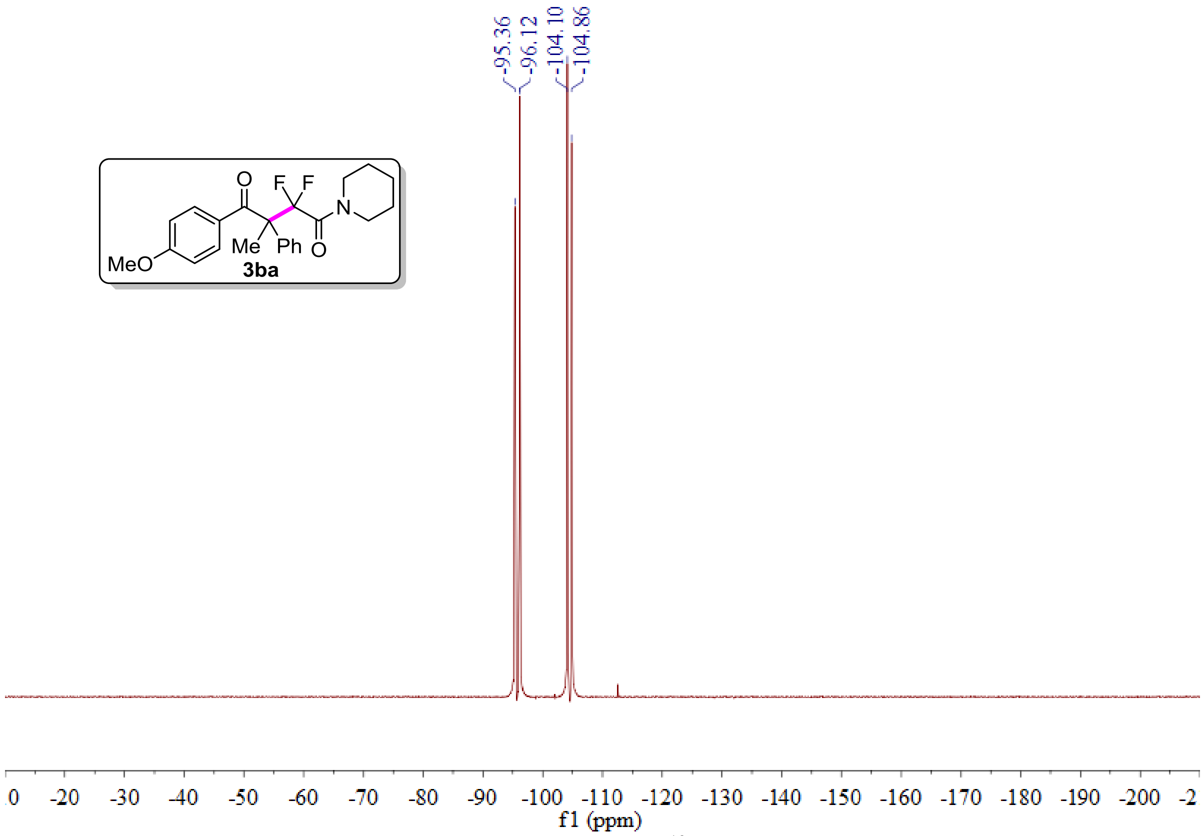


Supplementary Figure 159. $^{19}F$ NMR of 3ba

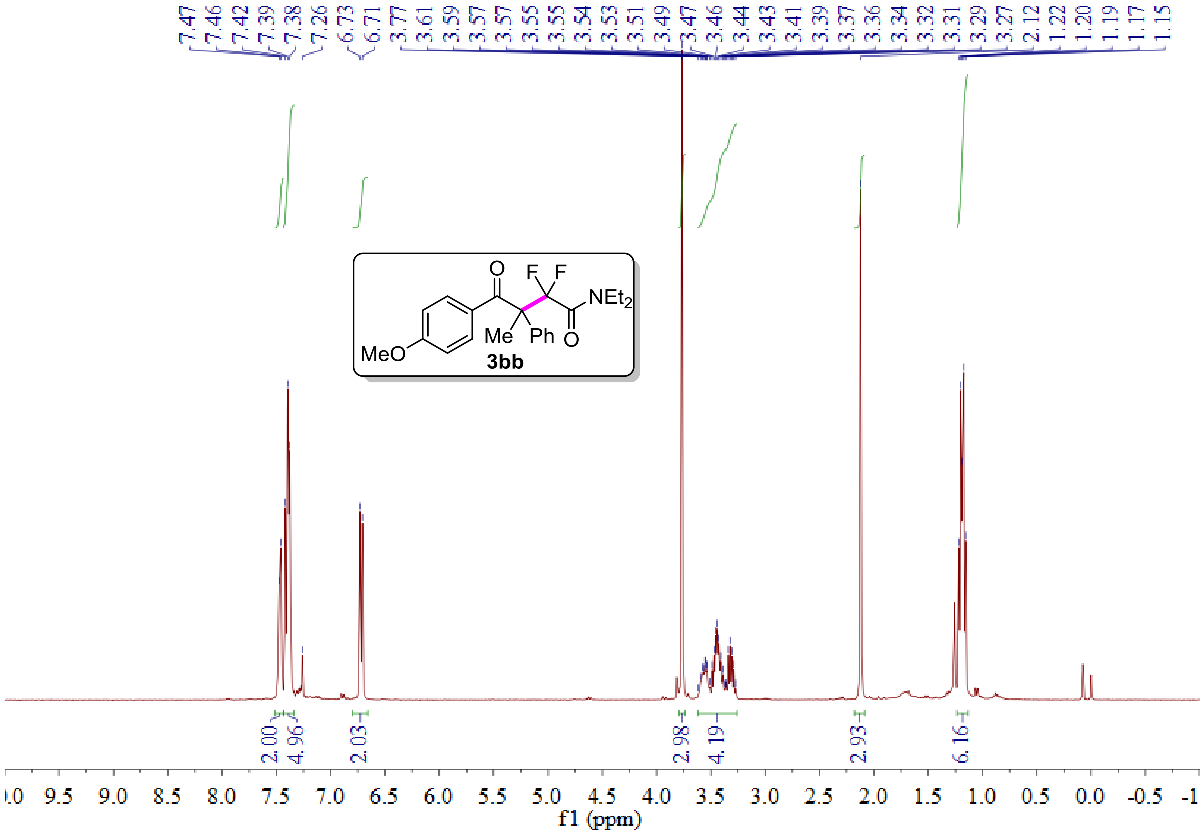


Supplementary Figure 160. $^{1}H$ NMR of 3bb

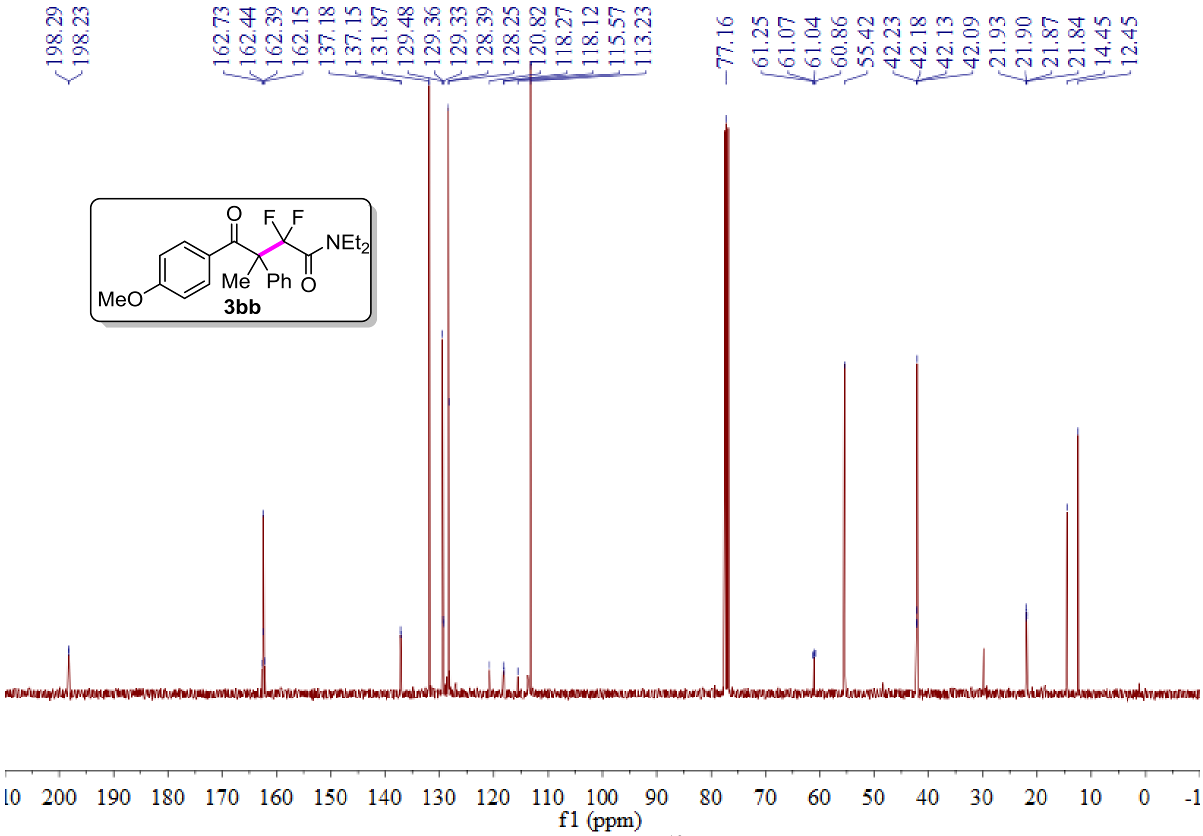


Supplementary Figure 161. $^{13}$C NMR of 3bb

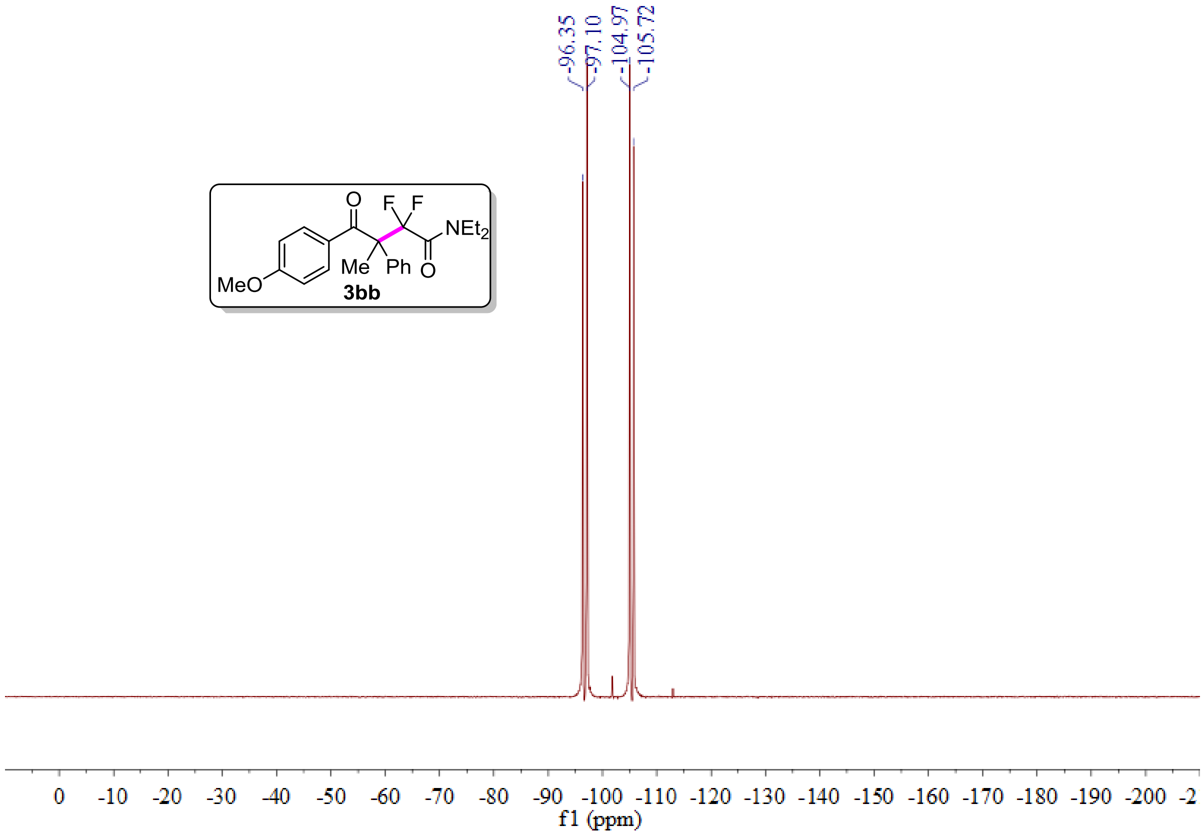


Supplementary Figure 162. $^{19}$F NMR of 3bb

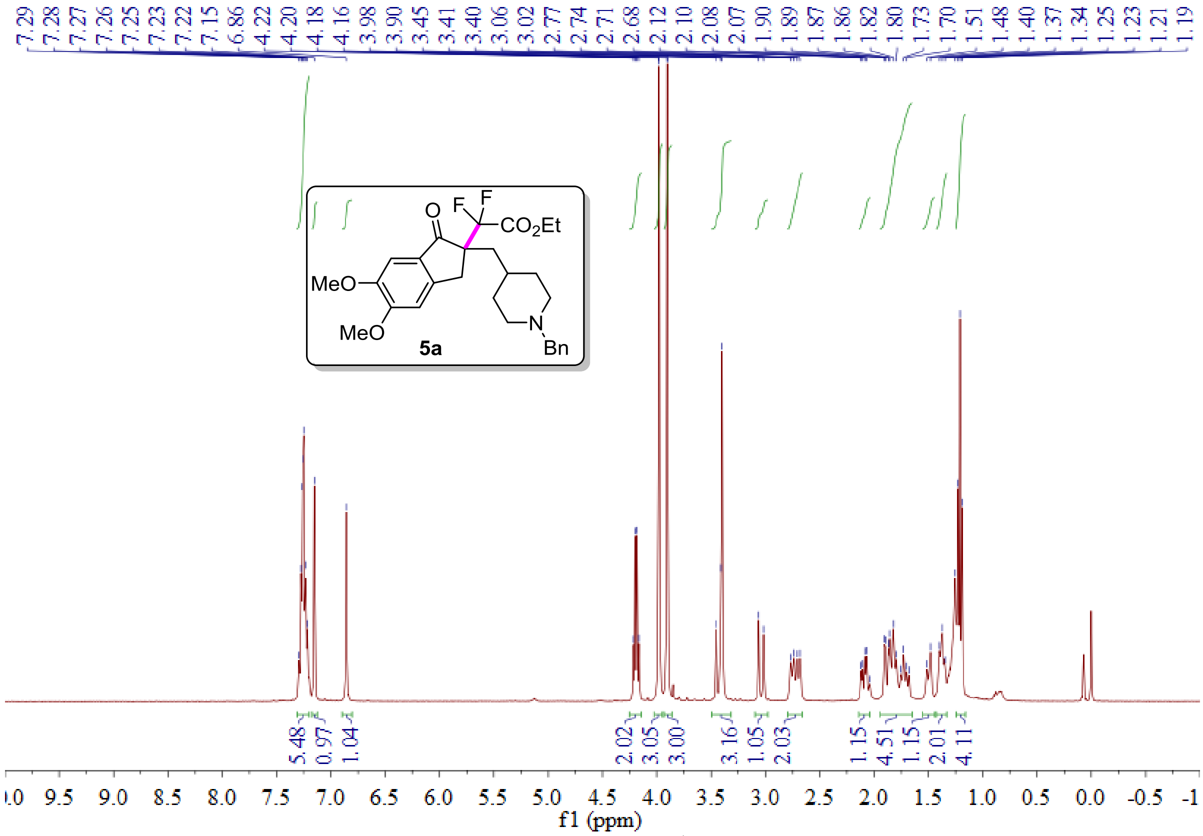

Supplementary Figure 163. $^{1}$H NMR of 5a

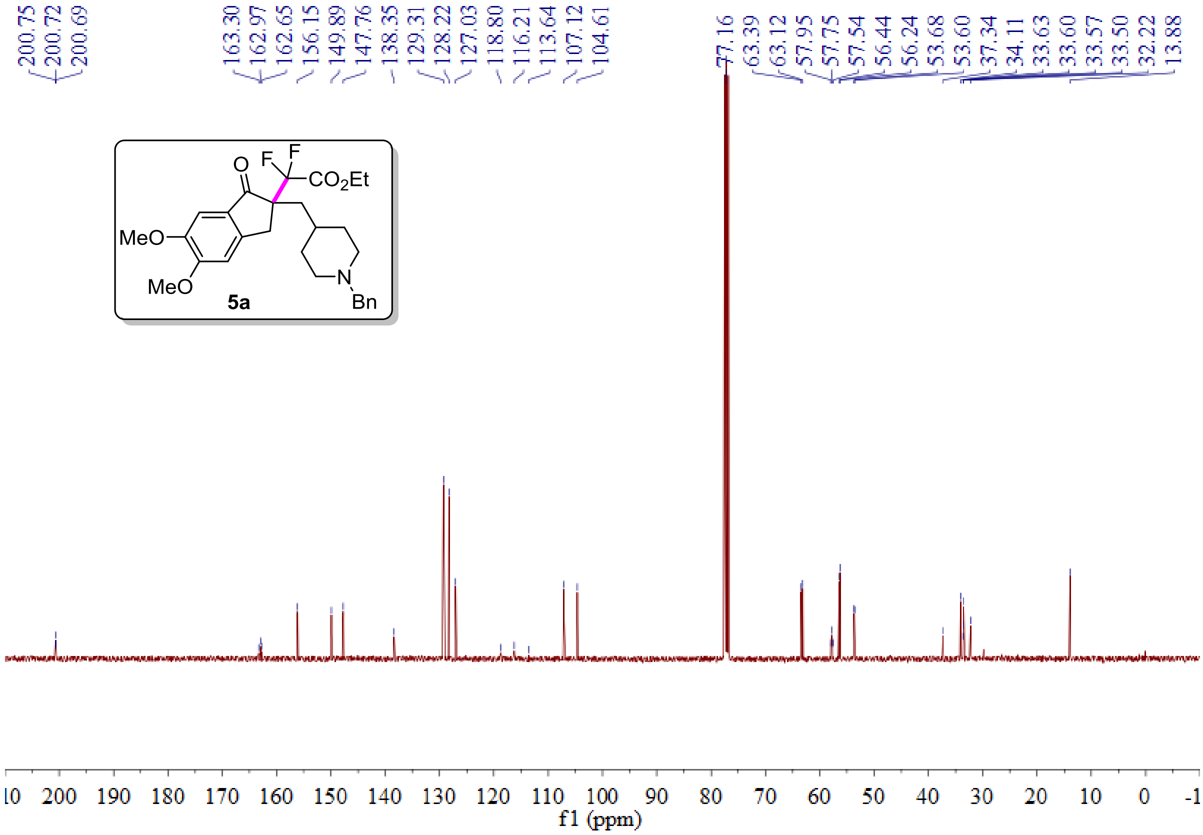

Supplementary Figure 164. $^{13}$C NMR of 5a

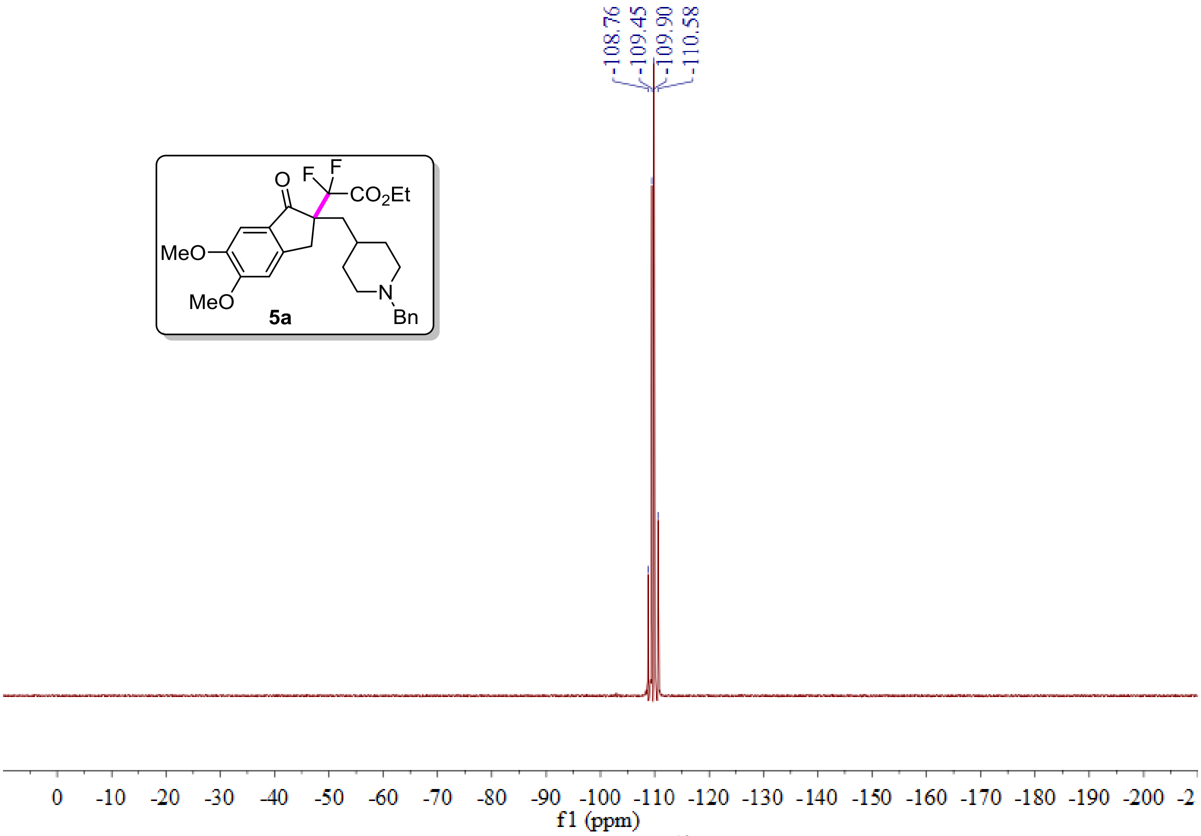

Supplementary Figure 165. $^{19}F$ NMR of 5a

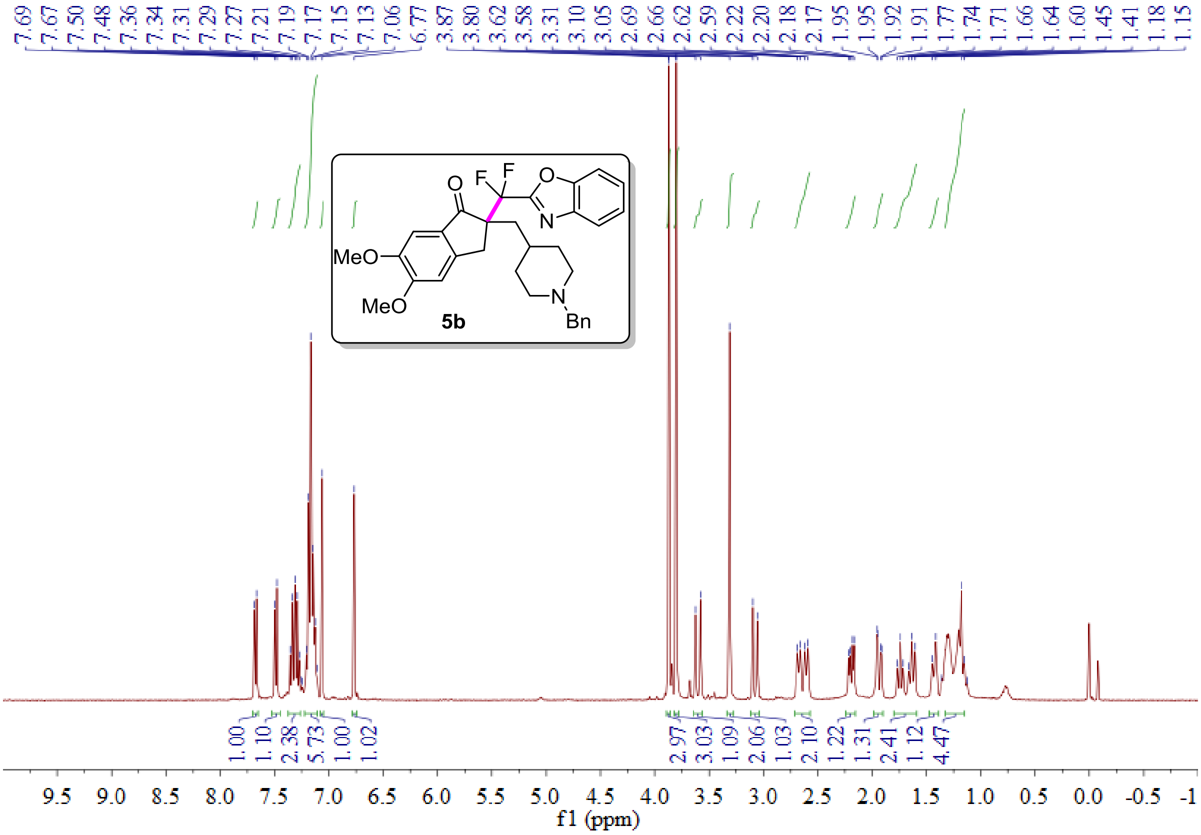

Supplementary Figure 166. $^{1}H$ NMR of 5b

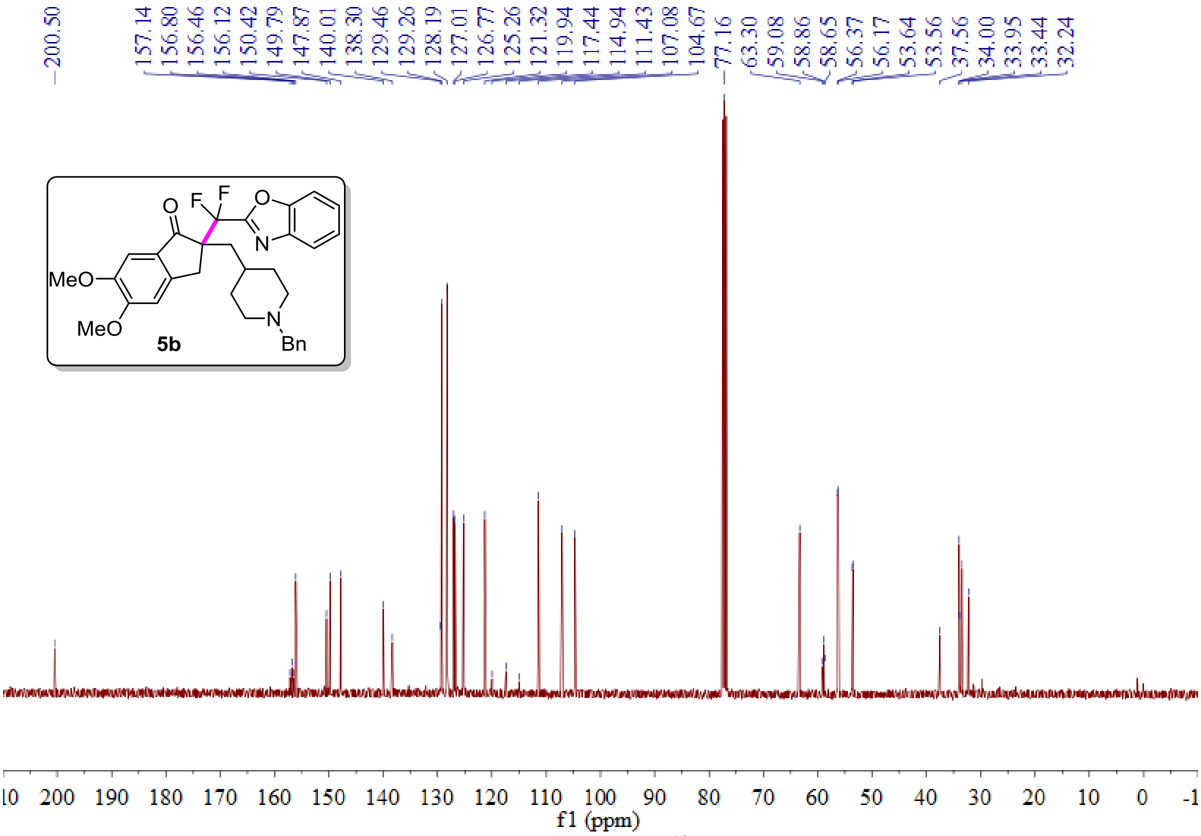


Supplementary Figure 167. $^{13}$C NMR of 5b

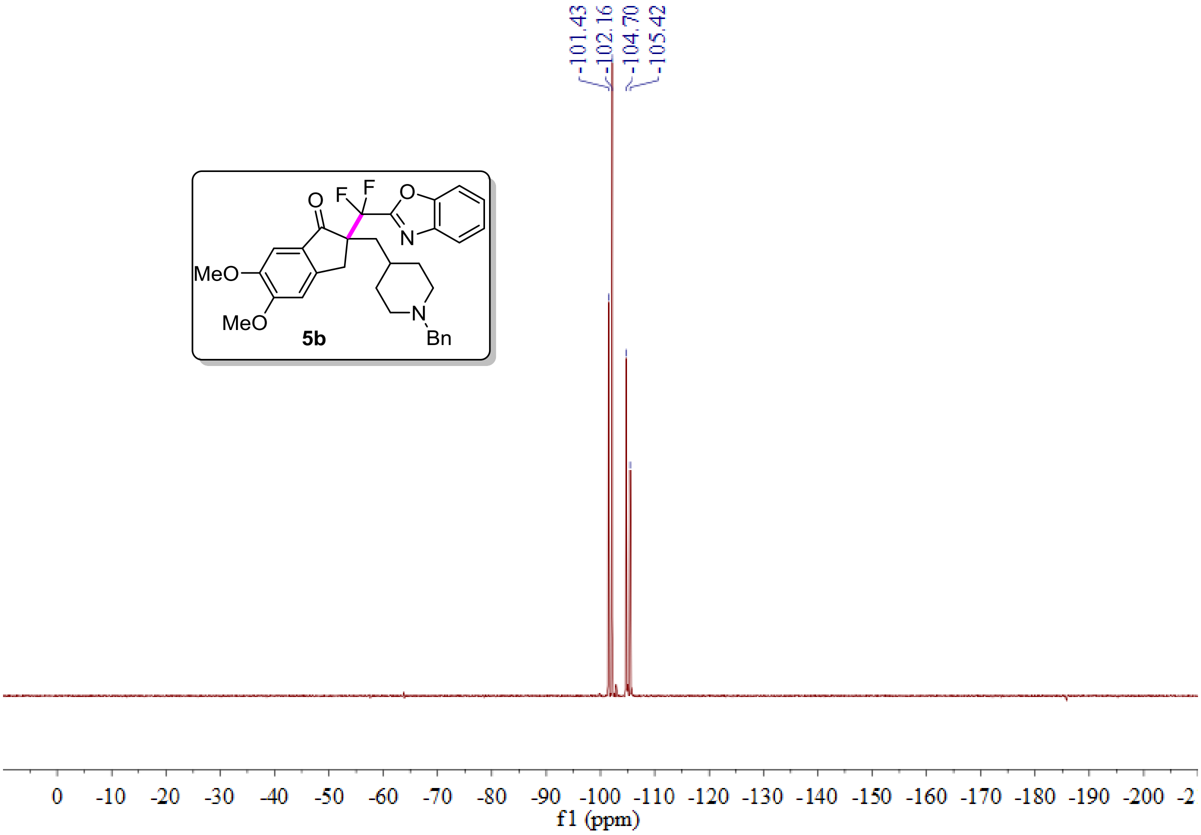


Supplementary Figure 168. $^{19}$F NMR of 5b

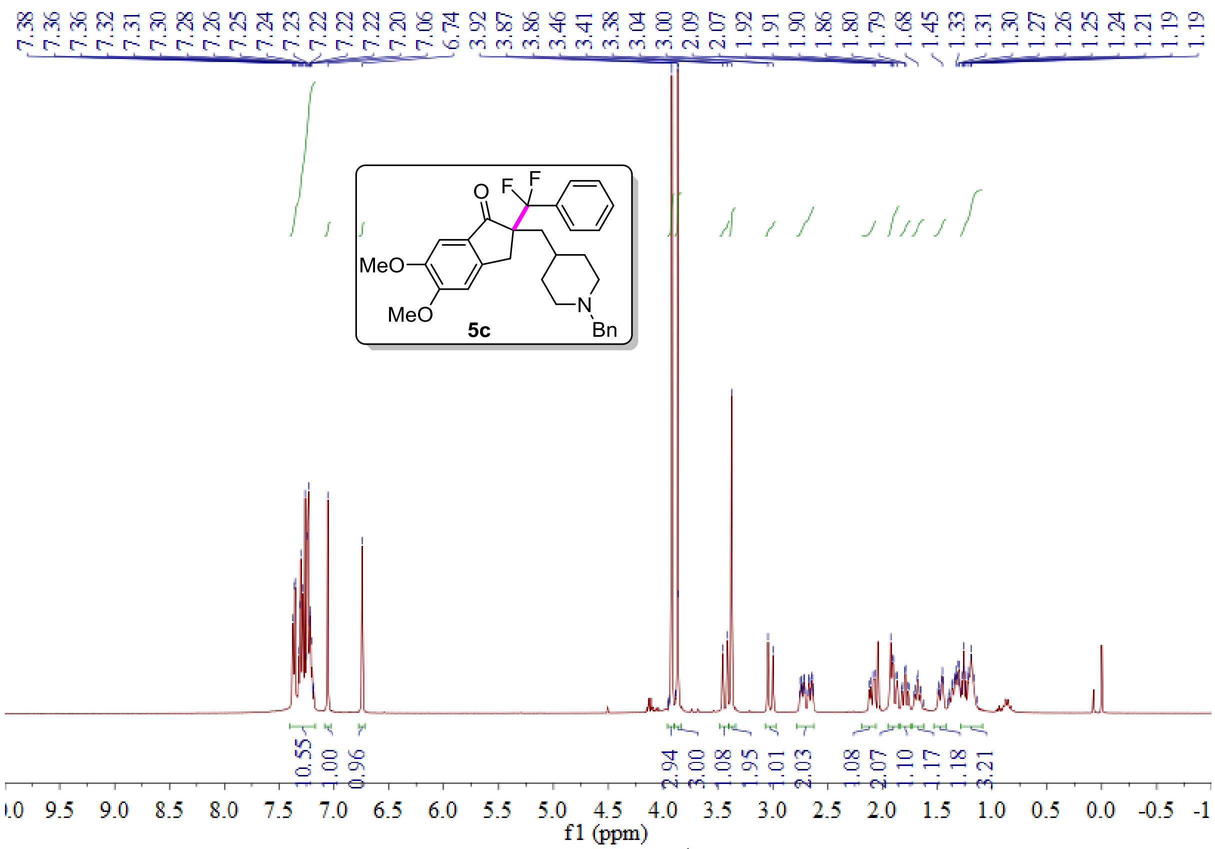


Supplementary Figure 169. $^{1}$H NMR of 5c

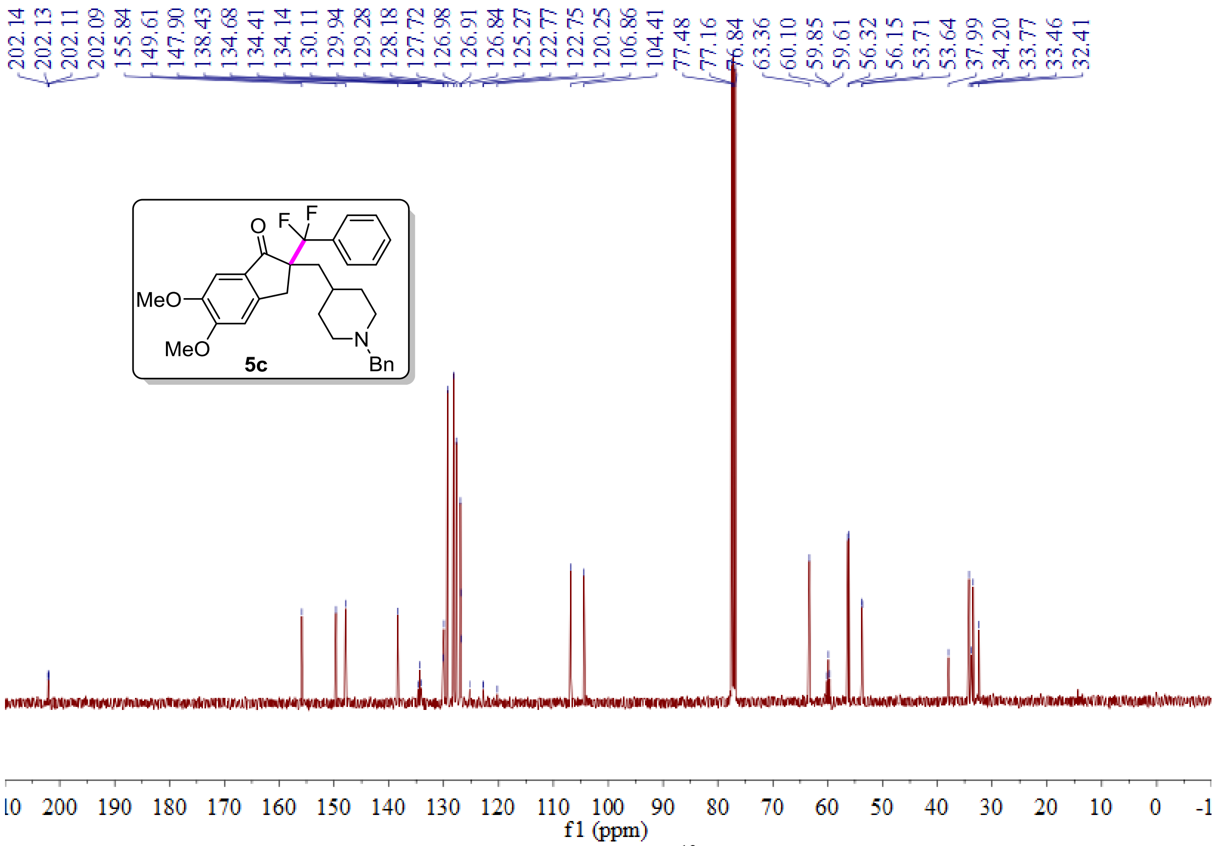


Supplementary Figure 170. $^{13}$C NMR of 5c

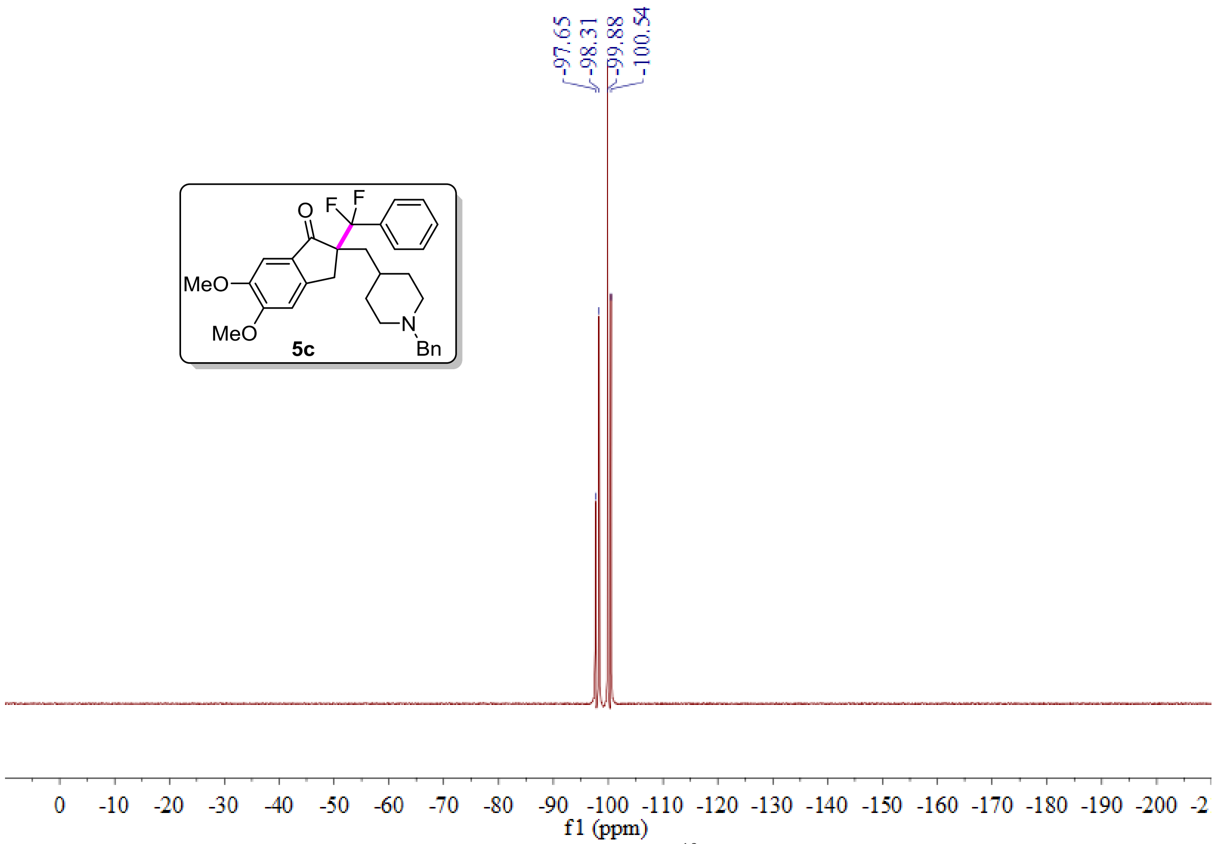

Supplementary Figure 171. $^{19}F$ NMR of 5c

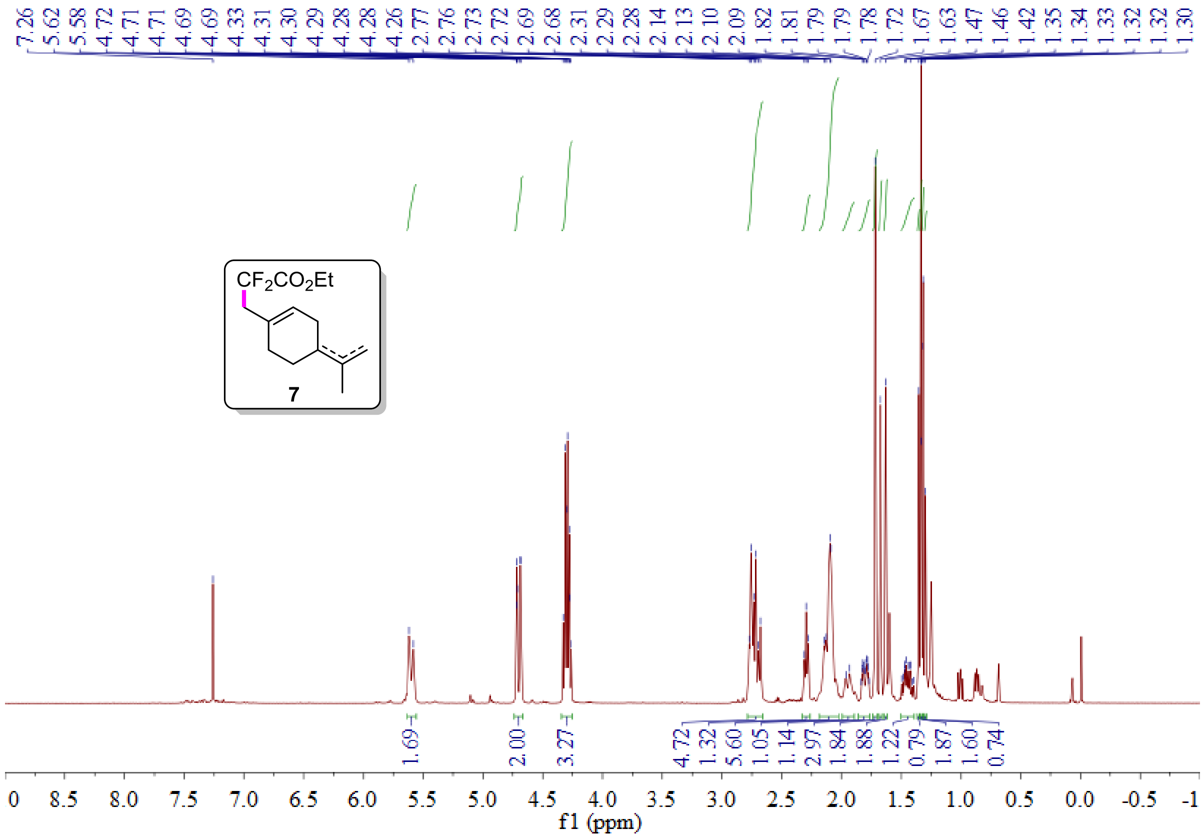

Supplementary Figure 172. $^{1}H$ NMR of 7

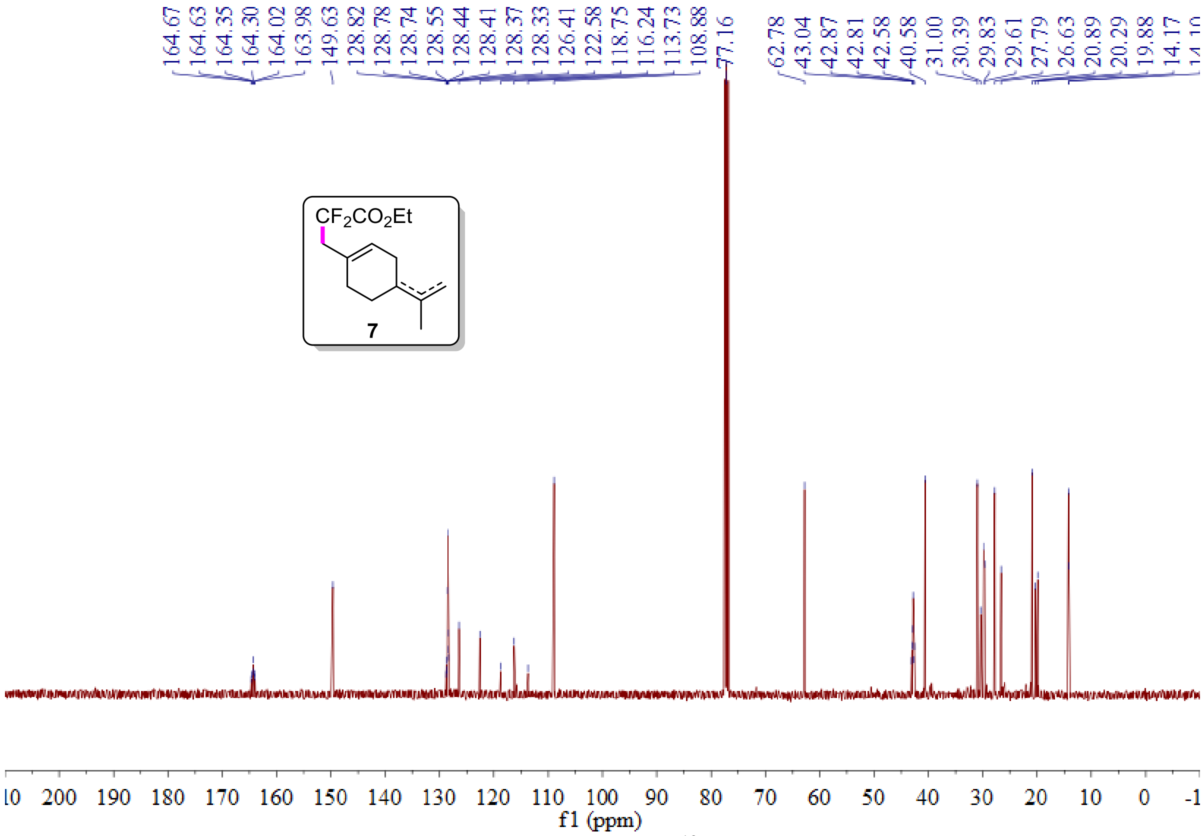


Supplementary Figure 173. $^{13}C$ NMR of 7

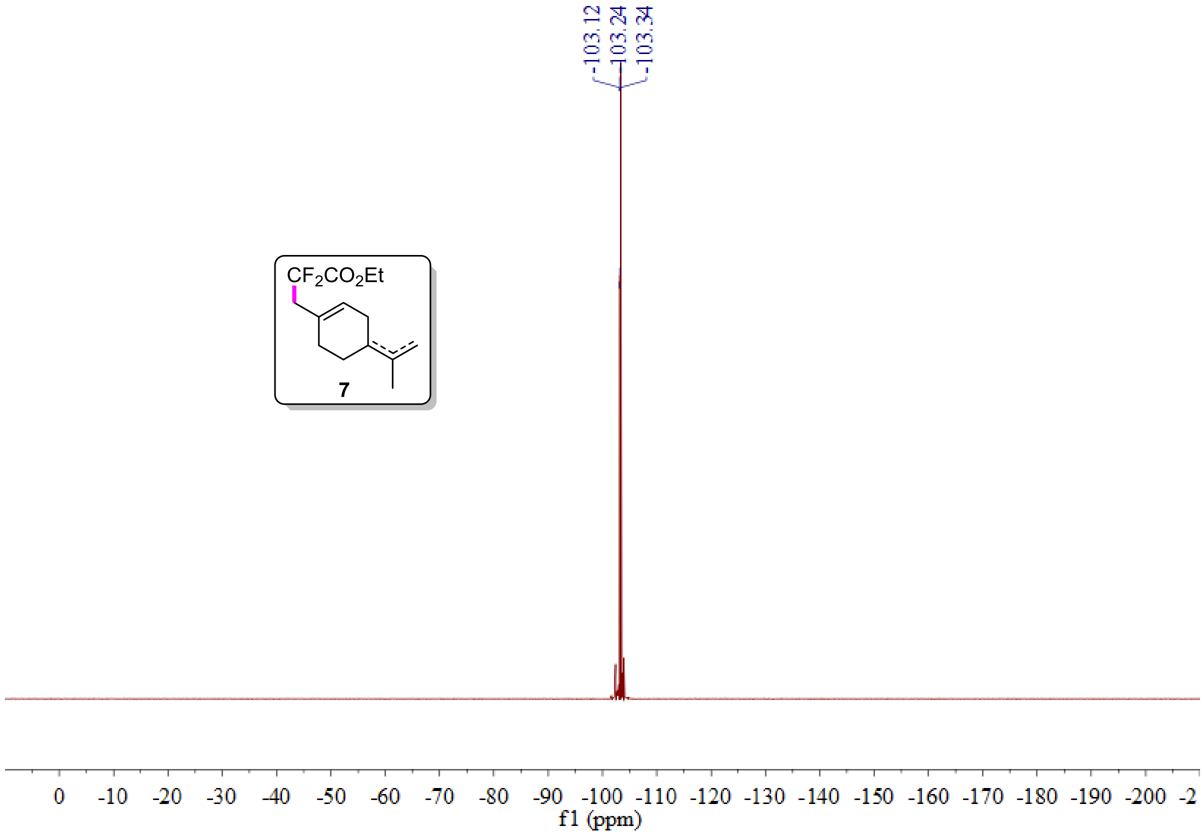


Supplementary Figure 174. $^{19}F$ NMR of 7

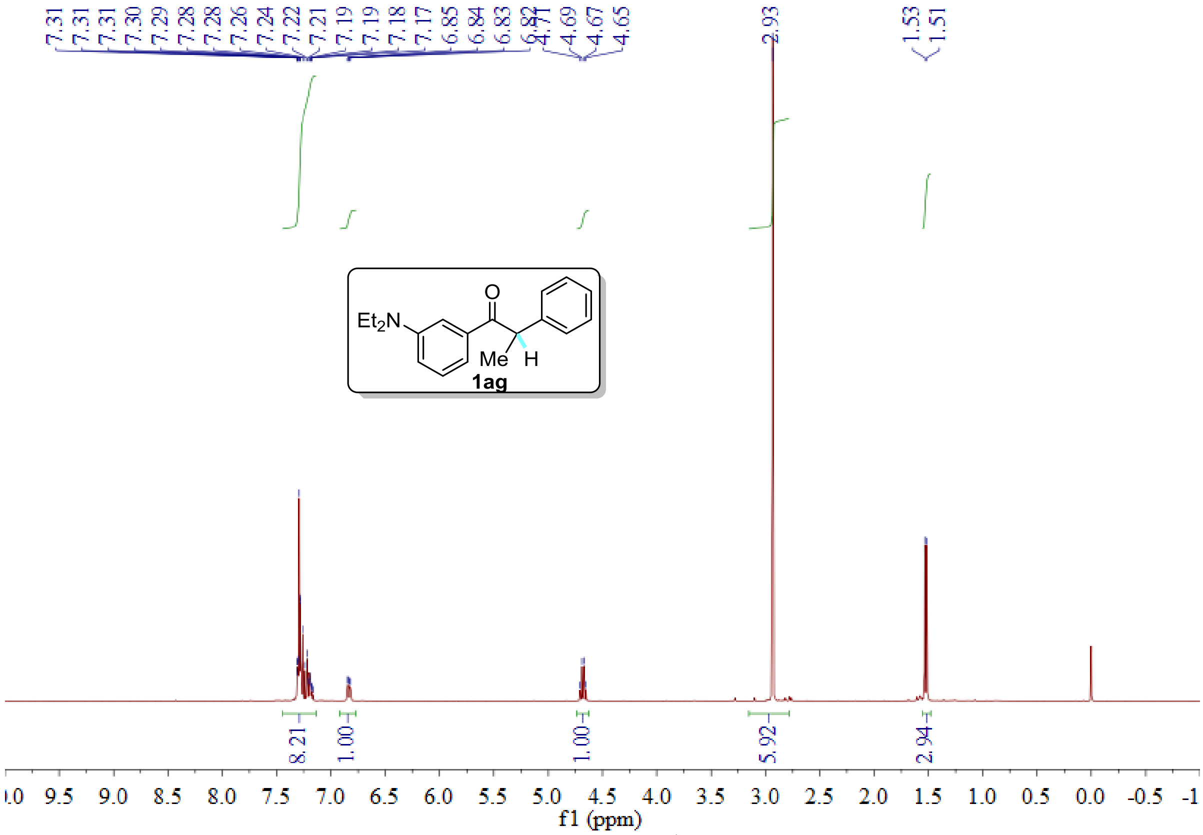


Supplementary Figure 175. $^{1}$H NMR of 1ag

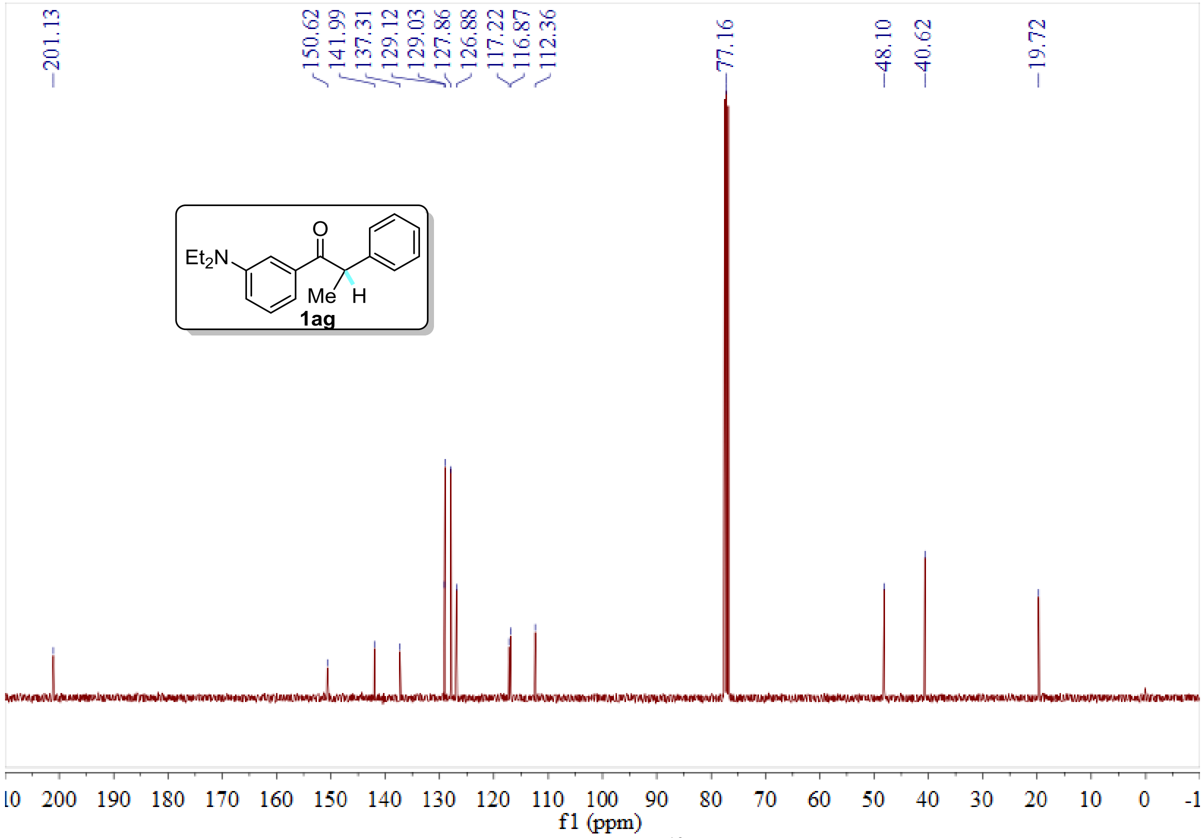


Supplementary Figure 176. $^{13}$C NMR of 1ag

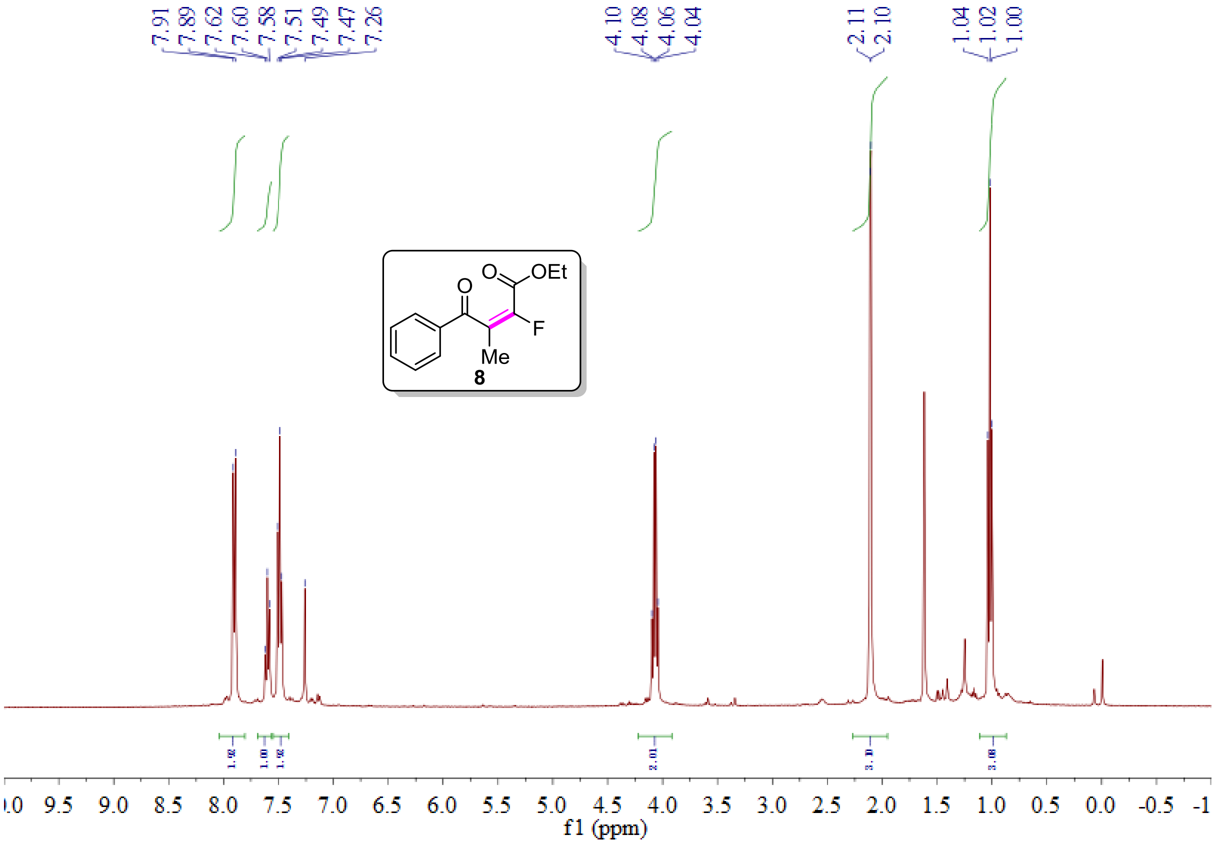


Supplementary Figure 177. $^{1}$H NMR of 8

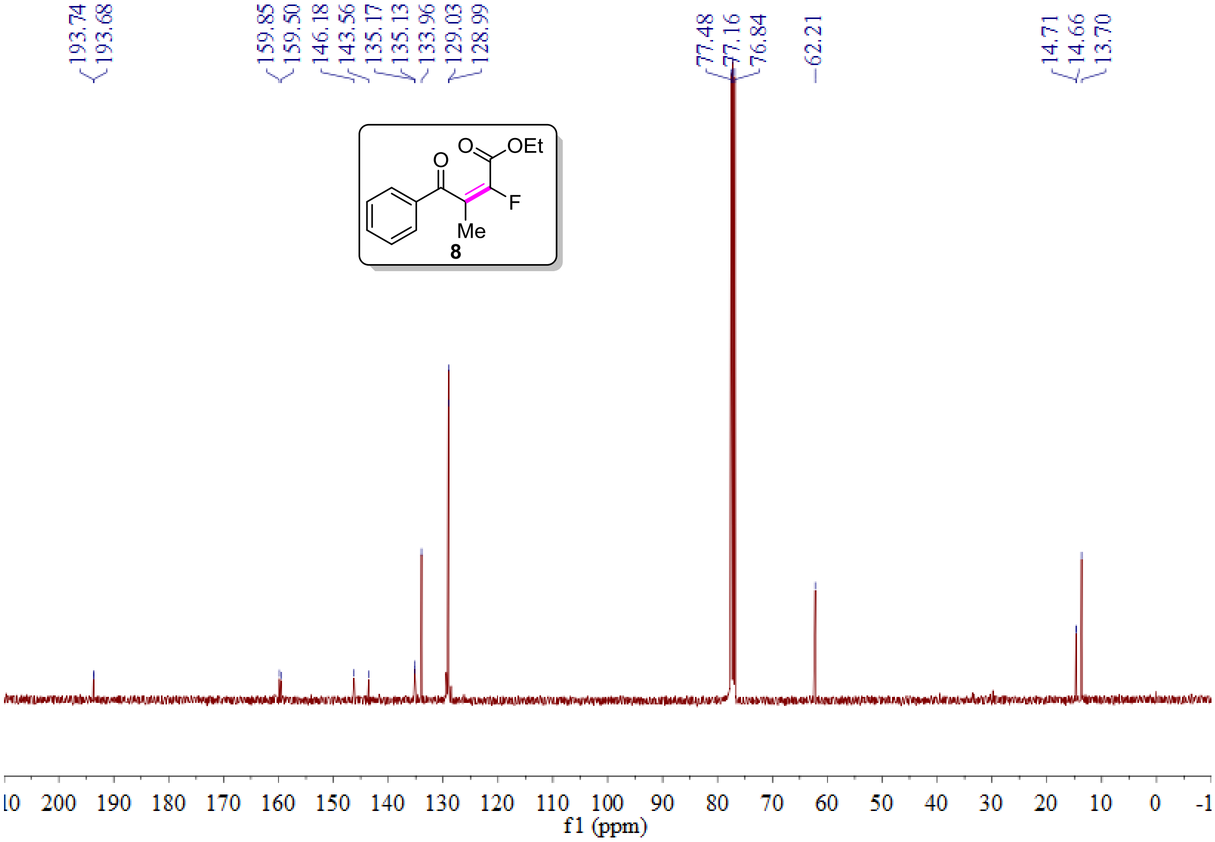


Supplementary Figure 178. $^{13}$C NMR of 8

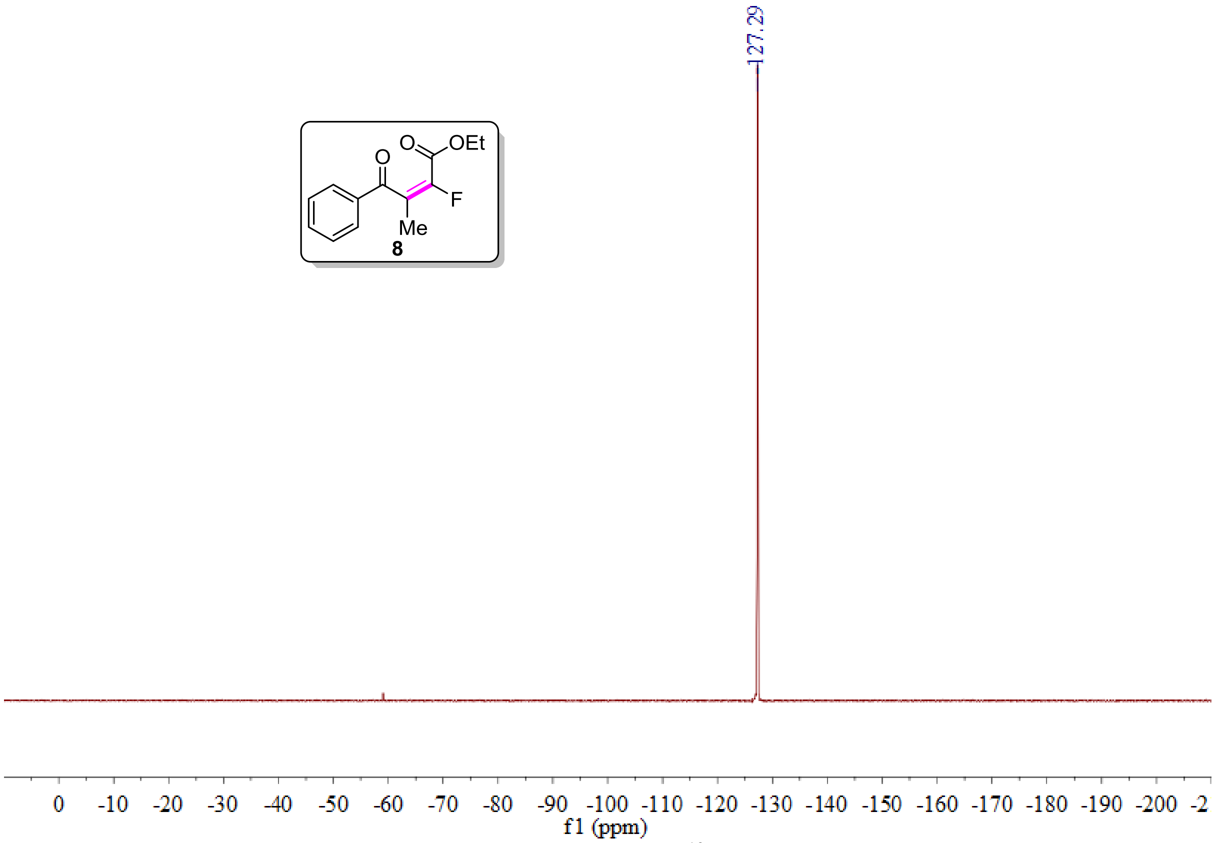


Supplementary Figure 179. $^{19}$F NMR of 8

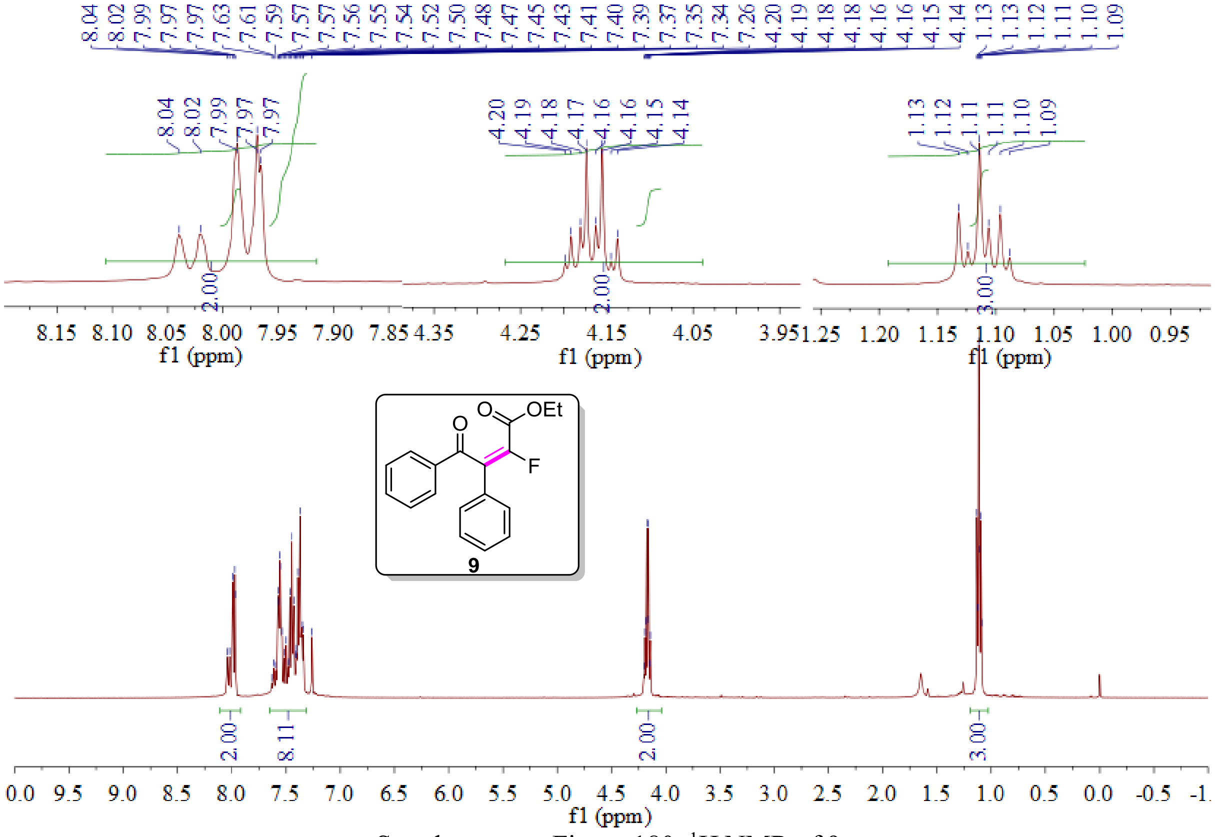


Supplementary Figure 180. $^{1}$H NMR of 9

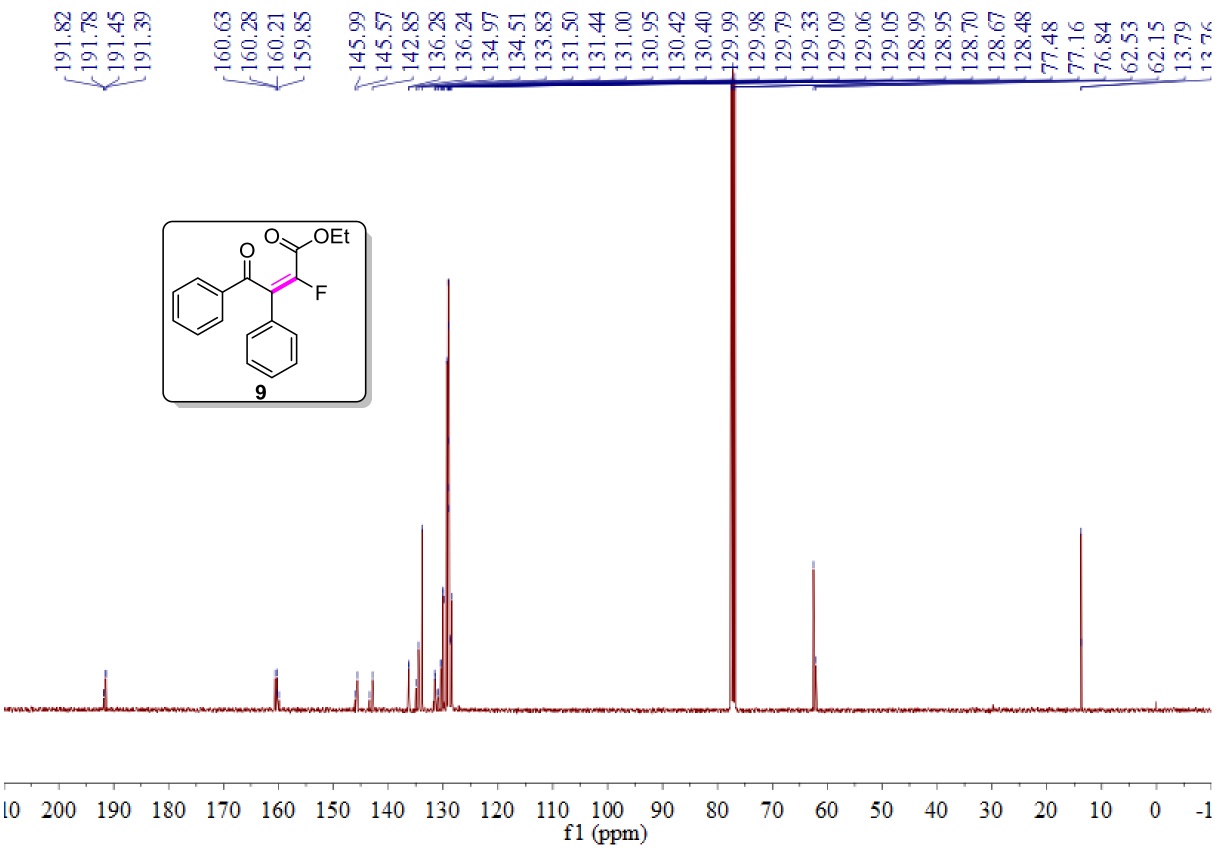

Supplementary Figure 181. $^{13}C$ NMR of 9

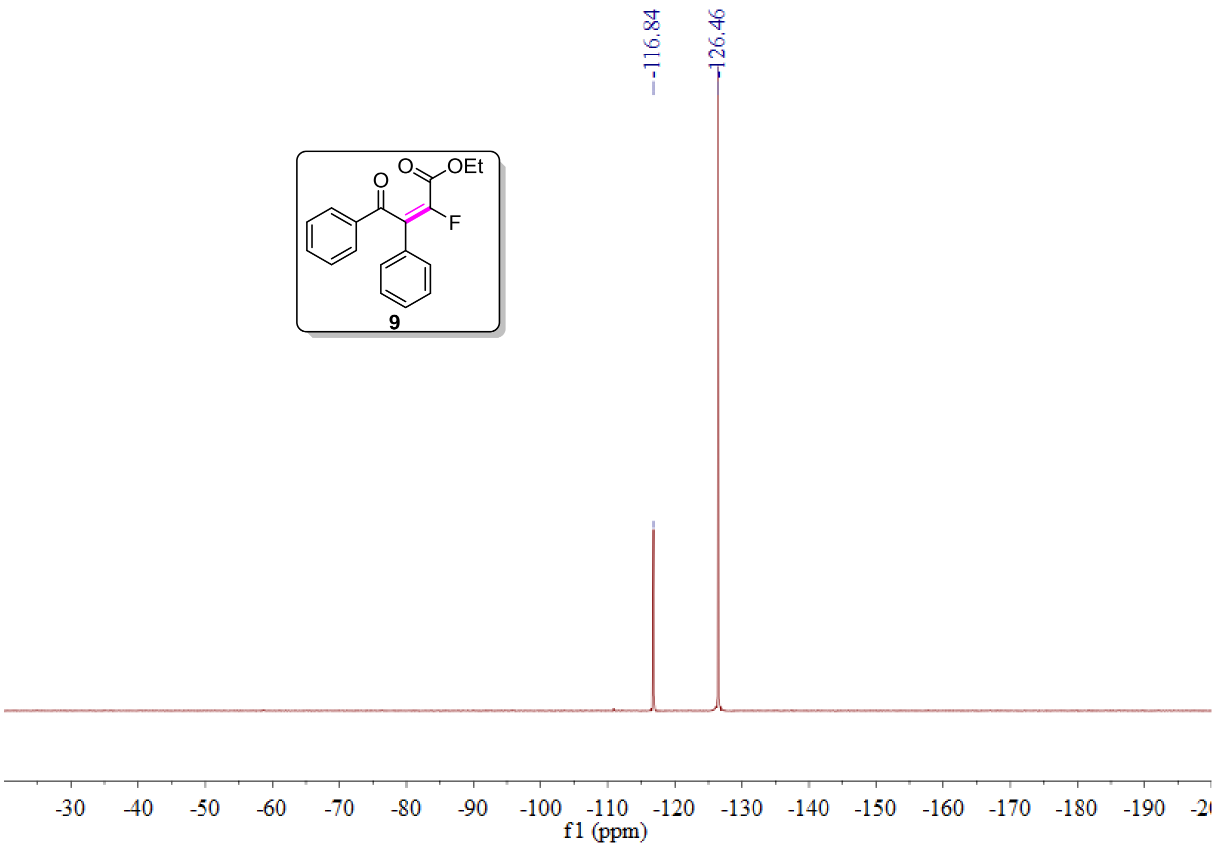

Supplementary Figure 182. $^{19}F$ NMR of 9

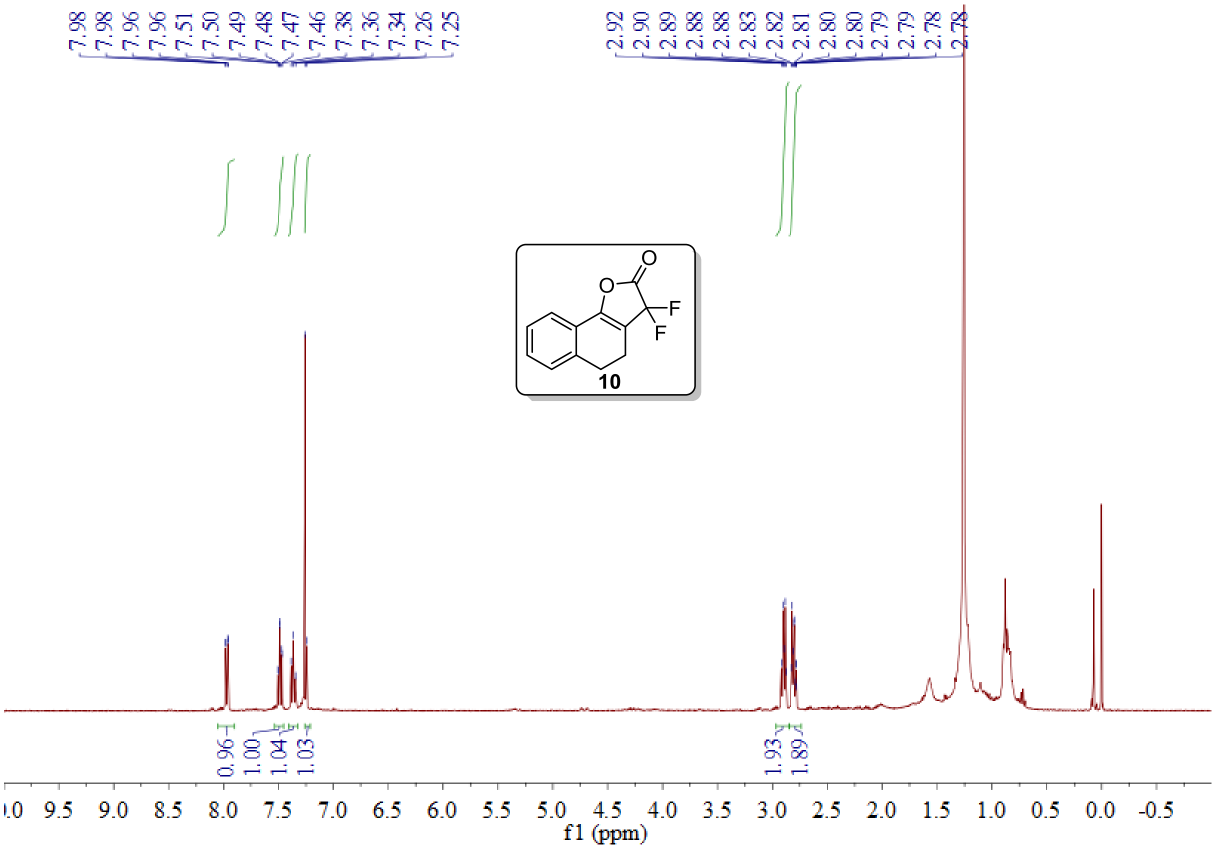


Supplementary Figure 183. $^{1}$H NMR of 10

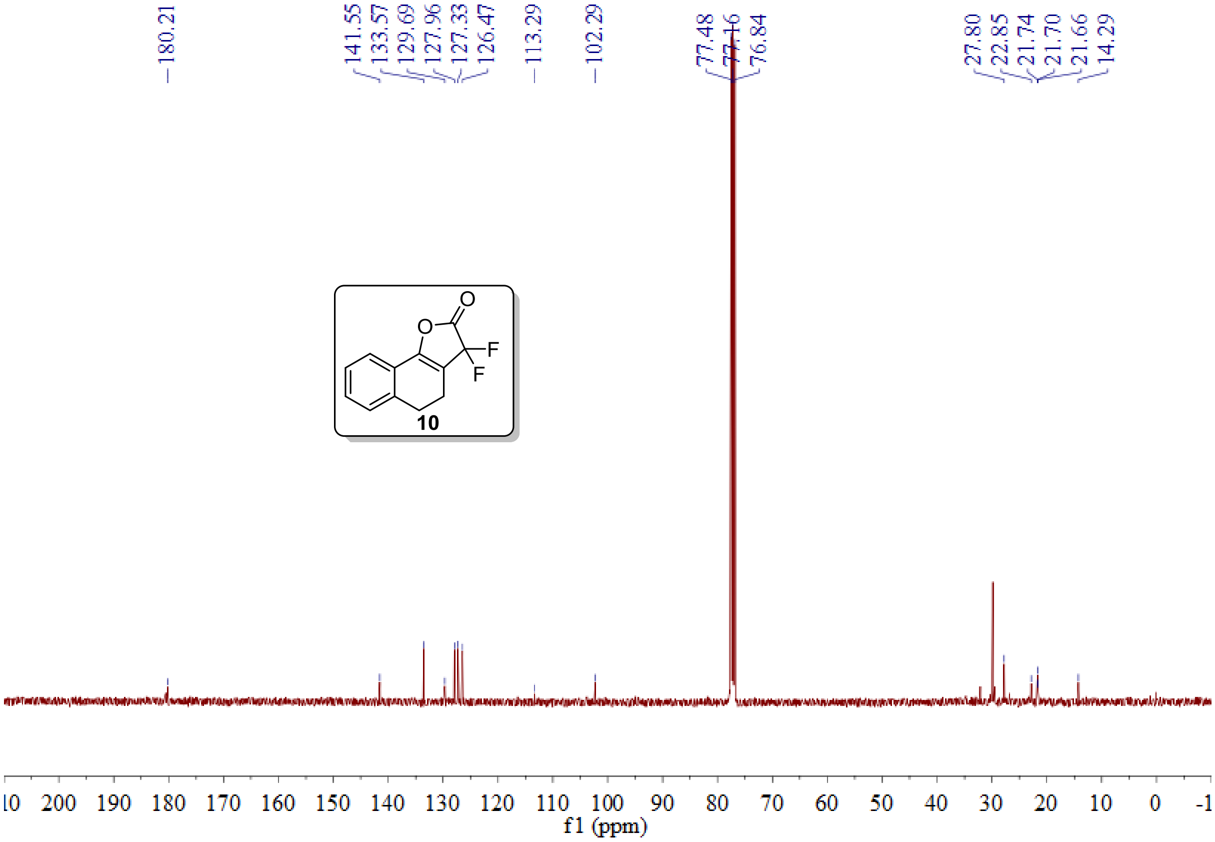


Supplementary Figure 184. $^{13}$C NMR of 10

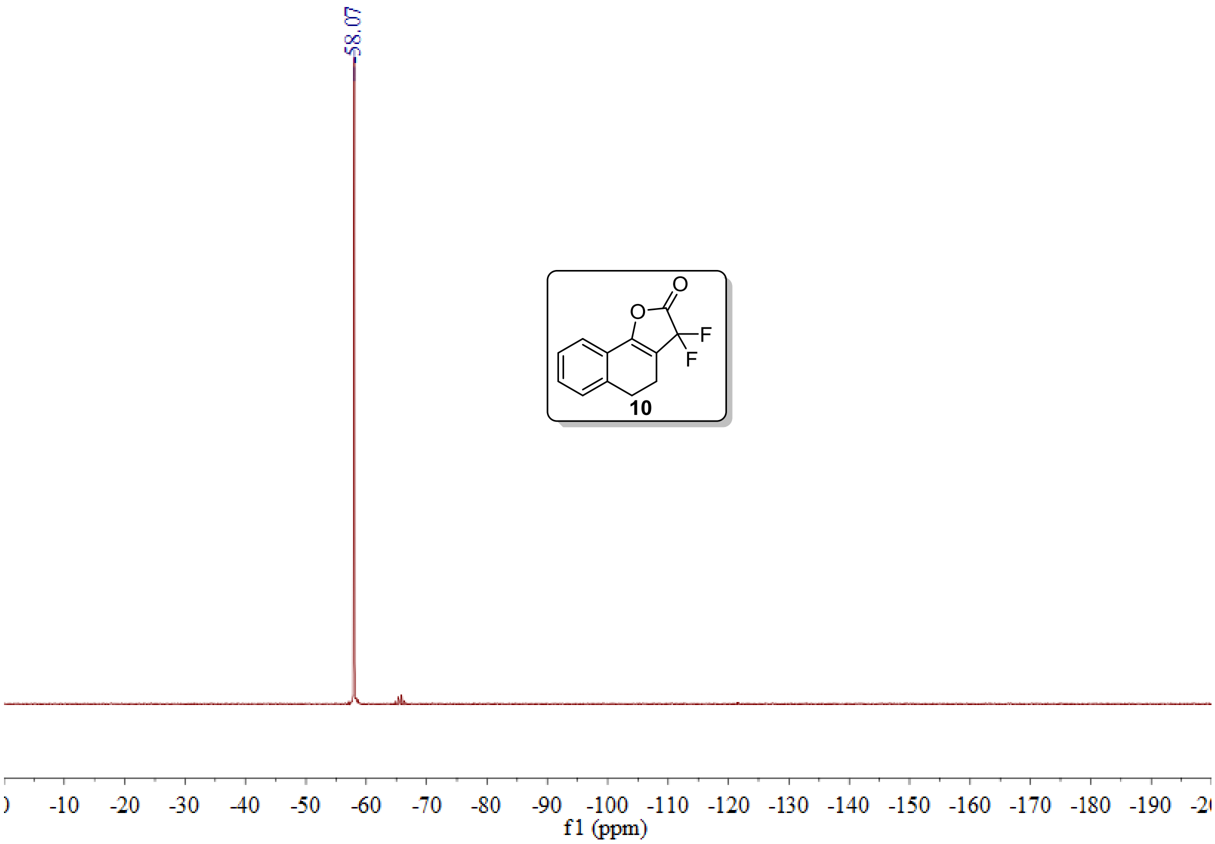

Supplementary Figure 185. $^{19}F$ NMR of 10

**Supplementary Table 1** Base Screening[a]

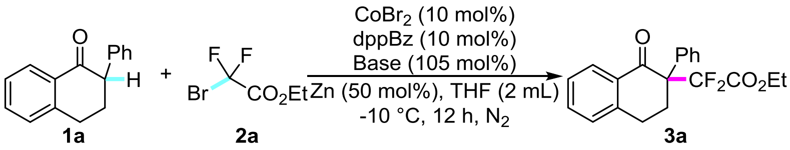


| Entry | Base | Yield (%)[b] |
|---|---|---|
| 1 | $K_2CO_3$ | trace |
| 2 | KOH | trace |
| 3 | $K_3PO_4$ | trace |
| 4[c] | $^t$BuOK | trace |
| 5[d] | $^t$BuOK | 17 |
| 6[e] | $^t$BuOK | 30 |
| 7[f] | $^t$BuOK | trace |
| 8 | KHMDS | 73 |
| 9 | LiHMDS | 77 |
| 10 | LDA | 90 |

[a] General conditions: **1a** (0.2 mmol), **2a** (3 eq.), $CoBr_2$ (10 mol%), dppBz (10 mol%), Base (105 mol%), Zn (50 mol%), THF (2 mL), -10 °C, 12 h, $N_2$. [b] Isolated Yield. [c] No Zn. [d] Zn (30 mol%). [e] Zn (50 mol%). [f] Mn (50 mol%).

**Supplementary Table 2** Catalyst Screening[a]

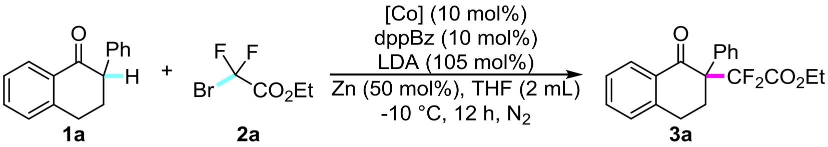


| Entry | [Co] | Yield (%)[b] |
|---|---|---|
| 1 | $Co(acac)_2$ | 42 |
| 2 | $CoCl_2$ | 61 |
| 3 | $Co(OAc)_2{\cdot}4H_2O$ | 30 |
| 4 | $CoI_2$ | 83 |
| 5 | $CoC_2O_4$ | 10 |
| 6 | $CoCl_2{\cdot}$dppe | 56 |
| 7 | / | trace |

[a] General conditions: **1a** (0.2 mmol), **2a** (3 eq.), [Co] (10 mol%), dppBz (10 mol%), LDA (105 mol%), Zn (50 mol%), THF (2 mL), -10 °C, 12 h, $N_2$. [b] Isolated Yields.

## **Supplementary Table 3** Ligand Screening[a]

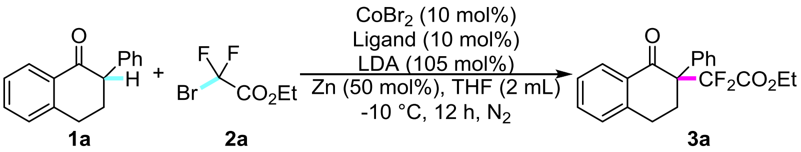


| Entry | Ligand | Yield (%)[b] | Entry | Ligand | Yield (%)[b] |
|---|---|---|---|---|---|
| 1 | L1 | trace | 14 | L13 | trace |
| 2 | L2 | trace | 15 | L14 | 50 |
| 3 | L3 | trace | 16 | XantPhos | 20 |
| 4 | dppe | 30 | 17 | L15 | 10 |
| 5 | L4 | 48 | 18 | dppp | 20 |
| 6 | L5 | trace | 19 | dppBz | 90 |
| 7 | L6 | trace | 20 | L16 | 15 |
| 8 | L7 | trace | 21 | L17 | 14 |
| 9 | L8 | trace | 22 | Phen | 20 |
| 10 | L9 | trace | 23 | L18 | trace |
| 11 | L10 | 5 | 24 | bpy | 23 |
| 12 | L11 | trace | 25 | L19 | trace |
| 13 | L12 | trace | | | |

[a] General conditions: **1a** (0.2 mmol), **2a** (3 eq.), $CoBr_2$ (10 mol%), Ligand (10 mol%), LDA (105 mol%), Zn (50 mol%), THF (2 mL), -10 °C, 12 h, $N_2$. [b] Isolated Yield.

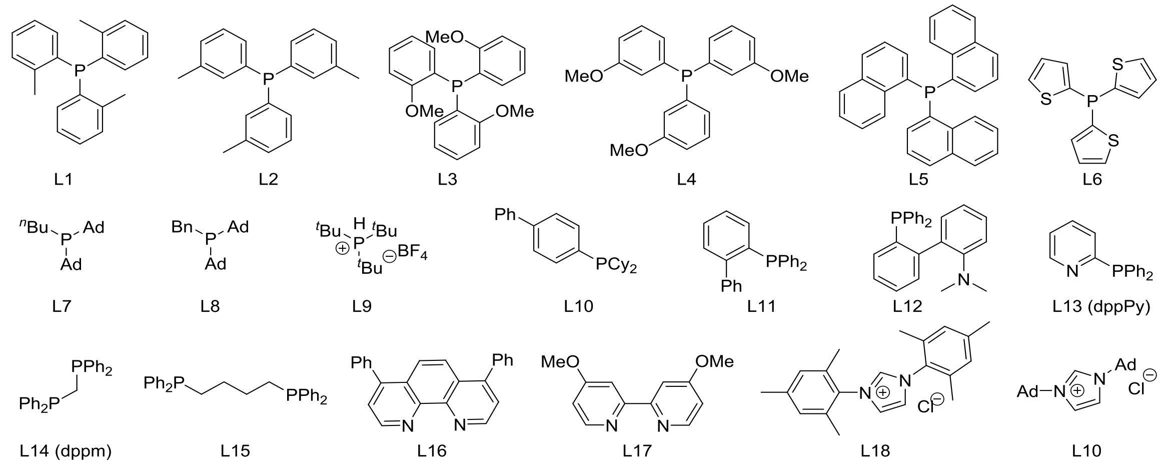


## **Supplementary Table 4** Loading of Fluoroalkylating Reagents Screening[a]

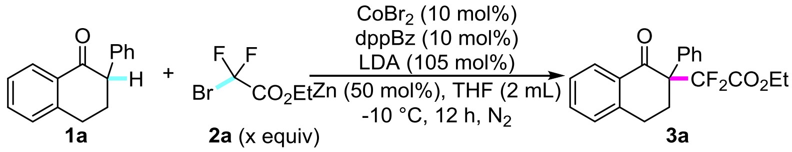


| Entry | x | Yield (%)[b] |
|---|---|---|
| 1 | 1.2 | 51 |
| 2 | 1.5 | 58 |
| 3 | 2.0 | 83 |
| 4 | 2.5 | 84 |
| 5 | 4 | 73 |
| 6 | 5 | 70 |

[a] General conditions: **1a** (0.2 mmol), **2a** (x eq.), $CoBr_2$ (10 mol%), dppBz (10 mol%), LDA (105 mol%), Zn (50 mol%), THF (2 mL), -10 °C, 12 h, $N_2$. [b] Isolated Yields.

**Supplementary Table 5** Temperature Screening[a]

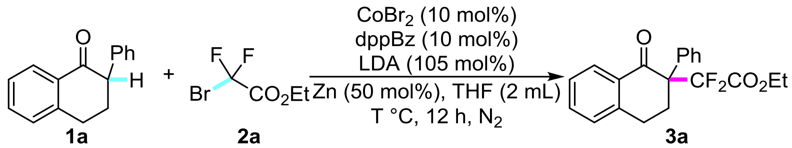


| Entry | T/°C | yield (%)[b] |
|---|---|---|
| 1 | -30 | 90 |
| 2[c] | -10 | 90 |
| 3 | 0 | 53 |
| 4 | 50 | 30 |

[a] General conditions: **1a** (0.2 mmol), **2a** (3 eq.), $CoBr_2$ (10 mol%), dppBz (10 mol%), LDA (105 mol%), Zn (50 mol%), THF (2 mL), T °C, 12 h, $N_2$. [b] Isolated Yields. [c] t = 24 h.

**Supplementary Table 6** Solvent Screening[a]

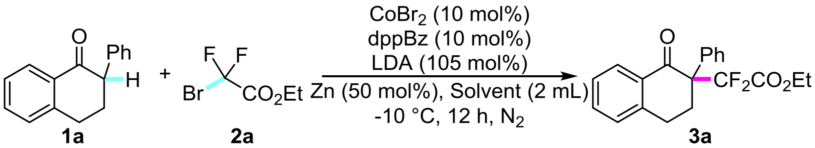


| Entry | Solvent | Yield (%)[b] |
|---|---|---|
| 1 | Toluene | trace |
| 2 | MeCN | 0 |
| 3 | DMF | 0 |
| 4 | NMP | 0 |
| 5 | Dioxane | 50 |

[a] General conditions: **1a** (0.2 mmol), **2a** (3 eq.), $CoBr_2$ (10 mol%), dppBz (10 mol%), LDA (105 mol%), Zn (50 mol%), Solvent (2 mL), -10 °C, 12 h, $N_2$. [b] Isolated Yields.

**Supplementary Figure 186** Aryl Ketones Containing Secondary α-C-H Bonds Used as Substrates

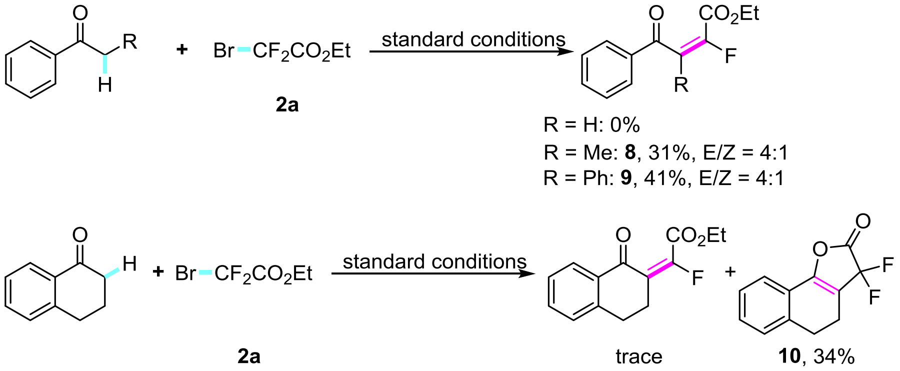


# Supplementary Methods

**General Information** NMR spectra were recorded on Bruker-400 MHz NMR spectrometer (400 MHz for $^1$H; 101 MHz for $^{13}$C and 376 MHz for $^{19}$F ($^1$H, $^{13}$C decoupled). $^1$H NMR chemical shifts were determined relative to internal $(CH_3)_4Si$ (TMS) at δ 0.00 or at the signal of a residual protonated solvent: $CDCl_3$ δ 7.26. $^{13}$C NMR chemical shifts were determined relative to $CDCl_3$ δ 77.16. $^{19}$F NMR chemical shifts were determined relative to $CFCl_3$ at δ 0.00. Data for $^1$H, $^{13}$C, $^{19}$F NMR are recorded as follows: chemical shift (δ, ppm), multiplicity (s = singlet, d = doublet, t = triplet, m = multiplet, q = quartet, br =

broad). High resolution mass spectra were recorded on P-SIMS-Gly of BrukerDaltonics Inc. using ESI-TOF (electrospray ionization-time of flight) or Micromass GCT using EI (electron impact).

**Materials** THF was distilled from sodium immediately before use. LDA was obtained from Energy-chemical used as received (2 mol/L). $CoBr_2$ was obtained from Energy-chemical and used as received. dppBz and zinc powder were obtained from aladdin and used as received. $BrCF_2CO_2Et$ was obtained from Fluorochem Ltd (UK) and used as received.

**Preparation of Substituted Aryl Ketones**

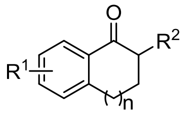


| Entry | | Entry | |
|---|---|---|---|
| 1a | $R^1$ = H, $R^2$ = Ph, n = 1 | 1j | $R^1$ = H, $R^2$ = Ph-Cl-3, n = 1 |
| 1b | $R^1$ = H, $R^2$ = Me, n = 1 | 1k | $R^1$ = 5-OMe, $R^2$ = Me, n = 1 |
| 1c | $R^1$ = H, $R^2$ = $^n$Bu, n = 1 | 1l | $R^1$ = 7-OMe, $R^2$ = Me, n = 1 |
| 1d | $R^1$ = H, $R^2$ = Bn, n = 1 | 1m | $R^1$ = 6-OMe, $R^2$ = Me, n = 1 |
| 1e | $R^1$ = H, $R^2$ = $^i$Pr, n = 1 | 1n | $R^1$ = 7-Br, $R^2$ = Me, n = 1 |
| 1f | $R^1$ = H, $R^2$ = Ph-Me-4, n = 1 | 1o | $R^1$ = H, $R^2$ = Ph, n = O |
| 1g | $R^1$ = H, $R^2$ = Ph-Cl-4, n = 1 | 1p | $R^1$ = H, $R^2$ = Me, n = 0 |
| 1h | $R^1$ = H, $R^2$ = Ph-Br-4, n = 1 | 1q | $R^1$ = 5-Me, $R^2$ = Me, n = 0 |
| 1i | $R^1$ = H, $R^2$ = Ph-F-4, n = 1 | 1r | $R^1$ = 5-Cl, $R^2$ = Me, n = 0 |

Substrates **1a**[4], **1b**[5], **1c**[6], **1f-1i**[6], **1k**[6], **1d**[7], **1e**[7], **1j**[7], **1l**[7], **1m-1o**[7] were prepared in accordance with methods described in the references.

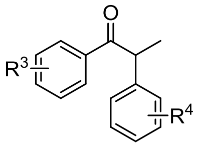


| Entry | | Entry | |
|---|---|---|---|
| 1s | $R^3$ = H, $R^4$ = H | 1ab | $R^3$ = 2-Naph, $R^4$ = H |
| 1t | $R^3$ = 4-Me, $R^4$ = H | 1ac | $R^3$ = H, $R^4$ = 2-Naph |
| 1u | $R^3$ = H, $R^4$ = 4-Me | 1ad | $R^3$ = H, $R^4$ = 4-F |
| 1v | $R^3$ = 3-OMe, $R^4$ = H | 1ae | $R^3$ = H, $R^4$ = 4-Cl |
| 1w | $R^3$ = 4-OMe, $R^4$ = H | 1af | $R^3$ = 4-OMe, $R^4$ = 4-F |
| 1x | $R^3$ = H, $R^4$ = 3-OMe | 1ag | $R^3$ = 3-N,N-di-Me, $R^4$ = H |
| 1y | $R^3$ = H, $R^4$ = 4-Ph | 1ah | $R^3$ = 3,4-di-OMe, $R^4$ = H |
| 1z | $R^3$ = 4-Ph, $R^4$ = H | | |
| 1aa | $R^3$ = 1-Naph, $R^4$ = H | | |

Substrates **1s-1z**[1,3], **1aa-1ah**[2,3] were prepared in accordance with methods described in the references.

$BrR_f$

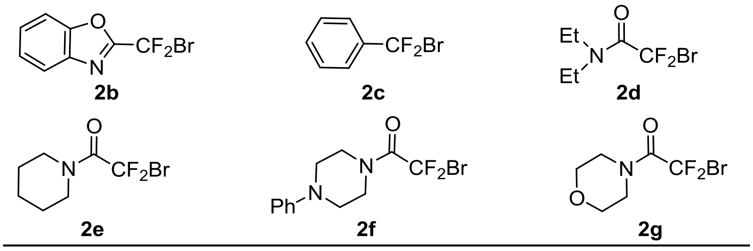


Substrates **2b**[8], **2c**[10], **2d**[9], **2e**[9], **2f**[9], **2g**[11] were prepared in accordance with methods described in the references.

**General Procedure for Cobalt-Catalyzed Difluoroalkyaltion of Tertiary Aryl Ketones.**

To a 50 mL of Schlenk tube was added aryl ketone **1** (1.0 equiv, 0.2 mmol), $CoBr_2$ (10 mol %, 0.02 mmol) and dppBz (10 mol %, 0.02 mmol) under air, followed by Zn (0.5 equiv, 0.1 mmol). The mixture was evacuated and backfilled with $N_2$ (3 times). THF (2 mL) was added then followed by LDA (105 mol%, 0.21 mmol) subsequently. The Schlenk tube was then sealed with a Teflon lined cap and put into a cooled bath (- 10°C). After stirring for 5 minutes, bormdifluoroacetate **2a** (3.0 equiv, 0.6 mmol) was added to the reaction mixture, and the Schlenk tube was then resealed with a Teflon lined cap and put back into the cooled bath (- 10°C). After stirring for another 12 hours, the reaction mixture was diluted with ethyl acetate (5 mL). The solvent was removed under reduced pressure, and the residue was purified by flash column chromatography on silica gel to give the desired product.

**Characterization Data for Alkyl Difluorides**

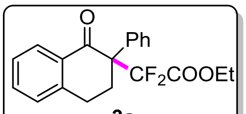


**3a**

Ethyl (R)-2,2-difluoro-2-(1-oxo-2-phenyl-1,2,3,4-tetrahydronaphthalen-2-yl)acetate (**3a**). The product **3a** is purified with silica gel chromatography (petroleum ether/ethyl acetate = 10/1 as the eluent) as yellow oil (90% yield). $^1$H NMR (400 MHz, Chloroform-*d*) δ 8.16 (d, *J* = 7.9, Hz, 1H), 7.49 – 7.41 (m, 1H), 7.26 – 7.34 (m, 6H), 7.10 (d, *J* = 7.6 Hz, 1H), 4.26 (qq, *J* = 10.7, 7.1 Hz, 2H), 3.01 (td, *J* = 13.5, 4.5 Hz, 1H), 2.92 – 2.83 (m, 2H), 2.73 – 2.64 (m, 1H), 1.22 (t, *J* = 7.1 Hz, 3H). $^{13}$C NMR (101 MHz, Chloroform-*d*) δ 196.07 (t, *J* = 2.9 Hz), 163.56 (t, *J* = 32.4 Hz), 143.17, 134.19, 132.29, 131.92, 129.02, 128.74, 128.39, 128.36, 128.16, 127.00, 115.22 (t, *J* = 257.6 Hz), 62.63, 60.87 (dd, *J* = 22.4, 20.9 Hz), 28.51 (dd, *J* = 5.8, 3.2 Hz), 25.27, 13.77. $^{19}$F NMR (376 MHz, Chloroform-*d*) δ -107.22 (d, *J* = 272.2 Hz, 1F), -110.19 (d, *J* = 272.1 Hz, 1F). HRMS (ESI) (*m/z*): $[M+H]^+$ calcd. for $C_{20}H_{19}O_3F_2$: 345.1297, found: 345.1301.

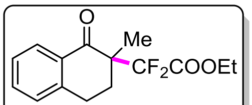


**3b**

Ethyl (S)-2,2-difluoro-2-(2-methyl-1-oxo-1,2,3,4-tetrahydronaphthalen-2-yl)acetate (**3b**). The product **3b** is purified with silica gel chromatography (petroleum ether/ethyl acetate = 10/1 as the eluent) as yellow oil (64% yield). $^1$H NMR (400 MHz, Chloroform-*d*) δ 8.01 (d, *J* = 7.7 Hz, 1H), 7.57 – 7.45 (m, 1H), 7.36 – 7.28 (m, 1H), 7.25 (d, *J* = 8.0 Hz, 1H), 4.37 (q, *J* = 7.1 Hz, 2H), 3.14 – 3.01 (m, 2H), 2.64 – 2.56 (m, 1H), 2.11 (dt, *J* = 13.7, 4.5 Hz, 1H), 1.52 (s, 3H), 1.37 (t, *J* = 7.1 Hz, 3H). $^{13}$C NMR (101 MHz, Chloroform-*d*) δ 196.59 (t, *J* = 3.1 Hz), 163.74 (t, *J* = 32.3 Hz), 142.87, 134.08, 131.18, 128.81, 128.23, 127.06, 116.41 (t, *J* = 257.2 Hz), 62.91, 51.93 (t, *J* = 21.9 Hz), 28.54 (t, *J* = 4.1 Hz), 24.86, 16.75 (t, *J* = 4.2 Hz), 13.98. $^{19}$F NMR (376 MHz, Chloroform-*d*) δ -111.56 (d, *J* = 266.0 Hz, 1F), -112.33 (d, *J* = 266.0 Hz, 1F). HRMS (ESI) (*m/z*): $[M+H]^+$ calcd. for $C_{15}H_{17}O_3F_2$: 283.1140, found: 283.1145.

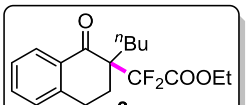


**3c**

Ethyl (S)-2-(2-butyl-1-oxo-1,2,3,4-tetrahydronaphthalen-2-yl)-2,2-difluoroacetate (**3c**). The product **3c** is purified with silica gel chromatography (petroleum ether/ethyl acetate = 10/1 as the eluent) as yellow oil (40% yield). $^1$H NMR (400 MHz, Chloroform-*d*) δ 8.02 (d, *J* = 7.8 Hz, 1H), 7.60 – 7.44 (m, 1H), 7.40 –

7.28 (m, 1H), 7.25 (d, *J* = 7.9 Hz, 1H), 4.33 (q, *J* = 7.1 Hz, 2H), 3.08 – 2.97 (m, 2H), 2.63 – 2.55 (m, 1H), 2.35 (dt, *J* = 13.6, 4.7 Hz, 1H), 1.96 – 1.78 (m, 2H), 1.58 – 1.49 (m, 1H), 1.43 - 1.28 (m, 6H), 0.90 (t, *J* = 7.1 Hz, 3H). $^{13}$C NMR (101 MHz, Chloroform-*d*) δ 196.82 (t, *J* = 3.5 Hz), 163.78 (t, *J* = 32.4 Hz), 143.09, 134.00, 131.39, 128.82, 128.23, 127.02, 117.02 (dd, *J* = 260.0, 256.6 Hz), 62.87, 55.10 (t, *J* = 20.5 Hz), 30.19 (t, *J* = 3.1 Hz), 26.64, 25.21 (dd, *J* = 5.3, 3.3 Hz), 25.04, 23.40, 13.97, 13.95. $^{19}$F NMR (376 MHz, Chloroform-*d*) δ -106.55 (d, *J* = 269.9 Hz, 1F), -107.72 (d, *J* = 269.9 Hz, 1F). HRMS (ESI) (*m/z*): $[M+Na]^+$ calcd. for $C_{18}H_{22}O_3F_2Na$: 347.1429, found: 347.1434.

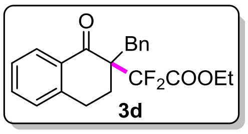


Ethyl (R)-2-(2-benzyl-1-oxo-1,2,3,4-tetrahydronaphthalen-2-yl)-2,2-difluoroacetate (**3d**). The product **3d** is purified with silica gel chromatography (petroleum ether/ethyl acetate = 10/1 as the eluent) as yellow oil (88% yield). $^{1}$H NMR (400 MHz, Chloroform-*d*) δ 8.04 (d, *J* = 7.8 Hz, 1H), 7.48 – 7.42 (m, 1H), 7.33 – 7.27 (m, 1H), 7.20 – 7.14 (m, 5H), 7.16 (d, *J* = 5.9 Hz, 1H), 4.40 – 4.28 (m, 2H), 3.65 (d, *J* = 13.8 Hz, 1H), 3.12 – 3.04 (m, 1H), 3.04 (d, *J* = 13.8 Hz, 1H), 2.74 (dt, *J* = 17.1, 6.3 Hz, 1H), 2.47 – 2.41 (m, 1H), 2.20 – 2.13 (m, 1H), 1.33 (t, *J* = 7.1 Hz, 3H). $^{13}$C NMR (101 MHz, Chloroform-*d*) δ 195.35 (t, *J* = 2.7 Hz), 163.34 (t, *J* = 32.5 Hz), 143.41, 135.84, 133.98, 132.49, 131.00, 128.71, 128.35, 128.15, 127.01, 126.89, 117.11 (t, *J* = 260.8 Hz), 63.20, 55.49 (t, *J* = 19.7 Hz), 37.28 (t, *J* = 4.0 Hz), 26.20 (t, *J* = 3.2 Hz), 25.42, 13.97. $^{19}$F NMR (376 MHz, Chloroform-*d*) δ -106.66 (d, *J* = 262.6 Hz, 1F), -107.38 (d, *J* = 262.5 Hz, 1F). HRMS (ESI) (*m/z*): $[M+H]^+$ calcd. for $C_{21}H_{21}O_3F_2$: 359.1453, found: 359.1451.

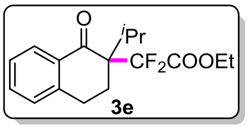


Ethyl (R)-2,2-difluoro-2-(2-isopropyl-1-oxo-1,2,3,4-tetrahydronaphthalen-2-yl)acetate (**3e**). The product **3e** is purified with silica gel chromatography (petroleum ether/ethyl acetate = 10/1 as the eluent) as yellow oil (40% yield). $^{1}$H NMR (400 MHz, Chloroform-*d*) δ 8.05 – 7.94 (m, 1H), 7.54 – 7.43 (m, 1H), 7.34 – 7.28 (m, 1H), 7.23 (d, *J* = 7.7 Hz, 1H), 4.34 -4.27 (m, 2H), 3.20 – 3.12 (m, 1H), 3.03 - 2.95 (m, 1H), 2.71 – 2.61 (m, 2H), 2.27 (ddd, *J* = 14.3, 8.5, 5.5 Hz, 1H), 1.32 (t, *J* = 7.1 Hz, 3H), 1.05 (dd, *J* = 6.9, 2.4 Hz, 3H), 0.93 (dd, *J* = 6.9, 1.0 Hz, 3H). $^{13}$C NMR (101 MHz, Chloroform-*d*) δ 197.32 (t, *J* = 4.1 Hz), 163.71 (t, *J* = 32.3 Hz), 143.16 , 133.94 , 132.74 , 128.79 , 127.92 , 126.97 , 118.36 (dd, *J* = 264.8, 255.9 Hz), 62.97, 57.68 (t, *J* = 19.5 Hz), 30.90 , 25.70 (d, *J* = 3.0 Hz) , 22.57 (t, *J* = 4.2 Hz), 18.88, 18.15 (d, *J* = 6.1 Hz), 13.91. $^{19}$F NMR (376 MHz, Chloroform-*d*) δ -102.26 (d, *J* = 267.8 Hz, 1F), -104.49 (d, *J* = 267.9 Hz, 1F). HRMS (ESI) (m/z): $[M+H]^+$ calcd. for $C_{17}H_{20}O_3F_2$: 333.1273, found: 333.1279.

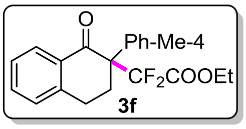


Ethyl (R)-2,2-difluoro-2-(1-oxo-2-(p-tolyl)-1,2,3,4-tetrahydronaphthalen-2-yl)acetate (**3f**). The product **3f** is purified with silica gel chromatography (petroleum ether/ethyl acetate = 10/1 as the eluent) as yellow oil (96% yield). $^{1}$H NMR (400 MHz, Chloroform-*d*) δ 8.15 (d, *J* = 7.8 Hz, 1H), 7.48 – 7.39 (m, 1H), 7.35 – 7.28 (m, 1H), 7.23 – 7.16 (m, 2H), 7.12 – 7.08 (m, 3H), 4.34 – 4.21 (m, 2H), 2.99 (td, *J* = 13.4, 4.3 Hz, 1H), 2.88 – 2.84 (m, 2H), 2.76 – 2.68 (m, 1H), 2.30 (s, 3H), 1.26 (t, *J* = 7.3 Hz, 3H). $^{13}$C NMR (101 MHz, Chloroform-*d*) δ 196.13 (t, *J* = 3.1 Hz), 163.64 (t, *J* = 32.3 Hz), 143.14, 138.18, 134.08, 131.90, 129.12,

128.89, 128.71, 128.09, 126.92, 115.19 (t, $J$ = 258.6 Hz), 62.62, 60.55 (t, $J$ = 22.2 Hz), 28.33 (dd, $J$ = 5.8, 3.1 Hz), 25.27, 21.09, 13.79. $^{19}$F NMR (376 MHz, Chloroform-*d*) δ -107.13 (d, $J$ = 271.8 Hz, 1F), -110.44 (d, $J$ = 271.7 Hz, 1F). HRMS (ESI) (*m/z*): $[M+H]^+$ calcd. for $C_{21}H_{21}O_3F_2$: 359.1453, found: 359.1459.

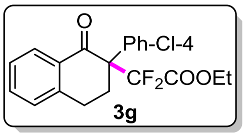


Ethyl (R)-2-(2-(4-chlorophenyl)-1-oxo-1,2,3,4-tetrahydronaphthalen-2-yl)-2,2-difluoroacetate (**3g**). The product **3g** is purified with silica gel chromatography (petroleum ether/ethyl acetate = 10/1 as the eluent) as yellow oil (88% yield). $^1$H NMR (400 MHz, Chloroform-*d*) δ 8.19 – 8.07 (m, 1H), 7.51 – 7.42 (m, 1H), 7.37 – 7.30 (m, 1H), 7.30 – 7.26 (m, 2H), 7.26 – 7.21 (m, 2H), 7.11 (d, $J$ = 7.7 Hz, 1H), 4.33 – 4.21 (m, 2H), 3.04 – 2.95 (m, 1H), 2.90 – 2.80 (m, 2H), 2.70 – 2.61 (m, 1H), 1.25 (t, $J$ = 7.1 Hz, 3H). $^{13}$C NMR (101 MHz, Chloroform-*d*) δ 195.71 (t, $J$ = 2.9 Hz), 163.43 (t, $J$ = 32.3 Hz), 142.99, 134.62, 134.47, 131.66, 130.87, 130.43, 128.83, 128.67, 128.25, 127.18, 114.98 (t, $J$ = 257.9 Hz), 62.84, 60.38 (dd, $J$ = 22.7, 21.0 Hz), 28.37 (dd, $J$ = 5.8, 3.2 Hz), 25.14, 13.84. $^{19}$F NMR (376 MHz, Chloroform-*d*) δ -107.37 (d, $J$ = 272.6 Hz, 1F), -110.35 (d, $J$ = 272.6 Hz, 1F). HRMS (ESI) (*m/z*): $[M+H]^+$ calcd. for $C_{20}H_{18}O_3ClF_2$: 379.0907, found: 379.0909.

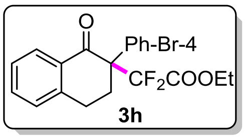


Ethyl (R)-2-(2-(4-bromophenyl)-1-oxo-1,2,3,4-tetrahydronaphthalen-2-yl)-2,2-difluoroacetate (**3h**). The product **3h** is purified with silica gel chromatography (petroleum ether/ethyl acetate = 10/1 as the eluent) as yellow oil (95% yield). $^1$H NMR (400 MHz, Chloroform-*d*) δ 8.18 – 8.09 (m, 1H), 7.52 – 7.38 (m, 3H), 7.36 – 7.29 (m, 1H), 7.17 (d, $J$ = 8.5 Hz, 2H), 7.11 (d, $J$ = 7.7 Hz, 1H), 4.33 – 4.21 (m, 2H), 3.03 – 2.95 (m, 1H), 2.90 – 2.80 (m, 2H), 2.70 – 2.61 (m, 1H), 1.25 (t, $J$ = 7.1 Hz, 3H). $^{13}$C NMR (101 MHz, Chloroform-*d*) δ 195.66 (t, $J$ = 2.9 Hz), 163.39 (t, $J$ = 32.2 Hz), 142.97, 134.48, 131.60, 131.40, 130.72, 128.83, 128.22, 127.16, 122.84, 114.89 (t, $J$ = 257.9 Hz), 62.84, 60.41 (dd, $J$ = 22.7, 21.0 Hz), 28.28 (dd, $J$ = 5.7, 3.3 Hz), 25.11, 13.83. $^{19}$F NMR (376 MHz, Chloroform-*d*) δ -107.44 (d, $J$ = 272.7 Hz, 1F), -110.39 (d, $J$ = 272.6 Hz, 1F). HRMS (ESI) (*m/z*): $[M+H]^+$ calcd. for $C_{20}H_{18}O_3BrF_2$: 423.0402, found: 423.0400.

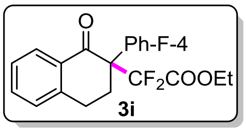


Ethyl (R)-2,2-difluoro-2-(2-(4-fluorophenyl)-1-oxo-1,2,3,4-tetrahydronaphthalen-2-yl)acetate (**3i**). The product **3i** is purified with silica gel chromatography (petroleum ether/ethyl acetate = 10/1 as the eluent) as yellow oil (88% yield). $^1$H NMR (400 MHz, Chloroform-*d*) δ 8.14 (d, $J$ = 7.9 Hz, 1H), 7.54 – 7.41 (m, 1H), 7.34 – 7.26 (m, 3H), 7.11 (d, $J$ = 7.7 Hz, 1H), 7.05 – 6.92 (m, 2H), 4.33 – 4.20 (m, 2H), 2.99 (td, $J$ = 14.2, 13.6, 4.6 Hz, 1H), 2.90 – 2.81 (m, 2H), 2.71 – 2.62 (m, 1H), 1.24 (t, $J$ = 7.1 Hz, 3H). $^{13}$C NMR (101 MHz, Chloroform-*d*) δ 195.83 (t, $J$ = 2.9 Hz), 163.47 (t, $J$ = 32.3 Hz), 162.71 (d, $J$ = 248.2 Hz), 143.01, 134.39, 131.71, 130.84 (d, $J$ = 8.2 Hz), 128.79, 128.24, 127.95, 127.13, 115.05 (t, $J$ = 258.6 Hz), 115.44 (d, $J$ = 21.5 Hz), 62.75, 60.29 (dd, $J$ = 22.7, 21.1 Hz), 28.49 (dd, $J$ = 5.7, 3.2 Hz), 25.15, 13.8. $^{19}$F NMR (376 MHz, Chloroform-*d*) δ -107.44 (d, $J$ = 272.4 Hz, 1F), -110.43 (d, $J$ = 272.4 Hz, 1F), -113.53

(s, 1F). HRMS (ESI) (*m/z*): $[M+H]^+$ calcd. for $C_{20}H_{18}O_3F_3$: 363.1203, found: 363.1208.

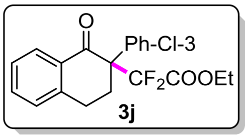

Ethyl (R)-2-(2-(3-chlorophenyl)-1-oxo-1,2,3,4-tetrahydronaphthalen-2-yl)-2,2-difluoroacetate (**3j**). The product **3j** is purified with silica gel chromatography (petroleum ether/ethyl acetate = 10/1 as the eluent) as yellow oil (88% yield). $^1$H NMR (400 MHz, Chloroform-*d*) δ 8.15 (d, *J* = 7.9 Hz, 1H), 7.52 – 7.41 (m, 1H), 7.35 – 7.11 (m, 6H), 4.33 – 4.18 (m, 2H), 3.00 (td, *J* = 14.2, 13.5, 4.6 Hz, 1H), 2.90 – 2.82 (m, 2H), 2.72 – 2.63 (m, 1H), 1.22 (t, *J* = 7.1 Hz, 3H). $^{13}$C NMR (101 MHz, Chloroform-*d*) δ 195.40 (t, *J* = 2.9 Hz), 163.28 (t, *J* = 32.3 Hz), 143.05, 134.50, 131.61, 129.55, 129.16, 128.82, 128.70, 128.31, 127.36, 127.20, 114.96 (t, *J* = 259.6 Hz), 62.78, 60.62 (dd, *J* = 22.7, 21.0 Hz), 28.45 (dd, *J* = 5.7, 3.3 Hz), 25.16, 13.76. $^{19}$F NMR (376 MHz, Chloroform-*d*) δ -107.37 (d, *J* = 272.8 Hz, 1F), -110.00 (d, *J* = 272.7 Hz, 1F). HRMS (ESI) (*m/z*): $[M+H]^+$ calcd. for $C_{20}H_{18}O_3ClF_2$: 379.0907, found: 379.0914.

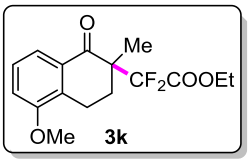

Ethyl (S)-2,2-difluoro-2-(5-methoxy-2-methyl-1-oxo-1,2,3,4-tetrahydronaphthalen-2-yl)acetate (**3k**). The product **3k** is purified with silica gel chromatography (petroleum ether/ethyl acetate = 10/1 as the eluent) as yellow oil (69% yield). $^1$H NMR (400 MHz, Chloroform-*d*) δ 7.57 (dd, *J* = 7.9, 0.9 Hz, 1H), 7.23 (d, *J* = 8.0 Hz, 1H), 7.00 (dd, *J* = 8.1, 0.8 Hz, 1H), 4.32 (q, *J* = 7.1 Hz, 2H), 3.83 (s, 3H), 3.11 (dt, *J* = 18.3, 4.7 Hz, 1H), 2.80 – 2.72 (m, 1H), 2.51 (ddd, *J* = 13.8, 11.2, 5.5 Hz, 1H), 2.11 – 2.05 (m, 1H), 1.46 (s, 3H), 1.33 (t, *J* = 7.2 Hz, 3H). $^{13}$C NMR (101 MHz, Chloroform-*d*) δ 196.99 (t, *J* = 2.8 Hz), 163.85 (t, *J* = 32.3 Hz), 156.70, 131.96, 127.30, 119.67, 116.35 (t, *J* = 256.9 Hz), 114.73, 62.87, 55.81, 51.66 (t, *J* = 21.9 Hz), 27.67 (t, *J* = 4.3 Hz), 18.71, 16.52 (t, *J* = 4.2 Hz), 14.00. $^{19}$F NMR (376 MHz, Chloroform-*d*) δ -111.92 (d, *J* = 266.6 Hz, 1F), -112.67 (d, *J* = 266.6 Hz, 1F). HRMS (ESI) (*m/z*): $[M+Na]^+$ calcd. for $C_{16}H_{18}F_2NaO_4$: 335.1071, found: 335.1075.

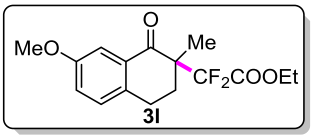

Ethyl (S)-2,2-difluoro-2-(7-methoxy-2-methyl-1-oxo-1,2,3,4-tetrahydronaphthalen-2-yl)acetate (**3l**). The product **3l** is purified with silica gel chromatography (petroleum ether/ethyl acetate = 10/1 as the eluent) as yellow oil (85% yield). $^1$H NMR (400 MHz, Chloroform-*d*) δ 7.47 (d, *J* = 2.8 Hz, 1H), 7.15 (d, *J* = 8.4 Hz, 1H), 7.08 (dd, *J* = 8.4, 2.8 Hz, 1H), 4.37 (q, *J* = 7.1 Hz, 2H), 3.81 (s, 3H), 3.06 – 2.93 (m, 2H), 2.60 – 2.53 (m, 1H), 2.08 (dt, *J* = 13.6, 4.6 Hz, 1H), 1.50 (s, 3H), 1.37 (t, *J* = 7.1 Hz, 3H). $^{13}$C NMR (101 MHz, Chloroform-*d*) δ 196.66 (t, *J* = 3.1 Hz), 163.77 (t, *J* = 32.3 Hz), 158.60, 135.47, 131.79, 130.05, 122.76, 116.39 (t, *J* = 257.1 Hz), 109.77, 62.89, 55.56, 51.86 (t, *J* = 22.0 Hz), 28.60 (dd, *J* = 5.1, 4.0 Hz), 24.02, 16.64 (t, *J* = 4.1 Hz), 13.99. $^{19}$F NMR (376 MHz, Chloroform-*d*) δ -111.59 (d, *J* = 266.2 Hz, 1F), -112.41 (d, *J* = 266.2 Hz, 1F). HRMS (ESI) (*m/z*): $[M+H]^+$ calcd. for $C_{16}H_{19}O_4F_2$: 313.1246, found: 313.1244.

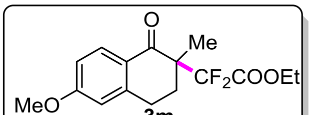
3m

Ethyl (S)-2,2-difluoro-2-(6-methoxy-2-methyl-1-oxo-1,2,3,4-tetrahydronaphthalen-2-yl)acetate (**3m**). The product **3m** is purified with silica gel chromatography (petroleum ether/ethyl acetate = 10/1 as the eluent) as yellow oil (75% yield). $^1$H NMR (400 MHz, Chloroform-*d*) δ 7.98 (d, *J* = 8.8 Hz, 1H), 6.83 (dd, *J* = 8.8, 2.4 Hz, 1H), 6.68 (d, *J* = 2.1 Hz, 1H), 4.37 (q, *J* = 7.1 Hz, 2H), 3.85 (s, 3H), 3.11 - 2.96 (m, 2H), 2.58 (ddd, *J* = 13.7, 11.1, 5.6 Hz, 1H), 2.11 – 2.04 (m, 1H), 1.50 (s, 3H), 1.37 (t, *J* = 7.1 Hz, 3H). $^{13}$C NMR (101 MHz, Chloroform-*d*) δ 195.34 (t, *J* = 2.8 Hz), 164.20, 163.86 (t, *J* = 32.3Hz), 145.44 , 130.79 , 124.69 , 116.57 (t, *J* = 258.0Hz), 113.81, 112.47, 62.86, 55.62, 51.79 (t, *J* = 21.9 Hz), 28.56 (dd, *J* = 5.3, 3.4 Hz), 25.31, 16.99 (t, *J* = 4.2 Hz), 14.01. $^{19}$F NMR (376 MHz, Chloroform-*d*) δ -111.62 (d, *J* = 264.7 Hz, 1F), -112.54 (d, *J* = 264.8 Hz, 1F). HRMS (ESI) (*m/z*): [M+H]$^+$ calcd. for $C_{16}H_{19}O_4F_2$: 313.1246, found: 313.1252.

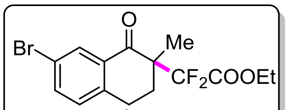
3n

Ethyl (S)-2-(7-bromo-2-methyl-1-oxo-1,2,3,4-tetrahydronaphthalen-2-yl)-2,2-difluoroacetate (**3n**). The product **3n** is purified with silica gel chromatography (petroleum ether/ethyl acetate = 10/1 as the eluent) as yellow oil (65% yield). $^1$H NMR (400 MHz, Chloroform-*d*) δ 8.12 (d, *J* = 2.2 Hz, 1H), 7.60 (dd, *J* = 8.2, 2.2 Hz, 1H), 7.14 (d, *J* = 8.2 Hz, 1H), 4.36 (q, *J* = 7.1 Hz, 2H), 3.01 (dd, *J* = 8.0, 4.9 Hz, 2H), 2.57 (dt, *J* = 13.9, 8.1 Hz, 1H), 2.10 (dt, *J* = 13.8, 4.8 Hz, 1H), 1.48 (s, 3H), 1.36 (t, *J* = 7.2 Hz, 3H). $^{13}$C NMR (101 MHz, Chloroform-*d*) δ 195.32 (t, *J* = 2.8 Hz), 163.52 (t, *J* = 32.2 Hz), 141.58, 136.83, 132.69, 130.94, 130.66, 121.07, 116.22 (t, *J* = 257.8 Hz), 63.05, 51.63 (t, *J* = 21.9 Hz), 28.47 (t, *J* = 4.2 Hz), 24.47, 16.72 (t, *J* = 4.2 Hz), 13.98. $^{19}$F NMR (376 MHz, Chloroform-*d*) δ -111.57 (s, 2F). HRMS (ESI) (*m/z*): [M+H]$^+$ calcd. for $C_{15}H_{16}O_3BrF_2$: 361.0245, found: 361.0253.

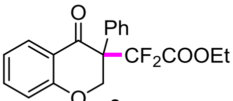
3o

Ethyl (S)-2,2-difluoro-2-(4-oxo-3-phenylchroman-3-yl)acetate (**3o**). The product **3o** is purified with silica gel chromatography (petroleum ether/ethyl acetate = 10/1 as the eluent) as yellow oil (50% yield). $^1$H NMR (400 MHz, Chloroform-*d*) δ 7.97 (d, *J* = 7.9 Hz, 1H), 7.44 – 7.33 (m, 6H), 7.07 – 6.98 (m, 1H), 6.83 (d, *J* = 8.4 Hz, 1H), 5.23 (t, *J* = 12.0, 2H), 4.36 – 4.24 (m, 2H), 1.28 (t, *J* = 7.2 Hz, 3H). $^{13}$C NMR (101 MHz, Chloroform-*d*) δ 190.20 (dd, *J* = 4.2, 1.1 Hz), 163.14 (t, *J* = 31.8 Hz), 160.66, 136.64, 129.59 (d, *J* = 2.4 Hz), 129.01, 128.62, 127.93, 122.01, 120.21, 117.80, 113.89 (dd, *J* = 263.8, 254.2 Hz), 69.34 (dd, *J* = 8.8, 3.7 Hz), 63.28, 59.18 (t, *J* = 21.1 Hz), 13.84. $^{19}$F NMR (376 MHz, Chloroform-*d*) δ -103.58 (d, *J* = 278.9 Hz, 1F), -110.21 (d, *J* = 278.9 Hz, 1F). HRMS (ESI) (m/z): [M+Na]$^+$ calcd. for $C_{19}H_{16}O_4F_2Na$: 369.0909, found: 369.0912.

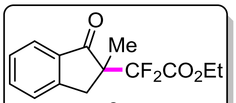
3p

Ethyl (S)-2,2-difluoro-2-(2-methyl-1-oxo-2,3-dihydro-1H-inden-2-yl)acetate (**3p**). The product **3p** is

purified with silica gel chromatography (petroleum ether/ethyl acetate = 10/1 as the eluent) as yellow oil (50% yield). $^{1}$H NMR (400 MHz, Chloroform-*d*) δ 7.78 (d, *J* = 7.6 Hz, 1H), 7.66 – 7.58 (m, 1H), 7.46 (d, *J* = 7.6 Hz, 1H), 7.43 – 7.37 (m, 1H), 4.21 (q, *J* = 7.1 Hz, 2H), 3.59 (d, *J* = 17.6 Hz, 1H), 2.94 (d, *J* = 17.7 Hz, 1H), 1.49 (s, 3H), 1.20 (t, *J* = 7.1 Hz, 3H). $^{13}$C NMR (101 MHz, Chloroform-*d*) δ 202.57 (t, *J* = 2.7 Hz), 162.91 (t, *J* = 32.8 Hz), 151.49, 135.60, 135.34 (t, *J* = 2.1 Hz), 128.09, 126.60, 124.85, 115.93 (t, *J* = 257.8 Hz), 63.17, 53.77 (t, *J* = 21.7 Hz), 37.06, 18.96 (t, *J* = 4.6 Hz), 13.80. $^{19}$F NMR (376 MHz, Chloroform-*d*) δ -109.86 (d, *J* = 264.1 Hz, 1F), -110.58 (d, *J* = 264.2 Hz, 1F). HRMS (ESI) (*m/z*): $[M+H]^+$ calcd. for $C_{14}H_{15}O_3F_2$: 269.0984, found: 269.0994.

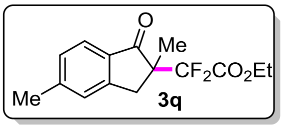


Ethyl (S)-2-(2,5-dimethyl-1-oxo-2,3-dihydro-1H-inden-2-yl)-2,2-difluoroacetate (**3q**). The product **3q** is purified with silica gel chromatography (petroleum ether/ethyl acetate = 10/1 as the eluent) as yellow oil (64% yield). $^{1}$H NMR (400 MHz, Chloroform-*d*) δ 7.57 (s, 1H), 7.44 (dd, *J* = 7.8, 1.2 Hz, 1H), 7.34 (d, *J* = 7.8 Hz, 1H), 4.22 (q, *J* = 7.1 Hz, 2H), 3.53 (d, *J* = 17.5 Hz, 1H), 2.88 (d, *J* = 17.5 Hz, 1H), 2.40 (s, 3H), 1.48 (s, 3H), 1.21 (t, *J* = 7.2 Hz, 3H). $^{13}$C NMR (101 MHz, Chloroform-*d*) δ 202.66 (t, *J* = 2.8 Hz), 162.96 (t, *J* = 32.7 Hz), 148.87, 138.12, 136.89, 135.48, 126.26, 124.70, 115.99 (t, *J* = 257.8 Hz), 63.15, 54.07 (t, *J* = 21.6 Hz), 36.70 (t, *J* = 3.4 Hz), 21.20, 19.00 (t, *J* = 4.7 Hz), 13.82. $^{19}$F NMR (376 MHz, Chloroform-*d*) δ -110.25 (s, 2F). HRMS (ESI) (*m/z*): $[M+Na]^+$ calcd. for $C_{15}H_{16}F_2NaO_3$: 305.0965, found: 305.0965.

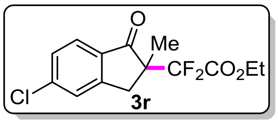


Ethyl (S)-2-(5-chloro-2-methyl-1-oxo-2,3-dihydro-1H-inden-2-yl)-2,2-difluoroacetate (**3r**). The product **3r** is purified with silica gel chromatography (petroleum ether/ethyl acetate = 10/1 as the eluent) as yellow oil (53% yield). $^{1}$H NMR (400 MHz, Chloroform-*d*) δ 7.71 (d, *J* = 8.2 Hz, 1H), 7.46 (s, 1H), 7.39 (d, *J* = 8.2 Hz, 1H), 4.24 (q, *J* = 7.1 Hz, 2H), 3.57 (d, *J* = 17.8 Hz, 1H), 2.92 (d, *J* = 17.8 Hz, 1H), 1.48 (s, 3H), 1.24 (t, *J* = 7.1 Hz, 3H). $^{13}$C NMR (101 MHz, Chloroform-*d*) δ 201.16 (t, *J* = 2.9 Hz), 162.78 (t, *J* = 32.5 Hz), 152.91, 142.21, 133.77 (t, *J* = 2.1 Hz), 128.97, 126.83, 125.95, 115.76 (t, *J* = 258.1 Hz), 63.32, 53.98 (t, *J* = 21.8 Hz), 36.79 (t, *J* = 3.5 Hz), 18.99 (t, *J* = 4.6 Hz), 13.87. $^{19}$F NMR (376 MHz, Chloroform-*d*) δ -110.10 (s, 2F). HRMS (ESI) (*m/z*): $[M+Na]^+$ calcd. for $C_{14}H_{13}ClF_2NaO_3$: 325.0419, found: 325.0419.

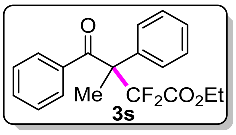


Ethyl (R)-2,2-difluoro-3-methyl-4-oxo-3,4-diphenylbutanoate (**3s**). The product **3s** is purified with silica gel chromatography (petroleum ether/ethyl acetate = 10/1 as the eluent) as yellow oil (80% yield). $^{1}$H NMR (400 MHz, Chloroform-*d*) δ 7.48 (d, *J* = 8.0 Hz, 2H), 7.44 – 7.38 (m, 6H), 7.29 – 7.20 (m, 2H), 4.41 – 4.29 (m, 2H), 2.12 (s, 3H), 1.36 (t, *J* = 7.1 Hz, 3H). $^{13}$C NMR (101 MHz, Chloroform-*d*) δ 199.36 (d, *J* = 4.6 Hz), 163.98 (t, *J* = 32.3 Hz), 136.00, 134.55, 132.95, 130.24, 129.43, 128.69, 128.44, 128.28, 114.66 (dd, *J* = 262.6, 252.8 Hz), 62.72, 61.90 (dd, *J* = 22.8, 20.0 Hz), 20.45 (dd, *J* = 5.9, 3.3 Hz), 13.96. $^{19}$F NMR (376 MHz, Chloroform-*d*) δ -106.64 (d, *J* = 270.4 Hz, 1F), -111.89 (d, *J* = 270.5 Hz, 1F). HRMS (ESI) (*m/z*): $[M+H]^+$ calcd. for $C_{19}H_{19}O_3F_2$: 333.1297, found: 333.1294.

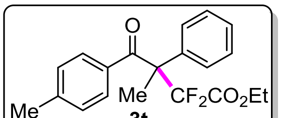

**3t**

Ethyl (R)-2,2-difluoro-3-methyl-4-oxo-3-phenyl-4-(p-tolyl)butanoate (**3t**). The product **3t** is purified with silica gel chromatography (petroleum ether/ethyl acetate = 10/1 as the eluent) as yellow oil (75% yield). $^{1}$H NMR (400 MHz, Chloroform-*d*) δ 7.41 – 7.35 (m, 7H), 7.05 (d, *J* = 8.1 Hz, 2H), 4.41 – 4.30 (m, 2H), 2.31 (s, 3H), 2.13 (s, 3H), 1.37 (t, *J* = 7.1 Hz, 3H). $^{13}$C NMR (101 MHz, Chloroform-*d*) δ 198.94 (d, *J* = 4.6 Hz), 164.06 (t, *J* = 32.3 Hz), 143.89, 136.33, 131.84, 130.48, 129.51, 128.98, 128.59, 128.36 ,114.72 (dd, *J* = 262.5, 252.5 Hz), 62.67, 61.95 (dd, *J* = 22.9, 19.9 Hz), 21.65, 20.58 (dd, *J* = 6.0, 3.3 Hz), 13.97. $^{19}$F NMR (376 MHz, Chloroform-*d*) δ -106.79 (d, *J* = 270.0 Hz, 1F), -112.07 (d, *J* = 270.0 Hz, 1F). HRMS (ESI) (*m/z*): [M+H]$^+$ calcd. for $C_{20}H_{21}O_3F_2$: 347.1453, found: 347.1455.

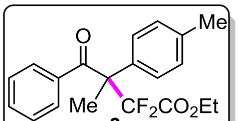

**3u**

Ethyl (R)-2,2-difluoro-3-methyl-4-oxo-4-phenyl-3-(p-tolyl)butanoate (**3u**). The product **3u** is purified with silica gel chromatography (petroleum ether/ethyl acetate = 10/1 as the eluent) as yellow oil (65% yield). $^{1}$H NMR (400 MHz, Chloroform-*d*) δ 7.42 – 7.40 (m, 2H), 7.36 – 7.30 (m, 1H), 7.21 – 7.14 (m, 4H), 7.09 (d, *J* = 8.1 Hz, 2H), 4.32 – 4.21 (m, 2H), 2.28 (s, 3H), 2.01 (s, 3H), 1.28 (t, *J* = 7.1 Hz, 3H). $^{13}$C NMR (101 MHz, Chloroform-*d*) δ 199.54 (d, *J* = 4.4 Hz), 164.05 (t, *J* = 32.4 Hz), 138.56, 134.62, 132.88, 130.23, 129.23, 129.18, 128.25, 114.67 (dd, *J* = 262.1, 252.5 Hz), 62.68, 61.61 (dd, *J* = 22.9, 20.1 Hz), 21.25, 20.40 (dd, *J* = 6.0, 3.3 Hz), 13.96. $^{19}$F NMR (376 MHz, Chloroform-*d*) δ -106.68 (d, *J* = 270.2 Hz, 1F), -111.90 (d, *J* = 270.2 Hz, 1F). HRMS (ESI) (*m/z*): [M+H]$^+$ calcd. for $C_{20}H_{21}O_3F_2$: 347.1453, found: 347.1458.

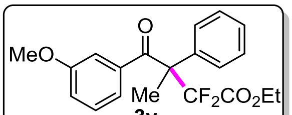

**3v**

Ethyl (R)-2,2-difluoro-4-(3-methoxyphenyl)-3-methyl-4-oxo-3-phenylbutanoate (**3v**). The product **3v** is purified with silica gel chromatography (petroleum ether/ethyl acetate = 10/1 as the eluent) as yellow oil (77% yield). $^{1}$H NMR (400 MHz, Chloroform-*d*) δ 7.48 – 7.33 (m, 5H), 7.14 – 7.10 (m, 2H), 6.97 (d, *J* = 7.9 Hz, 2H), 4.41 – 4.30 (m, 2H), 3.66 (s, 3H), 2.13 (s, 3H), 1.37 (t, *J* = 7.1 Hz, 3H). $^{13}$C NMR (101 MHz, Chloroform-*d*) δ 199.13 (d, *J* = 4.7 Hz), 163.97 (t, *J* = 32.3 Hz), 159.38, 135.96 (dd, *J* = 31.0, 1.6 Hz),. 129.47, 129.18, 128.70, 128.44, 122.85, 119.63, 114.69 (dd, *J* = 264.6, 253.5 Hz), 114.50, 62.73, 62.00 (dd, *J* = 22.9, 20.0 Hz), 55.32, 20.45 (dd, *J* = 5.8, 3.4 Hz), 13.97. $^{19}$F NMR (376 MHz, Chloroform-*d*) δ -106.70 (d, *J* = 270.4 Hz, 1F), -111.81 (d, *J* = 270.4 Hz, 1F). HRMS (ESI) (*m/z*): [M+H]$^+$ calcd. for $C_{20}H_{21}O_4F_2$: 363.1402, found: 363.1406.

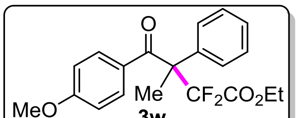

**3w**

Ethyl (R)-2,2-difluoro-4-(4-methoxyphenyl)-3-methyl-4-oxo-3-phenylbutanoate (**3w**). The product **3w** is purified with silica gel chromatography (petroleum ether/ethyl acetate = 10/1 as the eluent) as yellow oil

(85% yield). $^1$H NMR (400 MHz, Chloroform-*d*) δ 7.50 (d, *J* = 8.9 Hz, 2H), 7.45 – 7.29 (m, 5H), 6.72 (d, *J* = 8.9 Hz, 2H), 4.41 – 4.30 (m, 2H), 3.78 (s, 3H), 2.13 (s, 3H), 1.37 (t, *J* = 7.1 Hz, 3H). $^{13}$C NMR (101 MHz, Chloroform-*d*) δ 197.86 (d, *J* = 4.8 Hz), 164.12 (t, *J* = 32.3 Hz), 163.25, 136.53 (d, *J* = 2.1 Hz), 132.82, 129.53, 128.55, 128.31, 126.82, 114.68 (dd, *J* = 262.5, 251.9 Hz), 113.48, 62.66, 61.89 (dd, *J* = 22.9, 20.0 Hz), 55.52 , 20.75 (dd, *J* = 6.0, 3.3 Hz), 13.98. $^{19}$F NMR (376 MHz, Chloroform-*d*) δ -106.96 (d, *J* = 269.5 Hz, 1F), -112.27 (d, *J* = 269.5 Hz, 1F). HRMS (ESI) (*m/z*): [M+H]$^+$ calcd. for $C_{20}H_{21}O_4F_2$: 363.1402, found: 363.1398.

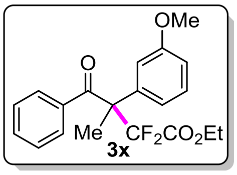


Ethyl (R)-2,2-difluoro-3-(3-methoxyphenyl)-3-methyl-4-oxo-4-phenylbutanoate (**3x**). The product **3x** is purified with silica gel chromatography (petroleum ether/ethyl acetate = 10/1 as the eluent) as yellow oil (76% yield). $^1$H NMR (400 MHz, Chloroform-*d*) δ 7.51 – 7.49 (m, 2H), 7.45 – 7.39 (m, 1H), 7.30 – 7.23 (m, 3H), 6.98 – 6.91 (m, 3H), 4.41 – 4.29 (m, 2H), 3.77 (s, 3H), 2.10 (s, 3H), 1.36 (t, *J* = 7.1 Hz, 3H). $^{13}$C NMR (101 MHz, Chloroform-*d*) δ 199.20 (d, *J* = 4.7 Hz), 163.94 (t, *J* = 32.3 Hz), 159.56, 137.39, 134.68, 132.91, 130.17, 129.29, 128.27, 122.10, 115.69,114.65 (dd, *J* = 263.3, 252.6 Hz), 113.71, 62.71, 61.83 (dd, *J* = 22.9, 19.8 Hz), 55.39, 20.47 (dd, *J* = 6.0, 3.3 Hz), 13.95. $^{19}$F NMR (376 MHz, Chloroform-*d*) δ -106.45 (d, *J* = 270.2 Hz, 1F), -111.73 (d, *J* = 270.2 Hz, 1F). HRMS (ESI) (*m/z*): [M+H]$^+$ calcd. for $C_{20}H_{21}O_4F_2$: 363.1402, found: 363.1402.

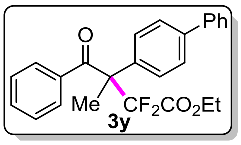


Ethyl (R)-3-([1,1'-biphenyl]-4-yl)-2,2-difluoro-3-methyl-4-oxo-4-phenylbutanoate (**3y**). The product **3y** is purified with silica gel chromatography (petroleum ether/ethyl acetate = 10/1 as the eluent) as yellow oil (76% yield). $^1$H NMR (400 MHz, Chloroform-*d*) δ 7.64 – 7.51 (m, 4H), 7.49 – 7.44 (m, 2H), 7.41 – 7.35 (m, 5H), 7.32 – 7.27 (m, 1H), 7.21 – 7.17 (m, 2H), 4.35 – 4.23 (m, 2H), 2.08 (s, 3H), 1.30 (t, *J* = 7.1 Hz, 3H). $^{13}$C NMR (101 MHz, Chloroform-*d*) δ 199.37 (d, *J* = 4.4 Hz), 163.99 (t, *J* = 32.3 Hz), 141.33, 140.17, 134.97, 134.55, 133.02, 130.28, 129.83, 128.96, 128.34, 127.78, 127.18, 127.02, 114.70 (dd, *J* = 262.4, 252.9 Hz), 62.78, 61.73 (dd, *J* = 22.8, 20.1 Hz), 20.49 (dd, *J* = 6.0, 3.2 Hz), 13.99. $^{19}$F NMR (376 MHz, Chloroform-*d*) δ -106.57 (d, *J* = 270.4 Hz, 1F), -111.70 (d, *J* = 270.4 Hz, 1F). HRMS (ESI) (*m/z*): [M+H]$^+$ calcd. for $C_{25}H_{23}O_3F_2$: 409.1610, found: 409.1606.

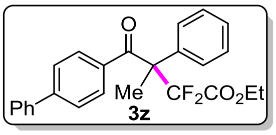


Ethyl (R)-4-([1,1'-biphenyl]-4-yl)-2,2-difluoro-3-methyl-4-oxo-3-phenylbutanoate (**3z**). The product **3z** is purified with silica gel chromatography (petroleum ether/ethyl acetate = 10/1 as the eluent) as yellow oil (65% yield). $^1$H NMR (400 MHz, Chloroform-*d*) δ 7.61 – 7.57 (m, 2H), 7.55 – 7.53 (m, 2H), 7.51 – 7.47 (m, 2H), 7.45 – 7.35 (m, 8H), 4.44 – 4.32 (m, 2H), 2.19 (s, 3H), 1.39 (t, *J* = 7.1 Hz, 3H). $^{13}$C NMR (101 MHz, Chloroform-*d*) δ 198.85 (d, *J* = 4.7 Hz), 164.02 (t, *J* = 32.3 Hz), 145.59 , 139.67 , 136.19 , 133.14 , 130.93 , 129.52 , 129.04 , 128.70 , 128.48 , 128.42 , 127.28 , 126.86 , 114.71 (dd, *J* = 262.6,

252.8 Hz), 62.72 , 61.99 (dd, *J* = 22.9, 20.0 Hz), 20.54 (dd, *J* = 6.0, 3.3 Hz), 13.98. $^{19}$F NMR (376 MHz, Chloroform-*d*) δ -106.64 (d, *J* = 270.3 Hz, 1F), -111.93 (d, *J* = 270.3 Hz, 1F). HRMS (ESI) (*m/z*): [M+H]$^+$ calcd. for $C_{25}H_{23}O_3F_2$: 409.1610, found: 409.1607.

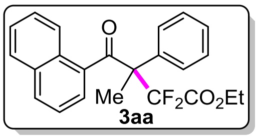

**3aa**

Ethyl (R)-2,2-difluoro-3-methyl-4-(naphthalen-1-yl)-4-oxo-3-phenylbutanoate (**3aa**). The product **3aa** is purified with silica gel chromatography (petroleum ether/ethyl acetate = 10/1 as the eluent) as yellow oil (71% yield). $^1$H NMR (400 MHz, Chloroform-*d*) δ 8.18 (d, *J* = 8.4 Hz, 1H), 7.90 – 7.78 (m, 1H), 7.61 – 7.45 (m, 7H), 7.17 – 7.09 (m, 1H), 6.86 (d, *J* = 7.2 Hz, 1H), 4.46 – 4.32 (m, 2H), 1.94 (s, 3H), 1.37 (t, *J* = 7.0 Hz, 3H). $^{13}$C NMR (101 MHz, Chloroform-*d*) δ 203.35 (d, *J* = 3.5 Hz), 163.89 (t, *J* = 32.4 Hz), 135.26, 135.12, 134.01, 131.57, 130.93, 129.29, 128.89, 128.60, 128.53, 127.83, 126.58, 125.67, 125.48, 123.85, 115.22 (dd, *J* = 263.3, 255.6 Hz), 62.97, 62.52 (dd, *J* = 22.1, 19.2 Hz), 19.21 (dd, *J* = 5.7, 3.5 Hz), 13.97. $^{19}$F NMR (376 MHz, Chloroform-*d*) δ -104.68 (d, *J* = 272.9 Hz, 1F), -108.53 (d, *J* = 272.9 Hz, 1F). HRMS (ESI) (*m/z*): [M+H]$^+$ calcd. for $C_{23}H_{21}O_3F_2$: 383.1453, found: 383.1457.

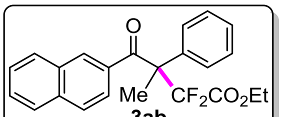

**3ab**

Ethyl (R)-2,2-difluoro-3-methyl-4-(naphthalen-2-yl)-4-oxo-3-phenylbutanoate (**3ab**). The product **3ab** is purified with silica gel chromatography (petroleum ether/ethyl acetate = 10/1 as the eluent) as yellow oil (60% yield). $^1$H NMR (400 MHz, Chloroform-*d*) δ 8.05 (s, 1H), 7.78 (d, *J* = 8.1 Hz, 1H), 7.73 – 7.66 (m, 2H), 7.57 – 7.52 (m, 2H), 7.49 – 7.44 (m, 3H), 7.41 – 7.37 (m, 3H), 4.45 – 4.33 (m, 2H), 2.21 (s, 3H), 1.40 (t, *J* = 7.1 Hz, 3H). $^{13}$C NMR (101 MHz, Chloroform-*d*) δ 199.28 (d, *J* = 4.7 Hz), 164.05 (t, *J* = 32.2 Hz), 136.26 (d, *J* = 2.0 Hz), 135.25 , 132.43 , 132.21 , 131.82 , 129.82 , 129.55 , 128.85 , 128.73 , 128.50 , 127.92 , 127.67 , 126.82 , 125.59 , 114.76 (dd, *J* = 262.7, 252.7 Hz), 62.74 , 62.12 (dd, *J* = 22.9, 20.0 Hz), 20.59 (dd, *J* = 5.9, 3.4 Hz), 14.00. $^{19}$F NMR (376 MHz, Chloroform-*d*) δ -106.64 (d, *J* = 270.3 Hz, 1F), -111.93 (d, *J* = 270.3 Hz, 1F). HRMS (ESI) (*m/z*): [M+H]$^+$ calcd. for $C_{23}H_{21}O_3F_2$: 383.1453, found: 383.1451.

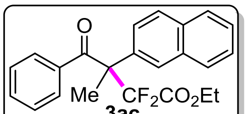

**3ac**

Ethyl (R)-2,2-difluoro-3-methyl-3-(naphthalen-2-yl)-4-oxo-4-phenylbutanoate (**3ac**). The product **3ac** is purified with silica gel chromatography (petroleum ether/ethyl acetate = 10/1 as the eluent) as yellow oil (65% yield). $^1$H NMR (400 MHz, Chloroform-*d*) δ 7.98 (s, 1H), 7.87 – 7.81 (m, 3H), 7.64 – 7.48 (m, 4H), 7.46 – 7.39 (m, 2H), 7.25 – 7.15 (m, 2H), 4.43 – 4.31 (m, 2H), 2.25 (s, 3H), 1.36 (t, *J* = 7.1 Hz, 3H). $^{13}$C NMR (101 MHz, Chloroform-*d*) δ 199.44 (d, *J* = 4.4 Hz), 164.03 (t, *J* = 32.2 Hz), 134.67, 133.62 (d, *J* = 2.1 Hz), 133.15, 133.11, 133.00, 130.24, 128.54, 128.35, 127.89, 127.71, 127.32, 127.30, 126.93, 126.55, 114.85 (dd, *J* = 262.6, 252.8 Hz), 62.77, 62.07 (dd, *J* = 22.8, 20.0 Hz), 20.67 (dd, *J* = 6.0, 3.3 Hz), 13.97. $^{19}$F NMR (376 MHz, Chloroform-*d*) δ -106.33 (d, *J* = 277.2 Hz, 1F), -111.49 (d, *J* = 270.7 Hz, 1F). HRMS (ESI) (*m/z*): [M+H]$^+$ calcd. for $C_{23}H_{21}O_3F_2$: 383.1453, found: 383.1453.

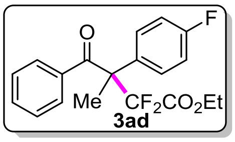


Ethyl (R)-2,2-difluoro-3-(4-fluorophenyl)-3-methyl-4-oxo-4-phenylbutanoate (**3ad**). The product **3ad** is purified with silica gel chromatography (petroleum ether/ethyl acetate = 10/1 as the eluent) as yellow oil (63% yield). $^{1}$H NMR (400 MHz, Chloroform-*d*) δ 7.49 – 7.47 (m, 2H), 7.44 (d, *J* = 7.4 Hz, 1H), 7.39 – 7.36 (m, 2H), 7.29 – 7.25 (m, 2H), 7.12 – 7.03 (m, 2H), 4.42 - 4.31 (m, 2H), 2.11 (s, 3H), 1.38 (t, *J* = 7.1 Hz, 3H). $^{13}$C NMR (101 MHz, Chloroform-*d*) δ 199.09 (d, *J* = 4.4 Hz), 163.87 (t, *J* = 32.3), 162.96 (d, *J* = 249.5), 134.38, 133.12, 131.89, 131.24 (dd, *J* = 8.2, 1.3 Hz), 130.22, 128.40, 115.52 (d, *J* = 21.5 Hz), 114.52 (dd, *J* = 262.3, 253.1 Hz), 62.81, 61.44 (dd, *J* = 22.9, 20.1 Hz), 20.64 (dd, *J* = 6.1, 3.2 Hz), 13.99. $^{19}$F NMR (376 MHz, Chloroform-*d*) δ -106.83 (d, *J* = 271.0 Hz, 1F), -112.03 (d, *J* = 271.0 Hz, 1F), -113.10 (s, 1F). HRMS (ESI) (*m/z*): [M+H]$^+$ calcd. for $C_{19}H_{18}O_3F_3$: 351.1203, found: 351.1205.

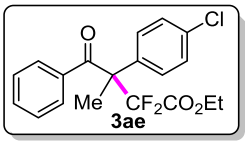


Ethyl (R)-3-(4-chlorophenyl)-2,2-difluoro-3-methyl-4-oxo-4-phenylbutanoate (**3ae**). The product **3ae** is purified with silica gel chromatography (petroleum ether/ethyl acetate = 10/1 as the eluent) as yellow oil (70% yield). $^{1}$H NMR (400 MHz, Chloroform-*d*) δ 7.50 – 7.43 (m, 3H), 7.37 – 7.23 (m, 6H), 4.42 – 4.30 (m, 2H), 2.11 (s, 3H), 1.37 (t, *J* = 7.1 Hz, 3H). $^{13}$C NMR (101 MHz, Chloroform-*d*) δ 198.91 (d, *J* = 4.4 Hz), 163.78 (t, *J* = 32.1 Hz), 134.97, 134.68 (d, J = 2.1 Hz), 134.23 (d, *J* = 2.1 Hz), 133.19, 130.81, 130.21, 128.69, 128.42, 114.44 (dd, *J* = 262.4, 253.4 Hz), 62.84, 61.54 (dd, *J* = 22.9, 20.0 Hz), 20.51 (dd, *J* = 6.0, 3.2 Hz), 13.97. $^{19}$F NMR (376 MHz, Chloroform-*d*) δ -106.70 (d, *J* = 271.3 Hz, 1F), -111.83 (d, *J* = 271.2 Hz, 1F). HRMS (ESI) (*m/z*): [M+H]$^+$ calcd. for $C_{19}H_{18}O_3ClF_2$: 367.0907, found: 367.0907.

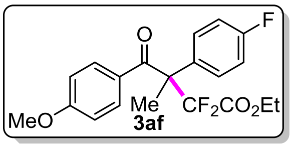


Ethyl (R)-2,2-difluoro-3-(4-fluorophenyl)-4-(4-methoxyphenyl)-3-methyl-4-oxobutanoate (**3af**). The product **3af** is purified with silica gel chromatography (petroleum ether/ethyl acetate = 10/1 as the eluent) as yellow oil (74% yield). $^{1}$H NMR (400 MHz, Chloroform-*d*) δ 7.50 (d, *J* = 8.8 Hz, 2H), 7.37 – 7.34 (m, 2H), 7.11 – 7.00 (m, 2H), 6.74 (d, *J* = 8.9 Hz, 2H), 4.42 – 4.31 (m, 2H), 3.79 (s, 3H), 2.13 (s, 3H), 1.38 (t, *J* = 7.1 Hz, 3H). $^{13}$C NMR (101 MHz, Chloroform-*d*) δ 197.55 (d, *J* = 4.8 Hz), 164.01 (t, *J* = 32.3 Hz), 163.42, 162.86 (d, *J* = 249.5 Hz), 132.81, 132.42, 131.32 (d, *J* = 8.0 Hz), 126.65, 115.48, 115.27, 114.50 (dd, J = 212.1, 254.5 Hz), 113.61, 62.73, 61.43 (dd, *J* = 22.9, 20.0 Hz), 55.57, 20.94 (dd, *J* = 6.0, 3.2 Hz), 14.00. $^{19}$F NMR (376 MHz, Chloroform-*d*) δ -107.08 (d, *J* = 270.1 Hz, 1F), -112.37 (d, J = 270.0 Hz, 1F), -113.36 (s, 1F). HRMS (ESI) (*m/z*): [M+H]$^+$ calcd. for $C_{20}H_{20}O_4F_3$: 381.1308, found: 381.1306.

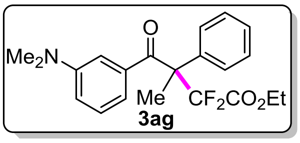


Ethyl (R)-4-(3-(dimethylamino)phenyl)-2,2-difluoro-3-methyl-4-oxo-3-phenylbutanoate (**3ag**). The product **3ag** is purified with silica gel chromatography (petroleum ether/ethyl acetate = 10/1 as the eluent)

as yellow oil (71% yield). $^{1}$H NMR (400 MHz, Chloroform-*d*) δ 7.41 – 7.36 (m, 5H), 7.10 – 7.02 (m, 1H), 6.90 (s, 1H), 6.78 – 6.72 (m, 2H), 4.36 (q, *J* = 7.0 Hz, 2H), 2.79 (s, 6H), 2.13 (s, 3H), 1.37 (t, *J* = 7.1 Hz, 3H). $^{13}$C NMR (101 MHz, Chloroform-*d*) δ 200.02 (d, *J* = 4.4 Hz), 164.07 (t, *J* = 32.3 Hz), 150.16 , 136.53 , 135.13 , 129.52 , 128.76 , 128.51 , 128.30 , 118.40 , 116.70 , 114.80 (dd, *J* = 262.1, 252.5 Hz), 114.02 , 62.66 , 62.10 (dd, *J* = 22.8, 19.9 Hz), 40.36 , 20.53 (dd, *J* = 6.0, 3.4 Hz), 13.98. $^{19}$F NMR (376 MHz, Chloroform-*d*) δ -106.77 (d, *J* = 269.9 Hz, 1F), -111.85 (d, *J* = 269.9 Hz, 1F). HRMS (ESI) (*m/z*): $[M+H]^+$ calcd. for $C_{21}H_{24}O_3NF_2$: 376.1719, found: 376.1720.

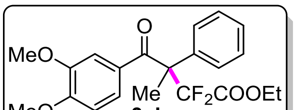

**3ah**

Ethyl (R)-4-(3,4-dimethoxyphenyl)-2,2-difluoro-3-methyl-4-oxo-3-phenylbutanoate (**3ah**). The product **3ah** is purified with silica gel chromatography (petroleum ether/ethyl acetate = 10/1 as the eluent) as yellow oil (65% yield). $^{1}$H NMR (400 MHz, Chloroform-*d*) δ 7.39 – 7.37 (m, 5H), 7.19 (s, 1H), 7.03 (d, *J* = 8.5 Hz, 1H), 6.63 (d, *J* = 8.6 Hz, 1H), 4.42 – 4.30 (m, 2H), 3.84 (s, 3H), 3.68 (s, 3H), 2.16 (s, 3H), 1.37 (t, *J* = 7.1 Hz, 3H). $^{13}$C NMR (101 MHz, Chloroform-*d*) δ 197.80 (d, *J* = 4.8 Hz), 164.09 (t, *J* = 32.2 Hz), 153.01, 148.41, 136.75, 129.57, 128.54, 128.29, 126.74 (d, *J* = 1.7 Hz), 125.32, 114.68 (dd, *J* = 262.6, 252.0 Hz).112.78, 109.79, 62.66, 61.94 (dd, *J* = 23.0, 20.1 Hz), 56.07, 55.77, 20.90 (dd, *J* = 5.8, 3.4 Hz), 13.98. $^{19}$F NMR (376 MHz, Chloroform-*d*) δ -107.09 (d, *J* = 269.4 Hz, 1F), -112.26 (d, *J* = 269.4 Hz, 1F). HRMS (ESI) (*m/z*): $[M+H]^+$ calcd. for $C_{21}H_{22}O_5F_2Na$: 415.1328, found: 415.1324.

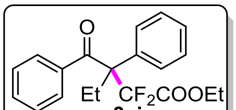

**3ai**

Ethyl (R)-3-benzoyl-2,2-difluoro-3-phenylpentanoate (**3ai**). The product **3ai** is purified with silica gel chromatography (petroleum ether/ethyl acetate = 10/1 as the eluent) as yellow oil (40% yield). $^{1}$H NMR (400 MHz, Chloroform-*d*) δ 7.44 (d, *J* = 7.9 Hz, 2H), 7.37 – 7.27 (m, 6H), 7.19 – 7.15 (m, 2H), 4.19 – 4.06 (m, 2H), 2.87 (dq, *J* = 14.8, 7.5 Hz, 1H), 2.37 (dq, *J* = 14.5, 6.9, 6.4 Hz, 1H), 1.15 (t, *J* = 7.1 Hz, 3H), 0.88 (t, *J* = 7.4 Hz, 3H). $^{13}$C NMR (101 MHz, Chloroform-*d*) δ 197.93, 163.66 (t, *J* = 32.1 Hz), 137.03 (t, *J* = 2.6 Hz), 136.26, 132.77, 129.88, 129.79, 128.50, 128.25, 128.15, 116.40 (dd, *J* = 264.8, 258.7 Hz), 64.97 (t, *J* = 20.2 Hz), 62.72, 27.86 (t, *J* = 2.6 Hz), 13.83, 10.56 (dd, *J* = 5.4, 2.1 Hz). $^{19}$F NMR (376 MHz, Chloroform-*d*) δ -105.83 (d, *J* = 262.3 Hz, 1F), -108.36 (d, *J* = 262.3 Hz, 1F). HRMS (ESI) (*m/z*): $[M+H]^+$ calcd. for $C_{20}H_{20}O_3F_2Na$: 369.1273, found: 369.1282.

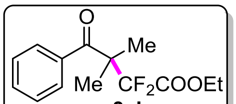

**3aj**

Ethyl 2,2-difluoro-3,3-dimethyl-4-oxo-4-phenylbutanoate (**3aj**). The product **3aj** is purified with silica gel chromatography (petroleum ether/ethyl acetate = 10/1 as the eluent) as yellow oil (41% yield). $^{1}$H NMR (400 MHz, Chloroform-*d*) δ 7.70 (d, *J* = 8.3 Hz, 2H), 7.54 – 7.48 (m, 1H), 7.47 – 7.39 (m, 2H), 4.35 (q, *J* = 7.1 Hz, 2H), 1.62 (s, 6H), 1.36 (td, *J* = 7.1, 1.1 Hz, 3H). $^{13}$C NMR (101 MHz, Chloroform-*d*) δ 203.63, 163.73 (t, *J* = 32.3 Hz), 137.61, 131.87, 128.40, 128.14, 116.28 (t, *J* = 257.2 Hz), 62.88, 54.87 (t, *J* = 21.8 Hz), 21.28 (t, *J* = 4.5 Hz), 14.01. $^{19}$F NMR (376 MHz, Chloroform-*d*) δ -111.40. $[M+H]^+$ calcd. for $C_{14}H_{16}F_2NaO_3$: 293.0965, found: 293.0956.

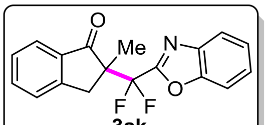

**3ak**

(S)-2-(benzo[d]oxazol-2-yldifluoromethyl)-2-methyl-2,3-dihydro-1H-inden-1-one (**3ak**). The product **3ak** is purified with silica gel chromatography (petroleum ether/ethyl acetate = 10/1 as the eluent) as yellow oil (57% yield). $^{1}$H NMR (400 MHz, Chloroform-*d*) δ 7.79 (d, *J* = 7.7 Hz, 1H), 7.74 (d, *J* = 7.4 Hz, 1H), 7.63 – 7.57 (m, 2H), 7.49 – 7.33 (m, 4H), 3.83 (d, *J* = 17.6 Hz, 1H), 3.07 (d, *J* = 17.6 Hz, 1H), 1.64 (s, 3H). $^{13}$C NMR (101 MHz, Chloroform-*d*) δ 202.43 (t, *J* = 2.2 Hz), 156.64 (t, *J* = 33.9 Hz), 151.56, 150.51, 139.98, 135.58, 135.36, 128.02, 126.88, 126.59, 125.30, 124.94, 121.43, 117.14 (t, *J* = 249.5 Hz), 111.50, 55.00 (t, *J* = 22.6 Hz), 37.47 (t, *J* = 2.8 Hz), 19.33 (t, *J* = 4.5 Hz). $^{19}$F NMR (376 MHz, Chloroform-*d*) δ -102.04 (d, *J* = 277.0 Hz, 1F), -105.22 (d, *J* = 277.1 Hz, 1F). HRMS (ESI) (*m/z*): [M+H]$^+$ calcd. for $C_{18}H_{14}F_2NO_2$: 314.0987, found: 314.1005.

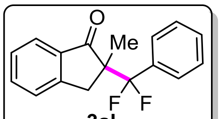

**3al**

(S)-2-(difluoro(phenyl)methyl)-2-methyl-2,3-dihydro-1H-inden-1-one (**3al**). The product **3al** is purified with silica gel chromatography (petroleum ether/ethyl acetate = 10/1 as the eluent) as yellow oil (60% yield). $^{1}$H NMR (400 MHz, Chloroform-*d*) δ 7.71 (d, *J* = 7.7 Hz, 1H), 7.59 – 7.52 (m, 1H), 7.46 – 7.30 (m, 7H), 3.67 (d, *J* = 17.7 Hz, 1H), 2.91 (d, *J* = 17.7 Hz, 1H), 1.45 (s, 3H). $^{13}$C NMR (101 MHz, Chloroform-*d*) δ 203.81 (t, *J* = 2.9 Hz), 151.77, 135.91 (t, *J* = 2.0 Hz), 135.34, 134.40 (t, *J* = 26.9 Hz), 129.99, 127.87, 127.76, 126.82 (t, *J* = 6.7 Hz), 126.31, 124.56, 122.59 (dd, *J* = 251.6, 249.7 Hz). 56.06 (t, *J* = 26.2 Hz), 37.37 (t, *J* = 3.1 Hz), 19.78 (t, *J* = 4.5 Hz). $^{19}$F NMR (376 MHz, Chloroform-*d*) δ -98.28 (d, *J* = 249.5 Hz, 1F), -101.63 (d, *J* = 249.5 Hz, 1F). HRMS (ESI) (*m/z*): [M+Na]$^+$ calcd. for $C_{17}H_{14}F_2ONa$: 295.0905, found: 295.0923.

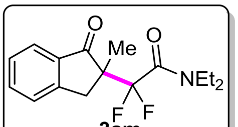

**3am**

(S)-N,N-diethyl-2,2-difluoro-2-(2-methyl-1-oxo-2,3-dihydro-1H-inden-2-yl)acetamide (**3am**). The product **3am** is purified with silica gel chromatography (petroleum ether/ethyl acetate = 10/1 as the eluent) as yellow oil (53% yield). $^{1}$H NMR (400 MHz, Chloroform-*d*) δ 7.81 (d, *J* = 7.6 Hz, 1H), 7.60 – 7.52 (m, 1H), 7.45 – 7.33 (m, 2H), 3.65 – 3.47 (m, 2H), 3.42 (d, *J* = 16.9 Hz, 1H), 3.30 – 3.21 (m, 2H), 2.89 (d, *J* = 16.9 Hz, 1H), 1.40 (s, 3H), 1.22 (t, *J* = 7.0 Hz, 3H), 1.05 (t, *J* = 7.1 Hz, 3H). $^{13}$C NMR (101 MHz, Chloroform-*d*) δ 203.26 (d, *J* = 6.2 Hz), 161.44 (t, *J* = 29.6 Hz), 150.53, 135.83 (dd, *J* = 4.9, 1.1 Hz), 134.55, 127.64, 126.57, 124.70, 120.77 (dd, *J* = 266.8, 261.4 Hz), 53.65 (dd, *J* = 21.5, 19.6 Hz), 41.55 (t, *J* = 6.4 Hz), 41.24, 38.97 (dd, *J* = 4.2, 2.2 Hz), 18.88 (dd, *J* = 8.2, 3.5 Hz), 14.30, 12.38. $^{19}$F NMR (376 MHz, Chloroform-*d*) δ -100.84 (d, *J* = 292.3 Hz, 1F), -103.17 (d, *J* = 292.3 Hz, 1F). HRMS (ESI) (*m/z*): [M+H]$^+$ calcd. for $C_{16}H_{20}NO_2F_2$: 296.1457, found: 296.1467.

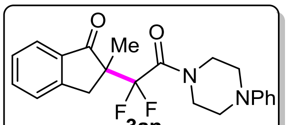

**3an**

(S)-2-(1,1-difluoro-2-oxo-2-(4-phenylpiperazin-1-yl)ethyl)-2-methyl-2,3-dihydro-1H-inden-1-one (**3an**). The product **3an** is purified with silica gel chromatography (petroleum ether/ethyl acetate = 10/1 as the eluent) as yellow oil (50% yield). $^1$H NMR (400 MHz, Chloroform-*d*) δ 7.83 (d, *J* = 7.6 Hz, 1H), 7.62 – 7.55 (m, 1H), 7.46 – 7.36 (m, 2H), 7.32 – 7.23 (m, 2H), 6.95 – 6.88 (m, 3H), 3.96 – 3.87 (m, 2H), 3.73 – 3.64 (m, 2H), 3.44 (d, *J* = 17.1 Hz, 1H), 3.30 – 3.07 (m, 4H), 2.92 (d, *J* = 17.0 Hz, 1H), 1.43 (s, 3H). $^{13}$C NMR (101 MHz, Chloroform-*d*) δ 203.21 (d, *J* = 6.2 Hz), 160.67 (t, *J* = 30.0 Hz), 150.82, 150.47, 135.77 (d, *J* = 5.1 Hz), 134.72, 129.37, 127.74, 126.61, 124.73, 120.83, 120.70 (dd, *J* = 266.7, 261.0 Hz), 116.83, 53.52 (dd, *J* = 21.0, 19.3 Hz), 49.85, 49.40, 45.67 (t, *J* = 6.3 Hz), 43.14, 38.84 (dd, J = 4.1, 2.1 Hz), 18.82 (dd, *J* = 8.2, 3.3 Hz). $^{19}$F NMR (376 MHz, Chloroform-*d*) δ -100.05 (d, *J* = 294.3 Hz, 1F), -102.16 (d, *J* = 294.3 Hz, 1F). HRMS (ESI) (*m/z*): [M+Na]$^+$ calcd. for $C_{22}H_{22}N_2O_2F_2Na$: 407.1542, found: 407.1545.

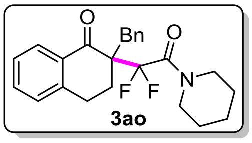


(R)-2-benzyl-2-(1,1-difluoro-2-oxo-2-(piperidin-1-yl)ethyl)-3,4-dihydronaphthalen-1(2H)-one (**3ao**). The product **3ao** is purified with silica gel chromatography (petroleum ether/ethyl acetate = 10/1 as the eluent) as yellow oil (80% yield). $^1$H NMR (400 MHz, Chloroform-*d*) δ 8.02 (d, *J* = 7.8 Hz, 1H), 7.40 – 7.32 (m, 1H), 7.26 – 7.11 (m, 6H), 7.07 (d, *J* = 7.6 Hz, 1H), 3.64 – 3.42 (m, 3H), 3.31 – 3.27 (m, 1H), 3.16 (s, 2H), 2.80 – 2.76 (m, 2H), 2.45 (td, *J* = 12.8, 6.4 Hz, 1H), 2.08 (d, *J* = 14.1 Hz, 1H), 1.55 - 1.42 (m, 6H). $^{13}$C NMR (101 MHz, Chloroform-*d*) δ 196.07 (d, *J* = 6.2 Hz), 160.57 (t, *J* = 29.4 Hz), 141.57, 136.29, 133.23, 132.79 (d, *J* = 3.6 Hz), 130.89, 128.44, 128.18, 127.87, 126.91 (d, *J* = 6.7 Hz), 121.38 (dd, *J* = 273.5, 262.6 Hz), 54.81 (dd, *J* = 19.9, 15.4 Hz), 46.93 (t, *J* = 7.0 Hz), 44.70, 36.65, 36.56, 26.58, 26.15 (d, *J* = 6.2 Hz), 25.70, 25.09, 24.50. $^{19}$F NMR (376 MHz, Chloroform-*d*) δ -95.09 (d, *J* = 288.8 Hz, 1F), -100.86 (d, *J* = 288.8 Hz, 1F). HRMS (ESI) (*m/z*): [M+H]$^+$ calcd. for $C_{24}H_{26}O_2F_2N$: 398.1926, found: 398.1931.

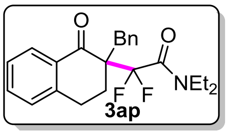


(R)-2-(2-benzyl-1-oxo-1,2,3,4-tetrahydronaphthalen-2-yl)-N,N-diethyl-2,2-difluoroacetamide (**3ap**). The product **3ap** is purified with silica gel chromatography (petroleum ether/ethyl acetate = 10/1 as the eluent) as yellow oil (78% yield). $^1$H NMR (400 MHz, Chloroform-*d*) δ 8.09 (d, *J* = 7.8 Hz, 1H), 7.46 – 7.39 (m, 1H), 7.32 – 7.20 (m, 6H), 7.14 (d, *J* = 7.6 Hz, 1H), 3.61 (dq, *J* = 14.0, 7.1 Hz, 1H), 3.42 (ddt, *J* = 28.0, 14.0, 7.0 Hz, 2H), 3.28 – 3.13 (m, 3H), 2.94 – 2.80 (m, 2H), 2.63 – 2.55 (m, 1H), 2.15 (d, *J* = 14.2 Hz, 1H), 1.22 (t, *J* = 7.0 Hz, 3H), 1.05 (t, *J* = 7.1 Hz, 3H). $^{13}$C NMR (101 MHz, Chloroform-*d*) δ 196.16 (d, *J* = 5.9 Hz), 161.67 (t, *J* = 29.6 Hz), 141.75, 136.26, 133.28, 132.65 (d, *J* = 3.3 Hz), 130.89, 128.46, 128.16, 127.85, 126.93, 126.85, 121.09 (dd, *J* = 273.2, 262.1 Hz), 54.96 (dd, *J* = 20.1, 15.7 Hz), 41.75 (t, *J* = 6.7 Hz), 41.47, 36.60 (d, *J* = 8.3 Hz), 26.17 (d, *J* = 5.0 Hz), 25.11, 14.39, 12.37. $^{19}$F NMR (376 MHz, Chloroform-*d*) δ -95.57 (d, *J* = 287.3 Hz, 1F), -101.59 (d, *J* = 287.3 Hz, 1F). HRMS (ESI) (*m/z*): [M+H]$^+$ calcd. for $C_{23}H_{26}O_2F_2N$: 386.1926, found: 386.1931.

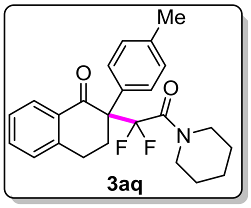

3aq

(R)-2-(1,1-difluoro-2-oxo-2-(piperidin-1-yl)ethyl)-2-(p-tolyl)-3,4-dihydronaphthalen-1(2H)-one (**3aq**). The product **3aq** is purified with silica gel chromatography (petroleum ether/ethyl acetate = 10/1 as the eluent) as yellow oil (75% yield). $^1$H NMR (400 MHz, Chloroform-*d*) δ 8.15 (d, *J* = 7.8 Hz, 1H), 7.32 – 7.17 (m, 4H), 7.06 (d, *J* = 7.8 Hz, 2H), 6.98 (d, *J* = 7.6 Hz, 1H), 3.49 (d, *J* = 38.6 Hz, 4H), 3.16 (td, *J* = 14.2, 5.9 Hz, 1H), 2.73 – 2.62 (m, 3H), 2.22 (s, 3H), 1.55 – 1.48 (m, 6H). $^{13}$C NMR (101 MHz, Chloroform-*d*) δ 194.50 (dd, *J* = 6.8, 1.6 Hz), 160.89 (t, *J* = 29.4 Hz), 142.50, 138.04, 133.43, 132.27 (d, *J* = 4.0 Hz), 129.52, 129.24, 128.63 (d, *J* = 4.8 Hz), 128.44, 128.37, 126.71, 118.85 (dd, *J* = 272.1, 257.5 Hz), 59.35 (dd, *J* = 21.4, 17.1 Hz), 47.00 (dd, *J* = 8.5, 5.6 Hz), 44.69, 28.88 (dd, *J* = 6.1, 1.7 Hz), 26.51, 25.77, 25.47, 24.55, 21.04. $^{19}$F NMR (376 MHz, Chloroform-*d*) δ -91.09 (d, *J* = 293.0 Hz, 1F), -104.98 (d, *J* = 293.0 Hz, 1F). HRMS (ESI) (*m/z*): [M+Na]$^+$ calcd. for $C_{24}H_{25}O_2F_2NNa$: 420.1746, found: 420.1746.

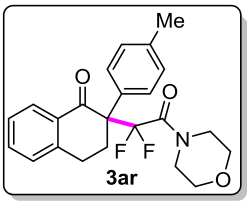

3ar

(R)-2-(1,1-difluoro-2-morpholino-2-oxoethyl)-2-(p-tolyl)-3,4-dihydronaphthalen-1(2H)-one (**3ar**). The product **3ar** is purified with silica gel chromatography (petroleum ether/ethyl acetate = 10/1 as the eluent) as yellow oil (75% yield). $^1$H NMR (400 MHz, Chloroform-*d*) δ 8.22 (d, *J* = 7.8 Hz, 1H), 7.44 – 7.37 (m, 1H), 7.32 – 7.26 (m, 3H), 7.15 (d, *J* = 7.9 Hz, 2H), 7.08 (d, *J* = 7.6 Hz, 1H), 3.78 – 3.62 (m, 8H), 3.22 (td, *J* = 14.0, 5.5 Hz, 1H), 2.84 – 2.72 (m, 3H), 2.31 (s, 3H). $^{13}$C NMR (101 MHz, Chloroform-*d*) δ 194.63 (dd, *J* = 6.7, 1.4 Hz), 161.28 (t, *J* = 29.9 Hz), 142.55, 138.22, 133.62, 132.09 (d, *J* = 3.9 Hz), 129.43, 129.30, 128.51, 128.32, 128.23 (d, *J* = 4.8 Hz), 126.78, 118.53 (dd, *J* = 270.8, 256.5 Hz), 66.79, 59.34 (dd, *J* = 21.2, 17.1 Hz), 46.72 (dd, *J* = 8.2, 5.2 Hz), 43.58, 28.75 (dd, *J* = 6.1, 1.8 Hz), 25.42, 21.05. $^{19}$F NMR (376 MHz, Chloroform-*d*) δ -91.36 (d, *J* = 293.9 Hz, 1F), -104.98 (d, *J* = 293.8 Hz, 1F). HRMS (ESI) (*m/z*): [M+Na]$^+$ calcd. for $C_{23}H_{23}O_3F_2NNa$: 422.1538, found: 422.1539.

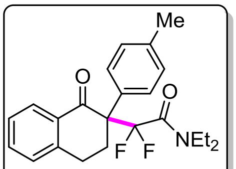

3as

(R)-N,N-diethyl-2,2-difluoro-2-(1-oxo-2-(p-tolyl)-1,2,3,4-tetrahydronaphthalen-2-yl)acetamide (**3as**). The product **3as** is purified with silica gel chromatography (petroleum ether/ethyl acetate = 10/1 as the eluent) as yellow oil (81% yield). $^1$H NMR (400 MHz, Chloroform-*d*) δ 8.13 (d, *J* = 7.9 Hz, 1H), 7.34 – 7.27 (m, 1H), 7.25 – 7.17 (m, 3H), 7.07 (d, *J* = 8.0 Hz, 2H), 6.99 (d, *J* = 7.6 Hz, 1H), 3.58 – 3.10 (m, 5H), 2.74 – 2.59 (m, 3H), 2.23 (s, 3H), 1.11 (t, *J* = 7.0 Hz, 3H), 1.05 (t, *J* = 7.1 Hz, 3H). $^{13}$C NMR (101 MHz, Chloroform-*d*) δ 194.78 (dd, *J* = 6.7, 1.5 Hz), 162.17 (t, *J* = 29.5 Hz), 142.64, 138.05, 133.48, 132.30 (d, *J* = 3.8 Hz), 129.46, 129.29, 128.95 (d, *J* = 5.1 Hz), 128.48, 128.31, 126.74, 118.69 (dd, *J* = 272.0, 256.6

Hz), 59.68 (dd, $J$ = 21.6, 17.7 Hz), 41.93 (dd, $J$ = 8.4, 5.1 Hz), 41.80, 29.15 (dd, $J$ = 6.3, 1.4 Hz), 25.56, 21.07, 14.36, 12.40. $^{19}$F NMR (376 MHz, Chloroform-$d$) δ -91.80 (d, $J$ = 290.5 Hz, 1F), -105.58 (d, $J$ = 290.4 Hz, 1F). HRMS (ESI) ($m/z$): [M+Na]$^+$ calcd. for $C_{23}H_{25}O_2F_2NNa$: 408.1746, found: 408.1756.

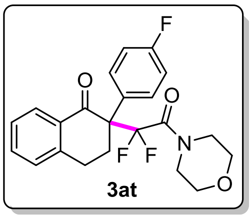


(R)-2-(1,1-difluoro-2-morpholino-2-oxoethyl)-2-(4-fluorophenyl)-3,4-dihydronaphthalen-1(2H)-one (**3at**). The product **3at** is purified with silica gel chromatography (petroleum ether/ethyl acetate = 10/1 as the eluent) as yellow oil (83% yield). $^1$H NMR (400 MHz, Chloroform-$d$) δ 8.21 (d, $J$ = 7.9 Hz, 1H), 7.45 – 7.38 (m, 3H), 7.35 – 7.28 (m, 1H), 7.10 (d, $J$ = 7.6 Hz, 1H), 7.07 – 6.98 (m, 2H), 3.78 – 3.61 (m, 8H), 3.26 – 3.18 (m, 1H), 2.86 – 2.67 (m, 3H). $^{13}$C NMR (101 MHz, Chloroform-$d$) δ 194.43 (dd, $J$ = 6.6, 1.4 Hz), 162.74 (d, $J$ = 248.3 Hz)., 161.14 (t, $J$ = 29.7 Hz), 142.41, 133.88, 131.99 (d, $J$ = 4.0 Hz), 131.40 (d, $J$ = 8.1 Hz), 128.51 (d, $J$ = 14.0 Hz), 127.22 (dd, $J$ = 4.0, 4.0 Hz), 126.98, 118.50 (dd, $J$ = 271.5, 256.9 Hz), 115.65, 115.44, 66.82, 59.16 (dd, $J$ = 21.2, 17.0 Hz), 46.76 (dd, $J$ = 8.2, 5.2 Hz), 43.65, 28.97 (dd, $J$ = 6.1, 1.7 Hz), 25.32. $^{19}$F NMR (376 MHz, Chloroform-$d$) δ -91.38 (d, $J$ = 294.4 Hz, 1F), -104.88 (d, $J$ = 294.3 Hz, 1F), -113.83 (s, 1F). HRMS (ESI) ($m/z$): [M+Na]$^+$ calcd. for $C_{22}H_{20}O_3F_3NNa$: 426.1287, found: 426.1293.

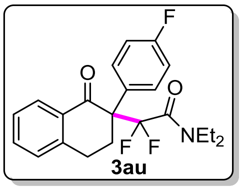


(R)-N,N-diethyl-2,2-difluoro-2-(2-(4-fluorophenyl)-1-oxo-1,2,3,4-tetrahydronaphthalen-2-yl)acetamide (**3au**). The product **3au** is purified with silica gel chromatography (petroleum ether/ethyl acetate = 10/1 as the eluent) as yellow oil (83% yield). $^1$H NMR (400 MHz, Chloroform-$d$) δ 8.21 (d, $J$ = 7.8 Hz, 1H), 7.58 – 7.36 (m, 3H), 7.35 – 7.27 (m, 1H), 7.09 (d, $J$ = 7.6 Hz, 1H), 7.07 – 6.95 (m, 2H), 3.65 – 3.19 (m, 5H), 2.84 – 2.65 (m, 3H), 1.20 (t, $J$ = 6.9 Hz, 3H), 1.13 (t, $J$ = 7.0 Hz, 3H). $^{13}$C NMR (101 MHz, Chloroform-$d$) δ 194.55 (dd, $J$ = 6.6, 1.6 Hz), 162.64 (d, $J$ = 248.5 Hz), 161.93 (t, $J$ = 29.4 Hz), 142.44, 133.71, 132.11 (d, $J$ = 4.0 Hz), 131.41, 13133, 128.52, 128.38, 127.82 (dd, $J$ = 5.1, 3.1 Hz), 126.90, 118.61 (dd, $J$ = 272.5, 257.0 Hz), 115.57, 115.36, 59.40 (dd, $J$ = 21.7, 17.4 Hz), 41.93 (dd, $J$ = 8.3, 5.2 Hz), 41.80, 29.30 (dd, $J$ = 4.0, 2.1 Hz), 25.40, 14.35, 12.38. $^{19}$F NMR (376 MHz, Chloroform-$d$) δ -91.79 (d, $J$ = 291.2 Hz, 1F), -105.45 (d, $J$ = 291.1 Hz, 1F), -114.13 (s, 1F). HRMS (ESI) ($m/z$): [M+Na]$^+$ calcd. for $C_{22}H_{22}O_2F_3NNa$: 412.1495, found: 412.1497.

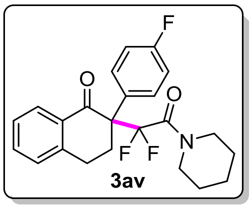


(R)-2-(1,1-difluoro-2-oxo-2-(piperidin-1-yl)ethyl)-2-(4-fluorophenyl)-3,4-dihydronaphthalen-1(2H)-one (**3av**). The product **3av** is purified with silica gel chromatography (petroleum ether/ethyl acetate = 10/1 as the eluent) as yellow oil (77% yield). $^1$H NMR (400 MHz, Chloroform-$d$) δ 8.22 (d, $J$ = 7.8 Hz, 1H),

7.43 – 7.40 (m, 3H), 7.35 – 7.28 (m, 1H), 7.09 (d, *J* = 7.6 Hz, 1H), 7.07 – 6.97 (m, 2H), 3.53 – 3.36 (m, 4H), 3.29 – 3.21 (m, 1H), 2.85 – 2.65 (m, 3H), 1.76 – 1.44 (m, 6H). $^{13}$C NMR (101 MHz, Chloroform-*d*) δ 194.37 (dd, *J* = 6.8, 1.7 Hz), 162.70 (d, *J* = 248.0 Hz), 160.73 (t, *J* = 29.2 Hz), 142.37, 133.72, 132.15 (d, *J* = 3.9 Hz), 131.49 (d, *J* = 8.0 Hz), 128.52, 127.57 (dd, *J* = 5.1, 4.0 Hz), 126.93, 118.83 (dd, *J* = 272.7, 257.6 Hz), 115.48 (d, *J* = 21.3 Hz), 59.14 (dd, *J* = 21.4, 17.1 Hz), 47.06 (dd, *J* = 8.4, 5.7 Hz), 44.77, 29.10 (dd, *J* = 6.1, 1.9 Hz), 26.57, 25.80, 25.38, 24.58. $^{19}$F NMR (376 MHz, Chloroform-*d*) δ -91.15 (d, *J* = 293.3 Hz, 1F), -104.86 (d, *J* = 293.2 Hz, 1F), -114.18 (s, 1F). HRMS (ESI) (*m/z*): [M+Na]$^+$ calcd. for $C_{23}H_{22}O_2F_3NNa$: 424.1495, found: 424.1497.

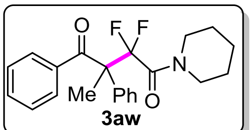

**3aw**

2,2-difluoro-3-methyl-3,4-diphenyl-1-(piperidin-1-yl)butane-1,4-dione (**3aw**). The product **3aw** is purified with silica gel chromatography (petroleum ether/ethyl acetate = 10/1 as the eluent) as yellow oil (65% yield). $^1$H NMR (400 MHz, Chloroform-*d*) δ 7.55 – 7.49 (m, 2H), 7.45 – 7.33 (m, 6H), 7.27 – 7.20 (m, 2H), 3.67 – 3.48 (m, 4H), 2.05 (s, 3H), 1.73 – 1.48 (m, 6H). $^{13}$C NMR (101 MHz, Chloroform-*d*) δ 200.26 (dd, *J* = 5.1, 2.1 Hz), 160.89 (t, *J* = 29.0 Hz), 137.76 (d, *J* = 3.5 Hz), 136.13 (d, *J* = 3.1 Hz), 131.45, 129.40, 128.95, 128.49, 128.36, 127.99, 118.54 (dd, *J* = 272.1, 257.8 Hz), 60.38 (dd, *J* = 21.4, 17.2 Hz), 47.06 (dd, *J* = 8.9, 5.6 Hz), 44.78, 26.55, 25.80, 24.60, 21.41 (dd, *J* = 6.0, 3.1 Hz). $^{19}$F NMR (376 MHz, Chloroform-*d*) δ -96.42 (d, *J* = 285.6 Hz, 1F), -104.50 (d, *J* = 285.7 Hz, 1F). HRMS (ESI) (*m/z*): [M+H]$^+$ calcd. for $C_{22}H_{24}O_2F_2N$: 372.1770, found: 372.1774.

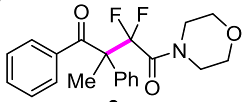

**3ax**

2,2-difluoro-3-methyl-1-morpholino-3,4-diphenylbutane-1,4-dione (**3ax**). The product **3ax** is purified with silica gel chromatography (petroleum ether/ethyl acetate = 10/1 as the eluent) as yellow oil (65% yield). $^1$H NMR (400 MHz, Chloroform-*d*) δ 7.53 – 7.46 (m, 2H), 7.45 – 7.33 (m, 6H), 7.29 – 7.22 (m, 2H), 3.82 – 3.58 (m, 8H), 2.06 (s, 3H). $^{13}$C NMR (101 MHz, Chloroform-*d*) δ 199.97 (dd, *J* = 5.1, 1.1 Hz), 161.37 (t, *J* = 29.4 Hz), 137.18 (d, *J* = 3.5 Hz), 135.82 (d, *J* = 4.1 Hz), 131.79, 129.42, 129.14, 128.57, 128.55, 128.09, 118.24 (dd, *J* = 271.1, 256.6 Hz), 66.87, 60.49 (dd, *J* = 21.4, 17.1 Hz), 46.85 (dd, *J* = 9.0, 5.1 Hz), 43.69, 21.17 (dd, *J* = 5.8, 3.2 Hz). $^{19}$F NMR (376 MHz, Chloroform-*d*) δ -95.48 (d, *J* = 287.4 Hz, 1F), -103.97 (d, *J* = 287.4 Hz, 1F). HRMS (ESI) (*m/z*): [M+H]$^+$ calcd. for $C_{21}H_{22}O_3F_2N$: 374.1562, found: 374.1567.

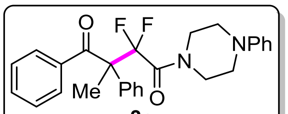

**3ay**

2,2-difluoro-3-methyl-3,4-diphenyl-1-(4-phenylpiperazin-1-yl)butane-1,4-dione (**3ay**). The product **3ay** is purified with silica gel chromatography (petroleum ether/ethyl acetate = 10/1 as the eluent) as yellow oil (60% yield). $^1$H NMR (400 MHz, Chloroform-*d*) δ 7.56 – 7.19 (m, 12H), 6.98 – 6.83 (m, 3H), 3.94 – 3.69 (m, 4H), 3.37 – 3.03 (m, 4H), 2.07 (s, 3H). $^{13}$C NMR (101 MHz, Chloroform-*d*) δ 200.02 (d, *J* = 4.7 Hz), 161.30 (t, *J* = 29.5 Hz), 150.93, 137.28 (d, *J* = 2.1 Hz), 135.90 (d, *J* = 3.1 Hz), 131.76, 129.44, 129.34, 129.14, 128.59, 128.54, 128.09, 120.63, 118.31 (dd, *J* = 271.3, 256.8 Hz), 116.70, 60.50 (dd, *J* = 21.4,

17.1 Hz), 49.82, 49.38, 45.97 (dd, $J$ = 9.1, 4.9 Hz), 43.37, 21.24 (dd, $J$ = 5.8, 3.1 Hz). $^{19}$F NMR (376 MHz, Chloroform-*d*) δ -95.09 (d, $J$ = 287.1 Hz, 1F), -103.68 (d, $J$ = 287.1 Hz, 1F). HRMS (ESI) (*m/z*): [M+Na]$^+$ calcd. for $C_{27}H_{26}N_2O_2F_2Na$: 471.1855, found: 471.1855.

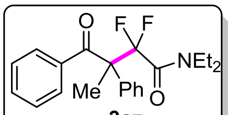

**3az**

N,N-diethyl-2,2-difluoro-3-methyl-4-oxo-3,4-diphenylbutanamide (**3az**). The product **3az** is purified with silica gel chromatography (petroleum ether/ethyl acetate = 10/1 as the eluent) as yellow oil (60% yield). $^1$H NMR (400 MHz, Chloroform-*d*) δ 7.46 – 7.39 (m, 2H), 7.38 – 7.24 (m, 6H), 7.20 – 7.11 (m, 2H), 3.48 (ddt, $J$ = 14.1, 7.0, 3.7 Hz, 1H), 3.42 – 3.29 (m, 2H), 3.24 (dq, $J$ = 14.0, 7.1 Hz, 1H), 1.98 (s, 3H), 1.16 – 1.07 (m, 6H). $^{13}$C NMR (101 MHz, Chloroform-*d*) δ 200.35 (d, J = 5.1 Hz), 162.13 (t, $J$ = 29.2 Hz), 137.64 (d, $J$ = 3.3 Hz), 136.38 (d, $J$ = 3.7 Hz), 131.53, 129.38, 129.04, 128.51, 128.36, 127.99, 118.35 (dd, $J$ = 272.0, 257.2 Hz), 60.73 (dd, $J$ = 21.7, 17.6 Hz), 42.05 (dd, J = 9.1, 5.1 Hz), 41.96, 21.62 (dd, $J$ = 6.0, 3.0 Hz), 14.39, 12.44. $^{19}$F NMR (376 MHz, Chloroform-*d*) δ -96.17 (d, $J$ = 283.7 Hz, 1F), -104.81 (d, $J$ = 283.6 Hz, 1F). HRMS (ESI) (*m/z*): [M+Na]$^+$ calcd. for $C_{21}H_{23}O_2F_2NNa$: 382.1589, found: 382.1593.

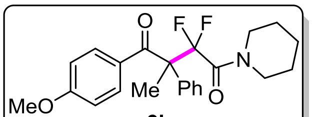

**3ba**

2,2-difluoro-4-(4-methoxyphenyl)-3-methyl-3-phenyl-1-(piperidin-1-yl)butane-1,4-dione (**3ba**). The product **3ba** is purified with silica gel chromatography (petroleum ether/ethyl acetate = 10/1 as the eluent) as yellow oil (88% yield). $^1$H NMR (400 MHz, Chloroform-*d*) δ 7.52 – 7.44 (m, 2H), 7.42 – 7.36 (m, 5H), 6.72 (d, $J$ = 8.6 Hz, 2H), 3.77 (s, 3H), 3.71 – 3.42 (m, 4H), 2.11 (s, 3H), 1.64 (m, 6H). $^{13}$C NMR (101 MHz, Chloroform-*d*) δ 198.08 (d, $J$ = 5.2 Hz), 162.35, 161.17 (t, $J$ = 29.0 Hz), 136.94 (d, $J$ = 2.7 Hz), 131.83, 129.51, 129.43 (d, $J$ = 3.4 Hz), 128.38, 128.26, 118.42 (dd, $J$ = 271.6, 256.8 Hz), 113.23, 60.69 (dd, $J$ = 21.4, 17.5 Hz), 55.42, 47.14 (dd, $J$ = 9.8, 5.4 Hz), 44.83, 26.59, 25.85, 24.67, 21.67 (dd, $J$ = 5.8, 3.3 Hz). $^{19}$F NMR (376 MHz, Chloroform-*d*) δ -95.74 (d, $J$ = 285.7 Hz, 1F), -104.48 (d, $J$ = 285.7 Hz, 1F). HRMS (ESI) (m/z): [M+Na]$^+$ calcd. for $C_{23}H_{25}O_3F_2NNa$: 424.1695, found: 424.1700.

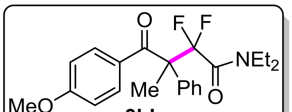

**3bb**

N,N-diethyl-2,2-difluoro-4-(4-methoxyphenyl)-3-methyl-4-oxo-3-phenylbutanamide (**3bb**). The product **3bb** is purified with silica gel chromatography (petroleum ether/ethyl acetate = 10/1 as the eluent) as yellow oil (85% yield). $^1$H NMR (400 MHz, Chloroform-*d*) δ 7.51 – 7.43 (m, 2H), 7.42 – 7.26 (m, 5H), 6.72 (d, $J$ = 8.5 Hz, 2H), 3.77 (s, 3H), 3.63 – 3.26 (m, 4H), 2.12 (s, 3H), 1.22 - 1.15 (m, 6H). $^{13}$C NMR (101 MHz, Chloroform-*d*) δ 198.26 (d, $J$ = 5.2 Hz), 162.44 (t, $J$ = 29.1 Hz). 162.39, 137.17 (d, $J$ = 3.6 Hz), 131.87, 129.48, 129.35 (d, $J$ = 3.2 Hz), 128.39, 128.25, 118.20 (dd, $J$ = 271.4, 256.2 Hz), 113.23, 61.05 (dd, $J$ = 21.8, 18.0 Hz), 55.42, 42.16 (dd, $J$ = 10.1, 5.1 Hz), 42.09, 21.89 (dd, $J$ = 6.0, 3.1 Hz), 14.45, 12.45. $^{19}$F NMR (376 MHz, Chloroform-*d*) δ -96.72 (d, $J$ = 282.3 Hz, 1F), -105.34 (d, $J$ = 282.3 Hz, 1F). HRMS (ESI) (m/z): [M+Na]$^+$ calcd. for $C_{22}H_{25}O_3F_2NNa$: 412.1695, found: 412.1700.

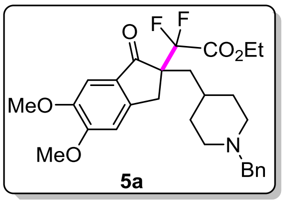

Ethyl (S)-2-(2-((1-benzylpiperidin-4-yl)methyl)-5,6-dimethoxy-1-oxo-2,3-dihydro-1H-inden-2-yl)-2,2-difluoroacetate (**5a**). The product **5a** is purified with silica gel chromatography (petroleum ether/ethyl acetate = 1/1 as the eluent) as white solid (73% yield). $^{1}$H NMR (400 MHz, Chloroform-*d*) δ 7.34 – 7.18 (m, 5H), 7.15 (s, 1H), 6.86 (s, 1H), 4.19 (q, *J* = 7.1 Hz, 2H), 3.98 (s, 3H), 3.90 (s, 3H), 3.53 – 3.34 (m, 3H), 3.04 (d, *J* = 17.8 Hz, 1H), 2.72 (dd, *J* = 23.2, 11.3 Hz, 2H), 2.08 (dt, *J* = 17.6, 8.8 Hz, 1H), 1.94 – 1.67 (m, 4H), 1.50 (d, *J* = 12.5 Hz, 1H), 1.37 (t, *J* = 11.1 Hz, 2H), 1.28 – 1.15 (m, 4H). $^{13}$C NMR (101 MHz, Chloroform-*d*) δ 200.72 (t, *J* = 2.7 Hz), 162.98 (t, *J* = 32.8 Hz), 156.15, 149.89, 147.76, 138.35, 129.31, 128.22, 127.03, 116.22 (t, *J* = 259.2 Hz), 107.12, 104.61, 63.39, 63.12, 57.75 (t, *J* = 20.5 Hz), 56.44, 56.24, 53.68, 53.60, 37.34, 34.11, 33.60 (t, *J* = 3.2 Hz), 33.50, 32.22, 13.88. $^{19}$F NMR (376 MHz, Chloroform-*d*) δ -109.11 (d, *J* = 257.5 Hz, 1F), -110.24 (d, *J* = 257.4 Hz, 1F). HRMS (ESI) (*m/z*): $[M+H]^+$ calcd. for $C_{28}H_{34}F_2NO_5$: 502.2400, found: 502.2419.

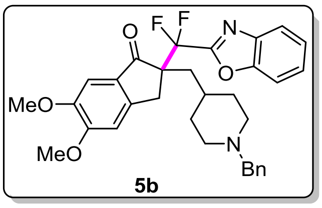

(S)-2-(benzo[d]oxazol-2-yldifluoromethyl)-2-((1-benzylpiperidin-4-yl)methyl)-5,6-dimethoxy-2,3-dihydro-1H-inden-1-one (**5b**). The product **5b** is purified with silica gel chromatography (petroleum ether/ethyl acetate = 1/1 as the eluent) as white solid (50% yield). $^{1}$H NMR (400 MHz, Chloroform-*d*) δ 7.68 (d, *J* = 7.7 Hz, 1H), 7.49 (d, *J* = 7.9 Hz, 1H), 7.31 (dt, *J* = 15.8, 7.4 Hz, 2H), 7.16 (m, 5H), 7.06 (s, 1H), 6.77 (s, 1H), 3.87 (s, 3H), 3.80 (s, 3H), 3.60 (d, *J* = 17.7 Hz, 1H), 3.31 (s, 2H), 3.08 (d, *J* = 17.8 Hz, 1H), 2.64 (dd, *J* = 27.6, 11.7 Hz, 2H), 2.19 (dd, *J* = 14.1, 6.3 Hz, 1H), 1.93 (dd, *J* = 14.2, 3.4 Hz, 1H), 1.69 (dt, *J* = 43.1, 12.0 Hz, 2H), 1.43 (d, *J* = 12.4 Hz, 1H), 1.38 – 1.06 (m, 4H). $^{13}$C NMR (101 MHz, Chloroform-*d*) δ 200.50, 156.80 (t, *J* = 34.1 Hz), 156.12, 150.42, 149.79, 147.87, 140.01, 138.30, 129.46, 129.26, 128.19, 127.01, 126.77, 125.26, 121.32, 117.44 (t, *J* = 251.5 Hz), 111.43, 107.08, 104.67, 63.30, 58.86 (t, *J* = 21.5 Hz), 56.37, 56.17, 53.64, 53.56, 37.56, 34.00, 33.95, 33.44, 32.24; $^{19}$F NMR (376 MHz, Chloroform-*d*) δ -101.79 (d, *J* = 272.1 Hz, 1F), -105.06 (d, *J* = 272.2 Hz, 1F). HRMS (ESI) (m/z): $[M+H]^+$ calcd. for $C_{32}H_{33}F_2N_2O_4$: 547.2408, found: 547.2411.

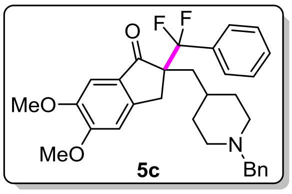

(S)-2-((1-benzylpiperidin-4-yl)methyl)-2-(difluoro(phenyl)methyl)-5,6-dimethoxy-2,3-dihydro-1H-inden-1-one (**5c**). The product **5c** is purified with silica gel chromatography (petroleum ether/ethyl acetate = 1/1 as the eluent) as white solid (61% yield). $^{1}$H NMR (400 MHz, Chloroform-*d*) δ 7.45 – 7.16 (m, 10H), 7.06 (s, 1H), 6.74 (s, 1H), 3.92 (s, 3H), 3.87 (s, 3H), 3.43 (d, *J* = 17.8 Hz, 1H), 3.38 (s, 2H), 3.02 (d, *J* = 17.8 Hz, 1H), 2.82 – 2.56 (m, 2H), 2.10 (dd, *J* = 14.0, 6.5 Hz, 1H), 1.97 – 1.85 (m, 2H), 1.79 (td,

$J$ = 11.5, 2.8 Hz, 1H), 1.68 (td, $J$ = 11.2, 2.8 Hz, 1H), 1.47 (dt, $J$ = 13.2, 2.8 Hz, 1H), 1.29 – 1.13 (m, 3H). $^{13}$C NMR (101 MHz, Chloroform-*d*) δ 202.12 (dd, $J$ = 3.4, 2.1 Hz), 155.84, 149.61, 147.90, 138.43, 134.68, 134.41, 134.14, 130.11, 129.94, 129.28, 128.18, 127.72, 126.98, 126.91, 126.84, 122.76 (dd, $J$ = 253.5, 251.6 Hz), 106.86, 104.41, 63.36, 59.85 (t, $J$ = 24.5 Hz), 56.32, 56.15, 53.71, 53.64, 37.99, 34.20, 33.77, 33.46, 32.41. $^{19}$F NMR (376 MHz, Chloroform-*d*) δ -97.98 (d, $J$ = 246.8 Hz, 1F), -100.21 (d, $J$ = 246.8 Hz, 1F). HRMS (ESI) (m/z): $[M+H]^+$ calcd. for $C_{31}H_{34}F_2NO_3$: 506.2507, found: 506.2500.

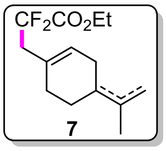


Ethyl 2,2-difluoro-3-(4-(propan-2-ylidene)cyclohex-1-en-1-yl)propanoate and Ethyl 2,2-difluoro-3-(4-(prop-1-en-2-yl)cyclohex-1-en-1-yl)propanoate (**7**). The product **7** is purified with silica gel chromatography (petroleum ether/ethyl acetate = 20/1 as the eluent) as yellow oil (12% yield). $^1$H NMR (400 MHz, Chloroform-*d*) δ 5.60 (d, $J$ = 14.2 Hz, 1.69H), 4.74 – 4.65 (m, 2H), 4.37 – 4.23 (m, 3.27H), 2.79 – 2.62 (m, 4.72H), 2.29 (t, $J$ = 6.2 Hz, 1.32H), 2.11 (dd, $J$ = 16.8, 4.9 Hz, 5.60H), 1.95 (d, $J$ = 11.7 Hz, 1.05H), 1.80 (ddq, $J$ = 12.6, 5.2, 2.5 Hz, 1.14H), 1.72 (s, 2.97H), 1.67 (s, 1.84H), 1.63 (s, 1.88H), 1.52 – 1.39 (m, 1.22H), 1.35 (s, 0.79H), 1.33 (d, $J$ = 1.4 Hz, 1.87H), 1.32 (d, $J$ = 1.4 Hz, 1.60H), 1.30 (s, 0.74H). $^{13}$C NMR (101 MHz, Chloroform-*d*) δ 164.32 (td, $J$ = 32.8, 4.7 Hz), 149.63, 128.78 (t, $J$ = 4.0 Hz), 128.55, 128.44, 128.37 (t, $J$ = 4.1 Hz), 126.41, 122.58, 118.75, 116.24, 113.73, 108.88, 62.78, 42.84 (td, $J$ = 23.1, 6.8 Hz), 40.58, 31.00, 30.39, 29.83, 29.61, 27.79, 26.63, 20.89, 20.29, 19.88, 14.17, 14.10. $^{19}$F NMR (376 MHz, Chloroform-*d*) δ -103.12, -103.24, -103.34. HRMS (ESI) (*m/z*): $[M+H]^+$ calcd. for $C_{14}H_{21}F_2O_2$: 259.1510, found: 259.1506.

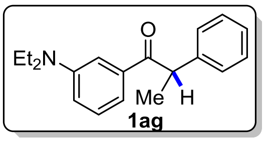


(S)-1-(3-(diethylamino)phenyl)-2-phenylpropan-1-one (**1ag**). The product **1ag** was purified with silica gel chromatography (petroleum ether/ethyl acetate = 10/1 as the eluent) as yellow oil (63% yield). $^1$H NMR (400 MHz, Chloroform-*d*) δ 7.38 – 7.14 (m, 8H), 6.83 (dd, $J$ = 8.1, 2.1 Hz, 1H), 4.68 (q, $J$ = 6.9 Hz, 1H), 2.93 (s, 6H), 1.52 (d, $J$ = 6.9 Hz, 3H). $^{13}$C NMR (101 MHz, Chloroform-*d*) δ 201.13, 150.62, 141.99, 137.31, 129.12, 129.03, 127.86, 126.88, 117.22, 116.87, 112.36, 48.10, 40.62, 19.72. HRMS (ESI) (m/z): $[M+H]^+$ calcd. for $C_{17}H_{20}NO$: 254.1545, found: 254.1535.

**General Procedure for Cobalt-Catalyzed Difluoroalkyaltion of Secondary Aryl Ketones.**

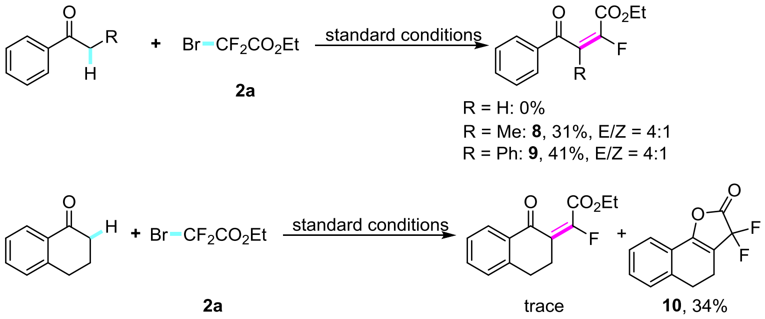


To a 50 mL of Schlenk tube was added secondary aryl ketone (1.0 equiv, 0.2 mmol), $CoBr_2$ (10 mol %, 0.02 mmol) and dppBz (10 mol %, 0.02 mmol) under air, followed by Zn (0.5 equiv, 0.1 mmol). The

mixture was evacuated and backfilled with $N_2$ (3 times). THF (2 mL) was added then followed by LDA (105 mol%, 0.21 mmol) subsequently. The Schlenk tube was then sealed with a Teflon lined cap and put into a cooled bath (- 10°C). After stirring for 5 minutes, bormdifluoroacetate **2a** (3.0 equiv, 0.6 mmol) was added to the reaction mixture, and the Schlenk tube was then resealed with a Teflon lined cap and put back into the cooled bath (- 10°C). After stirring for another 12 hours, the reaction mixture was diluted with ethyl acetate (5 mL). The solvent was removed under reduced pressure, and the residue was purified by flash column chromatography on silica gel to give the desired product.

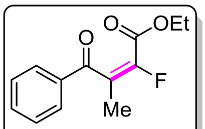

**8** (E/Z = 4:1)

Ethyl 2-fluoro-3-methyl-4-oxo-4-phenylbut-2-enoate (**8**). The product **8** was purified with silica gel chromatography (petroleum ether/ethyl acetate = 10/1 as the eluent) as yellow oil (31% yield, E/Z = 4:1). The definite stereo-structure of the product was deducted from the previous report.[12] (E)-$^{1}$H NMR (400 MHz, Chloroform-*d*) δ 7.90 (d, *J* = 7.8 Hz, 2H), 7.69 – 7.56 (m, 1H), 7.55 – 7.41 (m, 2H), 4.07 (q, *J* = 7.1 Hz, 2H), 2.11 (d, *J* = 3.4 Hz, 3H), 1.02 (t, *J* = 7.1 Hz, 3H). (E)-$^{19}$F NMR (376 MHz, Chloroform-*d*) δ -127.29. (E)-$^{13}$C NMR (101 MHz, Chloroform-*d*) δ 193.71 (d, *J* = 6.2 Hz), 159.68 (d, *J* = 35.5 Hz), 146.18, 143.56, 135.15 (d, *J* = 3.7 Hz), 133.96, 129.03, 128.99, 62.21, 14.69 (d, *J* = 5.7 Hz), 13.70. HRMS (ESI) (m/z): $[M+Na]^+$ calcd. for $C_{13}H_{13}FO_3Na$: 259.0746, found: 259.0744.

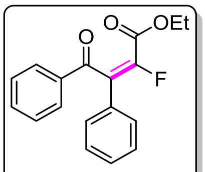

**9** (E/Z = 4:1)

Ethyl 2-fluoro-4-oxo-3,4-diphenylbut-2-enoate (**9**). The product **9** was purified with silica gel chromatography (petroleum ether/ethyl acetate = 10/1 as the eluent) as yellow oil (41% yield, E/Z = 4:1). $^{1}$H NMR (400 MHz, Chloroform-*d*) δ 8.11 – 7.92 (m, 2H), 7.65 – 7.31 (m, 8H), 4.27 – 4.04 (m, 2H), 1.19 – 1.03 (m, 3H). $^{19}$F NMR (376 MHz, Chloroform-*d*) δ -116.84, -126.46. $^{13}$C NMR (101 MHz, Chloroform-*d*) δ 191.80 (d, *J* = 3.6 Hz), 191.42 (d, *J* = 5.3 Hz), 160.45 (d, *J* = 34.8 Hz), 160.03 (d, *J* = 36.4 Hz), 145.99, 145.57, 143.41, 142.85, 136.26 (d, *J* = 3.8 Hz), 134.97, 134.51, 133.83, 131.47 (d, *J* = 6.0 Hz), 130.97 (d, *J* = 5.6 Hz), 130.41 (d, *J* = 1.5 Hz), 130.16 – 129.90 (m), 129.79, 129.33, 129.09, 129.06, 129.05, 128.99, 128.95, 128.69 (d, *J* = 3.0 Hz), 128.48, 62.53, 62.15, 13.79, 13.76. HRMS (ESI) (m/z): $[M+Na]^+$ calcd. for $C_{18}H_{15}FO_3Na$: 321.0903, found: 321.0894.

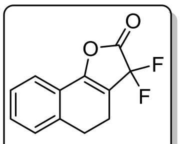

**10**

3,3-difluoro-4,5-dihydronaphtho[1,2-b]furan-2(3H)-one (**10**). The product **10** was purified with silica gel chromatography (petroleum ether/ethyl acetate = 10/1 as the eluent) as yellow oil (34% yield). $^{1}$H NMR (400 MHz, Chloroform-*d*) δ 8.05 – 7.90 (m, 1H), 7.54 – 7.45 (m, 1H), 7.41 – 7.33 (m, 1H), 7.26 – 7.21 (m, 1H), 2.97 – 2.85 (m, 2H), 2.85 – 2.74 (m, 2H). $^{19}$F NMR (376 MHz, Chloroform-*d*) δ -58.07. $^{13}$C NMR (101 MHz, Chloroform-*d*) δ 180.21, 141.55, 133.57, 129.69, 127.96, 127.33, 126.47, 113.29,

102.29, 27.80, 21.70 (t, $J$ = 4.0 Hz). HRMS EI (m/z): $[M]^+$ calcd. for $C_{12}H_8F_2O_2$: 222.0492, found: 222.0486.

# Mechanistic Studies

## 1. Radical clock experiment:

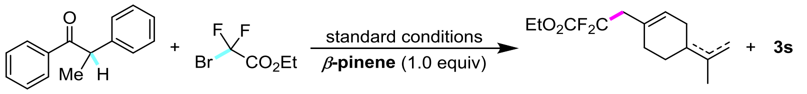


1s 2a 7, 12% (5 : 3) 35%

To a 50 mL of Schlenk tube was added ketone **1s** (1.0 equiv, 0.2 mmol), $CoBr_2$ (10 mol %, 0.02 mmol), Zn (50 mol%, 0.1 mmol) and dppBz (10 mol %, 0.02 mmol) under air. The mixture was evacuated and backfilled with $N_2$ (3 times). THF (2 mL) was added then followed by LDA (105 mol%, 0.21 mmol) subsequently. The Schlenk tube was then sealed with a Teflon lined cap and put into a cooled bath (-10 ℃). After stirring for 5 minutes, bormdifluoroacetate **2a** (3.0 equiv, 0.6 mmol) and **β-pinene** (1.0 equiv, 0.2 mmol), were added to the reaction mixture, and the Schlenk tube was then resealed with a Teflon lined cap and put back into the cooled bath (- 10 ℃). After stirring for another 12 hours, the reaction mixture was diluted with ethyl acetate (5 mL). The solvent was removed under reduced pressure, and the residue was purified by flash column chromatography on silica gel to give the desired product of **7** in 12% yield (5 : 3 isomer ratio).

## 2. Procedure of difluoroalkylation with Co(I) used as the catalyst:

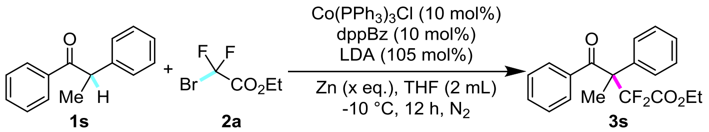


1s 2a 3s

| entry | x | yield/% |
|---|---|---|
| 1 | 0 | 70 |
| 2 | 0.5 | 71 |

To a 50 mL of Schlenk tube was added ketone **1s** (1.0 equiv, 0.2 mmol), $Co(PPh_3)_3Cl$ (10 mol%, 0.02 mmol) and dppBz (10 mol %, 0.02 mmol) under air, then was added with Zn (entry 1) or not (entry 2). THF (2 mL) was added then followed by LDA (105 mol%, 0.21 mmol) subsequently. The Schlenk tube was then sealed with a Teflon lined cap and put into a cooled bath (- 10 ℃). After stirring for 5 minutes, bormdifluoroacetate **2a** (3.0 equiv, 0.6 mmol) was added to the reaction mixture, and the Schlenk tube was then resealed with a Teflon lined cap and put back into the cooled bath (- 10 ℃). After stirring for another 12 hours, the reaction mixture was diluted with ethyl acetate (5 mL). The solvent was removed under reduced pressure, and the residue was purified by flash column chromatography on silica gel to give the desired product of **3s**.

## 3. Control experiments for the generation of difluoroalkyl radical:

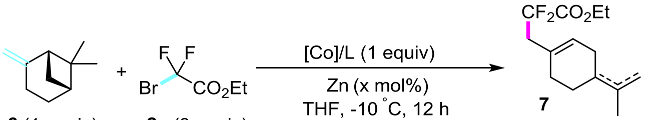


**6** (1 equiv) **2a** (3 equiv) **7**

$CoBr_2$/dppBz, x = 200, 65 %, 5:3 (1)
$CoBr_2$/dppBz, x = 0, 0 % (2)
$Co(PPh_3)_3Cl$, x = 0, 53 %, 5:3 (3)

To a 50 mL of Schlenk tube was added **6** (1.0 equiv, 0.2 mmol), $CoBr_2$ (1.0 equiv, 0.2 mmol) and dppBz (1.0 equiv, 0.2 mmol) under air, then was added with Zn (Eq. 1). THF (2 mL) was added subsequently. The Schlenk tube was then sealed with a Teflon lined cap and put into a cooled bath (- 10 ℃). After stirring for 12 hours, the reaction mixture was diluted with ethyl acetate (5 mL). The solvent was removed under reduced pressure, and the residue was purified by flash column chromatography on silica gel to give the desired product of **7**. To a 50 mL of Schlenk tube was added **6** (1.0 equiv, 0.2 mmol), $CoBr_2$ (1.0 equiv, 0.2 mmol) and dppBz (1.0 equiv, 0.2 mmol) under air (Eq. 2). THF (2 mL) was added subsequently. The Schlenk tube was then sealed with a Teflon lined cap and put into a cooled bath (- 10 ℃). After stirring for 12 hours, the reaction mixture was diluted with ethyl acetate (5 mL). The solvent was removed under reduced pressure, and the residue was purified by flash column chromatography on silica gel not to give the desired product of **7**. To a 50 mL of Schlenk tube was added **6** (1.0 equiv, 0.2 mmol), $CoCl(PPh_3)_3$ (1.0 equiv, 0.2 mmol) and dppBz (1.0 equiv, 0.2 mmol) under air (Eq. 3). THF (2 mL) was added subsequently. The Schlenk tube was then sealed with a Teflon lined cap and put into a cooled bath (- 10 ℃). After stirring for 12 hours, the reaction mixture was diluted with ethyl acetate (5 mL). The solvent was removed under reduced pressure, and the residue was purified by flash column chromatography on silica gel to give the desired product of **7**.

## 4. The Operation of an Initial Transmetallation:

To a 50 mL of Schlenk tube was added $Co(PPh_3)_3Cl$ (1.0 equiv, 0.2 mmol) and dppBz (1.0 equiv, 0.2 mmol) under air, then was added by **1a** (1.0 equiv, 0.2 mmol). THF (2 mL) was added then followed by LDA (105 mol%, 0.21 mmol) subsequently. The Schlenk tube was then sealed with a Teflon lined cap and put into a cooled bath (- 10 ℃). After stirring for 5 minutes, bormdifluoroacetate **2a** (3.0 equiv, 0.6 mmol) was added to the reaction mixture, and the Schlenk tube was then resealed with a Teflon lined cap and put back into the cooled bath (- 10 ℃). After stirring for another 10 minutes, the reaction mixture was diluted with ethyl acetate (5 mL). The solvent was removed under reduced pressure, and the residue was purified by flash column chromatography on silica gel to give the desired product of **3a**. To another 50 mL of Schlenk tube was added $Co(PPh_3)_3Cl$ (1.0 equiv, 0.2 mmol) and dppBz (1.0 equiv, 0.2 mmol) under air, then was added by bormdifluoroacetate **2a** (3.0 equiv, 0.6 mmol) and THF (2 mL). The Schlenk tube was then sealed with a Teflon lined cap and put into a cooled bath (- 10 ℃). After stirring for 5 minutes, **1a** (1.0 equiv, 0.2 mmol) and LDA (105 mol%, 0.21 mmol) were added to the reaction mixture, and the Schlenk tube was then resealed with a Teflon lined cap and put back into the cooled bath (- 10 ℃). After stirring for another 10 minutes, the reaction mixture was diluted with ethyl acetate (5 mL). The solvent was removed under reduced pressure, and the residue was purified by flash column chromatography on silica gel to give the desired product of **3a.**

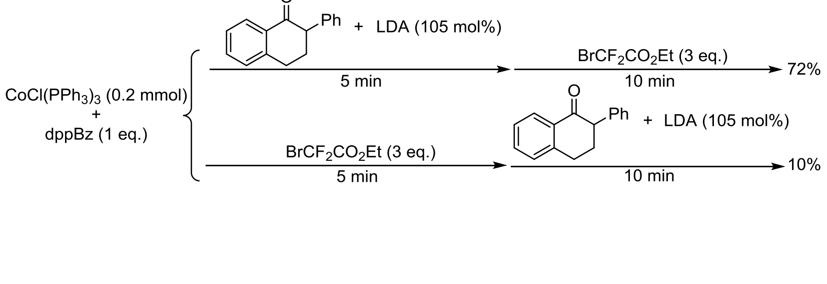

## Supplementary References:


(1) Rao, B., Tang, J. & Zeng, X. Synthesis of 2-Benzylphenyl Ketones by Aryne Insertion into Unactivated C–C Bonds. *Org. Letter.* **18**, 1678-1681 (2016).

(2) Zhang, Y.-M., Li, M., Li, W., Huang, Z., Zhu, S., Yang, B., Wang, X.-C. & Zhang, S. X.-A. A new class of "electro-acid/base"-induced reversible methyl ketone colour switches. *J. Mater. Chem. C.* **1**, 5309-5314 (2013).

(3) Li, Z. & Gevorgyan, V. Double Duty for Cyanogen Bromide in a Cascade Synthesis of Cyanoepoxides. *Angew. Chem. Int. Ed.* **50**, 2808-2810 (2011).

(4) Campbell, N. & Ciganek, E. A new synthesis of 3-hydroxy-1 : 2-benzofluorene. *J. Chem. Soc.* **0**, 3834-3836 (1956).

(5) Takeda, T. & Terada, M. Synthesis of Bulky Aryl Group-substituted Chiral Bis(guanidino)iminophosphoranes as Uncharged Chiral Organosuperbase Catalysts. *Aust. J. Chem*. **67**, 1124-1128 (2014).

(6) Hino, K., Nagai, Y., Uno, H., Masuda, Y., Oka, M. & Karasawa, T. A novel class of potential central nervous system agents. 3-Phenyl-2-(1-piperazinyl)-5H-1-benzazepines. *J. Med. Chem.* **31**, 107-117 (1988).

(7) Takeda, T. & Terada, M. Development of a Chiral Bis(guanidino)iminophosphorane as an Uncharged Organosuperbase for the Enantioselective Amination of Ketones. *J. Am. Chem. Soc.* **135**, 15306-15309 (2013).

(8) Dolbier Jr, W. R., Burkholder, C. R. & Médebielle, M. Syntheses of 2-(bromodifluoromethyl)benzoxazole and 5-(bromodifluoromethyl)-1,2,4-oxadiazoles. *J. Fluorine Chem.* **95**, 127-130 (1999).

(9) Maximilian, D. *et al. PCT Int. Appl.*, 2013092850, 27 Jun 2013.

(10) Prakash, G. K. S., Hu, J., Simon, J., Bellew, D. R. & Olah, G. A. Preparation of α,α-difluoroalkanesulfonic acids. *J. Fluorine Chem.* **125**, 595-601 (2004).

(11) Tarui, A., Shinohara, S., Sato, K., Omote, M. & Ando, A. Nickel-Catalyzed Negishi Cross-Coupling of Bromodifluoroacetamides. *Org. Letter.* **18**, 1128-1131 (2016).

(12) Li, Y., Liu, J., Zhao, S., Du, X., Guo, M., Zhao, W., Tang, X. & Wang, G. Copper-Catalyzed Fluoroolefination of Silyl Enol Ethers and Ketones toward the Synthesis of β−Fluoroenones. *Org. Lett.* **20**, 917−920 (2018).
